# Phylogeny Reconstruction with Alignment-Free Method That Corrects for Horizontal Gene Transfer

**DOI:** 10.1371/journal.pcbi.1004985

**Published:** 2016-06-23

**Authors:** Raquel Bromberg, Nick V. Grishin, Zbyszek Otwinowski

**Affiliations:** 1 Department of Biophysics and Department of Biochemistry, University of Texas Southwestern Medical Center at Dallas, Dallas, Texas, United States of America; 2 Howard Hughes Medical Institute, University of Texas Southwestern Medical Center at Dallas, Dallas, Texas, United States of America; Gotingen University, UNITED STATES

## Abstract

Advances in sequencing have generated a large number of complete genomes. Traditionally, phylogenetic analysis relies on alignments of orthologs, but defining orthologs and separating them from paralogs is a complex task that may not always be suited to the large datasets of the future. An alternative to traditional, alignment-based approaches are whole-genome, alignment-free methods. These methods are scalable and require minimal manual intervention. We developed SlopeTree, a new alignment-free method that estimates evolutionary distances by measuring the decay of exact substring matches as a function of match length. SlopeTree corrects for horizontal gene transfer, for composition variation and low complexity sequences, and for branch-length nonlinearity caused by multiple mutations at the same site. We tested SlopeTree on 495 bacteria, 73 archaea, and 72 strains of *Escherichia coli* and *Shigella*. We compared our trees to the NCBI taxonomy, to trees based on concatenated alignments, and to trees produced by other alignment-free methods. The results were consistent with current knowledge about prokaryotic evolution. We assessed differences in tree topology over different methods and settings and found that the majority of bacteria and archaea have a core set of proteins that evolves by descent. In trees built from complete genomes rather than sets of core genes, we observed some grouping by phenotype rather than phylogeny, for instance with a cluster of sulfur-reducing thermophilic bacteria coming together irrespective of their phyla. The source-code for SlopeTree is available at: http://prodata.swmed.edu/download/pub/slopetree_v1/slopetree.tar.gz.

## Author Summary

Due to their lack of distinct morphological features, bacteria and archaea were extremely difficult to classify until technology was developed to obtain their DNA sequences; these sequences could then be compared to estimate evolutionary relationships. Now, due to technological advances, there is a flood of available sequences from a wide variety of organisms. These advances have spurred the development of algorithms which can estimate evolutionary relationships using whole genomes, in contrast to the more traditional methods which used single genes earlier and now typically use groups of conserved genes. However, there are many challenges when attempting to infer evolutionary relationships, in particular horizontal gene transfer, where DNA is transferred from one organism to another, resulting in an organism’s genome containing DNA that does not reflect its evolution by descent. We developed a new whole-genome method for estimating evolutionary distances which identifies and corrects for horizontal transfer. We found that for SlopeTree and all other whole-genome methods we applied, horizontal transfer causes some evolutionary distances to be grossly underestimated, and that our correction corrects for this.

## Introduction

Learning how to obtain complete genomes was a critical step to understanding biology and was achieved as early as 1977 for the genome of bacteriophage *ɸ*X174 [1]. Since then, methods for obtaining full genome sequences have advanced tremendously [2–4], leading to a second critical transition, when the number of genome sequences became too large for traditional, alignment-based, phylogenetics [5–8]. Even during the time of Sanger sequencing, the number of bacterial genomes began to cross this threshold [9]. With the development of next generation sequencing technology, we are experiencing a flood of complete genomes and metagenomes [10].

Molecular phylogenetics enabled the classification of prokaryotic organisms. In 1977, a multiple sequence alignment (MSA) of the small subunit (SSU) 16S rRNA gene revealed the existence of the three domains of life [11], making the SSU rRNA the gold standard for phylogenetics [12–14]. As more sequences became available, additional genes were used as phylogenetic markers, including protein elongation factors EF-α/Tu and EF-2 [15–17], chaperones Hsp60 and Hsp70 [18, 19], the largest subunits of the RNA polymerase [20, 21], RecA [22], a variety of aminoacyl-tRNA synthetases [23] and others. Approaches using single genes originally generated a wealth of phylogenetic insight, but these trees were frequently incongruent with one another [24, 25]. To improve the accuracy of phylogenetic methods, phylogeneticists began to concatenate multiple conserved genes to produce larger MSAs and therefore better resolved trees [25–28]. The size and functional diversity of these gene groups is largely dependent on the number and diversity of taxa [29]. For instance, in the recent work of Lang and Eisen [25], an analysis of ~900 diverse prokaryotes from both bacteria and archaea identified only 24 suitable (i.e. paralog-free) genes. These consisted of a subset of ribosomal proteins, two translation factors that both interact with the ribosome, and the alpha subunit of a phenylalanyl-tRNA synthetase which was the only protein in the set not interacting with the ribosome and which contributed only ~5% of the overall alignment used to generate phylogeny. A similar situation was seen by Ciccarelli et al., in which for a group of 191 organisms, the set of 31 genes used in the final alignment consisted of 23 ribosomal proteins [30]. Widespread horizontal gene transfer (HGT) also interferes with a straightforward definition of evolution by descent [31–34]. Therefore, we are still making our way to a consensus to a definition of prokaryotic evolution by descent.

In contrast to the majority of traditional MSA-approaches, which often require extensive curation to produce high quality alignments of orthologs, alignment-free methods are scalable and require minimum manual intervention [35–38]. The idea of using complete genomes to perform phylogeny has a long history [39], but lay dormant until enough complete genomes became available. The rate at which these methods are now appearing reflects the pressing need for unsupervised, scalable methods. An additional advantage is that because they use complete genomes, they may provide a more sound approximation for organismal phylogeny [40]. Alignment-free methods compute similarity or distance metrics using a variety of statistical properties belonging to k-mers (fixed-length substrings, also called n-grams, n-mers, k-tuples, and k-words) in genomes. These methods are often divided into two main classes: methods using fixed-length word counts and methods using match lengths.

Word count methods relying on exact word matches include Composition Vector Trees (CVTrees) [41, 42], Feature Frequency Profiles (FFP) [43], and D2 statistics [44–46]. Each of these methods relies on different properties of counting exact matches fixed-length k-mers. CVTree calculates the frequency of all length-*k* k-mers in all proteomes; these frequency or composition vectors, after a background subtraction correcting for random neutral mutations, are then compared to one another and a correlation is calculated by means of the cosine of the angle between them, which is normalized to produce the final value. FFP tabulates the counts for all possible features in the genome of fixed length *k*, which as in CVTree also are passed through a normalization procedure to form a probability distribution vector (i.e. an FFP); distances are then calculated using Jensen-Shannon Divergence [47]. D2 measures sequence dissimilarity by the logarithm of the ratio between conserved and non-conserved k-mers. Word count methods employing inexact matches include Co-phylog [48] and Spaced Word Frequencies (SWF) [49, 50]. Co-phylog identifies seed alignments (exact or approximate matches) between the query and subject sequences and then extends them into longer alignments (i.e. ‘micro-alignments’); this method has an additional advantage in that it runs on raw next-generation sequencing data. SWF is similar to Co-phylog, using a mask consisting of positions that are either *match* or *don’t care*, and using the frequencies of these spaced words, with the *don’t care* positions ignored according to the specified pattern.

Match length methods can also be divided into those allowing zero mismatches or those allowing some number of mismatches. Exact match length methods include Average Common Substring (ACS) [51], Kr [52], and Underlying Approach (UA) [53]. Conceptually, the ACS method is the most similar to the method we present, and calculates its distance metric by means of variable length, exact matches between genomes or proteomes. For every position in one genome or proteome, ACS finds the longest length match in the other. This list of matches is then averaged, normalized, and a correction applied that transforms it from a similarity measure to a distance. The Kr method is closely related to the ACS method; taking two unaligned DNA sequences, Kr estimates the number of substitutions per site by determining for every suffix present in the one sequence the shortest prefix that is absent from the other (called shustrings). UA uses a scoring function on matching statistics between unique, independent subwords. k-Mismatch ACS (kmacs) [49] is an extension of the ACS approach which approximates the number of substring matches with up to *k* mismatches. Another, more recent extension of the ACS approach is ALFRED-G, also capable of computing lengths of shared sequences with mismatches allowed [54]. ACS-like methods which allow for *k*>1 mismatches can be highly costly in computational terms, but there has recently been some headway in improving their efficiency [55].

We present SlopeTree, a new alignment-free method which measures evolutionary distance by quantifying how quickly the number of matching sequences between two proteomes decays as a function of sequence length. The sequences that we employ to this end are k-mers, i.e. substrings of length *k*. The method considers uneven composition of amino acids, the possibility of backwards mutations, a background of coincidental matches over short k-mer lengths, and the issue of HGT. HGT is highly relevant for alignment-free methods because it adds a spurious contribution of similarity between genomes [56, 57]. There are multiple possible signatures of horizontally transferred proteins, for instance unusual codon usage [58–60]. We identified a novel signature based on analysis of multiple copies of almost identical protein sequences in a genome, and those multiple copies almost invariably belonged to one of two categories: one category was of EF-Tu translation factor, which is frequently present in multiple copies; and the second was of mobile elements, as inferred from a very narrow or scattered phylogenetic footprint, even within a single species. When annotated, these mobile elements consisted primarily of parasitic proteins resulting from phage infections. Another level of filtering is done by means of a dual evolutionary stability index indicating conservation and lack of stability, i.e. a *paralogy score*, with a large instability value representing very likely cases of HGT. A mobile element (ME) filter and a separate, conservation filter were built into SlopeTree using the earlier mentioned novel signature and the paralogy score. To measure the similarity between two proteomes, SlopeTree yields a *slope* (explained in the Algorithms section); a third HGT correction is based on the curvature of this slope. Therefore, SlopeTree is unique in that it is not only robust to HGT, but it explicitly identifies and corrects for HGT at multiple stages of the analysis. By subtracting the background of short length, coincidental matches and restricting itself to a range of longer lengths (~7 or more amino acids), SlopeTree is able to follow the evolution of the highly conserved segments of proteins, using approximately 10,000 to 40,000 amino acids per genome pair. The highly conserved regions that SlopeTree targets correspond to the alignable regions in an MSA.

For 72 strains of *Escherichia coli* and *Shigella*, 73 archaea, and 495 bacteria, we built trees using different degrees of HGT-correction. We compared these trees to trees based on phylogenetically broad concatenated alignments from the literature [25], in which supermatrices were constructed from 24 single-copy, ubiquitous genes and then passed to a Maximum Likelihood (ML) routine for tree-building. These comparisons were performed to assess the accuracy of our method and to identify potential biological sources for differences. The SlopeTree strain-level trees were remarkably stable for different inputs. Even when only mobile elements together with proteins that are not part of the core were considered, the tree topology was highly similar. The archaeal trees were more fluid upon restricting the method to the most conserved proteins, but the majority of clades and relationships between deep branches remained the same. The deep, short branches in the bacterial trees were the most unstable, which is related to a generic problem of defining phylogenetic relationships in evolutionary radiation. For archaea and bacteria, we calculated the symmetric difference distance [61] to the trees built from supermatrices for trees built by SlopeTree, ACS, CVTree, D2, kmacs, Spaced Words and ALFRED-G. By applying our ME filter and conservation filter to the data prior to running the main SlopeTree routines, we were able to significantly reduce the distances to the trees built from supermatrices not only for SlopeTree but for all other alignment-free methods. We observed approximately 20 bacteria whose placement on the phylogenetic trees frequently disagreed between alignment-free methods and the current NCBI classification. The consistency of these alternative placements for these bacteria when applying alignment-free methods suggests that their classification may require revision, or at the very least have complex histories. This is further supported by the fact that several of these bacteria had similar disagreements between the trees built from supermatrices and the NCBI classification.

## Results

### Algorithms

Our method is based on k-mers that are substrings of length *k*. The SlopeTree package includes both the main SlopeTree algorithm, which estimates evolutionary distance by quantifying how quickly the number of matching sequences between two proteomes decays as a function of sequence length, and several independent modules for filtering mobile elements and less-conserved proteins out of the data and recalculating distances for pairs still exhibiting significant HGT even after the earlier filtering steps. Altogether, the method consists of the following four modules: (1) a Mobile Element Filter, (2) a Conservation and Stability Filter, (3) the SlopeTree Main Algorithm and (4) a Pair-Wise Horizontal Gene Transfer (HGT) Correction. A flowchart is provided in S1 Fig.

The Mobile Element Filter exploits a novel signature which is based on analysis of multiple copies of almost identical protein sequences in a genome. These highly repetitive proteins proved almost always to be mobile elements. The Conservation and Stability Filter calculates for each protein a value, which we call a *paralogy* score, from the ratio of the sum of how many genes each of the protein’s k-mers has a match with in other genomes to the sum of the total number of genomes the protein’s k-mers have matches with. This ratio effectively separated orthologous proteins evolving by descent, which typically have a gene to genome ratio of one and therefore had paralogy scores of approximately one. Mobile elements on the other hand, have paralogy scores frequently much greater than one because their presence, absence, and copy number are much more unstable, while unconserved proteins which simply have no k-mer matches with any other proteins in the input have scores of 0.

The SlopeTree Main Algorithm estimates a distance for every pair of organisms from the decay in the number of exact sequence matches as a function of match length.

The Pair-Wise HGT Correction assesses the slopes produced by the SlopeTree Main Algorithm and identifies pairs of organisms that appear to have shared significant horizontal transfers; it runs the SlopeTree Main Algorithm on these pairs combined with a reference set to identify proteins that the pair shares but that are absent from the reference, and then it re-runs the SlopeTree Main Algorithm on just the pair, with the flagged proteins removed.

The four modules are not necessarily run together; for instance, the SlopeTree Main Algorithm can be run on unfiltered data or data passed through only one of the filters.

### Algorithm 1: Mobile Element Filter

**Input**. A set S of *n* proteomes 〈*S*_1_, *S*_2_, …, *S*_*n*_〉 and a set T = 〈*T*_1_, *T*_2_, …, *T*_*l*_〉, with T taken from *l* taxonomically diverse organisms where *T*_*i*_ consists solely of the highly conserved proteins of the organism *i*. In practice, *l* is generally much smaller than *n*, but this is not required.

**Output**. A set V = 〈*V*_1_, *V*_2_, …, *V*_*n*_〉 where each *V*_*i*_ consists of all proteins in *S*_*i*_, minus the mobile elements.

**Algorithm**. Let *p*_*ij*_ be the *j*^*th*^ protein in *S*_*i*_, and let $p_{k}^{ij}\left[h\right]$ be a k-mer from *p*_*ij*_ of length *k*, starting at index *h*, where *0*≤*h*<*f* given that *p*_*ij*_ has length *f*. For those k-mers at the end of each protein where *h*+*k*>*f*, the suffix is expanded by the necessary number of empty characters to fill the remainder of the k-mer. Each k-mer is stored as a 2-tuple consisting of the k-mer and the gene ID (*j*). Let *A*_*i*_ be the alphabetically sorted list of all 2-tuples from *S*_*i*_. For every protein *p*_*ij*_, there is a pair of integers, *r*_*ij*_ and *c*_*ij*_, both initialized to 0. Starting from the first k-mer in *A*_*i*_, we pass down the list until a k-mer with more than *u* mismatches with this first k-mer is found. For all proteins with k-mers in this block, *r*_*ij*_ is incremented. This process is repeated until the end of *A*_*i*_ is reached, always starting from the first k-mer to not be a member of the current block of matches.

Separately, we repeat the k-mer compilation process described above on T to generate a single, alphabetically sorted list of 2-tuples across all proteomes in T. Duplicates are removed from this list to make a new list B consisting of each k-mer and the number of times it appears in T. Those k-mers appearing only once are given a count of 1. Then for every k-mer in *A*_*j*_, we query B; the value of *c*_*ij*_ is increased by the count stored in B for every exact match between B and any k-mer in any protein *p*_*ij*_.

Having set all *r*_*ij*_ and *c*_*ij*_ for all *p*_*ij*_ in *S*_*i*_, we define a linear function such that all *p*_*ij*_ with *r*_*ij*_≥*ac*_*ij*_*+b* are removed from proteome *P*_*i*_ and the reduced proteome we call *V*_*i*_.

**Computational complexity**. For *n* organisms and *m* amino acids in S, let m = m_1_+m_2_+…+m_n_. For *l* organisms and *k* amino acids in T, let k = k_1_+k_2_+…+k_l_. The compilation of *A*_*i*_ is done in *O*(*m*) time, and the time required for sorting each *A*_*i*_ is *O*(*m*_*i*_ log *m*_*i*_), which summed over all *n* organisms is *O*(*m* log *m*). Similarly, the time to compile all k-mers in T is *O*(*k*) and to sort them requires *O*(*k* log *k*) time. The order of the algorithm is dominated by the sorting, and therefore the computational complexity of the filter is *O*(*m* log *m + k* log *k*).

### Algorithm 2: Conservation and Stability Filter

**Input**.A set W of *n*+*k* proteomes consisting of two sets of proteomes: a set V of *n* proteomes 〈*V*_1_, *V*_2_, …, *V*_*n*_〉 and a set U of *z* proteomes 〈*U*_1_, *U*_2_, …, *U*_*z*_〉, with U taken from taxonomically diverse organisms.

**Output**. A set H = 〈*H*_1_, *H*_2_, …, *H*_*n*+*k*_〉 where *H*_*i*_ is the subset of *W*_*i*_ containing conserved proteins with stable copy number.

**Algorithm**. Let *p*_*ij*_ be the *j*^*th*^ protein in *W*_*i*_, and let $p_{k}^{ij}\left[h\right]$ be a k-mer from *p*_*ij*_ of length *k*, starting at index *h*, where *0*≤*h*<*f* given that *p*_*ij*_ has length *f*. For those k-mers at the end of each protein where *h*+*k*>*f*, the suffix is expanded by the necessary number of empty characters to fill the remainder of the k-mer. Each k-mer is stored as a 3-tuple consisting of the k-mer, the proteome ID (*i*), and the gene ID (*j*). Let D be the alphabetically sorted list of all 3-tuples from both V and U.

We define a k-mer cluster to be a block of adjacent k-mers in D in which no k-mer has more than *u* mismatches with the previous k-mer. Starting from the first k-mer in D, we compare adjacent k-mers to identify all clusters in D. At the end of this process, the k-mers in adjacent clusters are checked against one another and merged by the same rule of no more than *u* mismatches, a step which circumvents the frequent problem of stray k-mers interrupting what would otherwise be a single block of matches. We call this final set of clusters C.

Every protein in *p*_*ij*_ from W is assigned a pair of integer arrays, *G*_*ij*_ and *F*_*ij*_ each initialized at every index to 0 (default size = 10). For each cluster in *C*, let *g* be the number of organisms from U with *at least* one k-mer in the cluster, and let *f* be the number of total 3-tuples in the cluster with k-mers from U, including repeats. We use *G*_*ij*_ and *F*_*ij*_ to accumulate the sums of *f* and *g*, respectively, for each cluster; the index of the array for a given cluster is selected by a function of the fraction of the total proteomes in U with hits in the cluster. If *y* is the number of proteomes in U with hits in the cluster, *o* = ⌊10*y*/*z*⌋. For every protein *p*_*ij*_ with a k-mer in a given cluster from C, let *g* and *f* be added to the values of *G*_*ij*_ and *F*_*ij*_ at index *o*, respectively.

After passing through all clusters in C, we assign a paralogy score for every protein *p*_*ij*_, for each possible value of *o*, where we define a paralogy score $X_{ij}^{o}$ for each value of *o* as $X_{ij}^{o}=\;\sum_{k=o}^{k<10}G_{ij}\left[o\right]/\sum_{k=0}^{k<10}F_{ij}[o]$. *H* consists of all proteomes in *V* and *U*, where only proteins that have $0<X_{ij}^{o}\leq$ orthology cutoff (default = 1.3) retained. How conserved the final set H is depends on the user’s selection of *o*.

The reference set U is not mandatory. When a reference set is absent, the whole set V is treated as the reference by the algorithm.

**Computational complexity**. As in Algorithm 1, the time to compile the sorted list of k-mers is *O*(*m* log *m*), where *m* is the total number of amino acids in W. The clustering is performed in *O*(*m*) time, and the calculation of final scores is performed in *O*(*n*+*k*) time. Therefore, the computational complexity of the filter is *O*(*m* log *m)*.

### Algorithm 3: Main SlopeTree Algorithm

**Input**. A set H of *n* proteomes 〈*H*_1_, *H*_2_, …, *H*_*n*_〉.

**Output**. A distance matrix D of SlopeTree evolutionary distances between all pairs in H, such that *D*_*ij*_ is the SlopeTree distance between proteomes *H*_*i*_ and *H*_*j*_.

**Algorithm**. Let *p*_*ij*_ be the *j*^*th*^ protein in *H*_*i*_, and let $p_{k}^{ij}\left[h\right]$ be a k-mer from *p*_*ij*_ of length *k*, starting at index *h*, where *0*≤*h*<*f* given that *p*_*ij*_ has length *f*. For those k-mers at the end of each protein where *h*+*k*>*f*, the suffix is expanded by the necessary number of empty characters to fill the remainder of the k-mer. Each k-mer is stored as a 3-tuple consisting of the k-mer, the proteome ID (*i*), and the gene ID (*j*). Let L be the alphabetically sorted list of all 3-tuples.

Let $m_{r}^{xy}$ be an exact sequence match of length *r*, where *1≤r≤k* for proteomes *P*_*x*_ and *P*_*y*_, where each match is counted exactly once. Let $M_{r}^{xy}$ be count of all $m_{r}^{xy}$, where the same sequence is only counted once. For all *r* in the evolutionarily relevant range, ~*r*>8 amino acids, we define *D*_*xy*_ as an estimate of the evolutionary distance between proteomes *P*_*x*_ and *P*_*y*_, where *D*_*xy*_ is the decay in the histogram of $\text{ln}(M_{r}^{xy})$ as a function of *r*.

**Computational complexity**. For *n* organisms and *m* amino acids, let m = m_1_+m_2_+…+m_n_. The compilation of L is done in *O*(*m*), and the sort within all organisms is equal to Σ*O*(*m*_*i*_ log *m*_*i*_) which is equal to *O*(*m* log *m*). The match-counting algorithm then requires *O*(*m* log *m* + *n*^*2*^) time. Thus, the time complexity is *O*(*m* log *m + n*^*2*^), with *m*>>*n*. We treat the alphabet size as a constant here.

### Algorithm 4: Pair-Wise Horizontal Gene Transfer (HGT) Correction

**Input**. A previously calculated SlopeTree distance matrix D (defined in Algorithm 3), a list Q of proteome pairs flagged as requiring additional correction, and a set R of proteomes, with R taken from taxonomically diverse organisms.

**Output**. A new distance matrix D`identical to D except for the distances between all pairs in Q, which have been recalculated.

**Algorithm**. Let *p*_*ij*_ be the *j*^*th*^ protein in *R*_*i*_, and let $p_{k}^{ij}\left[h\right]$ be a k-mer from *p*_*ij*_ of length *k*, starting at index *h*, where *0*≤*h*<*f* given that *p*_*ij*_ has length *f*. For those k-mers at the end of each protein where *h*+*k*>*f*, the suffix is expanded by the necessary number of empty characters to fill the remainder of the k-mer. Each k-mer is stored as a 3-tuple consisting of the k-mer, the proteome ID, and the gene ID. Let *S* be the alphabetically sorted list of all 3-tuples from *R*.

Let *v* and *w* be a pair in Q. Then for this pair, we compile an alphabetically sorted list of 3-tuples and call this list P. Let S and P be merged and this list passed to Algorithm 3, i.e. the SlopeTree Main Algorithm for counting matches. During the match-counting, let any protein *p*_*ij*_ contributing a match between *v* and *w* with a nit-score (proportional to the length of the match, described in Implementation) higher than some cutoff *x*, and with fewer than *y* hits among the reference set, be marked. Having reached the end of the merged list of S and P, and having marked all proteins from *v* and *w*, we rerun Algorithm 3 on P, but ignoring matches from the marked proteins, to produce a new distance, *D`*_*vw*_.

Let the original distance *D*_*vw*_ be replaced by the new distance *D`*_*vw*_, and the matrix *D`*be the matrix in which every element has been updated in this way for all pairs in *Q*.

**Computational complexity**. Compiling the alphabetically sorted list *S* takes *O*(*r* log *r*) time, where *r* is the total number of amino acids in R. Similarly, compiling *P* takes *O(p* log *p)* time, where *p* is the total number of amino acids in *v* and *w*. Each first iteration of the SlopeTree main algorithm then requires *O*(*r* log *r + p* log *p*) time, and running the pair requires *O*(*p* log *p*) time. This must be repeated for every pair in Q. For a total of *n* organisms, i.e. a distance matrix to recalculate that is *n* by *n*, the worst case scenario is that every pair has been flagged, requiring that n^2^/2 distances be recalculated, but in practice, and especially after having applied the filters described in Algorithms 1 and 2, the number of pairs in Q is much smaller.

### Implementation

The algorithms behind the four main modules of the SlopeTree package (S1 Fig) were described in the Algorithms section. Here we present some important details regarding their implementation, including how the methods address uneven composition of amino acids, the possibility of backwards mutations, and the background of coincidental matches over short k-mer lengths. The source-code for SlopeTree is available at http://prodata.swmed.edu/download/pub/slopetree_v1/slopetree.tar.gz.

#### Assigning unique ordinals to proteomes and proteins

The first operation of SlopeTree is to detect all organisms in the input (a source directory containing FASTA files is provided by the user), alphabetically sort them by name, and assign them a unique integer, which we refer to as a genome ID, starting from 0.

#### Assembling the k-mer lists

SlopeTree generates a list of all k-mers (default = 20-mers) from all proteomes in the input set by means of a sliding window. Those k-mers shorter than 20 (i.e. k-mers from the end of each protein) are buffered a ‘^’, signifying ‘no character’, and k-mers containing non-standard amino acids (e.g. U) are ignored. In the same way that each proteome is given an ID (described above), each protein is given an integer ID which is unique within (but not between) proteomes. Each k-mer then is associated with a proteome ID and a protein ID as a 3-tuple, and these 3-tuples are sorted alphabetically into a final list. To facilitate various operations embedded in the SlopeTree code, and to facilitate development, SlopeTree uses its own procedures for k-mer counting and sorting.

At the k-mer generation stage, SlopeTree also compares k-mers to a small set of conserved, hardcoded sequences from EF-Tu. Proteins with k-mers that overlap with these sequences by 60% or more are considered matches and are marked so that the filters, if applied subsequently, do remove them. This is to prevent EF-Tu, which is a highly conserved protein, from being eliminate due to its unusual copy number.

#### Removing low complexity sequences

Those k-mers with significantly reduced amino acid alphabets (i.e. low complexity sequences) are not included in the sorted list. For each k-mer, SlopeTree counts the total number of times each amino acid is present (*c*_*n*_). The low-complexity score (S) of the k-mer is calculated as the sum of the squares for these counts.

$$\text{S}\;=\;\sum_{n=1}^{20}c_{n}^{2}$$ (1)

The k-mers with scores above a given cutoff (*C*) are discarded. Originally, this cutoff was manually set to 130 for 20-mers after manual inspection of k-mers, but to allow for different values of *k*, *C* is calculated by SlopeTree as 6.5*k*.

#### Match-counting

The list of k-mers merged across all proteomes is passed to the main SlopeTree algorithm, a match-counting routine that recursively partitions the sorted list of 3-tuples into blocks having the same leading amino acid, with three base cases for the recursion: the end of the k-mers has been reached, with the match reaching the last character in the block; the current block consists of only one k-mer, meaning that the current k-mer has no matches; and the end of the k-mer list has been reached. At the beginning of the match-counting process, a 3-dimensional integer array *A* with dimensions (number of organisms) by (number of organisms) by (maximum possible match score value) is initialized to 0. Then, for each unique k-mer match of score *s* between any pair of organisms *p* and *q*, array *A* has the entry at *A*[*p*][*q*][*s*] incremented.

#### Scoring matches

The simplest scoring scheme for a match would be to score by the match length. We extended this to nit-scores. Prior to counting matches, for each proteome, the number of instances of each amino acid (*c*_*a*_) and the total number of amino acids (*T*) are counted, and amino acid frequencies (*f*_*a*_) of each proteome are then calculated:

$$f_{a}=\frac{c_{a}}{T}$$ (2)

For each proteome, for each amino acid, a nit-score (*s*_*a*_) is then calculated:

$$s_{a}=\;-\text{ln}\left(f_{a}\right)$$ (3)

To take into account that each proteome has its own set of nit-scores, this can be rewritten for a specific proteome *p* as:

$$s_{p,a}=\;-\text{ln}\left(f_{p,a}\right)$$ (4)

For a match *m* of length *l* between two organisms, *p* and *q*, where *m*[*i*] is the amino acid at match position *i*, the score *m*_*pq*_ for the match, *m*, would be:

$$m_{pq}=\;\sum_{i=1}^{l}\frac{1}{2}\left(s_{p,m\left[i\right]}+s_{q,m\left[i\right]}\right)$$ (5)

There were two motivations for using nit-scores. One was to improve the rejection of coincidental matches. Coincidences of more frequent amino acids were more likely, so relying on a nit-score provided better rejection, with stretches of frequent amino acids having to be longer to contribute to the evolutionarily relevant range of the data. The second consideration was to obtain a more fine-grained sampling than number of amino acids, which for 20-mers would have defined just 20 bins. However, the slope expressed in nits also had a composition-dependent relationship to the slope expressed in mutations. Because our target was a slope expressed in units of mutation, there was a need for a correction factor that was composition dependent.

#### Background subtraction

**Fig 1:**
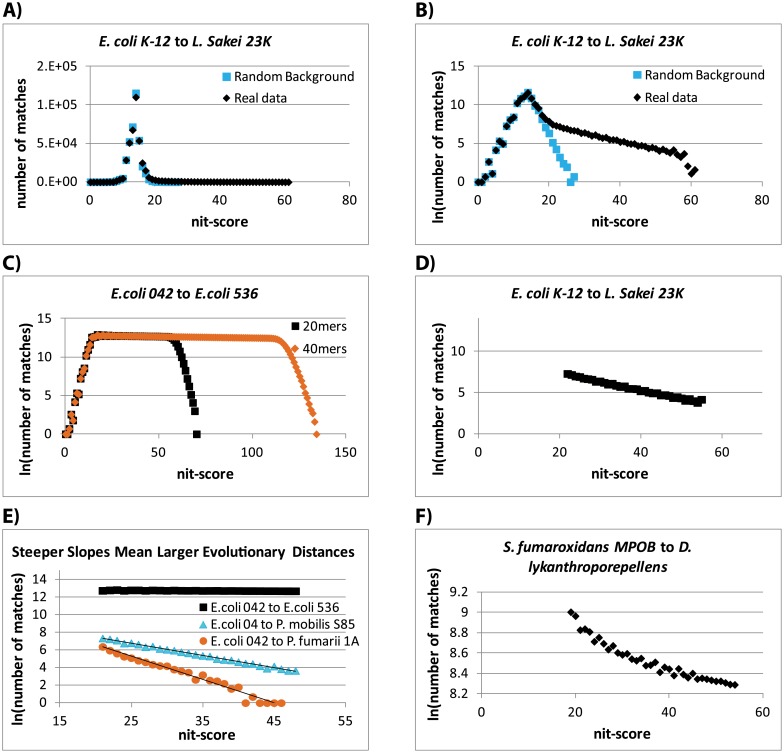
Main SlopeTree plots. (A) Number of matches between *Escherichia coli K-12* and *Lactobacillus sakei 23K* for range of nit-scores available to 20-mers (black), and same plot from randomized data (blue). (B) Natural log of number of matches between same bacteria as in (A) (black), and corresponding plot in natural log from randomized data (blue). (C) Natural log of number of matches between 2 bacteria of the same species, using 20-mers (black) and 40-mers (orange). (D) “Evolutionary signal” extracted from (B). (E) *E*.*coli 042* compared to 3 bacteria: *E*.*coli 536* (black); *Petrotoga mobilis S85* (blue); and *Pyrolobus fumarii 1A* (orange). (F) Natural log of number of matches between *Syntrophobacter fumaroxidans MPOB* and *Dehalogenimonas lykanthroporepellens*, a pair exhibiting HGT.

The match-counting produces a histogram of the number of unique k-mer matches shared by every pair, for a range of nit-scores (rounded to integers) from 0 to the maximum possible nit-score for the chosen k-mer length (*t*_*i*_). Because at the lower nit-score range, these histograms can also contain coincidental matches, SlopeTree subtracts a background from these histograms. During the initial k-mer compilation, a separate set of k-mers is generated in which the original proteins have had their amino acid order scrambled prior to applying the sliding window. This scrambling is performed on amino acid fragments of length 1 to 4. The sorted, merged k-mer list derived from the scrambled proteins is also passed through the SlopeTree match-counting algorithm (Algorithm 3), generating its own set of histograms in which the evolutionarily conserved sequences have been completely erased. SlopeTree’s background correction consists of subtracting the counts from the histograms obtained from randomized sequences from the histograms obtained from real data (Fig 1A and 1B).

#### Binning correction

**Fig 2:**
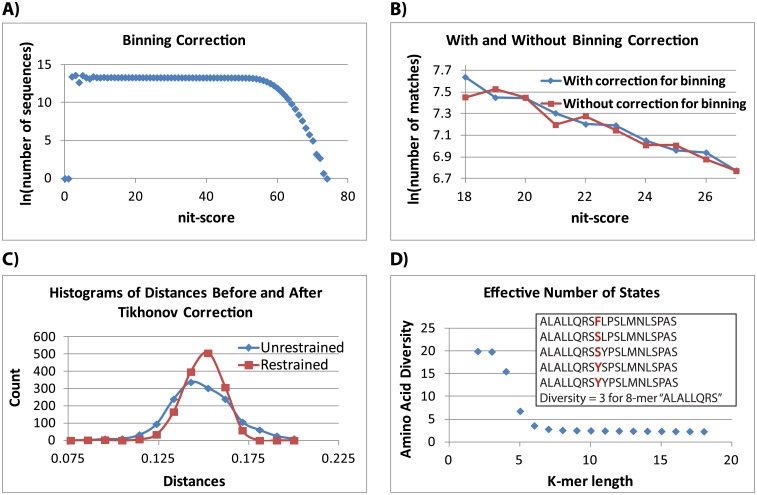
Refining SlopeTree evolutionary distances. A) Plot for binning correction. B) Corrected (blue) and uncorrected (red) data. C) Tikhonov positive restraint. D) Calculating an effective number of states to correct for nonlinearity in the SlopeTree distances.

SlopeTree also corrects for binning artifacts caused by amino acid frequencies and unusual patterns in amino acid composition. For every pair of organisms, an additional histogram is produced consisting of the nit-scores from every single sequence in either proteome, from length 1 to the k-mer length. Sequences are counted regardless of whether or not they have matches (Fig 2A). These sequences are scored using the nit-scores derived for the particular pair, just as in the main match-counting code (*b*_*i*_). To produce a histogram corrected for binning artifacts (*y*_*i*_), where *i* corresponds to rounded nit-scores, for each score in the natural log of the real data (*t*_*i*_), the natural log of these bin-correction counts (*b*_*i*_) is subtracted, and the average of the bin-correction (〈*B*〉) added back:

$$y_{i}=\;\text{ln}(t_{i})-\text{ln}\left(b_{i}\right)+\;\langle B\rangle ,$$ (6)

This correction was particularly important for improving the accuracy of the slope measurement because it mostly applied to the data in the lower nit-scores to which SlopeTree gives the highest weights (described below) (Fig 2B).

#### Bounds selection

SlopeTree uses the area of the histogram corresponding to the decay of evolutionarily conserved sequences. This requires that for each plot, the lower and upper bounds of this area be selected. For the nit-scores in which the counts for the scrambled data (see Background subtraction) are more than 25% the counts for the real data, the real data values are set to 0, and the left bound set to the nit-score with the maximum count. To select the right bound, the binning correction described above is used. This correction provides an estimate of the nit-score at which the cap on matching sequences, imposed by the maximum k-mer length, would cause the match counts to begin to decline (for nit-scores greater than ~55 in Fig 2A). For each binning correction plot, a rolling average 〈*R*〉 across the counts is calculated; starting at nit-score 0, ln(〈*R*〉) for each index is stored in a vector. This vector is then scanned for the largest nit-score at which the value of the natural log of the bin correction counts is within 0.1 of the natural log for the rolling average at that same index (*i*). The right bound is set to *i*-1, *assuming* the match counts are greater than 0 at this value. Otherwise, it is set to the lowest nit-score for which the pair had no matches.

#### Estimating evolutionary distances by measuring SlopeTree slopes

The histograms SlopeTree produces (Algorithm 3) consist of the number of unique k-mer matches between a pair of proteomes over the range of all possible nit-scores. These histograms, when plotted in natural log, exhibit a linear dependence at the higher nit-scores which corresponds to the decay of evolutionarily conserved sequences (Fig 1A and 1B). As the matches become sparser for higher nit-scores, the data becomes increasingly noisy. Therefore, the slope is measured using a weighted fit, where the scores with higher counts are given more weight (*w*(*i*)) than those with lower counts:

$$w_{i}=\frac{t_{i}}{t_{i}+W},$$ (7)

*w* is a constant set to 100 by default. Three fits are then calculated for the data: a linear fit, a quadratic fit, and a fit for the sum of two exponentials.

Linear fit:

y = ax+b (8)

d = −a (9)

Quadratic fit:

$$y=ax^{2}+\;bx+c$$ (10)

d = −(2ax+b) (11)

Double exponential fit:

$$y=Fe^{-lx}+Ge^{-mx}$$ (12)

d = max(l,m) (13)

The slope (*d*) is invariant in the linear equation. In the quadratic equation, the slope varies as a function of *x*, with the choice of *x* having an effect on the final trees. By default, *x* is set to 15.

#### Tikhonov positive restraint

SlopeTree applies a Tikhonov positive restraint to the *a*-coefficients. This requires two passes through the data: in the first pass, the average slope (〈*A*〉) over all plots, the root mean square deviation for the fit (*RMSD*), and the uncertainty of the slope (*σ*) are calculated. These values are then included in the summation terms used to calculate the restrained version of the fit. When calculating the fit for the quadratic equation, we first multiplied out the square of the quadratic equation, which we divided into sums, where $S_{40}\;=\;\sum x_{i}^{4}$ and $S_{21}\;=\;\sum x_{i}^{2}y_{i}$. These two terms were then modified by the Tikhonov restraint for the new fit (Fig 2C), such that U_40_ and U_21_ were used in the subsequent fit calculations:

$$\prime U_{40}=S_{40}+{\left(\frac{RMSD}{\sigma }\right)}^{2}$$ (14)

$$U_{21}=S_{21}+\;\langle A\rangle {\left(\frac{RMSD}{\sigma }\right)}^{2}$$ (15)

Slopes were always either negative or in the case of extremely similar organisms, approximately 0. We reversed the sign for all slopes, making distances positive with larger values corresponding to larger distances. SlopeTree uses all three fits at different stages of calculation, but the evolutionary distances used for the trees are derived from the quadratic fit, because the linear fit was too sensitive to HGT and the double exponential proved unstable.

#### Converting slopes to evolutionary distances

We performed two operations to convert our slopes into evolutionary distances. During the initial compilation of the sorted k-mer list, the entropy for each pair of organisms was calculated. For organisms *p* and *q*, this entropy (*H*_*pq*_) is calculated as:

$$H_{pq}=-\Sigma_{k=1}^{20}\frac{\left(\frac{c_{i}\left(k\right)}{T_{p}}+\frac{c_{j}\left(k\right)}{T_{q}}\right)}{2}\text{ln}\frac{\left(\frac{c_{i}\left(k\right)}{T_{p}}+\frac{c_{j}\left(k\right)}{T_{q}}\right)}{2}$$ (16)

The final slopes are the slopes derived from the quadratic fit multiplied by their respective entropy.

The other operation was necessary due to backwards mutations (i.e. revertants). Alignment-based methods have very complex mathematics for the accumulation of multiple mutations. However, alignment-free methods only have to consider multiple mutations when they revert to their original position. In the absence of backwards mutations, the slope would be the evolutionary distance for the highly conserved subset of a proteome. This simplified the evolutionary model, which essentially became a two-state model for each amino acid in the starting k-mer (either preserved or not). It was necessary to know, at least roughly, what number of amino acids a starting position could mutate to. In principle, this number would be 19, but in highly conserved positions that were still variable, selection restricted the effective number of possible states. If the total number of possible states was *n*, and *D* was the evolutionary distance, *d* the slope, and *x* the point at which the slope was taken for the quadratic, then our model was that:

D = −wln((w−dH)/w) (17)

w=1−1/n (18)

d=−(2ax+b) (19)

This formula was easy to invert to pass from slope to evolutionary distance, but there remained the problem of how to estimate the factor of *n*. We performed a somewhat simplified calculation in order to estimate this value by observing the number of alternative amino acids in k-mers longer than *n*. We found the possible range of *n* to be somewhere between 2.8 and 20 (Fig 2D). Our estimate was likely a lower bound for the actual number. Because of the finite length of the evolutionary distances, we did not observe all possible alternative states, so this presumably caused the estimate to be an underestimation of the actual *n*. This restrained the range of the nonlinearity correction in our model. We expect that the true number would be much closer to the bottom of the range than 20, and *n* = 2.8 is the default setting. But even taking the smallest value corresponding to the largest correction for nonlinearity, within the groups of free-living bacteria or free-living archaea, this nonlinearity correction is not large. As *n* becomes larger, the formula becomes more linear; a nonlinearity correction using *n* = 20 would be minimal.

This is an incomplete description, because the number of alternative amino acids will be different at every position, and this will make the nonlinearity correction somewhat different from simply averaging the number of possible states. However, seeing as how there is already some uncertainty in our nonlinearity correction, this is a secondary consideration. Furthermore, distance-based methods are robust in terms of the nonlinearity of their measure with respect to evolutionary distance. This robustness depends on the type of the phylogenetic inference from the distance method. CVTree is the best example of limited sensitivity to nonlinearity correction; it has a highly nonlinear distance measure, but nevertheless produces meaningful trees. Considering that we faced a minimal range of nonlinearity uncertainty, in terms of tree construction, this could not have been a major factor.

#### Mobile element (ME) filtering (Algorithm 1)

Alphabetically sorted k-mer lists for each proteome are generated at the very beginning of a SlopeTree run. For each organism separately, these k-mers are clustered by comparing immediately neighboring sequences in the list. By default, k-mers that are identical in 19 out of 20 amino acids are put into the same cluster. The values for *a* and *b*, mentioned in Algorithm 1, are by default 1.0 and 3.0, respectively. This filter makes it possible to identify the elements that are highly repetitive within a single genome, which are almost always parasitic elements such as phage proteins. These are removed from the analysis. EF-Tu is the one consistent exception to this. EF-Tu is frequently present in multiple copies in a single genome.

#### Conservation filtering (Algorithm 2)

The k-mers in the final alphabetically sorted list across all organisms are compared to their immediate neighbors and grouped together if *x* amino acids (default = 13 out of 20) are identical (i.e. same amino acid in the same position). The default value of 13 matches (for 20-mers) for clustering is adjustable, with a higher cutoff (e.g. 19 or 20) being suitable for strain-level phylogeny. At the end of the clustering and counting process, paralogy scores are calculated by dividing the protein count field by the genome count field. Orthologs generally have a value of 1 for this ratio, whereas paralogs and mobile elements have ratios that are often much higher. These values are summed for each protein across all clusters. A final value of 0 causes the protein to be marked for elimination. Proteins with a paralogy score greater than an orthology cutoff (default = 1.3) are also eliminated. The default value of 1.3 was chosen in consideration for EF-Tu.

Paralogy scores can be calculated for a range of conservation levels. A parameter, which we refer to as *o* in the text, refers to the level of filtering that was applied. The two variables mentioned above, genome count and protein count, are both arrays (default size = 10) in the implementation (arrays *G*_*ij*_ and *F*_*ij*_ in Algorithm 2). Genome count and protein count for index 0 (i.e. *o* = 0) of this table would be updated for every cluster regardless of cluster size. For index 2 (*o* = 2) of the table, on the other hand, the value would only be updated only for clusters in which 20% or more of the reference set was represented. Paralogy scores calculated from higher indices of the table therefore produced smaller proteomes consisting of more conserved proteins.

#### Pair-wise HGT correction (Algorithm 4)

First the pair-wise HGT correction identifies pairs with signs of HGT. Pairs in which the double exponential weighted RMSD (*x)* produces a better fit than the quadratic fit weighted RMSD (*y)* are flagged for the correction (default cutoff: *x/y* < 0.9). A shallow slope (i.e. indicating evolutionary closeness) but a high RMSD for the linear fit (default: RMSD>0.12; slope<0.06) also cause a pair to be flagged, because the RMSD is typically very low for slopes from truly close organisms.

For each flagged pair, two iterations through the SlopeTree match-counting code are performed. First, k-mers from a flagged pair are passed through the match-counting code alongside a diverse, pre-selected reference set. Two tables of integers are updated during this match-counting, where each element corresponds to a protein from either of the flagged organisms; one table logs all matches between a protein and the reference set, while the other logs all matches between the flagged pair. Only matches of a given length or longer (default = 12 or more amino acids) are counted for these two tables. At the end of the match-counting, these tables are compared; proteins shared by the pair that are not present in a certain number (default = 3) of reference set organisms are flagged. The pair, without the reference set, is then passed through the match-counting code once more, with all flagged proteins excluded.

#### Flagging potentially problematic inputs

SlopeTree identifies potential problems in the input such as: reduced genomes (<140,000 amino acids), under-representation of conserved genes, over-representation of conserved genes, and candidate status. Reduced genomes are detected at the early k-mer-counting step. Candidate division organisms are identified simply by scanning the name of the organism for ‘Candidatus.’ SlopeTree identifies proteomes with an under- or over- representation of conserved genes by means of calculations performed during the k-mer clustering described in Algorithm 2. To identify an over-representation of conserved genes, SlopeTree calculates the average number of hits for a cluster per reference proteome, for every cluster containing a large fraction (default = 0.9) of the reference set. Generally, such clusters come from conserved proteins, and this average number of hits is close to 1. For every cluster, for every organism represented in the cluster, the difference between the number of hits that the organism has in the cluster and the average number of hits per reference organism is stored as a running sum. Some organisms are left with much higher values for these sums than others; the IDs of these organisms are written to file. SlopeTree identifies proteomes with an under-representation of conserved genes in a similar manner, using the same set of clusters discussed above (i.e. 90% or more of the reference set present in the cluster). For every organism, SlopeTree counts the number of times the organism has a hit in one of these cluster. At the end of the process, some organisms which were frequently absent from these conserved clusters had significantly lower values for this count, and were also written to file.

These tests identified that genomic sequences based on WGS assembly of environmental reads can have particular characteristics, such as paralogy, rather different from complete genome assemblies. This is very likely due to the intrinsic difficulties in performing assembly based on a non-homogeneous source.

Series of SlopeTree (ST) trees were generated for 72 *Escherichia coli* and *Shigella*, 73 archaea, and 495 bacteria. SlopeTree provides two filters that remove proteins from the input prior to the distance calculations. The Mobile Element (ME) Filter (Algorithm 1) removes mobile elements by taking advantage of their unique copy number patterns within individual proteomes. The Conservation and Stability Filter (Algorithm 2) removes proteins exhibiting an unstable pattern of presence and absence in a taxonomically diverse reference set, with a parameter (*o*) corresponding to the fraction of reference organisms that have to have k-mer matches with a given protein the protein to be retained. SlopeTree also provides additional, separate correction for horizontal gene transfer (HGT) (Algorithm 4) which identifies specific pairs of organisms that appear to have transferred genes and re-calculates the distance using the main SlopeTree routine, with the suspicious proteins removed from the data. This correction is not expected to be effective for extremely ancient transfers, but is adequate for recent transfers such as those involving phage proteins.

We built ST-trees on “raw” (i.e. no filtering) proteomes, proteomes filtered of mobile elements, proteomes filtered of mobile elements and also non-conserved, unstable proteins, and finally filtered proteomes passed through the additional HGT-correction. Most of these trees were pruned of organisms flagged by SlopeTree as problematic, e.g. reduced organisms. For comparison purposes, we calculated symmetric difference (SD) [61] distances between all ST-trees and the supermatrix trees [25], which we call Eisen-495 (bacteria) and Eisen-73 (archaea), and Eisen-445 and Eisen-71 for their pruned counterparts (S2 Fig). We also calculated the distances to the Eisen-trees for trees built using other alignment-free methods, namely Average Common Substring (ACS), CVTree, D2, kmacs, and Spaced Words and ALFRED-G. These alternative methods were given both raw data and also a variety of filtered inputs.

SlopeTree proved to be an effective tool for strain-level phylogeny, despite the number of matches between strains of the same species being enormous and most distances being very close to zero (Fig 1C and 1E). SlopeTree was applied to archaea and bacteria separately because matches for organisms belonging to different domains can be very sparse, branch-length nonlinearity is magnified at very large genetic distances (e.g. between the domains of life), and there are cases of occasional but extensive HGT between domains [62–64].

### Filtering for Mobile Elements and by Stability and Conservation

We observed occasional curvature in the SlopeTree histograms (Fig 1F). The linear fit was inadequate for plots exhibiting this curvature. Manual inspection of the proteins associated with long length matches between organisms with unexpectedly close distances identified several cases of horizontal gene transfer (HGT). We implemented a quadratic fit to address this, which produced better slopes for a number of cases. However, the quadratic fit also performed poorly when it came to large-scale HGT, e.g. cases involving single copy phages. For this reason, we developed the two filters and the final HGT correction (Algorithms 1–2, 3).

Mobile elements are often present in multiple copies in a single genome, with their k-mers therefore also being present in multiple copies; we used this feature of mobile element k-mer copy number to identify and remove these proteins. This criteria removed an average of 118 proteins from each archaea (stdev = 116) and 162 proteins from each bacteria (stdev = 246). The archaea with the most mobile elements removed was *Methanosarcina acetivorans C2A*, which had 744 proteins removed out of a total 4540. The bacteria with the most mobile elements removed, and which did not show issues with data quality, was *Arthrospira platensis NIES-39*, which had 2143 proteins removed out of a total 6630. The effect this filtering had on the distance to the Eisen-trees was variable; SlopeTree and CVTree show negligible difference before and after the application of the filter; ACS and kmacs showed a small reduction in distance to the Eisen-trees; and D2 and Spaced Words showed a significant reduction in distance to the Eisen-trees.

The conservation filter used a taxonomically diverse reference set of organisms to identify proteins with k-mers that had hits for a minimum fraction (~*o*) of the reference set, and calculated paralogy scores that provided an estimate of a protein’s copy number profile across the entire reference set. This filter was applied to the majority of the ST-trees, in conjunction with the ME-filter. The purpose was to observe how the phylogenetic trees might change as the input was reduced to an increasingly conserved core, and to assess whether these automatic filters could help produce higher quality trees while keeping the methods completely unsupervised. As a validation, we generated histograms from the paralogy scores for proteins with specific keywords in their annotations, with for example ‘ribosomal’ as an instance of a core protein and ‘chemotaxis’ as an instance of an unstable, often horizontally transferred protein (S3 Fig). The former has a sharp peak at the paralogy score of 1 which decreased but does not disappear for increasing *o*. The latter has two peaks at 0 and 5, with all paralogy scores of 1 disappearing by *o = 2*, indicating that chemotaxis proteins are frequently absent or present in multiple copies. Proteins with paralogy scores less than 1 and greater than 1.3 are filtered out; therefore, as *o* is raised, chemotaxis and other similar proteins are gradually eliminated while the majority of ribosomal proteins and other stable, conserved proteins are retained. For every method, this filtering steadily reduced the distance to the Eisen-trees (Table 1) and organisms that were misplaced (according to the NCBI taxonomy) in the unfiltered trees were frequently placed correctly in the more filtered trees.

**Table 1:** Distance to Eisen trees for SlopeTree and for five other whole-genome methods, over different levels of mobile-element and conservation filtering.

Distance to Eisen-495 tree and Eisen-445 tree	Symmetric difference	Distance to Eisen-73 tree and Eisen-71 tree	Symmetric difference
**ST—raw**	**506**	**ST—raw**	**56**
**ST—pruned**	**426**	**ST—pruned**	**52**
**ST—pruned, ME**	**432**	**ST—pruned, ME**	**54**
**ST—pruned, ME, o = 3**	**388**	**ST—pruned, ME, o = 3**	**42**
**ST—pruned, ME, o = 3, HGT**	**384**	**ST—o = 3, HGT**	**50**
**ST—pruned, ME, o = 5**	**388**	**ST—ME, o = 5**	**38**
**ST—pruned, ME, o = 5, HGT**	**390**	**ST—ME, o = 5, HGT**	**38**
**ST—pruned, ME, o = 7**	**404**	**ST—ME, o = 7**	**42**
**ST—pruned, ME, o = 7, HGT**	**404**	**ST—ME, o = 7, HGT**	**42**
**ACS—raw**	**554**	**ACS—raw**	**58**
**ACS—pruned**	**480**	**ACS—pruned**	**56**
**ACS—pruned, ME**	**474**	**ACS—pruned, ME**	**50**
**ACS—pruned, ME, o = 3**	**412**	**ACS—pruned, ME, o = 3**	**36**
**ACS—pruned, ME, o = 5**	**420**	**ACS—pruned, ME, o = 5**	**34**
**ACS—pruned, ME, o = 7**	**410**	**ACS—pruned, ME, o = 7**	**34**
**CVTree—raw**	**676**	**CVTree—raw**	**64**
**CVTree—pruned**	**868**	**CVTree—pruned**	**60**
**CVTree—pruned, ME**	**868**	**CVTree—pruned, ME**	**62**
**CVTree—pruned, ME, o = 3**	**838**	**CVTree—pruned, ME, o = 3**	**34**
**CVTree—pruned, ME, o = 5**	**832**	**CVTree—pruned, ME, o = 5**	**34**
**CVTree—pruned, ME, o = 7**	**830**	**CVTree—pruned, ME, o = 7**	**34**
**D2—raw**	**528**	**D2—raw**	**50**
**D2—pruned**	**458**	**D2—pruned**	**44**
**D2—pruned, ME**	**416**	**D2—pruned, ME**	**36**
**D2—pruned, ME, o = 3**	**386**	**D2—pruned, ME, o = 3**	**32**
**D2—pruned, ME, o = 5**	**366**	**D2—pruned, ME, o = 5**	**34**
**D2—pruned, ME, o = 7**	**366**	**D2—pruned, ME, o = 7**	**32**
**kmacs—raw**	**524**	**kmacs—raw**	**50**
**kmacs—pruned**	**448**	**kmacs—pruned**	**48**
**kmacs—pruned, ME**	**440**	**kmacs—pruned, ME**	**48**
**kmacs—pruned, ME, o = 3**	**390**	**kmacs—pruned, ME, o = 3**	**36**
**kmacs—pruned, ME, o = 5**	**372**	**kmacs—pruned, ME, o = 5**	**34**
**kmacs—pruned, ME, o = 7**	**350**	**kmacs—pruned, ME, o = 7**	**32**
**spaced—raw**	**810**	**spaced—raw**	**88**
**spaced—pruned**	**720**	**spaced—pruned**	**80**
**spaced—pruned, ME**	**684**	**spaced—pruned, ME**	**76**
**spaced—pruned, ME, o = 3**	**478**	**spaced—pruned, ME, o = 3**	**64**
**spaced—pruned, ME, o = 5**	**452**	**spaced—pruned, ME, o = 5**	**56**
**spaced—pruned, ME, o = 7**	**430**	**spaced—pruned, ME, o = 7**	**46**

To be valid inputs to SlopeTree, proteomes cannot be filtered beyond a certain level. This is because SlopeTree distances are derived from the decay of k-mers as a function of match length, and when the average proteome size drops below ~100–200 proteins, the algorithm begins to encounter pairs that no longer have measurable or informative slopes. This defines a filtering limit for SlopeTree in the vicinity of *o* = 8 or *o* = 9, but not all alignment-free methods have this constraint.

The pair-wise HGT correction was designed to correct very occasional but serious error when a single copy phage was transferred between distal organisms. The mobile element filter is not designed to identify single copy phages, which represent a rare category of phages. The ME filter, conservation filter, and pair-wise HGT correction are separate modules in SlopeTree that are applied at different times and address slightly different issues in the data. However, they overlap in many of the proteins they remove; for instance, the mobile element filter and conservation filter both remove many proteins that the HGT filter would remove, were the conservation filter not applied. In general, we found that by *o = 3* or *o* = 5, most problematic proteins were already removed and the HGT filter had little impact on the final trees.

### SlopeTree Applied to Strains: 72 *Escherichia coli* and *Shigella*

**Fig 3:**
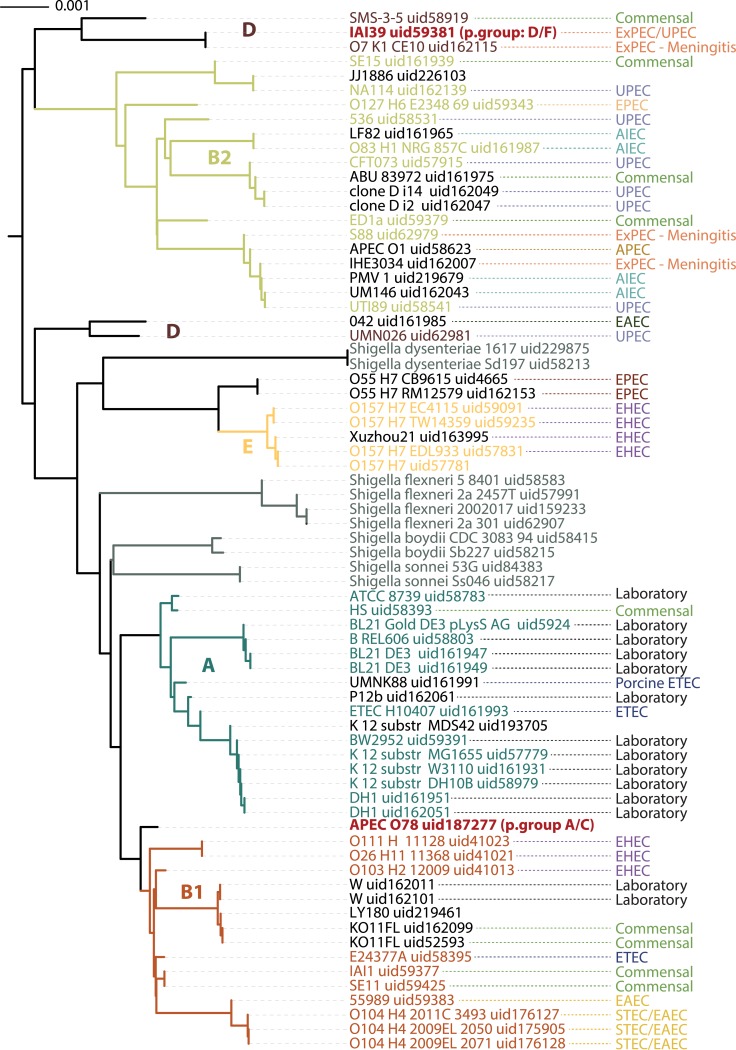
ST-tree from 72 *E*. *coli* and *Shigella* using 20-mers and unfiltered data.

A series of ST-trees was built for 62 *E*.*coli* and 10 *Shigella* (Fig 3 and S4 Fig), which were all the complete proteomes available for these species at the time of this writing. This was to test the range at which SlopeTree could still resolve sensible evolutionary distances. *Escherichia fergusonii* and *Escherichia blattae* were included in the run as outgroups to root the trees, but were removed from the final distance matrices prior to tree-building because their presence excessively compressed the other distances. To assess whether longer k-mers might produce more accurate distances at the strain-level, we built a tree using 20-mers (Fig 3) and another using 40-mers. We did not observe an improvement; the 20-mer and 40-mer trees were in very close agreement, with topological differences arising from short branches mainly in the B2 phylogroup. We built additional trees using proteomes filtered for mobile elements, and also proteomes filtered for stability and conservation, in which the reference set for the conservation filter was simply the entire input. The average number of proteins per proteome for the 72 *E*.*coli* and *Shigella*, prior to filtering, was 4730 (stdev = 485). When the set was filtered just for mobile elements, the average size was reduced to an average of 4282 proteins (stdev = 402). This set, with mobile elements removed, was filtered against itself for the smallest possible filtering parameter (*o* = 0), reduced the average proteome size to 4071 (stdev = 362); for self-filtering on *o* = 5, the average size was then 3465 (stdev = 209); and for *o* = 10, the average size was 1290 (stdev = 9). For all trees, the trees were highly similar to the unfiltered trees. We performed more aggressive conservation filtering against a reference set of 30 diverse bacteria (*o* = 3), leaving an average of 343 (stdev = 41) proteins per proteome. This was done to investigate whether the trees built from the most conserved genes across the entire domain of bacteria matched those built without filtering and those built with loose filtering. Again, we observed only minor changes in topology, mostly involving short branches. As an additional validation, we reduced the unfiltered 20-mer tree to the set considered in Touchon et al. [65] which was used as a reference for another alignment-free method in Sims et al. [66]; these two topologies were also found to be in agreement.

The ST strain-level topology also agreed with current phylogroups of *E*.*coli* and *Shigella*. There are different means for determining phylogroups, with some assignments varying between approaches [67, 68]; SlopeTree supports the grouping of *E*. *coli IAI39 uid59381*with phylogroup D and *E*. *coli APEC O78 uid187277* with phylogroup C. Pathotypes do not follow phylogeny [69] and when they were mapped the trees, their placement was scattered. The genes responsible for pathogenicity are frequently mobile elements [56, 70, 71], so we constructed an ST-tree from mobile elements and less conserved proteins removed during filtering on *o* = 0, to investigate whether strains of the same pathotype would cluster. We did not see this effect; not surprisingly, this tree differed from the other trees in several placements, but nevertheless held many groupings in common, particularly between the more closely related strains (S4 Fig).

When strains differ by very few mutations in DNA, most of these will not cause changes in coding sequence. For such cases, performing phylogenetic analyses by following the easily identifiable mutations at the DNA level is the more accurate and practical approach.

### SlopeTree Applied to 73 Archaea

**Fig 4:**
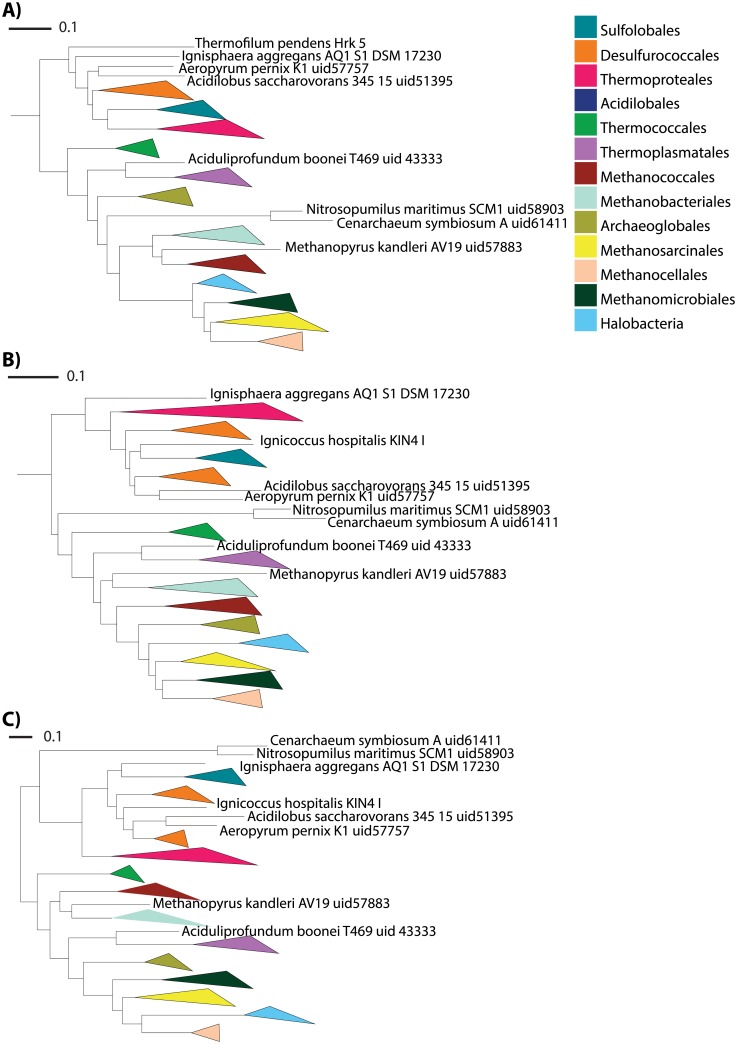
Phylogenetic trees for 73 Archaea. A) ST-tree, raw and pruned. B) ST-tree, pruned, with mobile element filtering and conservation filtering (*o* = 5). C) Eisen-71 tree.

A series of ST-trees was constructed for 73 archaea (Fig 4 and S5 Fig). These 73 were all the archaea in Lang et al. [25] that had available proteomes in NCBI. Two archaea were pruned from the distance matrix prior to building the trees: *Candidatus Korarchaeum cryptofilum OPF8 uid58601*, and *Nanoarchaeum equitans Kin4 M uid58009*. Both were automatically flagged by SlopeTree for having an unusually low number of conserved genes compared to the rest of the set. As with the strain-level analysis, we generated both unfiltered ST-trees and also filtered ST-trees, and also applied our pair-wise HGT correction. These trees were compared to the Eisen-73 and Eisen-71 trees. Differences in filtering parameters produced some changes in topology, with distances to the Eisen-73 tree generally decreasing as filtering increased. For instance, without filtering (but with pruning), the symmetric difference distance was 52, compared to 38 for filtering on *o* = 5. For the purpose of comparison, we also built trees on unfiltered and filtered data using five other alignment-free methods: ACS (S6 Fig), CVTree (S7 Fig), D2 (S8 Fig), kmacs (S9 Fig), and Spaced Words (S10 Fig). A smaller set of trees, due to the long run-time of the program, was calculated for ALFRED-G (S11 Fig). The symmetric difference distances to the Eisen-73 and Eisen-71 trees are shown in Table 1, with more distances available in S1 Table.

### SlopeTree Applied to 495 Bacteria

**Fig 5:**
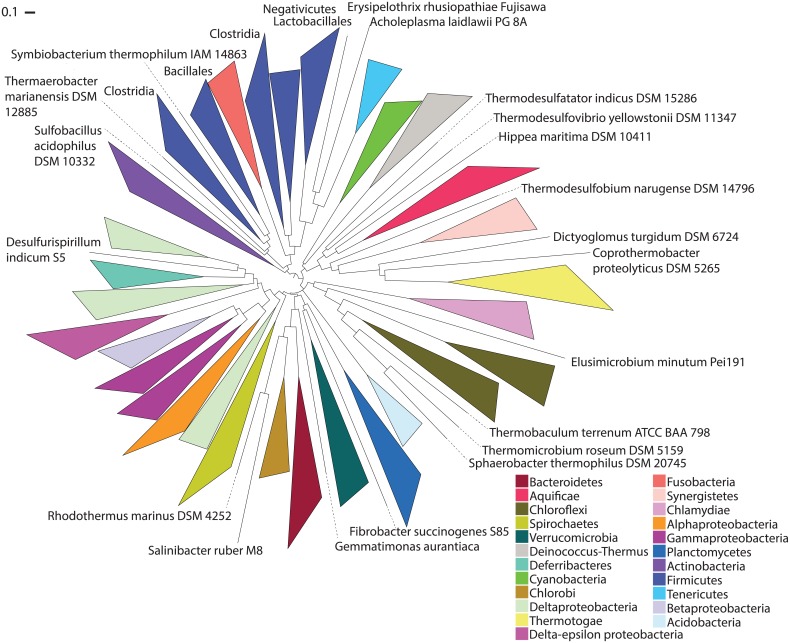
ST-tree of 495 bacteria. Tree was pruned to 445, with mobile element filtering and conservation filtering (*o* = 3). Pair-wise HGT correction was performed.

We built a series of ST-trees for 495 bacteria on unfiltered data, filtered data (varying the value of *o*), and with and without the final pair-wise HGT correction (Fig 5 and S12 Fig). As the root, we chose the division between the gram-negative and gram-positive bacteria. Organisms identified by SlopeTree as problematic (e.g. unusual number of conserved genes, reduced genomes, significantly fragmented assemblies, candidate division, etc.) were retained throughout the entire SlopeTree run, but pruned from the majority of the final trees (S1 Text). Mobile element and conservation filtering reduced the distance to the Eisen-495 tree for all methods, fixing several misplacements of individual organisms as well as shifting whole branches to locations more in keeping with the current NCBI classifications. By ‘misplacement’ we mean a disagreement with the current NCBI classification. For the purpose of comparison, we built trees on full and filtered data using ACS (S13 Fig), CVTree (S14 Fig), D2 (S15 Fig), kmacs (S16 Fig), and Spaced Words (S17 Fig). We also built trees using ALFRED-G, but could only test the *o* = 5 and *o* = 7 inputs due to the long run-time of the program (S18 Fig). The ALFRED-G distances are included in S1 Table.

There is no consensus regarding the positions of the deep branches of phylogenetic trees. Even the attempt to root the tree on the division between gram-positive and gram-negative bacteria could not be done cleanly, with the Chlamydiae, Cyanobacteria and Spirochaetes moving between these two groups for different levels of filtering. Not just SlopeTree, but all alignment-free methods have changes in their tree topologies as the inputs are filtered more aggressively. Nevertheless, we observed some stable features in the ST-trees that are stable for the other methods as well. These include a clade consisting of the Gammaproteobacteria, Betaproteobacteria, and Alphapoteobacteria. The Bacteroidetes, Chlorobi, and Gemmatimonadetes form another stable clade, typically neighboring a group consisting of the Spirochaetes and some subset of the Planctomycetes-Verrucomicrobia-Chlamydia (PVC) superphylum [72, 73]. These features are consistent with the Eisen-495 tree. The Deltaproteobacteria however are almost always polyphyletic or paraphyletic. The position of the Acidobacteria is also variable, grouping with the Proteobacteria (mainly the Deltaproteobacteria) or the PVC group. The Epsilonproteobacteria are consistently monophyletic, but they group with the Proteobacteria for raw and less-filtered trees (up to *o* = 3) and the Aquificae or PVC group for more filtered trees (*o* = 5 or more).

SlopeTree usually places the Aquificae and a diverse, sulfur-reducing thermophilic group with the gram-negative bacteria, close to a group of Deltaproteobacteria. Filtering and the pair-wise HGT correction move this clade to an area that is separate from the majority of the gram-negative bacteria (Proteobacteria, Bacteroidetes, Chlorobi, Verrucomicrobia, Planctomycetes, etc.) and the gram-positive bacteria (Actinobacteria, Firmicutes) alike. The Cyanobacteria are also often found in this area; they are typically on a short, deep branch and in the filtered trees, they neighbor the Deinococcus-Thermus. In the unfiltered ST-tree in which the pair-wise HGT correction was not performed, the Cyanobacteria are grouped with the Proteobacteria, which agrees with the Eisen-495 tree. However, a cursory investigation of the prospective HGT pairs for the members of Cyanobacteria present in the analysis revealed numerous possible transfers with the Proteobacteria, and the pair-wise HGT correction alone, even with no filtering, moved the Cyanobacteria away from the gram-negative bacteria and into the neutral area. This area also often includes a clade consisting of the Thermotogae and Synergistetes, another stable group whose placement in the trees varies between this area and a placement deep within the gram-positive bacteria.

The remainder of the tree consists predominantly of gram-positive bacteria. The Firmicutes and Actinobacteria typically share a common root, in agreement with the Eisen-495 tree. The Firmicutes are polyphyletic in all ST-trees, with the Tenericutes branching from within them. Whether the Tenericutes are their own phylum or belong within the Firmicutes is debated [74]; SlopeTree consistently groups them within the Firmicutes, matching the Eisen-495 tree. The occasional presence of the Thermotogae within the Firmicutes is at least in part due to a clear instance of HGT discussed later, but it has been observed that the Thermotogae and Firmicutes, in particular Clostridia, show similarity at the whole-genome level [75, 76]. The Fusobacteria are also in this clade, first nested within the Firmicutes but then more and more basal as filtering increases. The placement of the Fusobacteria with the gram-positive bacteria, despite their being gram negative, has support [76, 77]. This generally gram-positive clade also often included the Chloroflexi. Like the Thermotogae, the Chloroflexi mostly stain Gram negative, but are monoderms [78]. This placement is seen in the majority of trees produced by the other alignment-free methods and is also seen in the Eisen-495 tree.

### Bacteria That Diverge from the Eisen-495 Tree or the NCBI Classification

It is to be expected that different phylogenetic methods will produce different phylogenetic trees. However, the set of organisms that is misplaced in the trees according to the current NCBI taxonomy is remarkably consistent between all alignment-free methods and many of these misplacements were present in the supermatrix tree and specifically discussed in Lang et al. [25]. We discuss some of them below.

#### *Coprothermobacter proteolyticus*, Dictyoglomi, Thermotogae and Synergistetes

*C*. *proteolyticus*, currently classified as a member of Clostridia, is a thermophilic, gram-negative bacterium which was classified first as *Thermobacteroides proteolyticus* before being reclassified as a Firmicute, order Thermoanaerobacterales [79]. Through the entire range of ST-trees without exception, it maintains a stable position alongside *Dictyoglomus turgidum DSM 6724*, a member of the Dictyoglomi. Together, *C*. *proteolyticus* and *D*. *turgidum* neighbor the Thermotogae, and this group in turn neighbors the Synergistetes. This placement is supported by the Eisen-495 tree and by other, independent observations from the literature [80, 81]. Trees built by CVTree, D2, ACS, kmacs, Spaced Words, and ALFRED-G also support this classification.

#### A sulfur-reducing thermophilic cluster

There was tendency for sulfur-reducing thermophiles to cluster together in the tree, irrespective of their phylum. This cluster generally consisted of the Aquificae, a group of Deltaproteobacteria, and four additional bacteria: *Thermodesulfobium narugense* (Clostridia), *Thermodesulfatator indicus DSM 15286* (Thermodesulfobacteria), *Thermodesulfovibrio yellowstonii DSM 11347* (Nitrospira), and *Hippea maritima* (Deltaproteobacteria). All four were specifically described in Lang et al. [25] for their unusual phylogeny. *H*. *maritima* was placed in the Desulfurellaceae family of the Deltaproteobacteria by means of 16S rRNA [82]; Lang et al. propose [25] to move it to the Epsilonproteobacteria. In the ST-phylogeny, *H*. *maritima* consistently appears closest to the Aquificae, forming a clade with this phylum in every ST-tree except for the most stringently filtered (o = 7) ST-tree, in which it finally joins a clade consisting of Nitrospirae, Fibrobacteres, Verrucomicrobia, Planctomycetes, and the Epsilonproteobacteria. For *T*. *yellowstonii*, until filtering at *o* = 5, it groups with the Aquificae, but then moves to the Epsilonproteobacteria. On the other hand, *T*. *narugense*, for *o* = 3 groups with *C*. *proteolyticus*, *D*. *turgidum*, the Thermotogae and the Synergistetes. This is the placement supported in the Eisen-495 tree. However, for *o = 5* and *o* = 7, it groups with the Deinoccocus-Thermus. *T*. *desulfatator*, for the totally raw tree, the pruned tree, and filtered for *o* = 0, o = 1, and o = 5, it is found among the sulfur-reducing group; for *o* = 3 and *o* = 7, and also in the tree where no conservation filtering (only mobile element filtering) has been performed, it groups with the Deltaproteobacteria.

These four, together with the Aquificae, indicate that the less filtered trees, which provide a phylogenetic perspective that is inaccessible to alignment-based approaches, sometimes reflect phenetics over phylogeny. This grouping was present in all alignment-free methods, persisting to different extents as the data were filtered.

#### *Acidithiobacillus ferrooxidans ATCC 23270* and *Acidithiobacillus caldus*

NCBI currently classifies these two acidophiles as Gammaproteobacteria. In the unfiltered ST-tree, they form a basal group within the Gammaproteobacteria as well. However, their placement is unstable, and filtering can move them to within the Betaproteobacteria or make them a basal group for the two phyla. Compounding this ambiguity is the fact that under the heaviest conservation, they return to the Gammaproteobacteria. This ill-defined behavior was apparent in the other alignment-free phylogenies as well. It has been noted before that the *Acidithiobacillales* behave ambivalently [83, 84]. Lang et al. [25] propose the creation of an “eta-proteobacteria” lineage for them. The alignment-free trees do not contradict this proposal.

#### *Dehalogenimonas lykanthroporepellens* and *Dehalococcoides mccartyi 195*

*D*. *lykanthroporepellens* and *D*. *mccartyi* are members of the Chloroflexi. Both stain Gram negative, with the former being a mesophile—a somewhat unusual feature for a Chloroflexi. Both were classified by means of the 16S rRNA gene [85, 86]. When no filtering was performed, SlopeTree misclassified this pair, grouping *D*. *lykanthroporepellens* with the Gammaproteobacteria and *D*. *mccartyi* with the Firmicutes. This pair was also misclassified by all other alternative methods (ACS, CVTree, D2 and Spaced Words) up to some level of filtering, although D2 showed the most robustness to this misplacement. The misplacement of *D*. *lykanthroporepellens* is due to a phage transfer shared with *Syntrophobacter fumaroxidans*, and *Desulfarculus baarsi* (Gammaproteobacteria). The pair-wise HGT correction also flagged the Firmicute *Natranaerobius thermophilus JW/NM-WN-LF* as being a possible partner of *D*. *mccartyi*, and removed several transporters prior to recalculating the evolutionary distance. The lightest level of conservation filtering (*o* = 0) was sufficient to fix the misplacement of these two Chloroflexi. We also found that the pair-wise HGT correction, even without filtering, also corrected their placement.

#### *Rhodothermus marinus* and *Salinibacter ruber*

Every ST-tree contains the Bacteroidetes and Chlorobi clade. However, the family Rhodothermaceae, which consists of *R*. *marinus* and *S*. *ruber* and is classified as belonging to the Bacteroidetes, is frequently either grouped with the Chlorobi or placed on a branch basal to both phyla. The Eisen-495 tree places this pair of bacteria with the Bacteroidetes, but all alignment-free methods frequently set this pair apart from the Bacteroidetes. When no ME filtering or conservation filtering were performed, or for very low levels of conservation filtering, ACS, CVTree and kmacs can completely misplace these two bacteria. For instance when no mobile element and conservation filtering are performed, kmacs groups the pair with the three Actinobacteria discussed above, the Myxococcales, and Deinococcus-Thermus.

### Pair-Wise HGT Correction and Examples of HGT

We observed two main classes of HGT for the pair-wise HGT correction. The first was associated with single copy phages. *D*. *lykanthroporepellens* and both *Syntrophobacter fumaroxidans* and *Desulfarculus baarsi* serve as an example of this. The second was related to adaptation-associated proteins. *Petrotoga mobilis* and *Mahella australiensis*, which shared a transfer of proteins associated with resistance to a toxic environment, are an example. Both were addressed by means of a combination of mobile element filtering and a sufficiently high value for *o*, and in general this was our preferred approach because it is significantly more efficient than running the HGT filter on a large number of pairs. However, we did observe that for a very low *o*, or when filtering was not applied, the pair-wise HGT correction was able to correct the placement of *D*. *lykothroporepellens*, *D*. *mccartyi*, and *P*. *mobilis* (S19 Fig). In addition, it amended the placement of *Leptospira biflexa serovar Patoc* and *Leptospira interrogans serovar Lai*, two Spirochaetes which every alignment-free method misplaced unless using a very high level of conservation filtering. *Rhodothermus marinus* and *Salinibacter ruber M8*, classified as Bacteroidetes, were also moved from the Chlorobi back to the Bacteroidetes. The correction also caused some substantial reordering of the deeper branches. The Gammaproteobacteria, which are completely monophyletic in the uncorrected tree, are split into two groups in the HGT-corrected tree, in both cases forming a monophyletic clade with the Betaproteobacteria; this split is often seen in the other alignment-free methods and may be an indication of a missing “eta” class for the Proteobacteria [25, 84]. The pair-wise HGT correction also removed the Cyanobacteria from the Proteobacteria, placing them close to the root alongside the Deinococcus-Thermus which were also shifted out of the Firmicutes. The Spirochaetes and Chlamydiae were also moved from the gram-positive bacteria to the gram-negative bacteria.

### Distances to Eisen-trees and Other Whole-Proteome or Alignment-Free Methods

The symmetric difference distance [61] was calculated between all alignment-free trees and the Eisen-trees, using the treedist program in PHYLIP [87]. However, we note that the Eisen-trees are only approximations of the real evolutionary history, and that the methods should not be judged as “better” or “worse” purely according to their distances to these approximations. The kmacs method, with mobile element filtering and conservation filtering on *o = 7*, achieved the closest tree to the Eisen-tree for both bacteria and archaea, with a symmetric difference distance of 350 and 32. D2 also achieved a distance of 32 to the Eisen-71 tree. For bacteria and archaea, SlopeTree achieved 384 and 38, both at *o* = 5.

**Fig 6:**
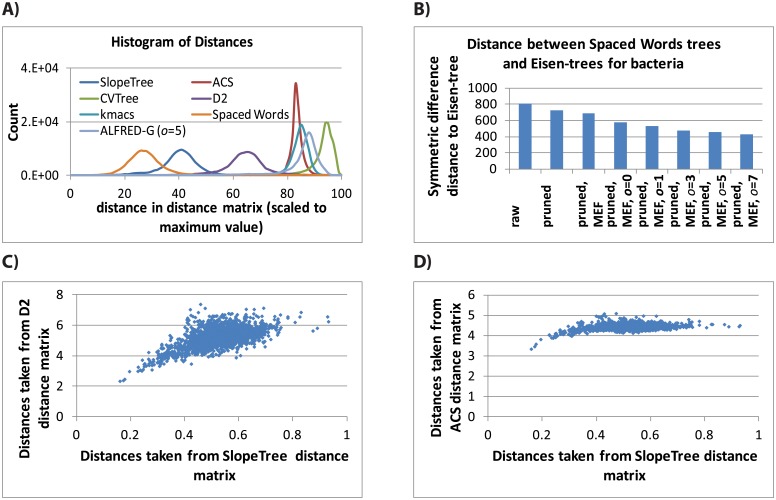
SlopeTree and other alignment-free methods. A) Histogram of scaled distances produced by each method. B) Decrease in symmetric difference distance to the Eisen-495 tree for Spaced Words method. C) SlopeTree distances to D2 distances for a matching set of randomly selected organism pairs. D) SlopeTree distances to ACS distances for a matching set of randomly selected organism pairs.

Filtering lessened the distance to the Eisen-trees for all methods (Fig 6B and Table 1). We observed a distinct difference in the nature of the branch lengths between different methods; D2, SlopeTree and Spaced Words fall into one group, having a wider range of branch lengths, while ACS, CVTree, kmacs, and ALFRED-G have branch lengths that are restricted to a more narrow range (Fig 6A, 6C and 6D). ACS appears to be the most restricted in this regard, and we found that by applying the conservation filter, the range for a given method’s distances was somewhat widened.

## Discussion

We tested SlopeTree, a new, alignment-free method for phylogenetic reconstruction, on a set of strains and also on two domains of life. The method implements three types of gene-filtering: filtering for parasitic elements using copy number within a genome; filtering of genes by their overall conservation; and filtering of gene pairs indicating HGT. The method also includes a bulk correction for genome-specific HGT, it corrects for nonlinearity of the distance measure, and it corrects for compositional bias affecting the background. Some of these corrections work cleanly, for example the mobile element (ME) filter which removes parasitic elements. Others represent only minor corrections to the distance estimate. The biggest influence came from the filtering of gene pairs and filtering for overall conservation, which corrected for various artifacts and helped in the analysis of the global patterns of co-evolution. For sets of core genes and also for complete genomes, SlopeTree produced trees that were close but not identical to those produced by traditional MSA approaches [25]. Our results point to the general validity of species evolution by descent, but with various types of exceptions.

Non-sexual, clonal evolution with horizontal transfer creates a problem for defining the rules of species evolution. These rules would inform us on how to interpret genomic data, given the assumption of evolution by descent. The traditional approach to this problem is to define the genes that always evolve together [25, 88, 89]. Such analyses are generally limited to the number of genes that are trustworthy, and these sets of genes in practice frequently correspond to ribosomal genes and proteins that interact with the ribosome [25, 30]. However, if possible, we would like to have a concept of species evolution in prokaryotes that is not dominated by the evolution of the ribosome.

Alignment-free approaches using complete genomes are an alternative to MSA approaches. We expect alignment-free methods, which look for consensus phylogenetic signals at the level of individual k-mers rather than gene-long alignments, to provide alternative insights into evolutionary history. For instance, alignment-free methods identified a cluster of sulfur-reducing thermophiles which was absent from the traditional MSA tree. To assess alignment-free methods, their trees can be compared to the ribosomal evolution tree, which is what we did here, but it may not always be clear to what extent disagreements are due to the method or to the lack of co-evolution.

### SlopeTree Distance Measure Is Closely Related to Accumulation of Mutations

Different measures of evolution, for instance different alignment-free methods, will produce different trees. Generally, these measures are correlated, generating highly concordant trees. Each alignment-free method defines similarity between organisms in its own units, but it still needs to be established how each of these measures can be transformed into units of accumulation-of-mutations and with what level of accuracy. SlopeTree was designed to provide a measure with a close relationship to the accumulation of mutations. In the absence of selection, this relationship would be given by a simple formula, but at larger evolutionary distances, the slope is defined by slowly evolving protein segments subject to strong negative selection. At the domain level, the relationship becomes nonlinear and requires calibration between the slope and the number of accumulated mutations. At very large distances, such as those between domains, the slope loses its relationship to evolutionary distance entirely. However, this is only significant for rooting archaeal and bacterial phylogenies.

The uniformity of the branch lengths from the “root” to the tips in the SlopeTree trees is not an artifact of the distance measure being nonlinear or saturating at some value. It may be a consequence of looking at a large number of conserved sites and if a particular locus evolved faster for a particular genome pair, its contribution becomes much smaller. Heterotachy, which is variable between positions in an alignment, has very different consequences in terms of branch length estimation for alignment-based methods and current alignment-free methods. Considering that there is much larger variability in branch lengths by alignment-based methods, it appears that more uniform branch lengths are a consequence of two factors: averaging between more proteins and potentially smaller sensitivity to heterotachy which is variable between positions in an alignment.

### SlopeTree Filtering Benefits Other Methods

SlopeTree includes a filter for mobile elements and a conservation filter which is applied to all proteomes prior to the main run. A conservation filter follows, which is adjustable. As the level of filtering increased, the distances between the ST-trees and the Eisen-73 or the Eisen-495 trees decreased. All other alignment-free methods that we tested also benefited from filtering the data prior to running, at least in terms of their distances becoming closer to the Eisen trees. An additional benefit to this is that filtering the data beforehand decreases the run-times.

The number of matches contributing to the assessment of evolutionary distances can be limited for longer distances or small genomes. Including mismatches adds a substantial number of informative, i.e. non-random, matches to the analysis. As can be seen with kmacs, the inclusion of mismatches can greatly improve phylogenetic distances. SlopeTree is essentially a type of survival analysis; therefore, it can apply to partial matches just as well as to those that are exact, and it is our expectation that such extension will produce even better results.

## Materials and Methods

### Downloading Proteomes, Selecting Input Sets, and Building Eisen-Trees

The archive all.faa.tar.gz was downloaded from the NCBI ftp website (ftp://ftp.ncbi.nih.gov/genomes/Bacteria/) in May 2015. The archive taxdump.tar.gz was downloaded from the NCBI taxonomy website (ftp://ftp.ncbi.nlm.nih.gov/pub/taxonomy/) also in May 2015. In the NCBI taxonomy, the root nodes for bacteria and archaea are 2 and 2157, respectively, and out of the 2774 organisms in the FASTA archive, 165 were identified as archaea and 2607 as bacteria. The Maximum Likelihood trees, S1 and S4 files from Lang et al. [25], built from the concatenations of 24 conserved proteins, were downloaded and organisms compared to those present in the FASTA archive. Allowing for some imperfect matches (e.g. *Haliangium ochraceum SMP 2 DSM 14365* in the ML tree, opposed to *Haliangium ochraceum DSM 14365* in the archive) and some differences in strains (e.g. *Eubacterium siraeum DSM 15702 uid54603* in the ML tree, opposed to *Eubacterium siraeum uid197160* in the archive), 73 archaea and 495 bacteria were found in common between the ML trees and the archive. Two lists were compiled of organisms to remove from the ML trees and these lists and trees were given as input to the program nw_prune, from the package newick-utils (version 1.6) [90]:

./nw_prune Eisen_newick_ML_journal.pone.0062510.s008.txt $(cat pruning_bacteria.txt) > eisen_495_tree_bacteria_newick.txt

./nw_prune Eisen_ML_841_journal.pone.0062510.s011.txt $(cat pruning_archaea.txt) > eisen_73__tree_archaea_newick.txt

These two supermatrix-derived trees are referred to as the Eisen-73 tree and the Eisen-495 tree and were produced for comparison purposes (S2 Fig).

### Neighbor Joining

For all distance matrices produced by SlopeTree and the other methods discussed here, we used rapidNJ version 2.0.1 [91] to construct the trees.

./rapidnj distance_matrix.txt > distance_matrix_tree.txt

### Pruning Trees

A raw tree consisting of the full sets bacteria and archaea is available for each method (SlopeTree and alternative alignment-free methods). The remaining trees were pruned of the organisms that SlopeTree automatically flagged as problematic, 2 for archaea and 50 for bacteria (S1 Text). The distance matrices were pruned of the flagged organisms before being passed to rapidNJ. Pruned versions of the Eisen-trees were also created (S2 Fig), using nw_prune as described above with the organisms flagged by SlopeTree added to the file of organisms to prune. This was necessary for the pruned trees to be comparable to the Eisen-trees.

### Building SlopeTree Trees

The scripts we refer to in this section are included in the SlopeTree package (http://prodata.swmed.edu/download/pub/slopetree_v1/slopetree.tar.gz).

The figures for all of the trees in this manuscript were generated using the ITOL web-server [92, 93].

### Commands for Constructing the Raw SlopeTree Trees for the Sets of Bacteria, Archaea and *E*.*coli*

All bacterial proteomes were moved to the directory FAA within the directory Bacteria. All archaeal proteomes were moved to the directory FAA within the directory Archaea. All proteomes for the strain-level analysis were moved to the directory FAA within the directory Ecoli. The distance matrices for these two sets were then generated with the following two scripts:

bash dSTm.sh Bacteria/ 20 B../Taxonomy/

bash dSTm.sh Archaea/ 20 A../Taxonomy/

bash dSTm.sh Ecoli/ 20 B../Taxonomy/

The distance matrices were then passed to rapidNJ. We refer to these trees as the “raw” trees.

### Selecting the Reference Sets for Bacteria and Archaea

We manually selected thirty diverse bacteria from the raw ST-tree as our reference set for the bacterial runs. Similarly, we manually selected ten diverse archaea for the archaeal runs. The specific organisms selected are listed in S2 Text.

### Building ST-Trees with Mobile Elements Removed

The reference sets for bacteria and archaea were moved to Bacteria_ref/FAA and Archaea_ref/FAA, respectively. We then filtered them for conservation, using our conservation filter, for the parameter of *o* = 7:

For bacteria:

bash pFilt.sh Bacteria_ref/ 20

./fpwrite Bacteria_ref/–f 10 –o 7

For archaea:

bash pFilt.sh Archaea_ref/ 20

./fpwrite Archaea_ref/–f 10 –o 7

These commands generated proteomes that had been reduced to their core proteins. These reduced proteomes were moved to new directories Bacteria_ref_10_7/FAA and Archaea_ref_10_7/FAA and the list of merged and sorted 20-mers generated for each of them:

bash dMT.sh Bacteria_ref_10_7/ 20 B

bash dMT.sh Archaea_ref_10_7/ 20 A

This created a set of sorted 20-mers from conserved proteins from a diverse reference set for bacteria and for archaea. These sets were used as the reference for the mobile element filtering:

./mef Bacteria/ Bacteria_ref_10_7/MERGED_TAGS/

./mef Archaea/ Archaea_ref_10_7/MERGED_TAGS/

./mef Ecoli/ Bacteria_ref_10_7/MERGED_TAGS/

This produced, for bacteria, archaea and our set of *E*.*coli*, a set of proteomes in which the mobile elements were eliminated. These reduced proteomes were automatically written out to Bacteria/FAA_mobelim, Archaea/FAA_mobelim and Ecoli/FAA_mobelim. We moved these reduced proteomes to Bacteria_MEF/FAA, Archaea_MEF/FAA and Ecoli_MEF/FAA and moved the organisms that had been chosen for the reference sets to FAA_ref directories within each main directory. We then ran the main SlopeTree script to produce the final distance matrices:

bash dSTm.sh Bacteria_MEF/ 20 B../Taxonomy/

bash dSTm.sh Archaea_MEF/ 20 A../Taxonomy/

bash dSTm.sh Ecoli_MEF/ 20 B../Taxonomy/

Trees were then built using rapidnj.

### Building Trees Filtered by Conservation

The FAA and FAA_ref directories from Bacteria_MEF/ and Archaea_MEF/, and the FAA directory for Ecoli_MEF, were copied to Bacteria_MEF_CF, Archaea_MEF_CF, and ECOLI_MEF_CF, respectively. We then ran the filtering code:

bash pFilt.sh Bacteria_MEF_CF/ 20 B

bash pFilt.sh Archaea_MEF_CF/ 20 A

bash pFilt.sh Ecoli_MEF_CF/ 20 B

For bacteria and archaea separately, we generated five sets of proteomes filtered on *o* = 0, *o* = 1, *o* = 3, *o* = 5 and *o* = 7. The following two commands use o = 3 as an example:

./fpwrite Bacteria_MEF_CF/–f 10 –o 3

./fpwrite Archaea_MEF_CF/–f 10 –o 3

This command generated filtered proteomes, still divided into main set and reference set, for both bacteria and archaea. These filtered proteomes were moved to their own directories, Bacteria_MEF_CF_10_3 and Archaea_MEF_CF_10_3 for the case of *o* = 3 and so on for other values of *o*. Finally, each of these new directories, which contained an FAA and FAA_ref that had been reduced for both mobile elements and also less conserved proteins, was passed to the main SlopeTree script:

bash dSTm.sh Bacteria_MEF_CF_10_3/ 20 B../Taxonomy/

bash dSTm.sh Archaea_MEF_CF_10_3/ 20 A../Taxonomy/

Similar steps were followed to generate the filtered proteomes for our set of *E.coli*, using the same set of 30 bacteria in FAA_ref for the more aggressive filtering. In addition, *E.coli* was filtered against itself, i.e. no reference set. All that was required for this self-filtering was to not provide an FAA_ref directory when pFilt.sh was run.

Trees were then built with rapidNJ.

### Trees Filtered of Mobile Elements, Conservation, and Horizontal Gene Transfer

The correction for HGT was applied only to proteomes already filtered of mobile elements and filtered on *o* = 3, *o* = 5, and *o* = 7. For *o* = 3, the command was:

./fh Bacteria_MEF_CF_10_3/

For each data set, this command produced new distance matrix which was then passed to rapidNJ.

### Building Alternative Trees

Trees were built using several other, alignment-free methods: ACS, CVTree, D2, kmacs, Spaced Words, and ALFRED-G. Each method was run on the 495 bacteria and 73 archaea for: a) raw proteomes, b) proteomes filtered of mobile elements, and c) proteomes filtered of mobile elements and also filtered for conservation on *o* = 0, 1, 3, 5, and 7. The final pair-wise HGT-correction which was applied to the SlopeTree runs for *o* = 3, 5, and 7 was not applied to these alternative methods because unlike the mobile element filter and conservation filter, the pair-wise HGT correction currently cannot be run independently of SlopeTree. For the matrices produced by these alternative methods, we built trees using rapidNJ.

### Average Common Substring

Version 1.2 of the ACS code was used to build the ACS trees with the following command:

./ACS -a <path to ACS directory>/ACS_input_file—o distance_matrix.txt—A -A ACS_matrix.txt

Trees were built using rapidnj on the file written out by the -o option.

### Composition Vector Tree (CVTree)

Version 4.2 of CVTree was used. The commands to build the matrices were the following:

./cvtree—i -i cvtree_input_file.txt -d FAA/ -k 6 -t aa -c out/

./batch_dist.pl 1.5 cvtree_input_file.txt out/ out_matrix_k6.txt

### D2 Method

Version 1.0 of D2 was used. The command to build the matrices was the following:

java -Xmx126g -jar jD2Stat_1.0.jar -a aa -i input.faa -o matrix

### kmacs

We ran kmacs with k = 14:

./kmacs input.faa 14

### Spaced Words

We ran Spaced Words with k = 12 and Euclidean distances. Evolutionary distances were not available for amino acid sequences:

./spaced—k -k 12 –d EU input_file.faa

### ALFRED-G

We ran ALFRED-G with k = 6 and x = 1.

build/alfred.x -f input.fas -o output.txt -k 6 -x 1

### Comparing Trees

All trees were compared to the Eisen-trees using the treedist tool from PHYLIP [87] for the symmetric difference distance. Using a keys file generated for the purpose of finding matches between the original FASTA archive and the Eisen-trees, we renamed the nodes of the Eisen-trees and alignment-free trees so that they were identical and renamed the two tree files intree and intree2 for treedist.

## Supporting Information

S1 Fig Phylogeny reconstruction flowchart for SlopeTree. SlopeTree has 3 main parts: The mobile-element filtering (Algorithm 1) and the conservation/stability filtering (Algorithm 2); the SlopeTree main method (Algorithm 3) which produces a distance matrix and tree; and the pair-wise HGT correction (Algorithm 4) which reprocesses pairs that were flagged as showing signs of HGT. When not using mobile-element filtering or conservation filtering, Start #2 is the original starting point. Three pairs are shown for the pair-wise HGT correction code; this number can be in the 100s or 1000s depending on the input set. All proteomes are in FASTA format.
(PDF)

S2 Fig Eisen-trees used as a reference. A) Eisen-73 tree. B) Eisen-71 tree (pruned Eisen-73). C) Eisen-495 tree. D) Eisen-445 tree (pruned Eisen-495).
(PDF)

S3 Fig Paralogy score histograms over different values of conservation/stability filtering parameter *o*. A) Histogram for all proteins with ‘ribosomal’ in their annotation, i.e. an example of paralogy scores for a highly conserved protein. B) Histogram for all proteins with ‘chemotaxis’ in their annotation, i.e. an example of paralogy scores for a non-conserved, frequently transferred protein.
(PDF)

S4 Fig SlopeTree (ST) applied at the strain level. Full set used consisted of 72 *Escherichia coli*, 10 *Shigella* (4 *S*. *flexneri*, 2 *S*. *boydii*, and 2 *S*. *sonnei*), *Escherichia fergusonii*, and *Escherichia blattae*. All trees used 20-mers unless otherwise specified. Due to the closeness of some organisms, some distances in the final Newick trees were negative; these were changed to 0 to avoid ‘backwards’ branches. This had no effect on the topology. A) ST on 72 *E*. *coli*, 10 *Shigella*, *E*. *fergusonii* and E. *blattae*. B) ST on 72 *E*. *coli* and 10 *Shigella*. C) ST using 40-mers on 72 *E*.*coli* and *10 Shigella*. D) ST on 72 *E*. *coli* and 10 *Shigella*, filtered for mobile elements. E) ST on 72 *E*. *coli* and 10 *Shigella*, filtered for mobile elements and self-filtered on *o* = 0. F) ST on 72 *E*. *coli* and 10 *Shigella*, filtered for mobile elements and self-filtered on *o* = 5. G) ST on 72 *E*. *coli* and 10 *Shigella*, filtered for mobile elements and self-filtered on *o* = 10. H) ST on 72 *E*. *coli* and 10 *Shigella*, filtered for mobile elements and filtered against a reference set of 30 diverse bacteria on *o* = 3. I) ST on 72 *E*. *coli* and 10 *Shigella*, built from mobile elements and proteins discarded when self-filtering on *o* = 0.
(PDF)

S5 Fig SlopeTree (ST) trees for archaea. A) Raw ST-tree for 73 archaea. Unfiltered and unpruned. B) Raw ST-tree for 71 archaea. Unfiltered and pruned. C) ST- tree for 71 archaea. Filtered of mobile elements and pruned. D) ST-tree for 71 archaea. Filtered of mobile elements, pruned, and filtered by stability and conservation on *o* = 0. E) ST-tree for 71 archaea. Filtered of mobile elements, pruned, and filtered by stability and conservation on *o* = 1. F) ST-tree for 71 archaea. Filtered of mobile elements, pruned, and filtered by stability and conservation on *o* = 3. G) ST-tree for 71 archaea. Filtered of mobile elements, pruned, filtered by stability and conservation on *o* = 3, and final pair-wise HGT correction applied. H) ST-tree for 71 archaea. Filtered of mobile elements, pruned, and filtered by stability and conservation on *o* = 5. I) ST-tree for 71 archaea. Filtered of mobile elements, pruned, filtered by stability and conservation on *o* = 5, and final pair-wise HGT correction applied. J) ST-tree for 71 archaea. Filtered of mobile elements, pruned, and filtered by stability and conservation on *o* = 7. K) ST-tree for 71 archaea. Filtered of mobile elements, pruned, filtered by stability and conservation on *o* = 7, and final pair-wise HGT correction applied.
(PDF)

S6 Fig Average Common Substring (ACS) trees for archaea. A) Raw ACS-tree for 73 archaea. Unfiltered and unpruned. B) Raw ACS-tree for 71 archaea. Unfiltered and pruned. C) ACS- tree for 71 archaea. Filtered of mobile elements and pruned. D) ACS-tree for 71 archaea. Filtered of mobile elements, pruned, and filtered by stability and conservation on *o* = 0. E) ACS-tree for 71 archaea. Filtered of mobile elements, pruned, and filtered by stability and conservation on *o* = 1. F) ACS-tree for 71 archaea. Filtered of mobile elements, pruned, and filtered by stability and conservation on *o* = 3. G) ACS-tree for 71 archaea. Filtered of mobile elements, pruned, and filtered by stability and conservation on *o* = 5. H) ACS-tree for 71 archaea. Filtered of mobile elements, pruned, and filtered by stability and conservation on *o* = 7.
(PDF)

S7 Fig CVTree trees for archaea. A) Raw CVTree on raw 73 archaea. Unfiltered and unpruned. B) Raw CVTree on 71 archaea. Unfiltered and pruned. C) CVTree on 71 archaea. Filtered of mobile elements and pruned. D) CVTree on 71 archaea. Filtered of mobile elements, pruned, and filtered by stability and conservation on *o* = 0. E) CVTree on 71 archaea. Filtered of mobile elements, pruned, and filtered by stability and conservation on *o* = 1. F) CVTree on 71 archaea. Filtered of mobile elements, pruned, and filtered by stability and conservation on *o* = 3. G) CVTree on 71 archaea. Filtered of mobile elements, pruned, and filtered by stability and conservation on *o* = 5. H) CVTree on 71 archaea. Filtered of mobile elements, pruned, and filtered by stability and conservation on *o* = 7.
(PDF)

S8 Fig D2 trees for archaea. A) D2 on raw 73 archaea. Unfiltered and unpruned. B) D2 on raw 71 archaea. Unfiltered and pruned. C) D2 on 71 archaea. Filtered of mobile elements and pruned. D) D2 on 71 archaea. Filtered of mobile elements, pruned, and filtered by stability and conservation on *o* = 0. E) D2 on 71 archaea. Filtered of mobile elements, pruned, and filtered by stability and conservation on *o* = 1. F) D2 on 71 archaea. Filtered of mobile elements, pruned, and filtered by stability and conservation on *o* = 3. G) D2 on 71 archaea. Filtered of mobile elements, pruned, and filtered by stability and conservation on *o* = 5. H) D2 on 71 archaea. Filtered of mobile elements, pruned, and filtered by stability and conservation on *o* = 7.
(PDF)

S9 Fig kmacs trees for archaea. A) kmacs on raw 73 archaea. Unfiltered and unpruned. B) kmacs on raw 71 archaea. Unfiltered and pruned. C) kmacs on 71 archaea. Filtered of mobile elements and pruned. D) kmacs on 71 archaea. Filtered of mobile elements, pruned, and filtered by stability and conservation on *o* = 0. E) kmacs on 71 archaea. Filtered of mobile elements, pruned, and filtered by stability and conservation on *o* = 1. F) kmacs on 71 archaea. Filtered of mobile elements, pruned, and filtered by stability and conservation on *o* = 3. G) kmacs on 71 archaea. Filtered of mobile elements, pruned, and filtered by stability and conservation on *o* = 5. H) kmacs on 71 archaea. Filtered of mobile elements, pruned, and filtered by stability and conservation on *o* = 7.
(PDF)

S10 Fig Spaced Words (SW) trees for archaea. A) SW tree for raw 73 archaea. Unfiltered and unpruned. B) SW tree for raw 71 archaea. Unfiltered and pruned. C) SW tree for 71 archaea. Filtered of mobile elements and pruned. D) SW tree for 71 archaea. Filtered of mobile elements, pruned, and filtered by stability and conservation on *o* = 0. E) SW tree for on 71 archaea. Filtered of mobile elements, pruned, and filtered by stability and conservation on *o* = 1. F) SW tree for 71 archaea. Filtered of mobile elements, pruned, and filtered by stability and conservation on *o* = 3. G) SW tree for 71 archaea. Filtered of mobile elements, pruned, and filtered by stability and conservation on *o* = 5. H) SW tree for 71 archaea. Filtered of mobile elements, pruned, and filtered by stability and conservation on *o* = 7.
(PDF)

S11 Fig ALFRED-G (ALF) trees for archaea. A) ALF tree for raw 73 archaea. Unfiltered and unpruned. B) ALF tree for raw 71 archaea. Unfiltered and pruned. C) ALF tree for 71 archaea. Filtered of mobile elements and pruned. D) ALF tree for 71 archaea. Filtered of mobile elements, pruned, and filtered by stability and conservation on *o* = 0. E) ALF tree for 71 archaea. Filtered of mobile elements, pruned, and filtered by stability and conservation on *o* = 7.
(PDF)

S12 Fig SlopeTree (ST) trees for bacteria. A) Raw ST-tree for 495 bacteria. Unfiltered and unpruned. B) Raw ST-tree for 445 bacteria. Unfiltered and pruned. C) ST- tree for 445 bacteria. Filtered of mobile elements and pruned. D) ST-tree for 445 bacteria. Filtered of mobile elements, pruned, and filtered by stability and conservation on *o* = 0. E) ST-tree for 445 bacteria. Filtered of mobile elements, pruned, and filtered by stability and conservation on *o* = 1. F) ST-tree for 445 bacteria. Filtered of mobile elements, pruned, and filtered by stability and conservation on *o* = 3. G) ST-tree for 445 bacteria. Filtered of mobile elements, pruned, filtered by stability and conservation on *o* = 3, and final pair-wise HGT correction applied. H) ST-tree for 445 bacteria. Filtered of mobile elements, pruned, and filtered by stability and conservation on *o* = 5. I) ST-tree for 445 bacteria. Filtered of mobile elements, pruned, filtered by stability and conservation on *o* = 5, and final pair-wise HGT correction applied. J) ST-tree for 445 bacteria. Filtered of mobile elements, pruned, and filtered by stability and conservation on *o* = 7. K) ST-tree for 445 bacteria. Filtered of mobile elements, pruned, filtered by stability and conservation on *o* = 7, and final pair-wise HGT correction applied.
(PDF)

S13 Fig Average Common Substring (ACS) trees for bacteria. Trees rooted using ‘Root mid-point’ option in ITOL server. A) Raw ACS-tree for 495 bacteria. Unfiltered and unpruned. B) Raw ACS-tree for 445 bacteria. Unfiltered and pruned. C) ACS- tree for 445 bacteria. Filtered of mobile elements and pruned. D) ACS-tree for 445 bacteria. Filtered of mobile elements, pruned, and filtered by stability and conservation on *o* = 0. E) ACS-tree for 445 bacteria. Filtered of mobile elements, pruned, and filtered by stability and conservation on *o* = 1. F) ACS-tree for 445 bacteria. Filtered of mobile elements, pruned, and filtered by stability and conservation on *o* = 3. G) ACS-tree for 445 bacteria. Filtered of mobile elements, pruned, and filtered by stability and conservation on *o* = 5. H) ACS-tree for 445 bacteria. Filtered of mobile elements, pruned, and filtered by stability and conservation on *o* = 7.
(PDF)

S14 Fig CVTree applied to bacteria. Trees rooted using ‘Root mid-point’ option in ITOL server. A) CVTree on raw 495 bacteria. Unfiltered and unpruned. B) CVTree on raw 445 bacteria. Unfiltered and pruned. C) CVTree on 445 bacteria. Filtered of mobile elements and pruned. D) CVTree on 445 bacteria. Filtered of mobile elements, pruned, and filtered by stability and conservation on *o* = 0. E) CVTree on 445 bacteria. Filtered of mobile elements, pruned, and filtered by stability and conservation on *o* = 1. F) CVTree on 445 bacteria. Filtered of mobile elements, pruned, and filtered by stability and conservation on *o* = 3. G) CVTree on 445 bacteria. Filtered of mobile elements, pruned, and filtered by stability and conservation on *o* = 5. H) CVTree on 445 bacteria. Filtered of mobile elements, pruned, and filtered by stability and conservation on *o* = 7.
(PDF)

S15 Fig D2 applied to bacteria. Trees rooted using ‘Root mid-point’ option in ITOL server. A) D2 on raw 495 bacteria. Unfiltered and unpruned. B) D2 on raw 445 bacteria. Unfiltered and pruned. C) D2 on 445 bacteria. Filtered of mobile elements and pruned. D) D2 on 445 bacteria. Filtered of mobile elements, pruned, and filtered by stability and conservation on *o* = 0. E) D2 on 445 bacteria. Filtered of mobile elements, pruned, and filtered by stability and conservation on *o* = 1. F) D2 on 445 bacteria. Filtered of mobile elements, pruned, and filtered by stability and conservation on *o* = 3. G) D2 on 445 bacteria. Filtered of mobile elements, pruned, and filtered by stability and conservation on *o* = 5. H) D2 on 445 bacteria. Filtered of mobile elements, pruned, and filtered by stability and conservation on *o* = 7.
(PDF)

S16 Fig kmacs applied to bacteria. Trees rooted using ‘Root mid-point’ option in ITOL server. A) kmacs on raw 495 bacteria. Unfiltered and unpruned. B) kmacs on raw 445 bacteria. Unfiltered and pruned. C) kmacs on 445 bacteria. Filtered of mobile elements and pruned. D) kmacs on 445 bacteria. Filtered of mobile elements, pruned, and filtered by stability and conservation on *o* = 0. E) kmacs on 445 bacteria. Filtered of mobile elements, pruned, and filtered by stability and conservation on *o* = 1. F) kmacs on 445 bacteria. Filtered of mobile elements, pruned, and filtered by stability and conservation on *o* = 3. G) kmacs on 445 bacteria. Filtered of mobile elements, pruned, and filtered by stability and conservation on *o* = 5. H) kmacs on 445 bacteria. Filtered of mobile elements, pruned, and filtered by stability and conservation on *o* = 7.
(PDF)

S17 Fig Spaced Words (SW) applied to bacteria. Trees rooted using ‘Root mid-point’ option in ITOL server. A) SW on raw 495 bacteria. Unfiltered and unpruned. B) SW on raw 445 bacteria. Unfiltered and pruned. C) SW on 445 bacteria. Filtered of mobile elements and pruned. D) SW on 445 bacteria. Filtered of mobile elements, pruned, and filtered by stability and conservation on *o* = 0. E) SW on 445 bacteria. Filtered of mobile elements, pruned, and filtered by stability and conservation on *o* = 1. F) SW on 445 bacteria. Filtered of mobile elements, pruned, and filtered by stability and conservation on *o* = 3. G) SW on 445 bacteria. Filtered of mobile elements, pruned, and filtered by stability and conservation on *o* = 5. H) SW on 445 bacteria. Filtered of mobile elements, pruned, and filtered by stability and conservation on *o* = 7.
(PDF)

S18 Fig ALFRED-G (ALF) applied to bacteria. Trees rooted using ‘Root mid-point’ option in ITOL server. Only a partial set of trees was calculated due to the long run-time of the program. A) ALF on 445 bacteria. Filtered of mobile elements, pruned, and filtered by stability and conservation on *o* = 5. B) ALF on 445 bacteria. Filtered of mobile elements, pruned, and filtered by stability and conservation on *o* = 7.
(PDF)

S19 Fig Examples of 2 main classes of large-scale HGT and their corrections. A) A pair sharing a single copy phage. B) A pair sharing large-scale transfer of proteins associated with adaptation to environment. C) For pair sharing phage, effect on plots that mobile-element filtering combined with conservation filtering have, compared to the pair-wise HGT correction. D) For a pair sharing adaptive proteins, effect on plots that mobile element filtering and conservation filtering have compared to the pair-wise HGT correction.
(PDF)

S1 Table Distance to Eisen trees for SlopeTree and for six other whole-genome methods, over different levels of mobile-element and conservation filtering. (XLSX)

S1 Text Bacteria pruned from the trees due to having reduced genomes, candidate status, missing conserved genes, copy number of conserved genes significantly different from average, an excessive number of FASTA files, or a discrepancy identified by the mobile element filter (i.e. unusual copy number pattern for majority of proteins). A) Pruned for archaea. B) Pruned for bacteria.
(DOC)

S2 Text The manually selected reference organisms for the SlopeTree runs that required a reference (NCBI classification information included). A) Archaea. B) Bacteria.
(DOCX)
