# Supplementary figures and images for: Phylogeny Reconstruction with Alignment-Free Method That Corrects for Horizontal Gene Transfer
Source: PLoS Comput Biol. 2016 Jun 23;12(6):e1004985. doi: 10.1371/journal.pcbi.1004985 (PMC4918981; doi:10.1371/journal.pcbi.1004985)

Mobile-element (Algorithm 1) and conservation/stability (Algorithm 2) filtering

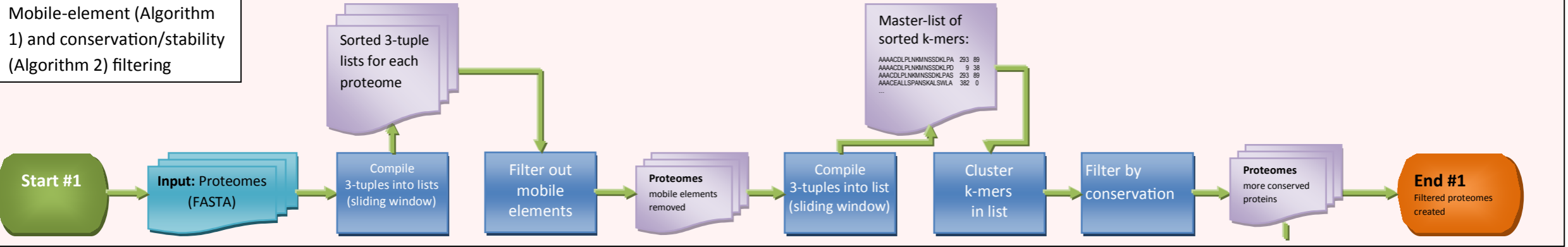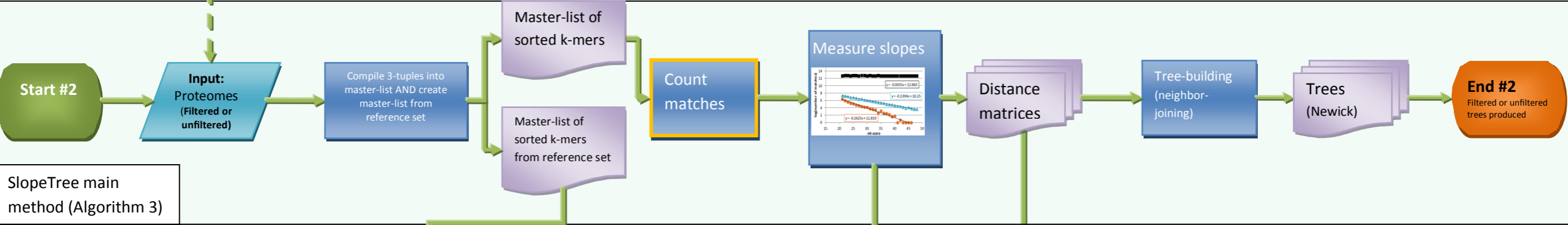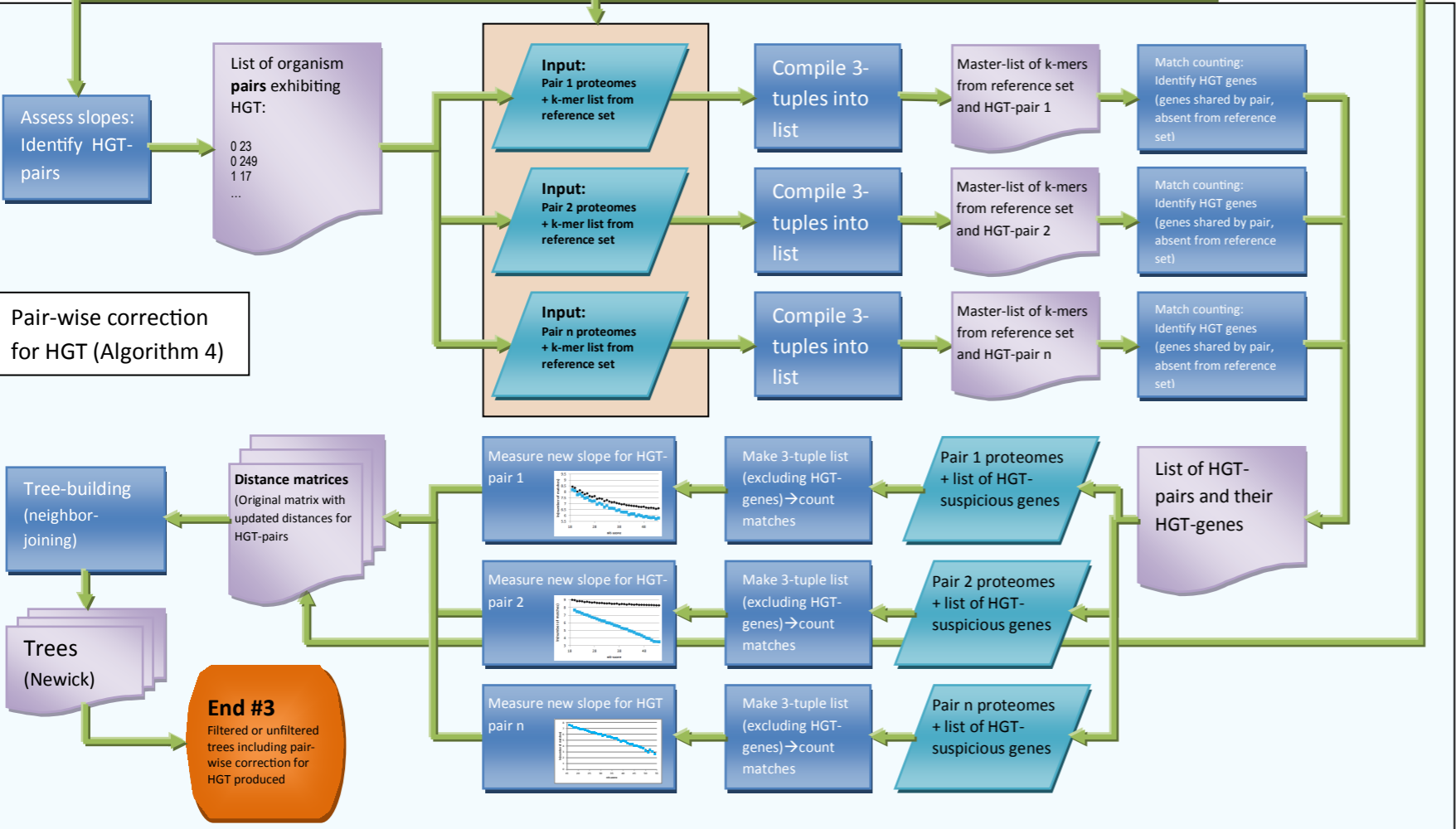

Supplement: S1 Fig — SlopeTree has 3 main parts: The mobile-element filtering (Algorithm 1) and the conservation/stability filtering (Algorithm 2); the SlopeTree main method (Algorithm 3) which produces a distance matrix and tree; and the pair-wise HGT correction (Algorithm 4) which reprocesses pairs that were flagged as showing signs of HGT. When not using mobile-element filtering or conservation filtering, Start #2 is the original starting point. Three pairs are shown for the pair-wise HGT correction code; this number can be in the 100s or 1000s depending on the input set. All proteomes are in FASTA format. (PDF) [file pcbi.1004985.s001.pdf]

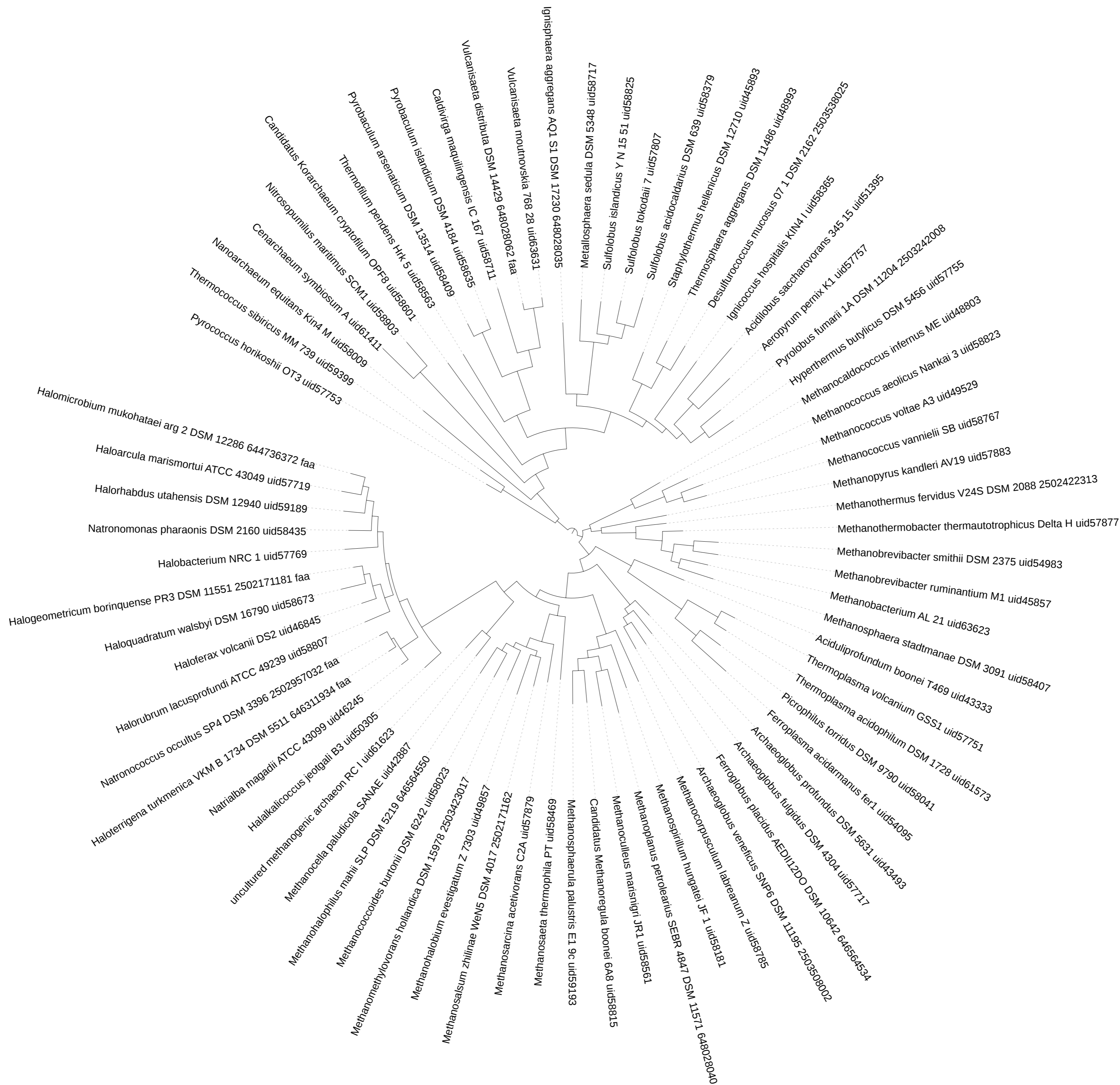

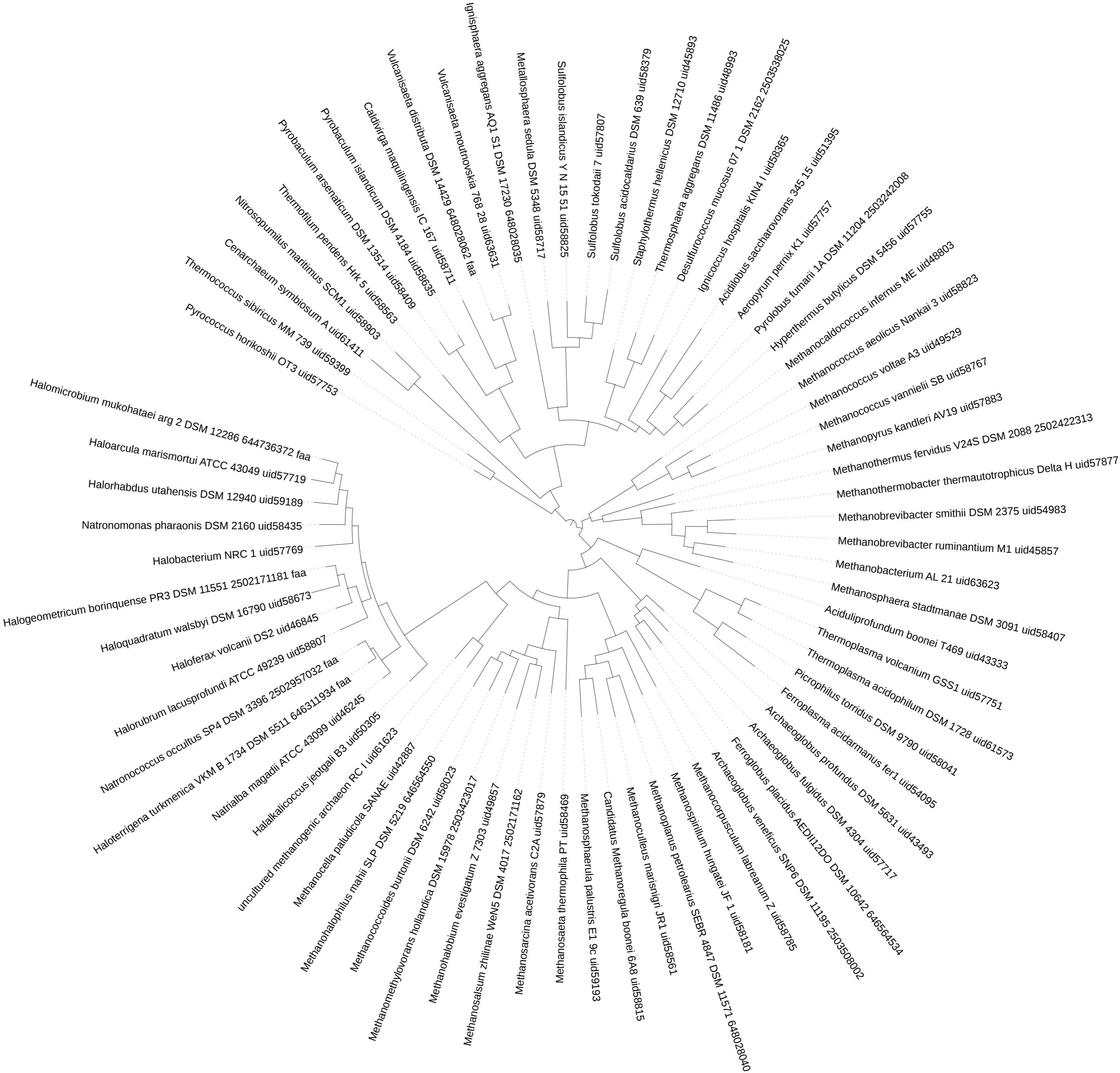

Tree scale: 0.1

C

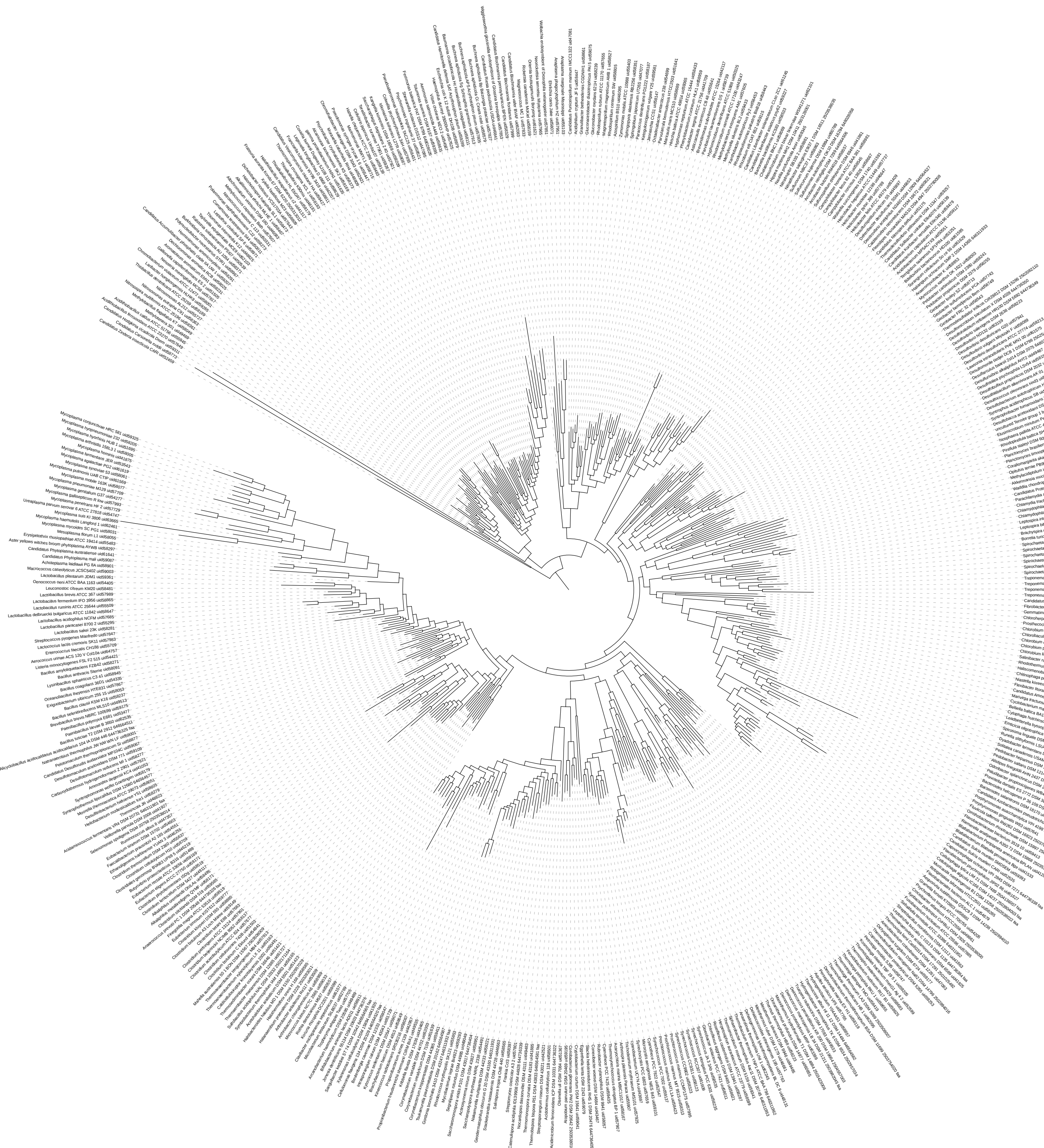

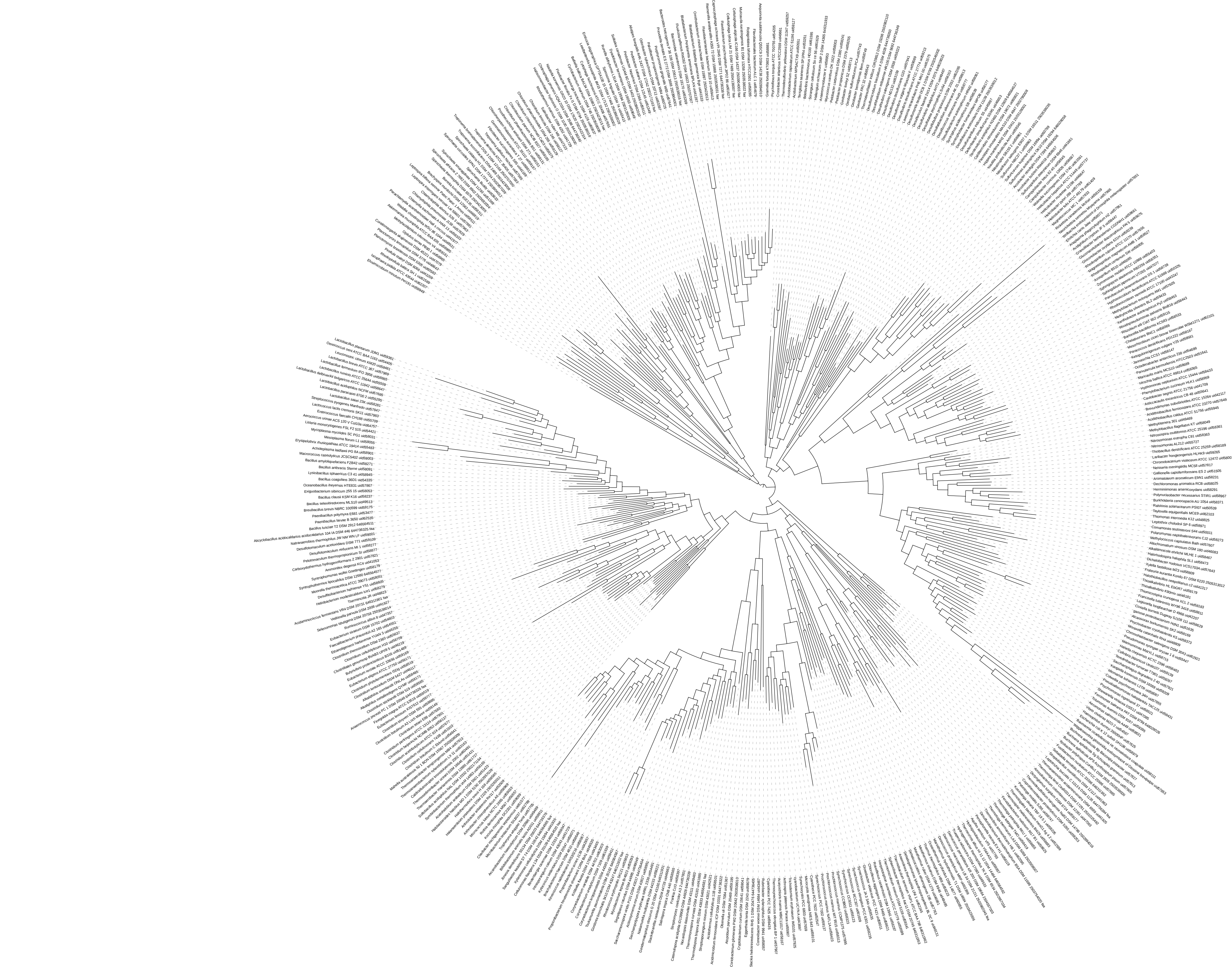

Supplement: S2 Fig — A) Eisen-73 tree. B) Eisen-71 tree (pruned Eisen-73). C) Eisen-495 tree. D) Eisen-445 tree (pruned Eisen-495). (PDF) [file pcbi.1004985.s002.pdf]

A)

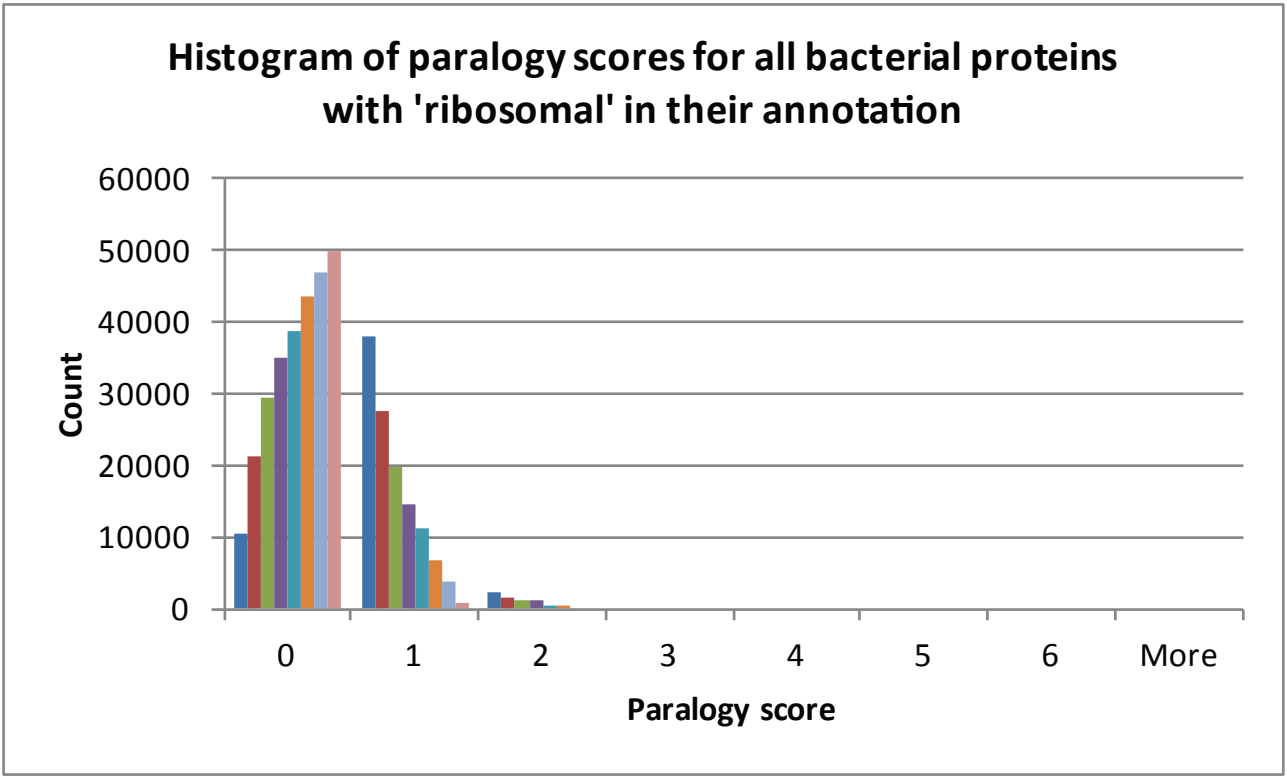

B)

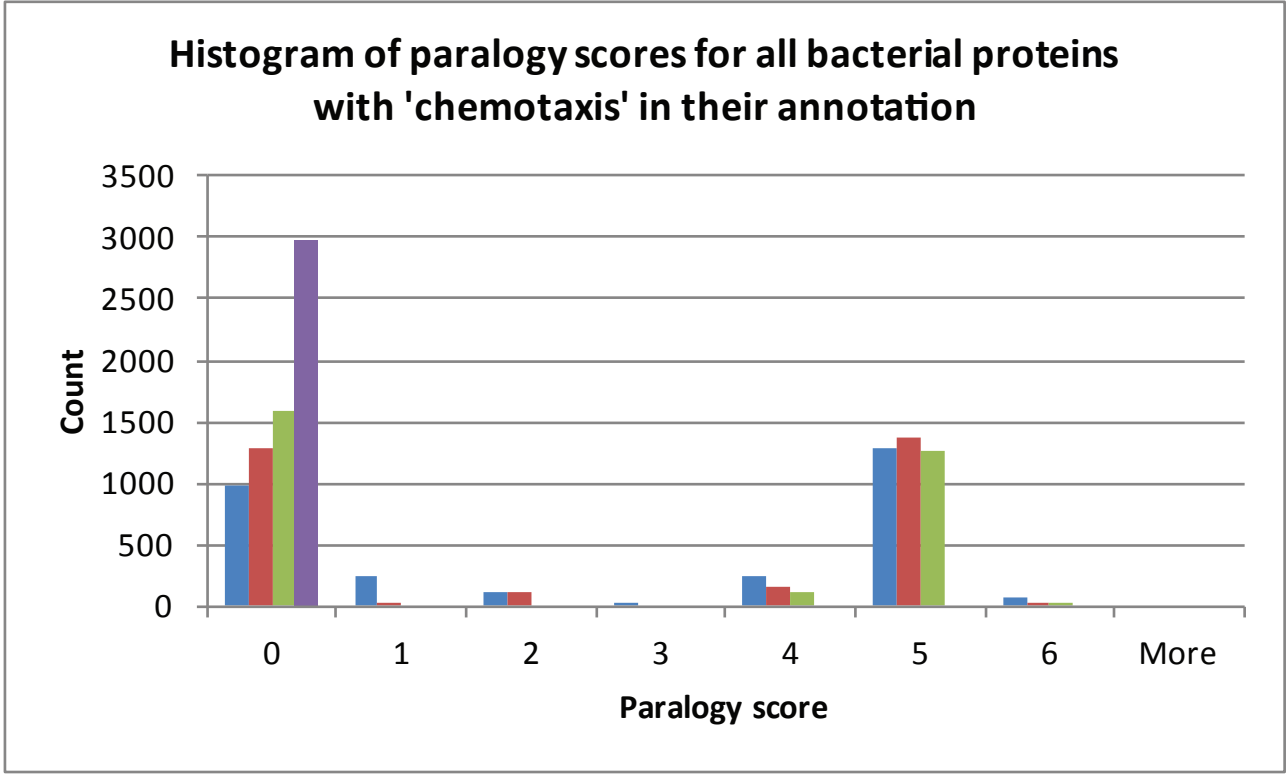

Supplement: S3 Fig — A) Histogram for all proteins with ‘ribosomal’ in their annotation, i.e. an example of paralogy scores for a highly conserved protein. B) Histogram for all proteins with ‘chemotaxis’ in their annotation, i.e. an example of paralogy scores for a non-conserved, frequently transferred protein. (PDF) [file pcbi.1004985.s003.pdf]

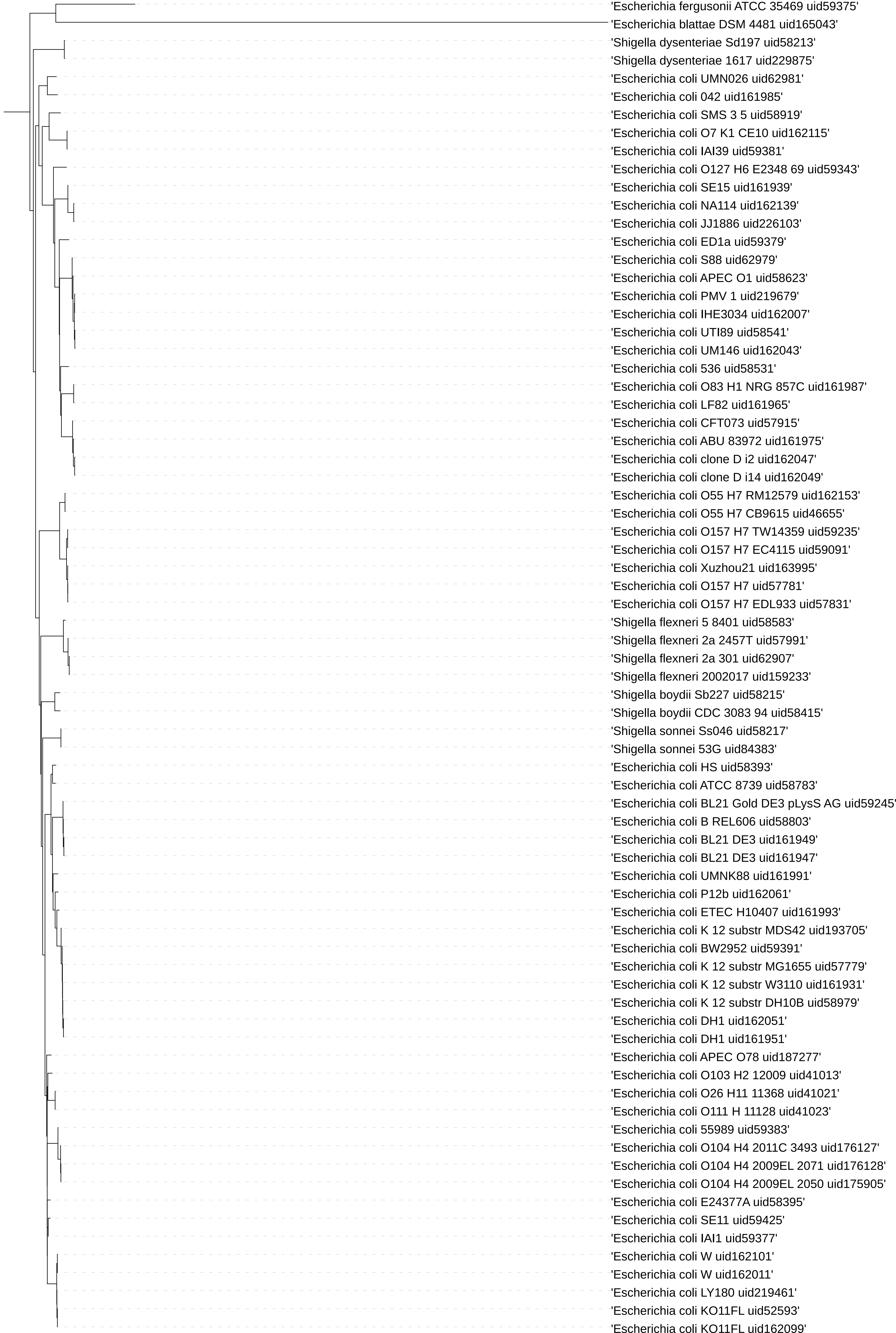

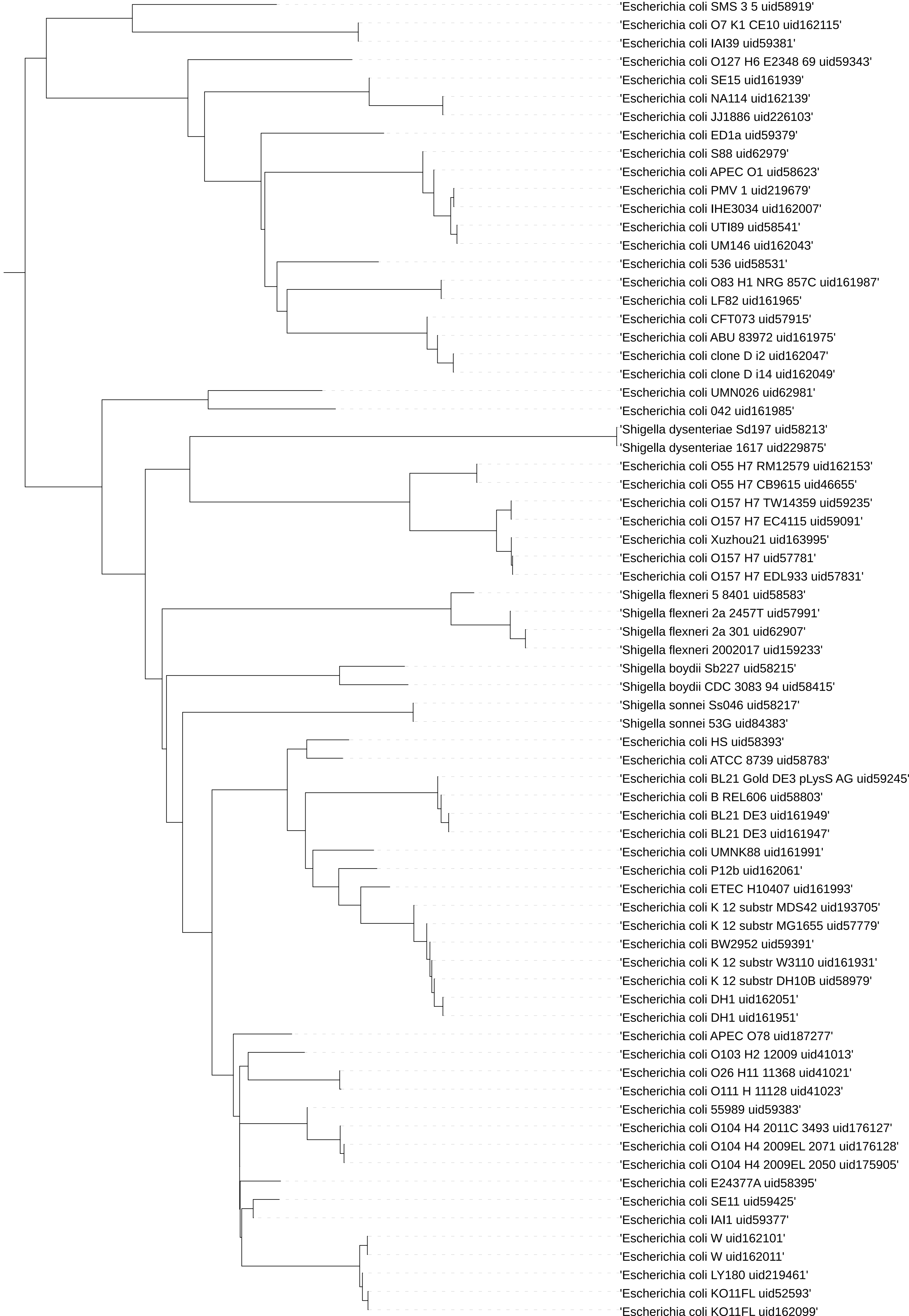

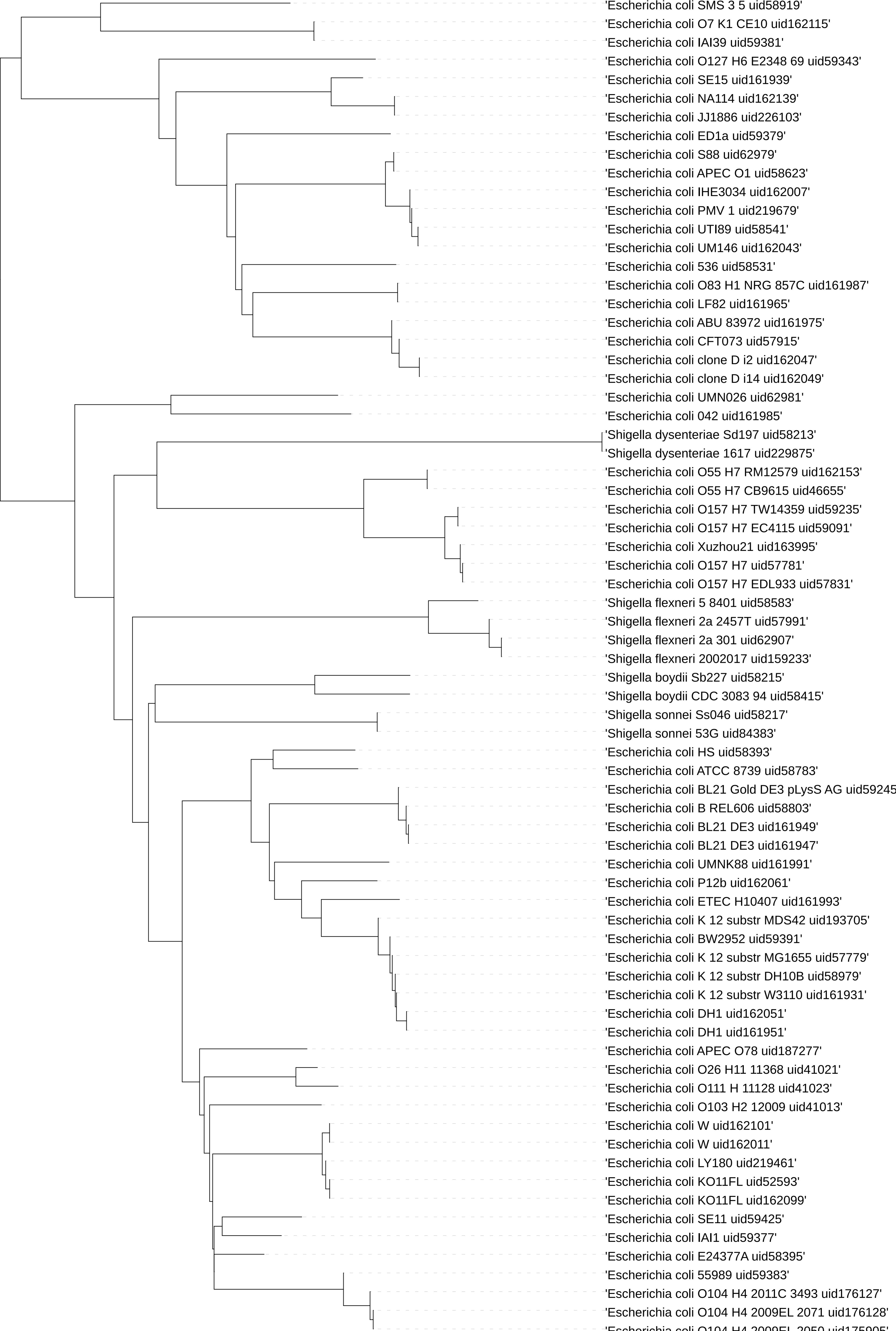

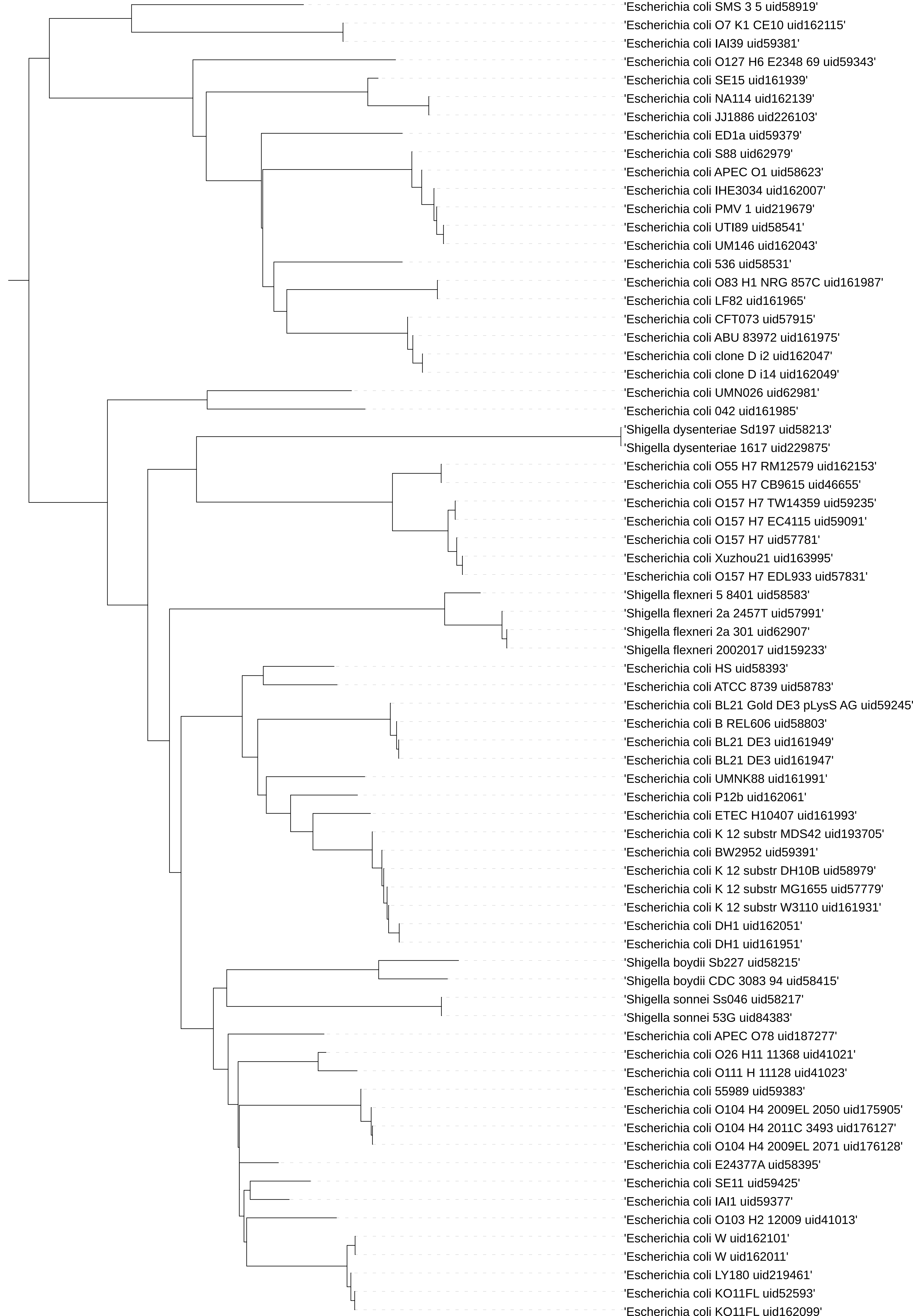

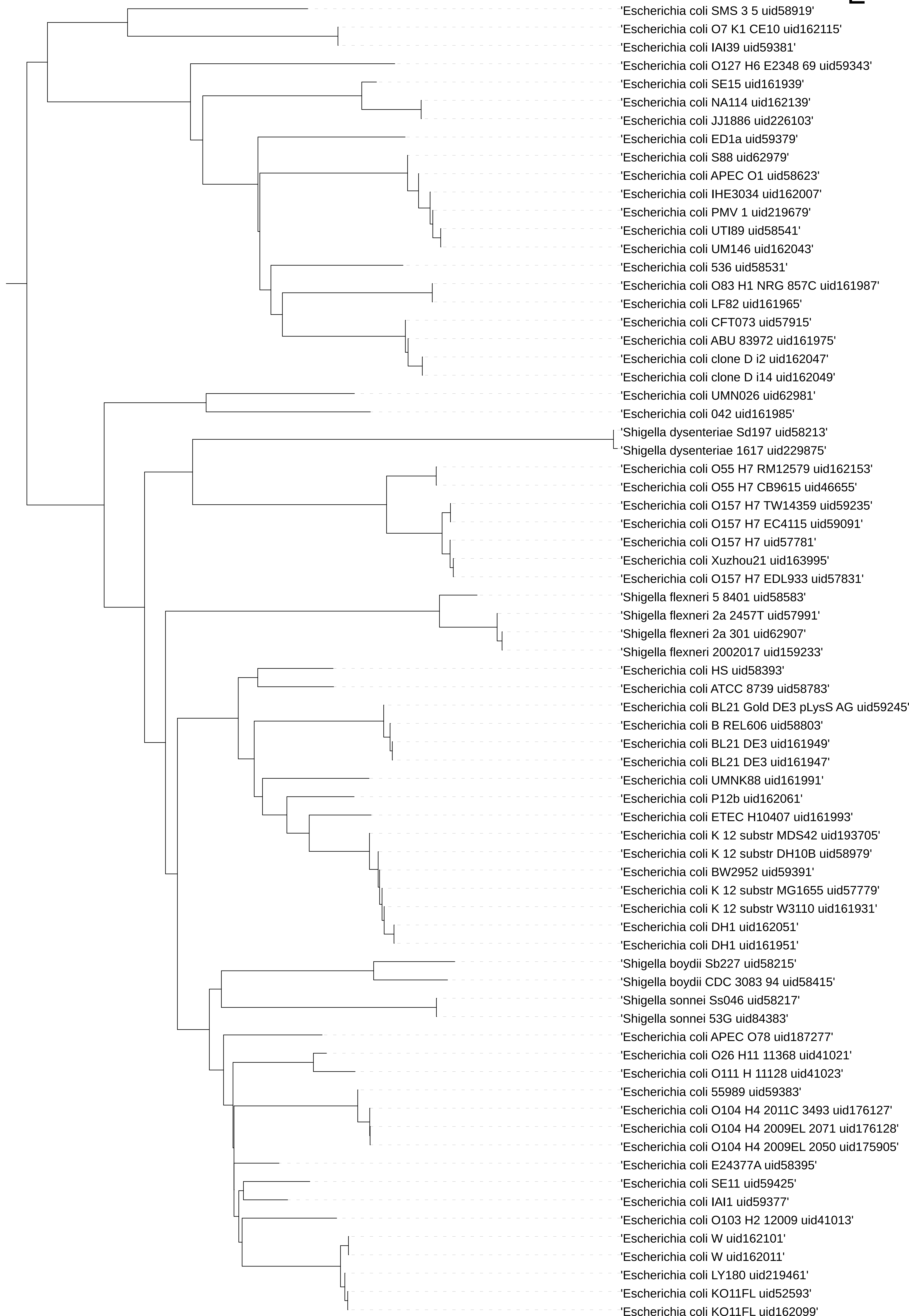

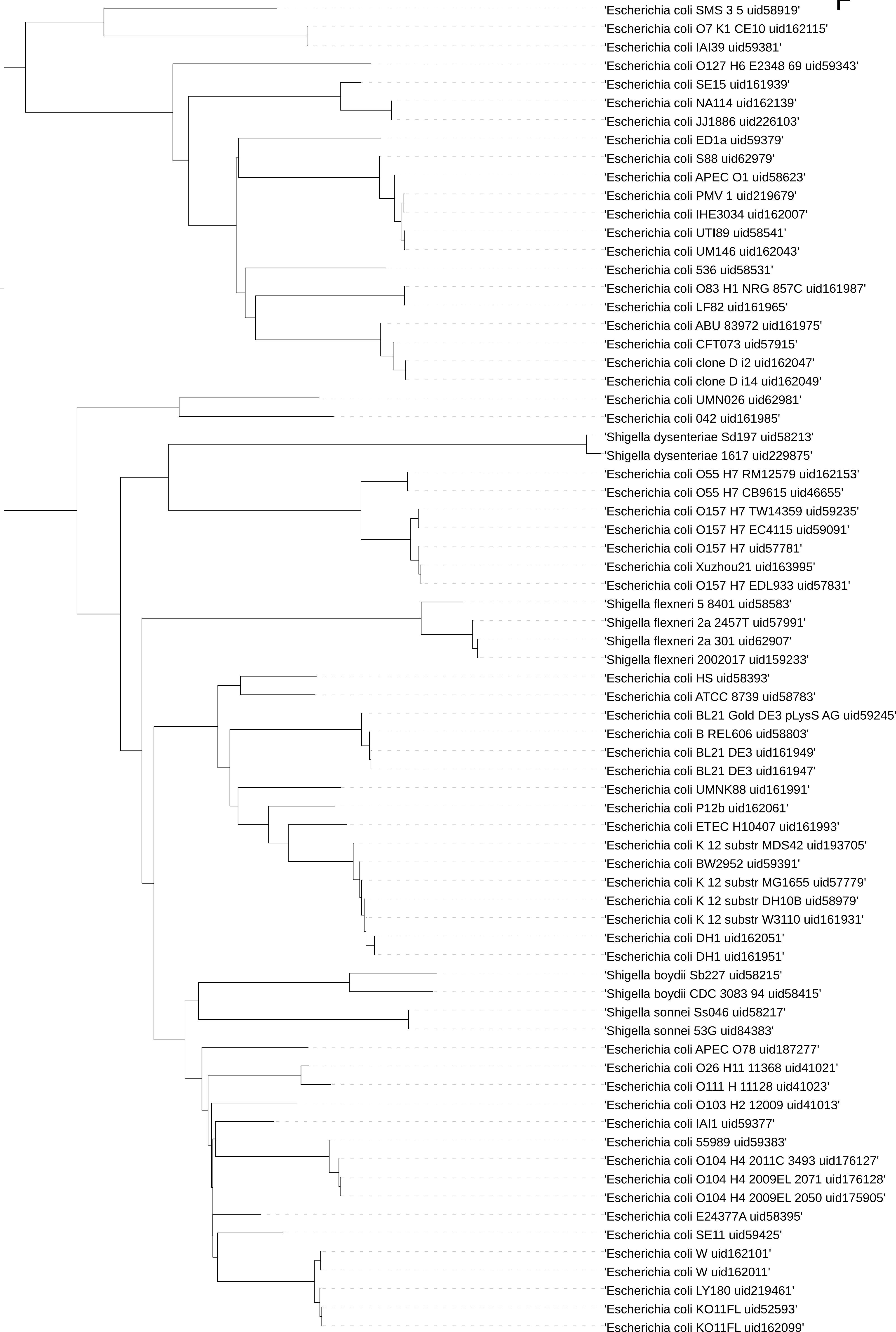

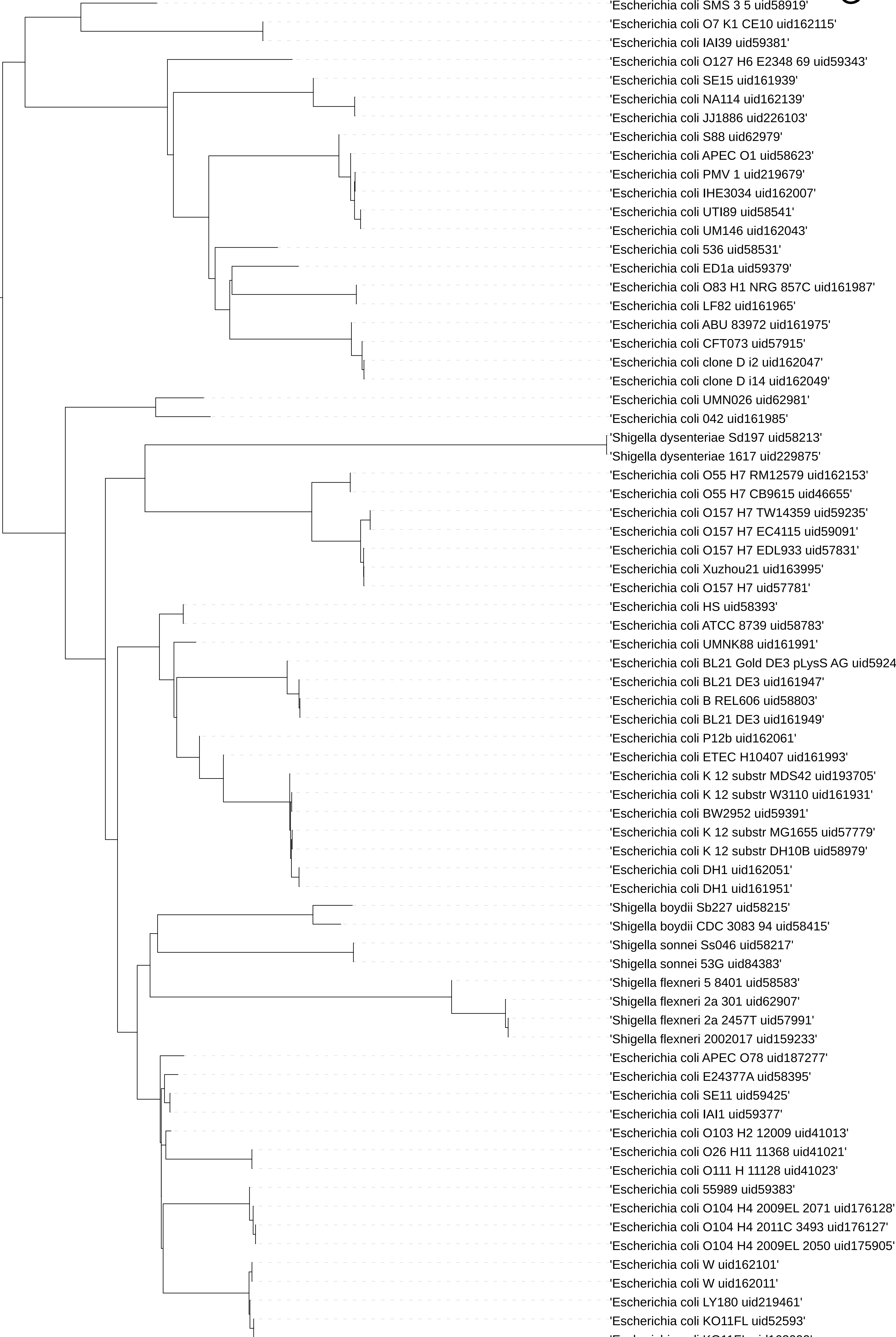

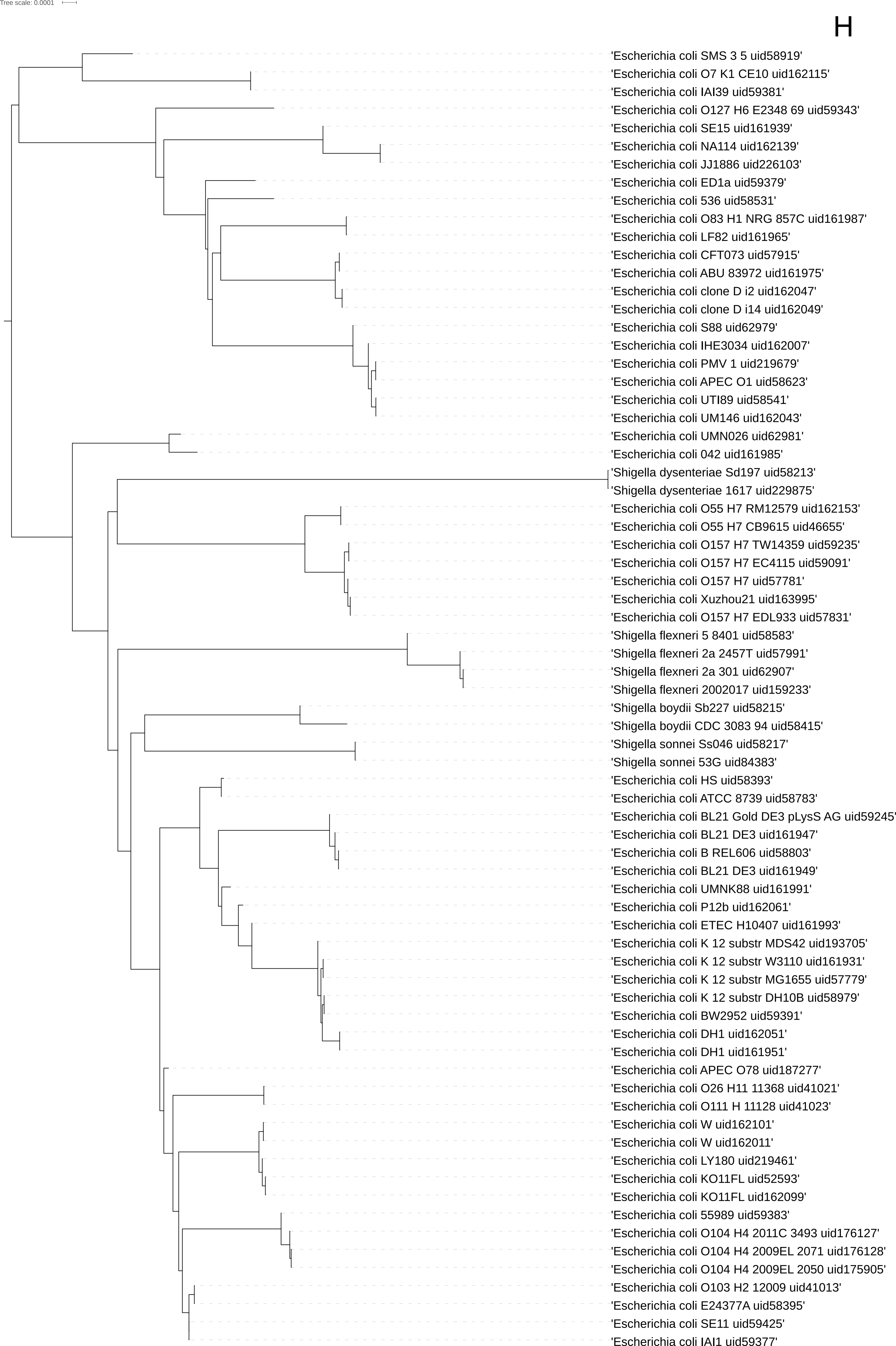

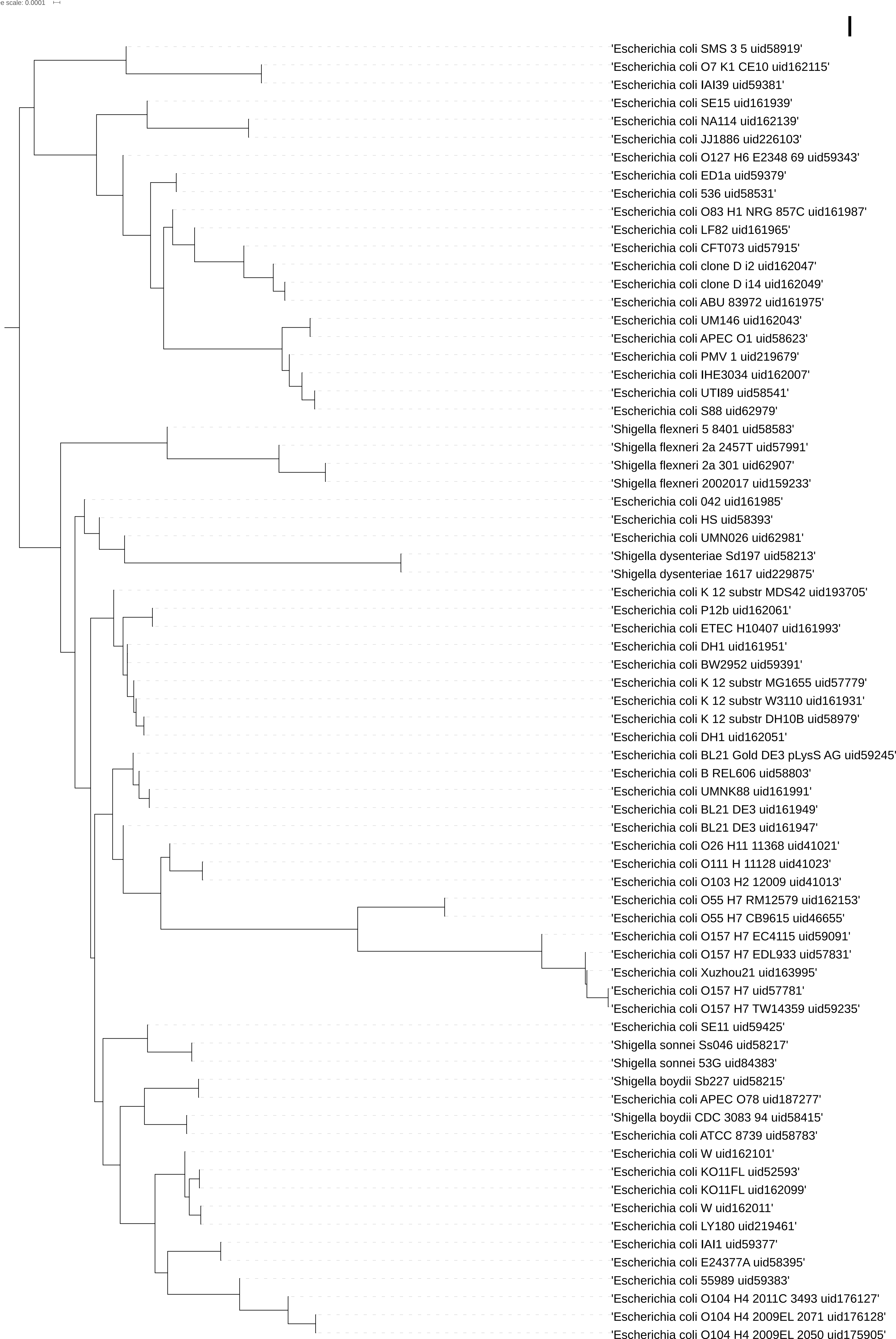

Supplement: S4 Fig — Full set used consisted of 72 Escherichia coli, 10 Shigella (4 S. flexneri, 2 S. boydii, and 2 S. sonnei), Escherichia fergusonii, and Escherichia blattae. All trees used 20-mers unless otherwise specified. Due to the closeness of some organisms, some distances in the final Newick trees were negative; these were changed to 0 to avoid ‘backwards’ branches. This had no effect on the topology. A) ST on 72 E. coli, 10 Shigella, E. fergusonii and E. blattae. B) ST on 72 E. coli and 10 Shigella. C) ST using 40-mers on 72 E.coli and 10 Shigella. D) ST on 72 E. coli and 10 Shigella, filtered for mobile elements. E) ST on 72 E. coli and 10 Shigella, filtered for mobile elements and self-filtered on o = 0. F) ST on 72 E. coli and 10 Shigella, filtered for mobile elements and self-filtered on o = 5. G) ST on 72 E. coli and 10 Shigella, filtered for mobile elements and self-filtered on o = 10. H) ST on 72 E. coli and 10 Shigella, filtered for mobile elements and filtered against a reference set of 30 diverse bacteria on o = 3. I) ST on 72 E. coli and 10 Shigella, built from mobile elements and proteins discarded when self-filtering on o = 0. (PDF) [file pcbi.1004985.s004.pdf]

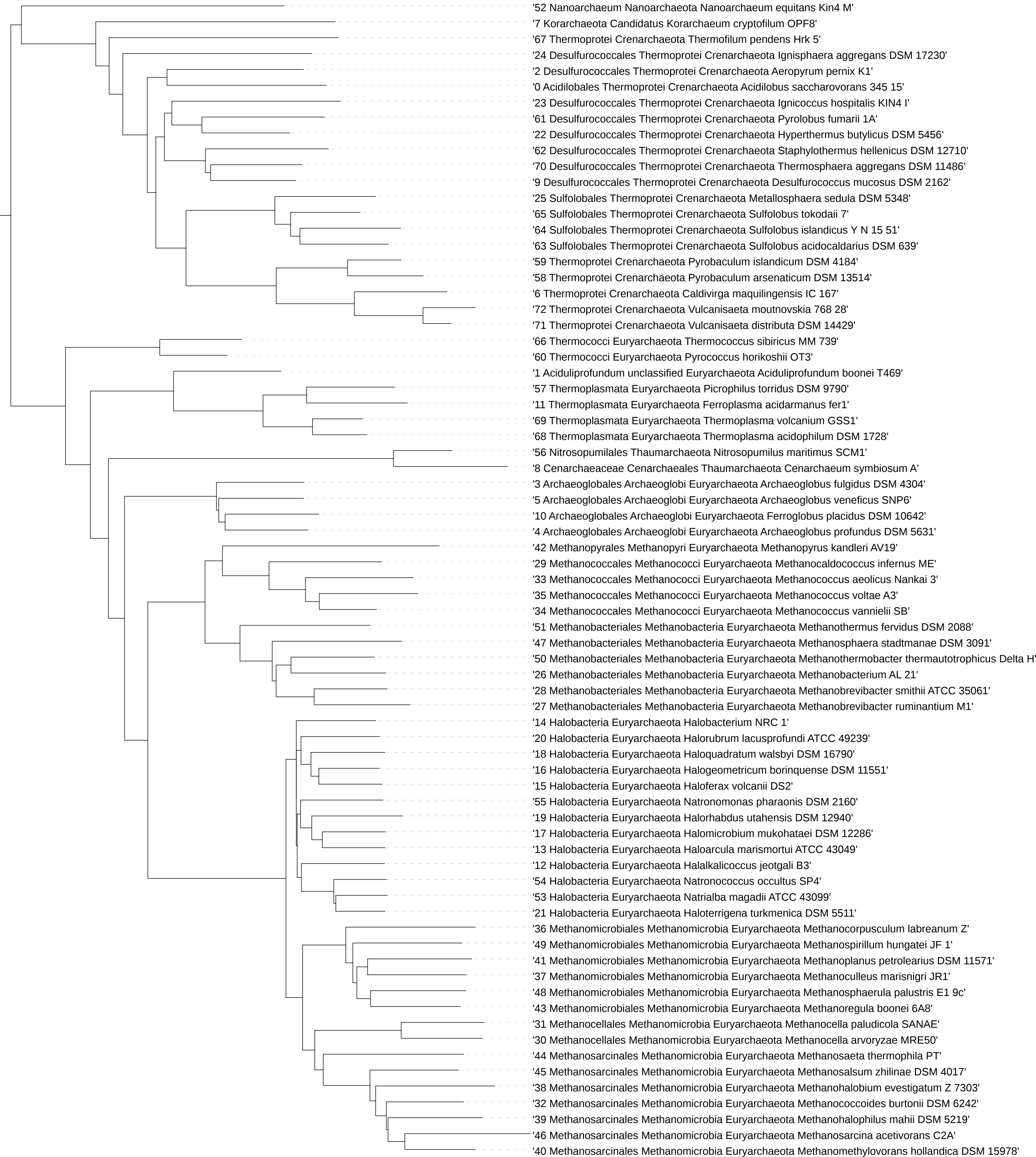

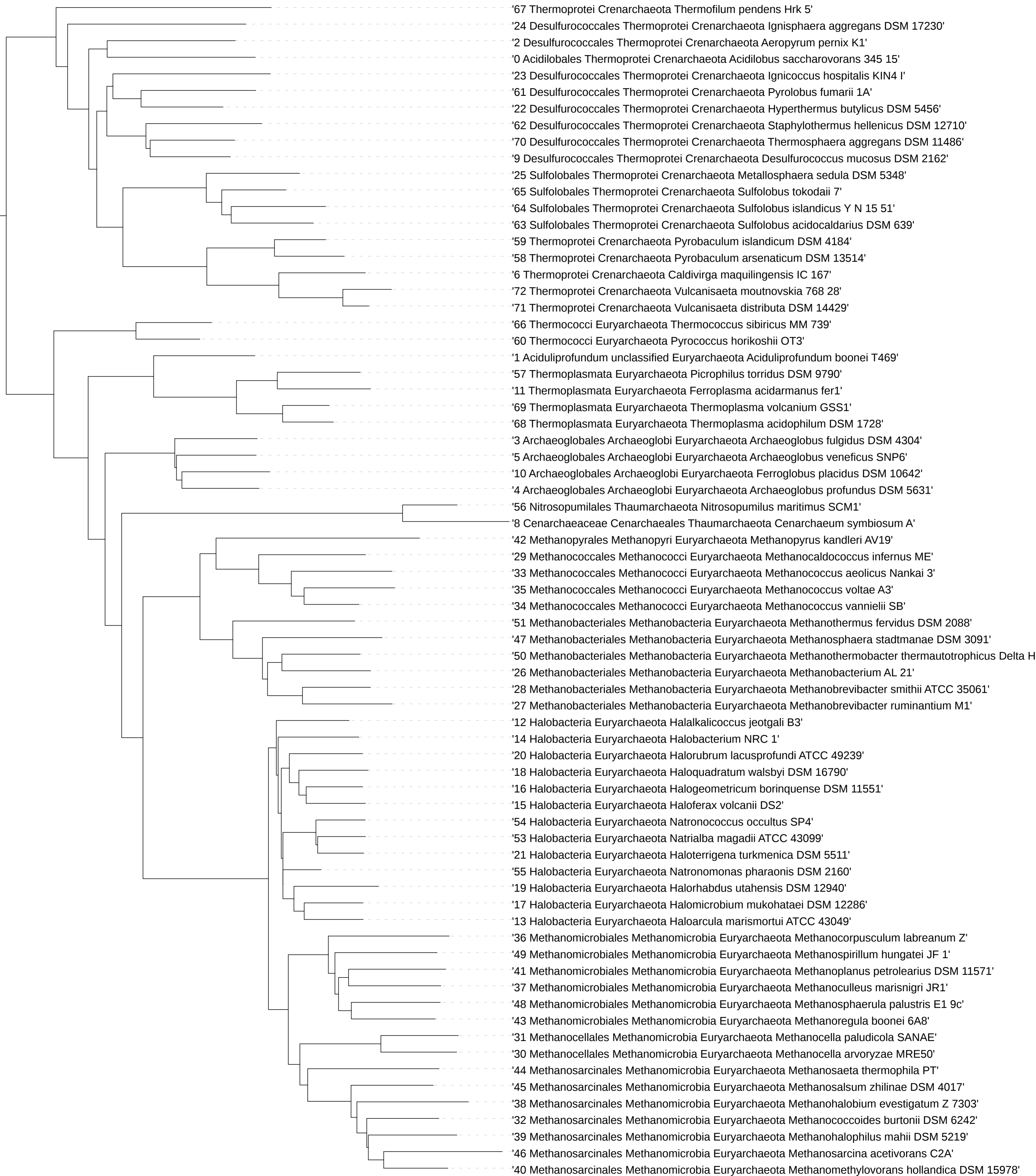

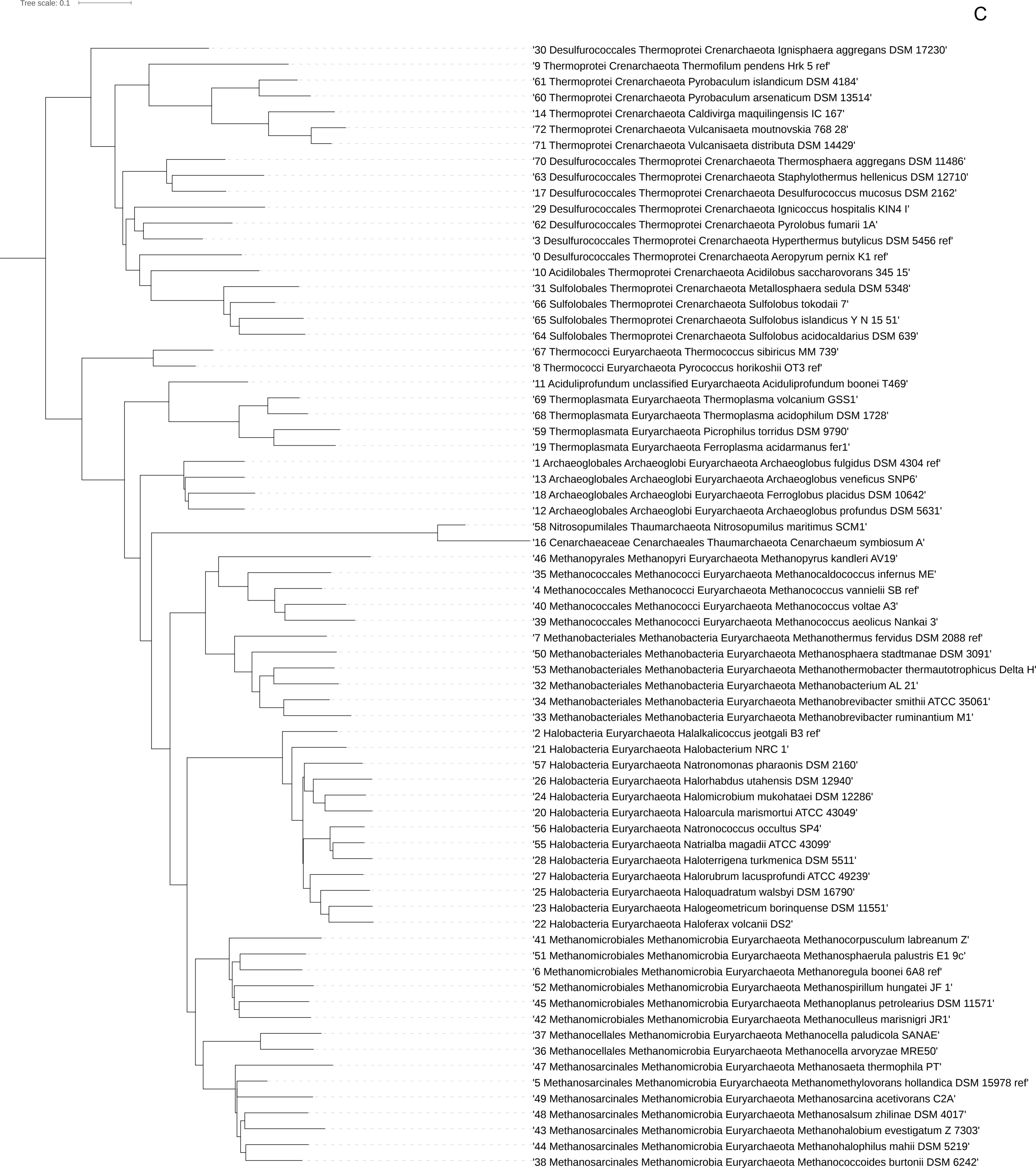

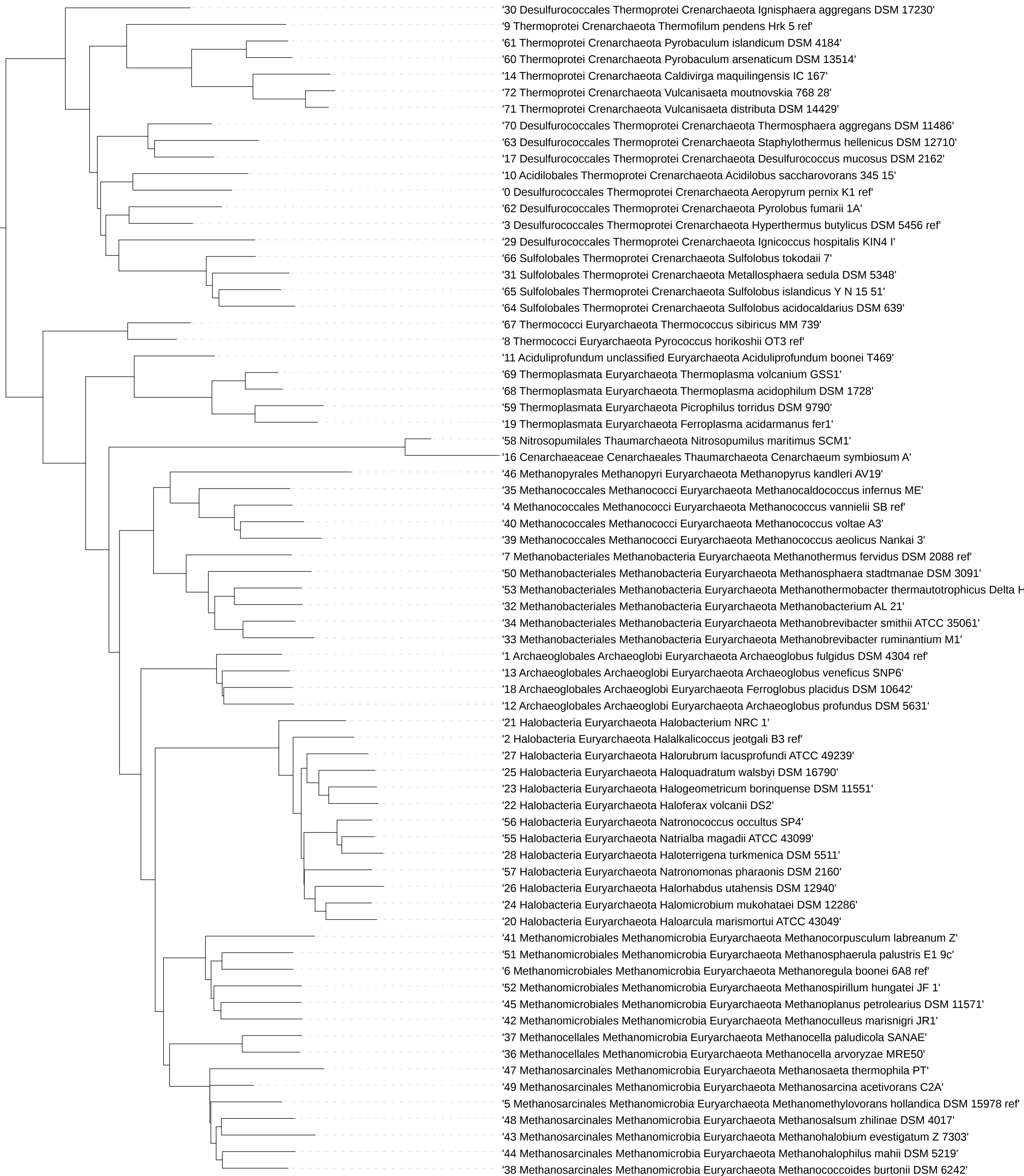

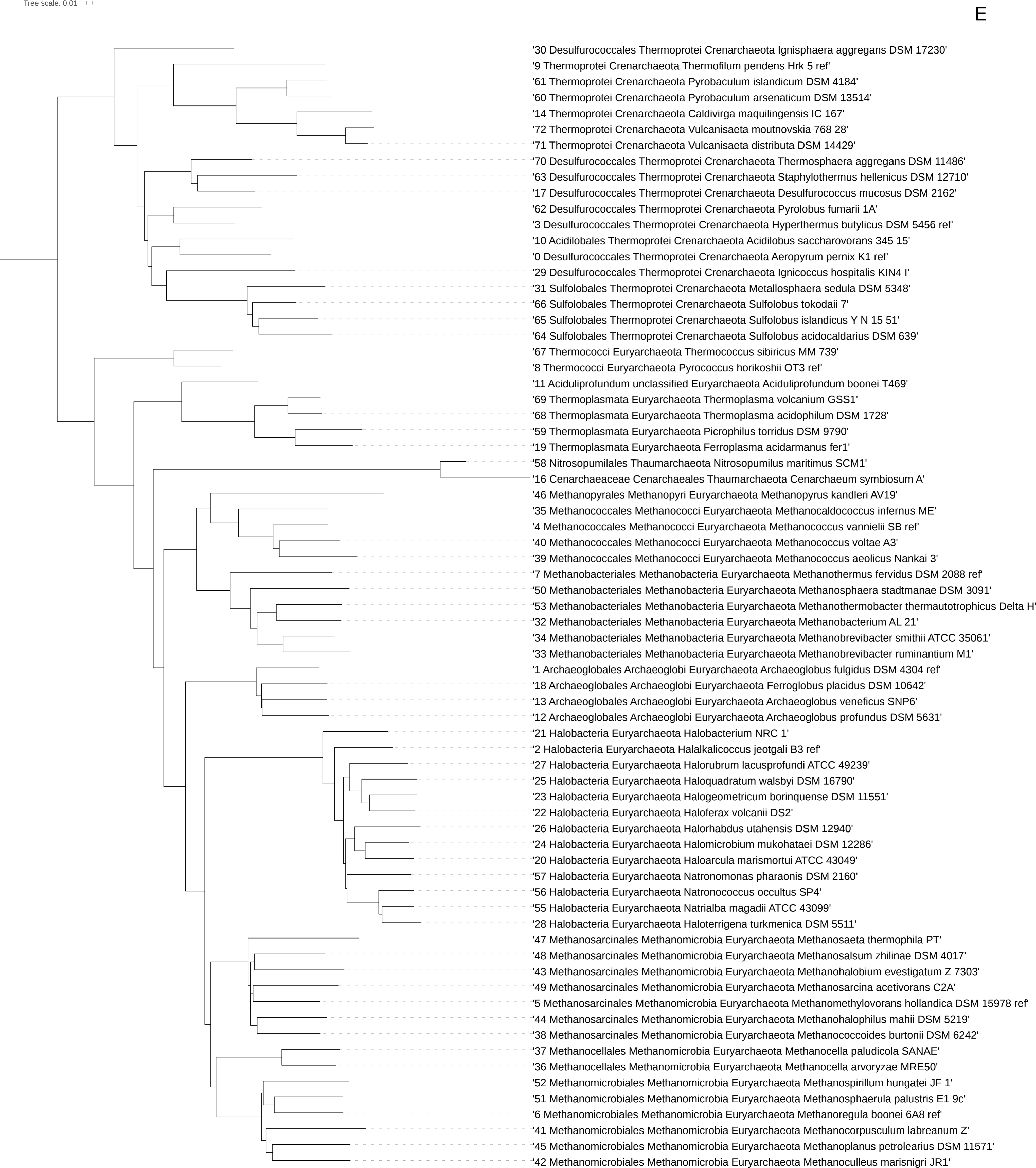

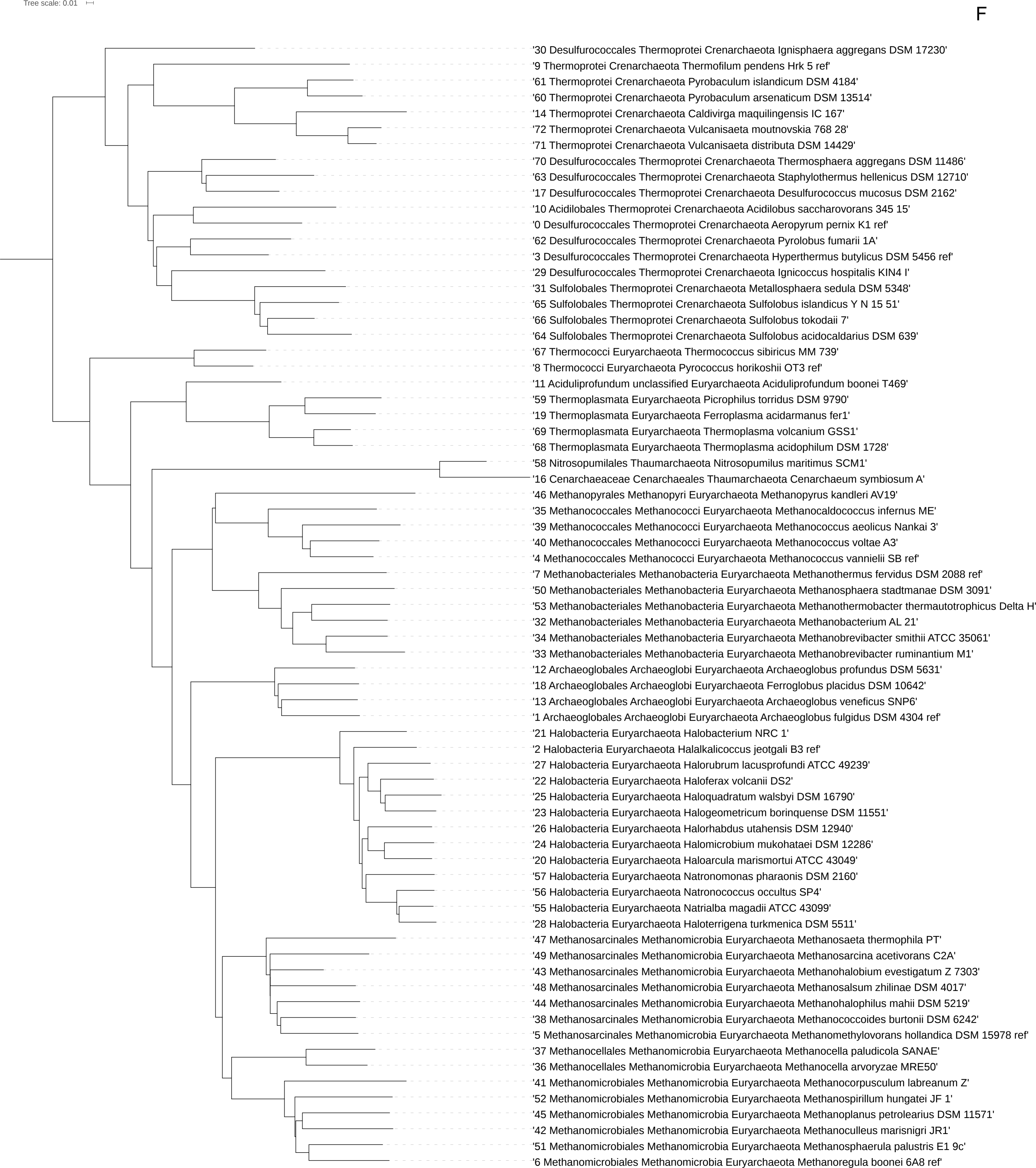

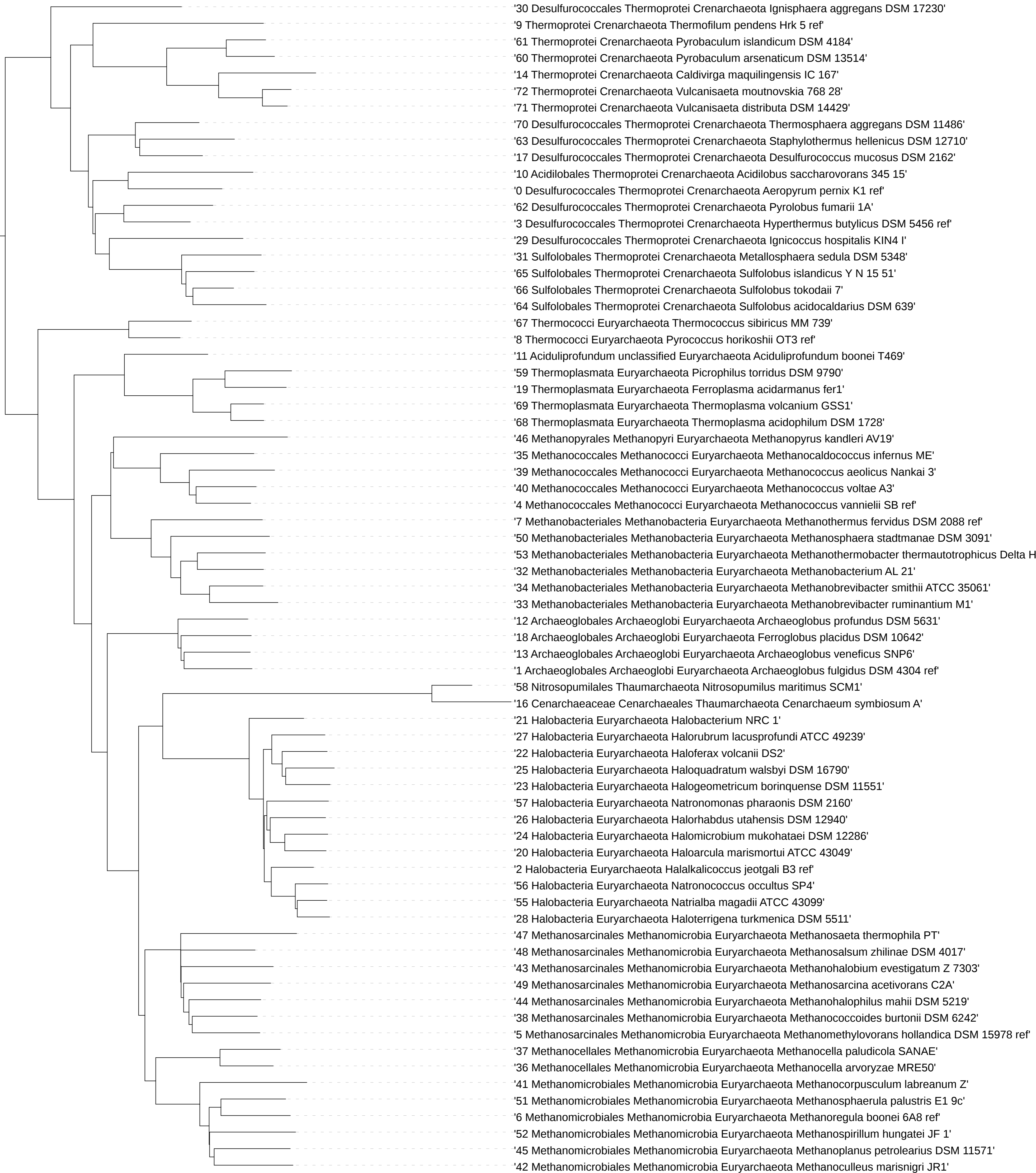

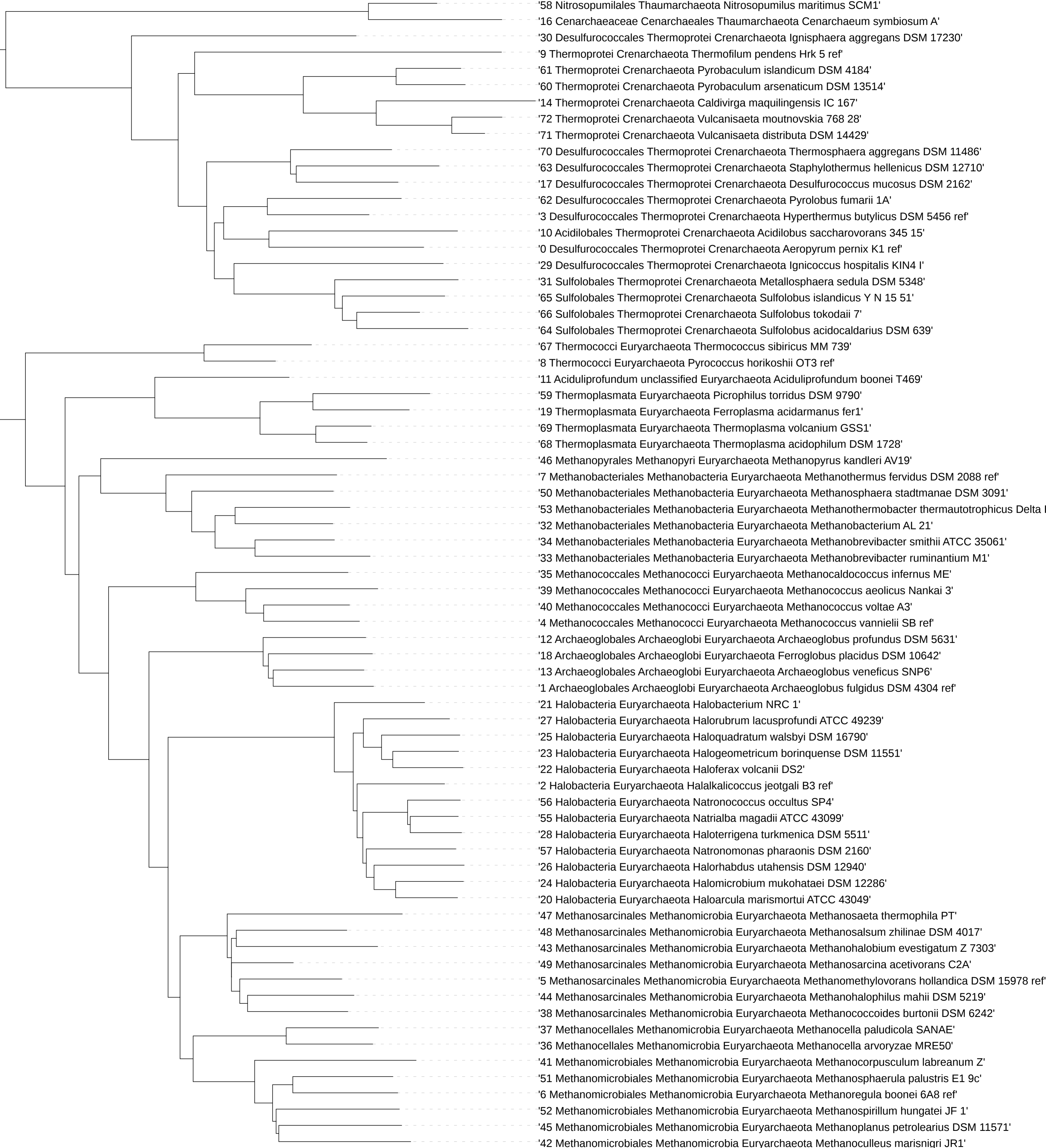

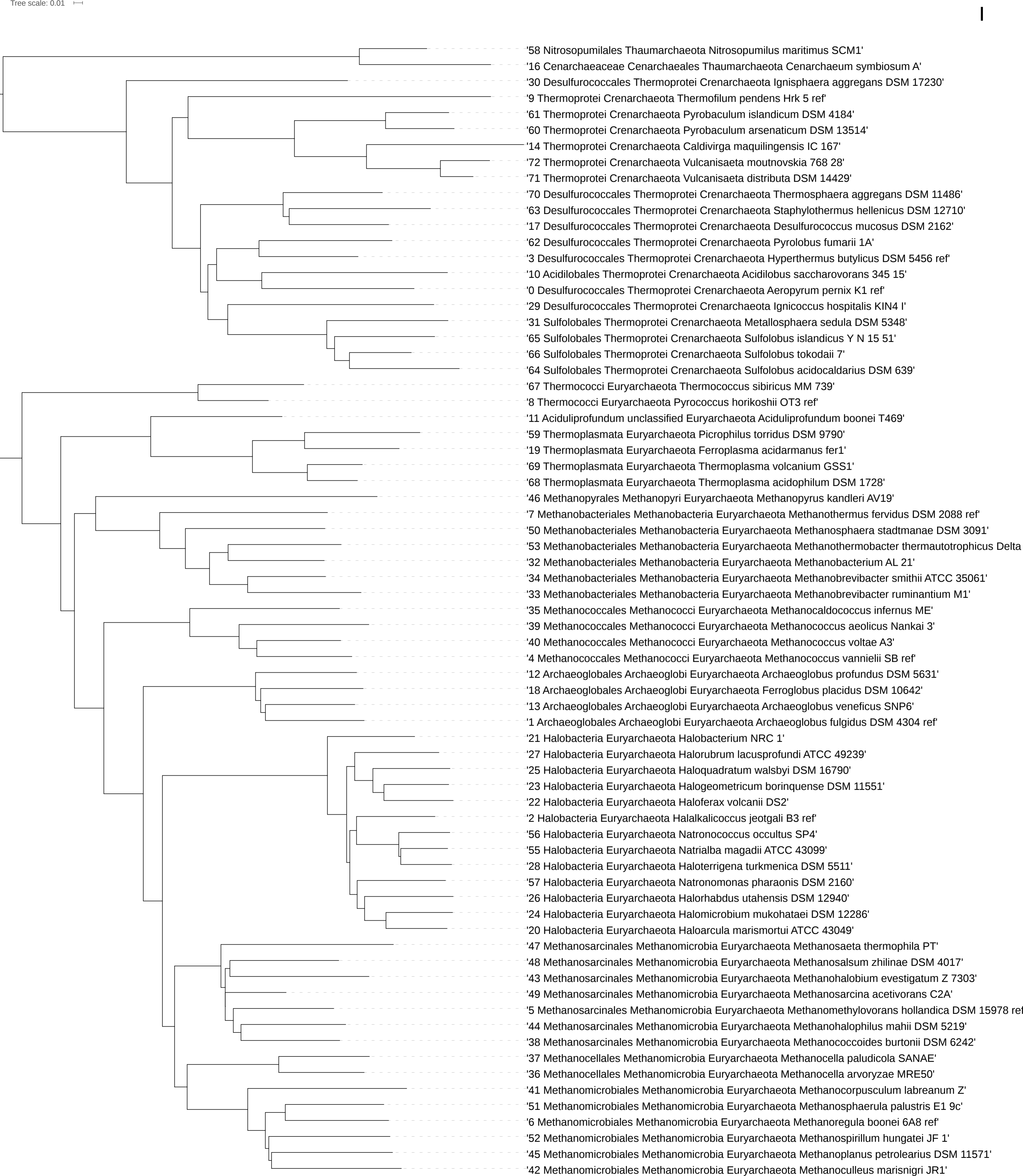

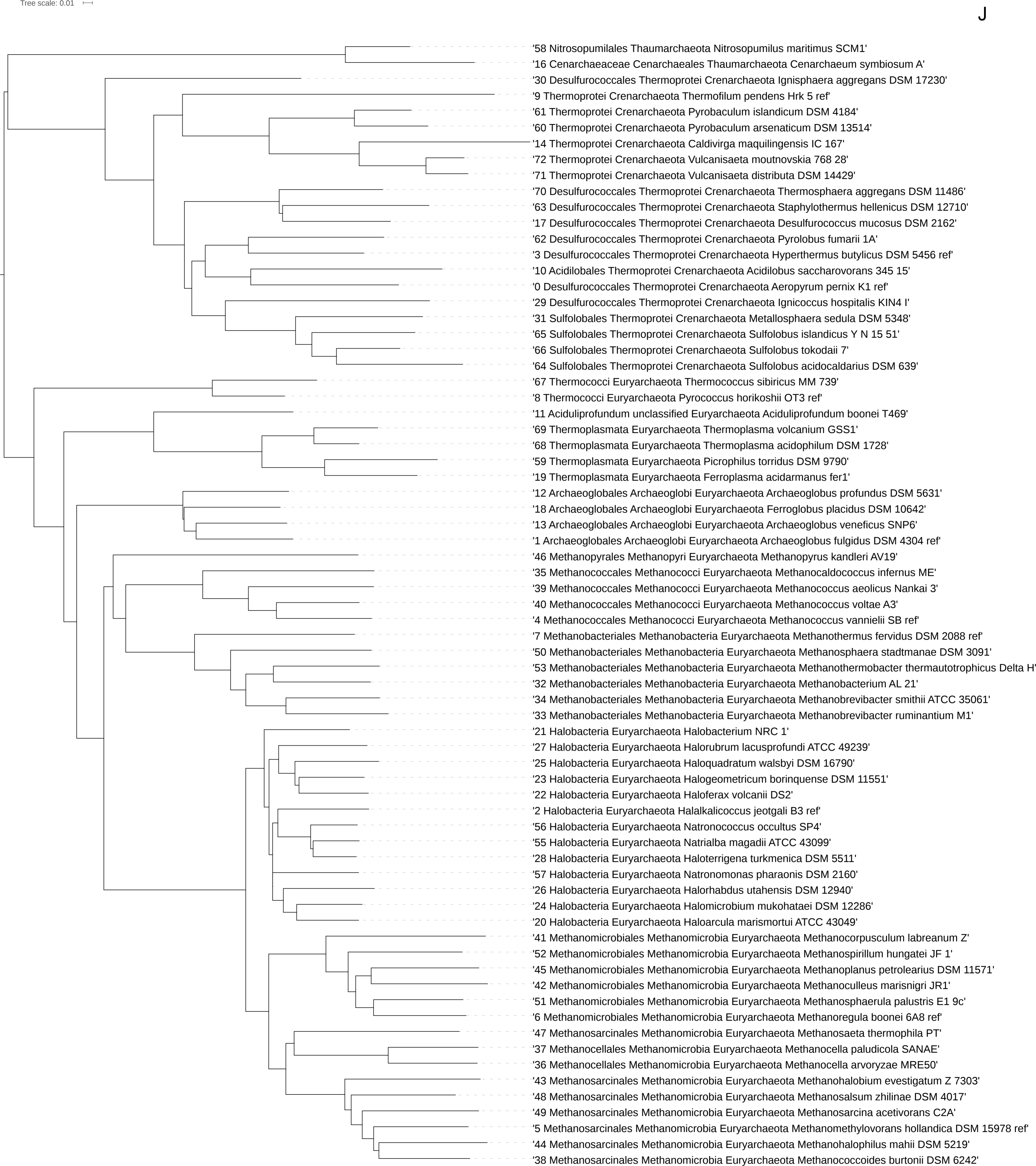

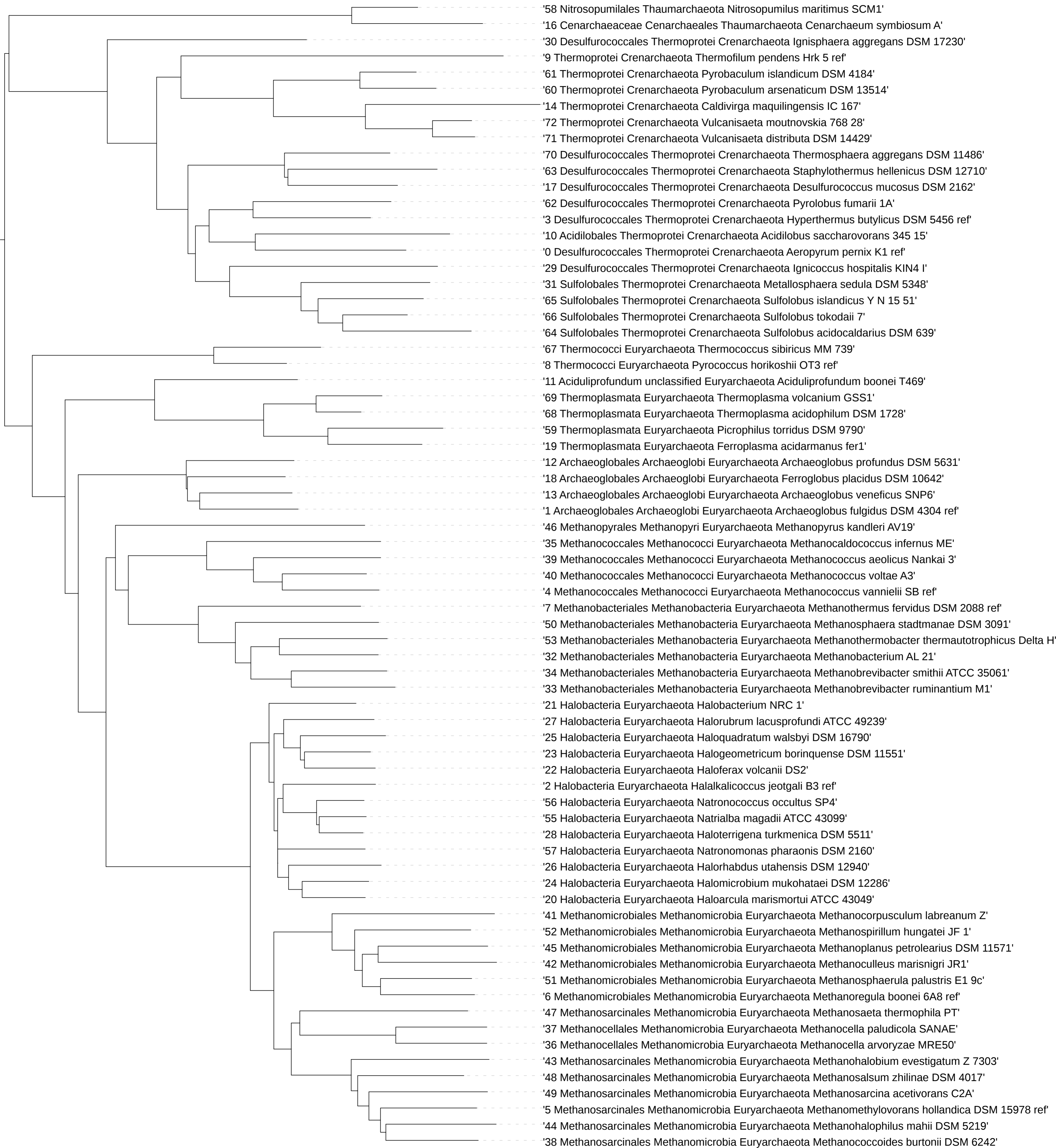

Supplement: S5 Fig — A) Raw ST-tree for 73 archaea. Unfiltered and unpruned. B) Raw ST-tree for 71 archaea. Unfiltered and pruned. C) ST- tree for 71 archaea. Filtered of mobile elements and pruned. D) ST-tree for 71 archaea. Filtered of mobile elements, pruned, and filtered by stability and conservation on o = 0. E) ST-tree for 71 archaea. Filtered of mobile elements, pruned, and filtered by stability and conservation on o = 1. F) ST-tree for 71 archaea. Filtered of mobile elements, pruned, and filtered by stability and conservation on o = 3. G) ST-tree for 71 archaea. Filtered of mobile elements, pruned, filtered by stability and conservation on o = 3, and final pair-wise HGT correction applied. H) ST-tree for 71 archaea. Filtered of mobile elements, pruned, and filtered by stability and conservation on o = 5. I) ST-tree for 71 archaea. Filtered of mobile elements, pruned, filtered by stability and conservation on o = 5, and final pair-wise HGT correction applied. J) ST-tree for 71 archaea. Filtered of mobile elements, pruned, and filtered by stability and conservation on o = 7. K) ST-tree for 71 archaea. Filtered of mobile elements, pruned, filtered by stability and conservation on o = 7, and final pair-wise HGT correction applied. (PDF) [file pcbi.1004985.s005.pdf]

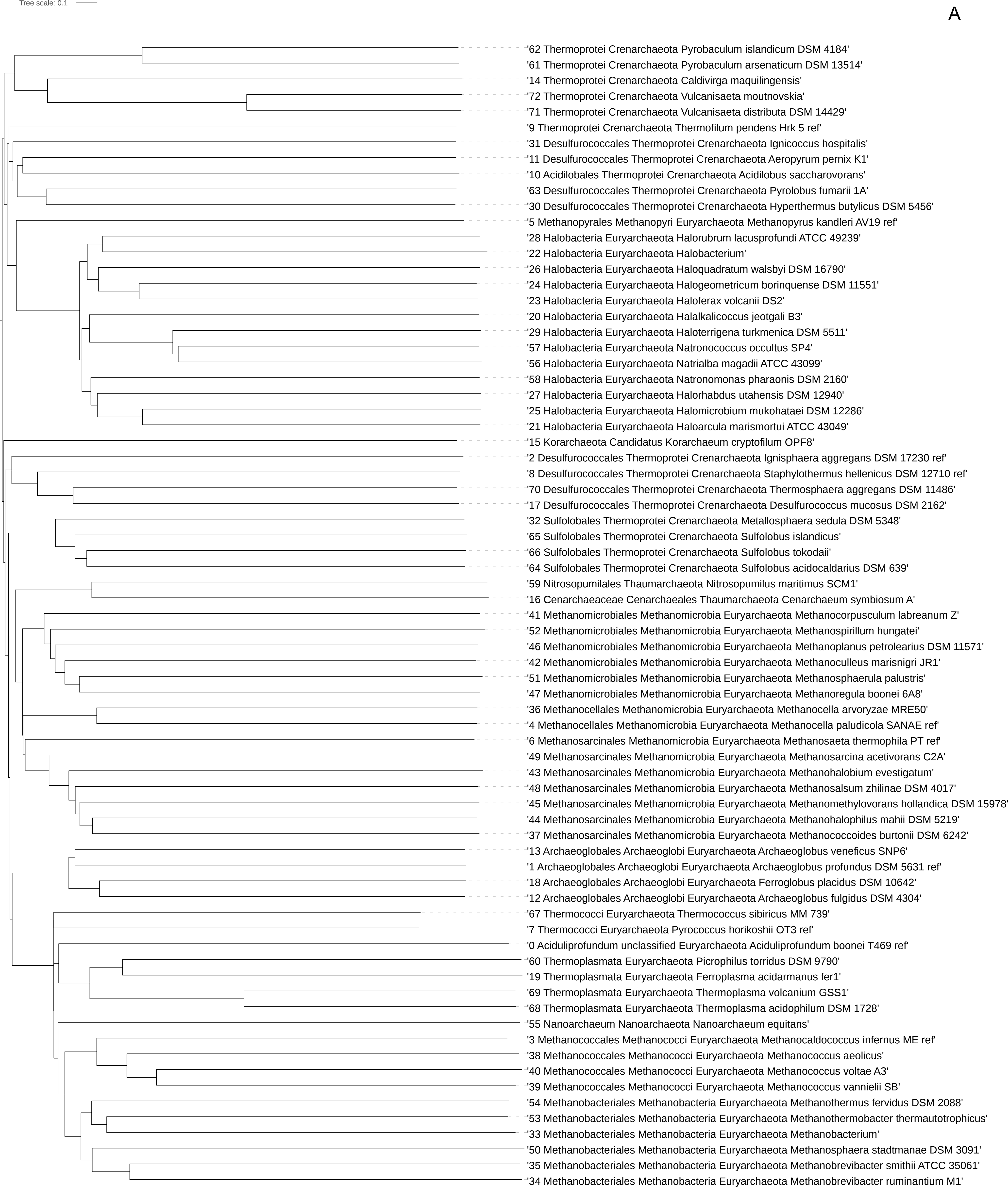

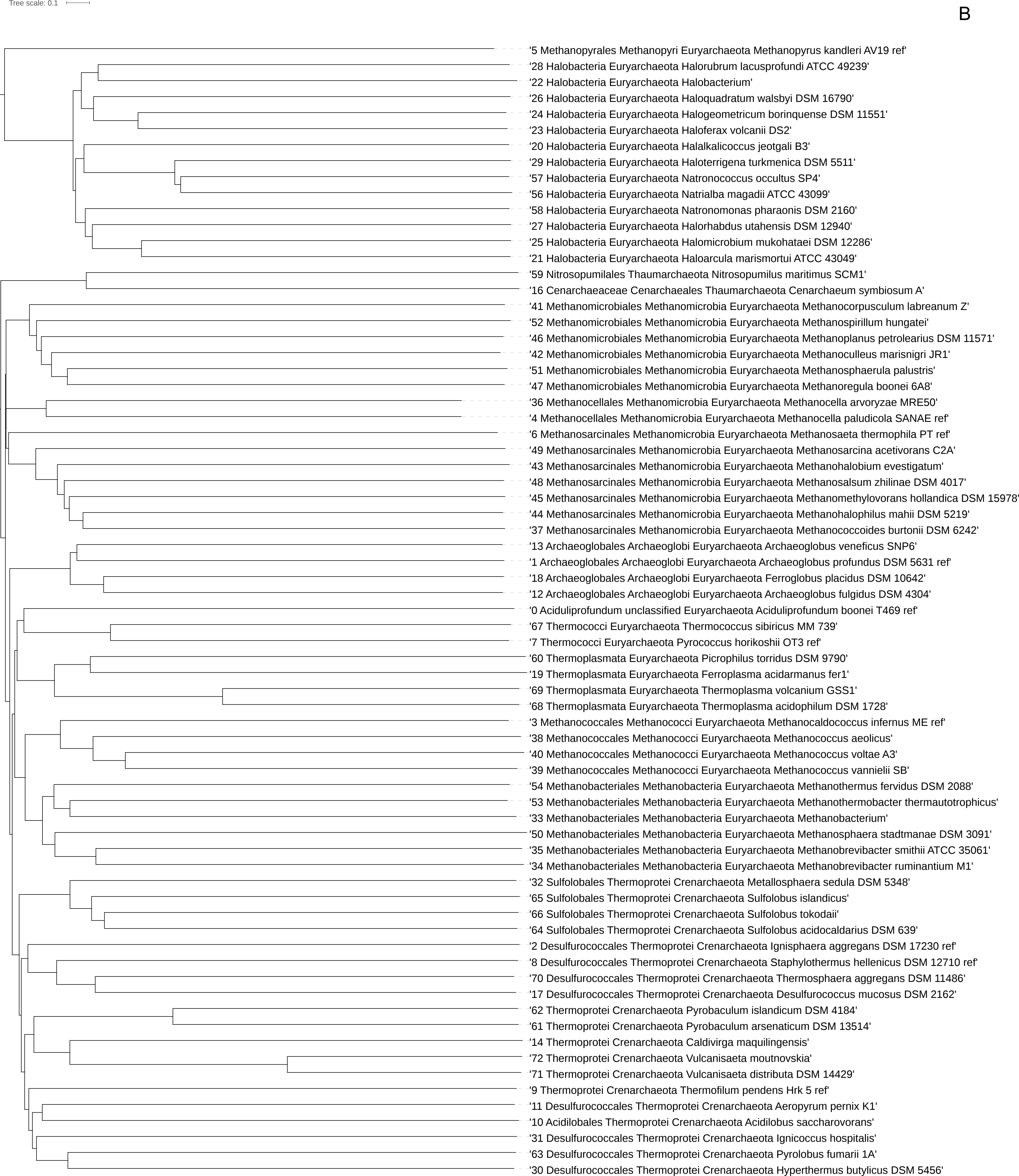

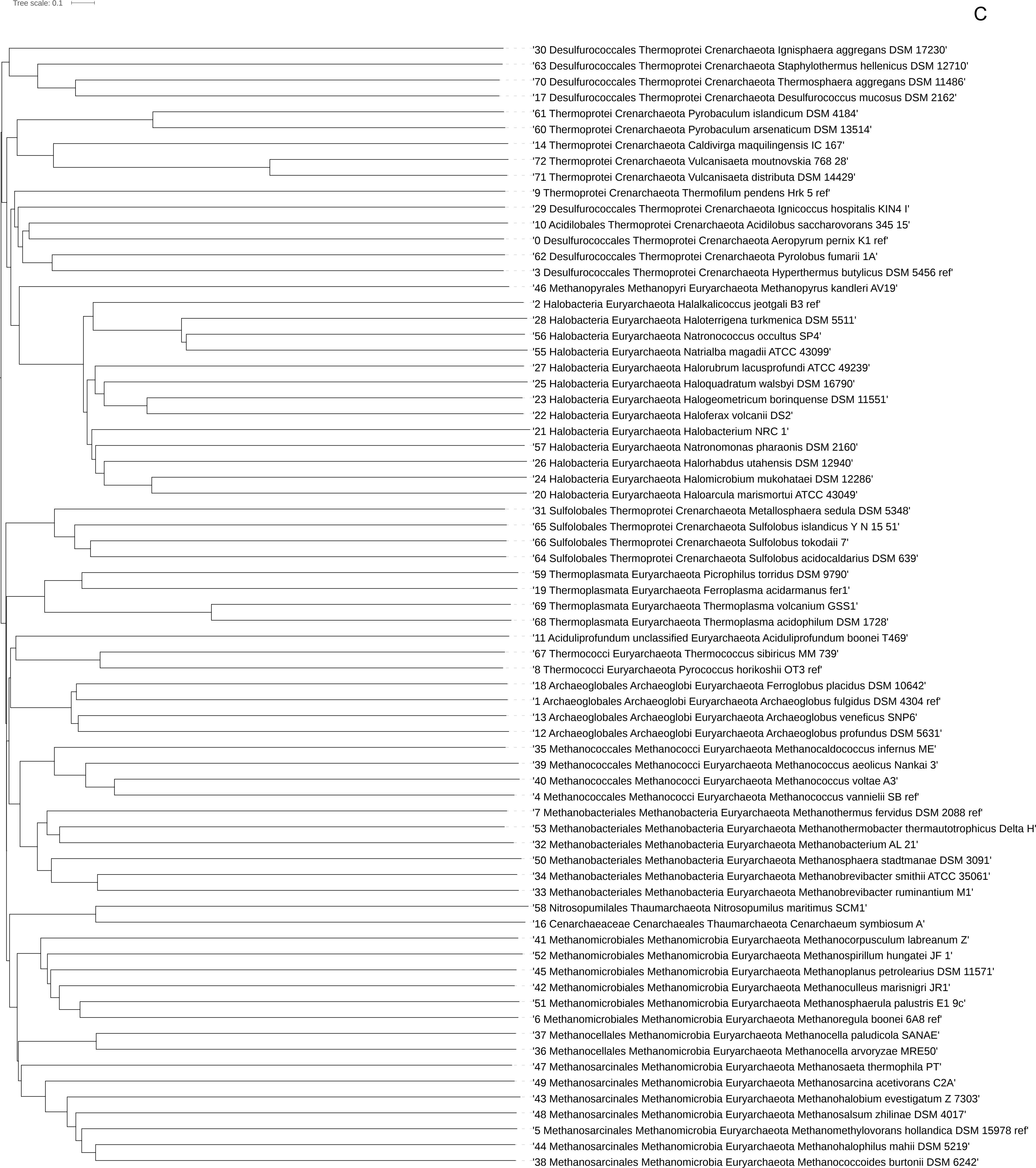

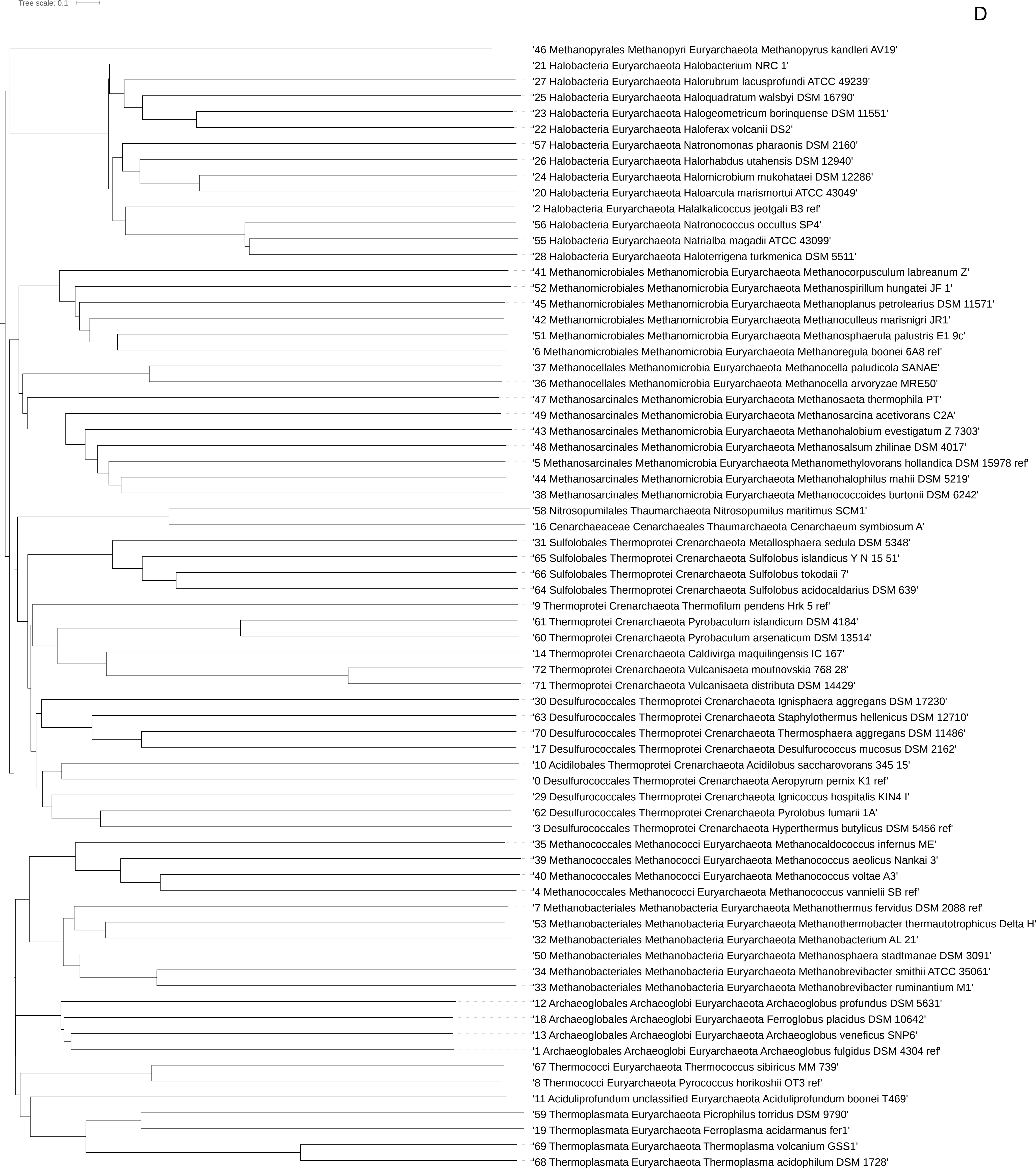

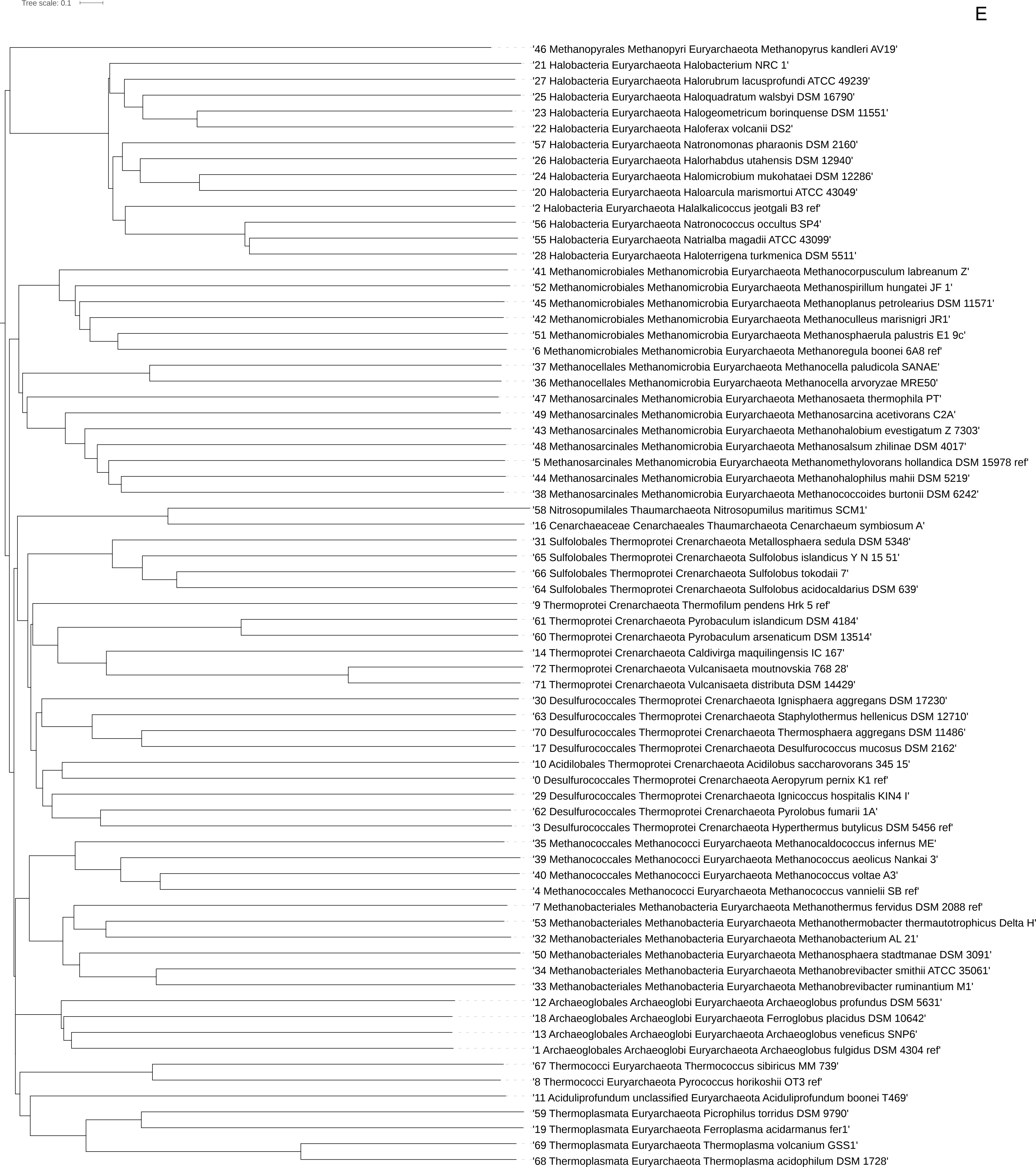

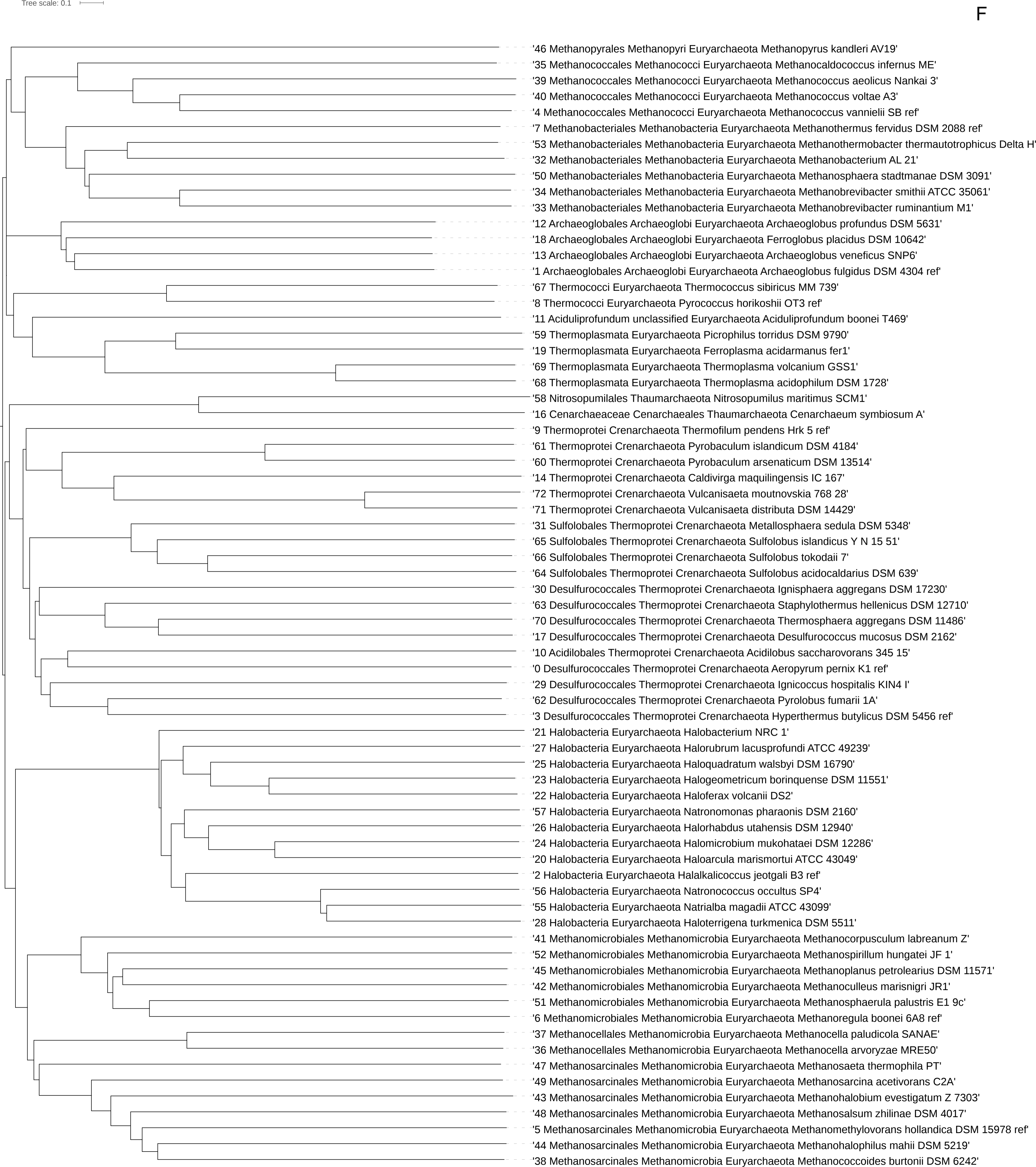

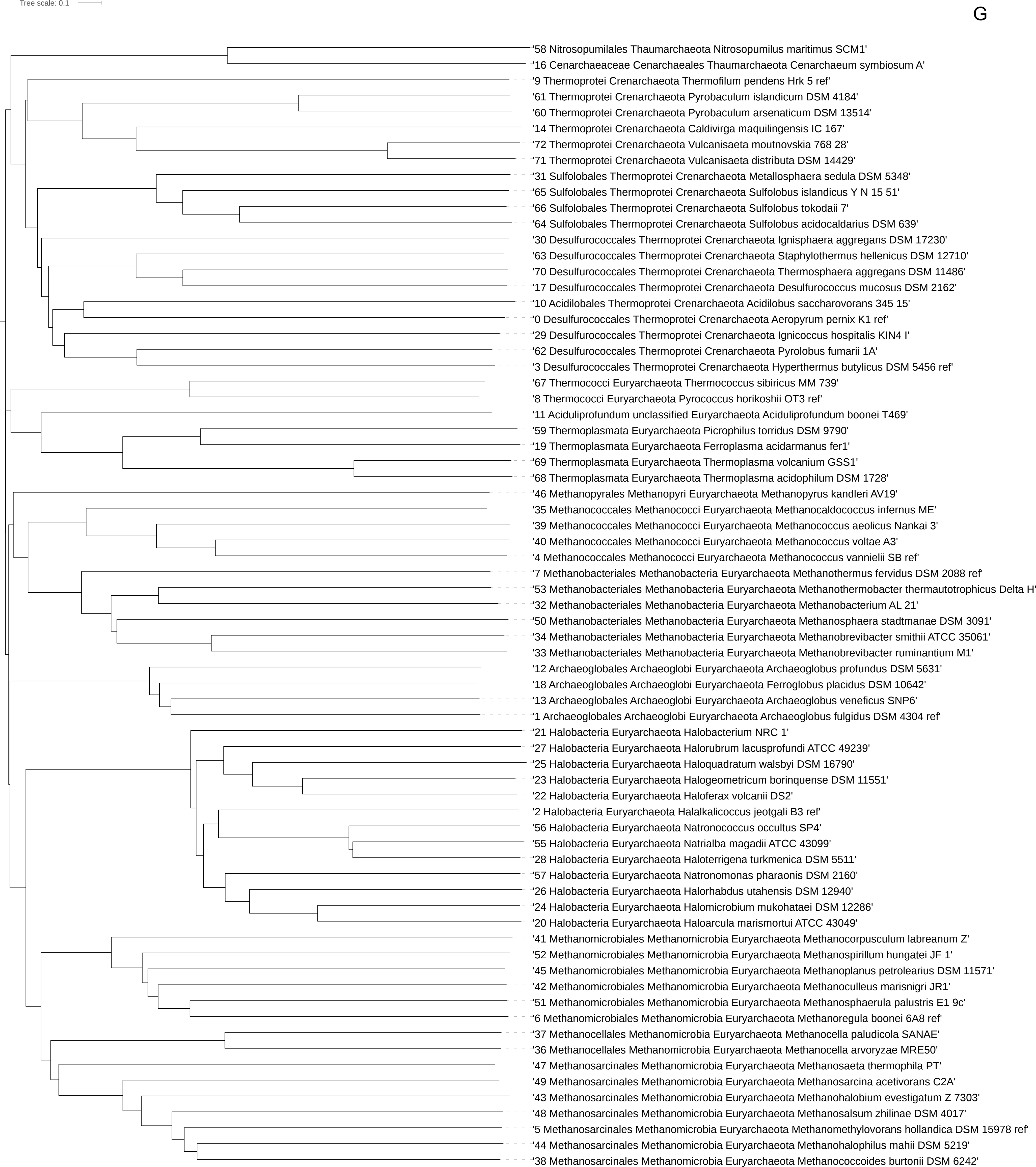

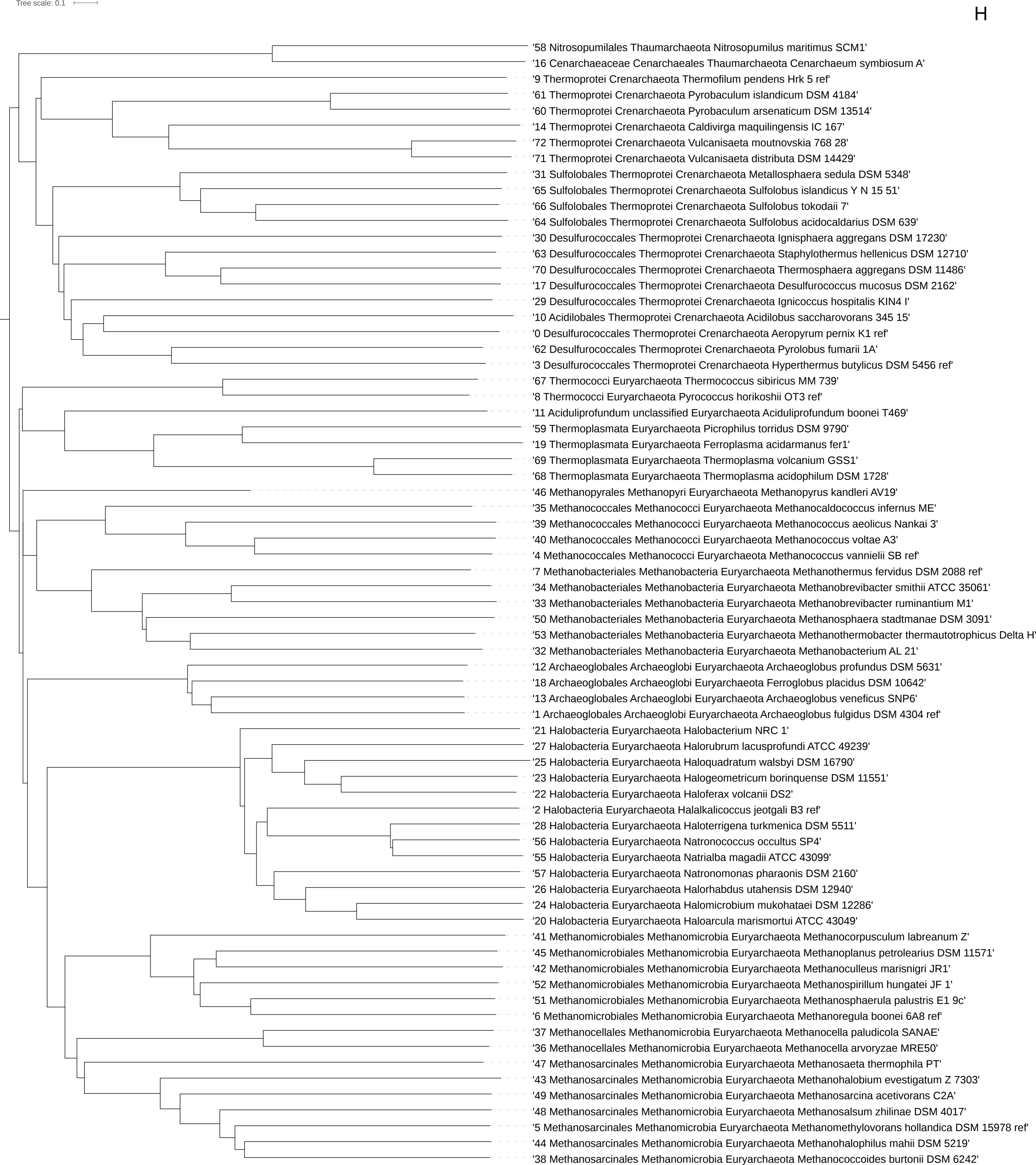

Supplement: S6 Fig — A) Raw ACS-tree for 73 archaea. Unfiltered and unpruned. B) Raw ACS-tree for 71 archaea. Unfiltered and pruned. C) ACS- tree for 71 archaea. Filtered of mobile elements and pruned. D) ACS-tree for 71 archaea. Filtered of mobile elements, pruned, and filtered by stability and conservation on o = 0. E) ACS-tree for 71 archaea. Filtered of mobile elements, pruned, and filtered by stability and conservation on o = 1. F) ACS-tree for 71 archaea. Filtered of mobile elements, pruned, and filtered by stability and conservation on o = 3. G) ACS-tree for 71 archaea. Filtered of mobile elements, pruned, and filtered by stability and conservation on o = 5. H) ACS-tree for 71 archaea. Filtered of mobile elements, pruned, and filtered by stability and conservation on o = 7. (PDF) [file pcbi.1004985.s006.pdf]

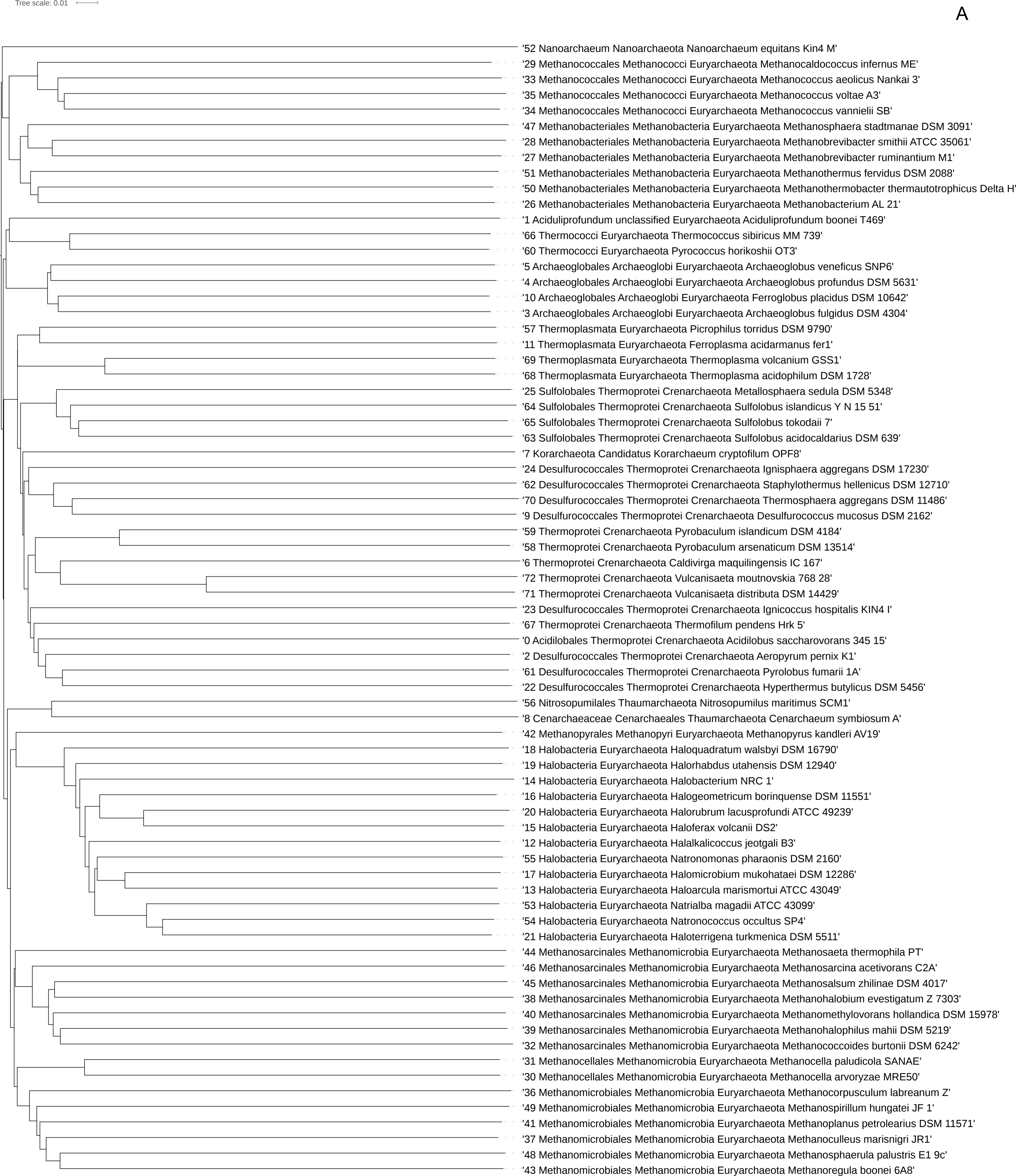

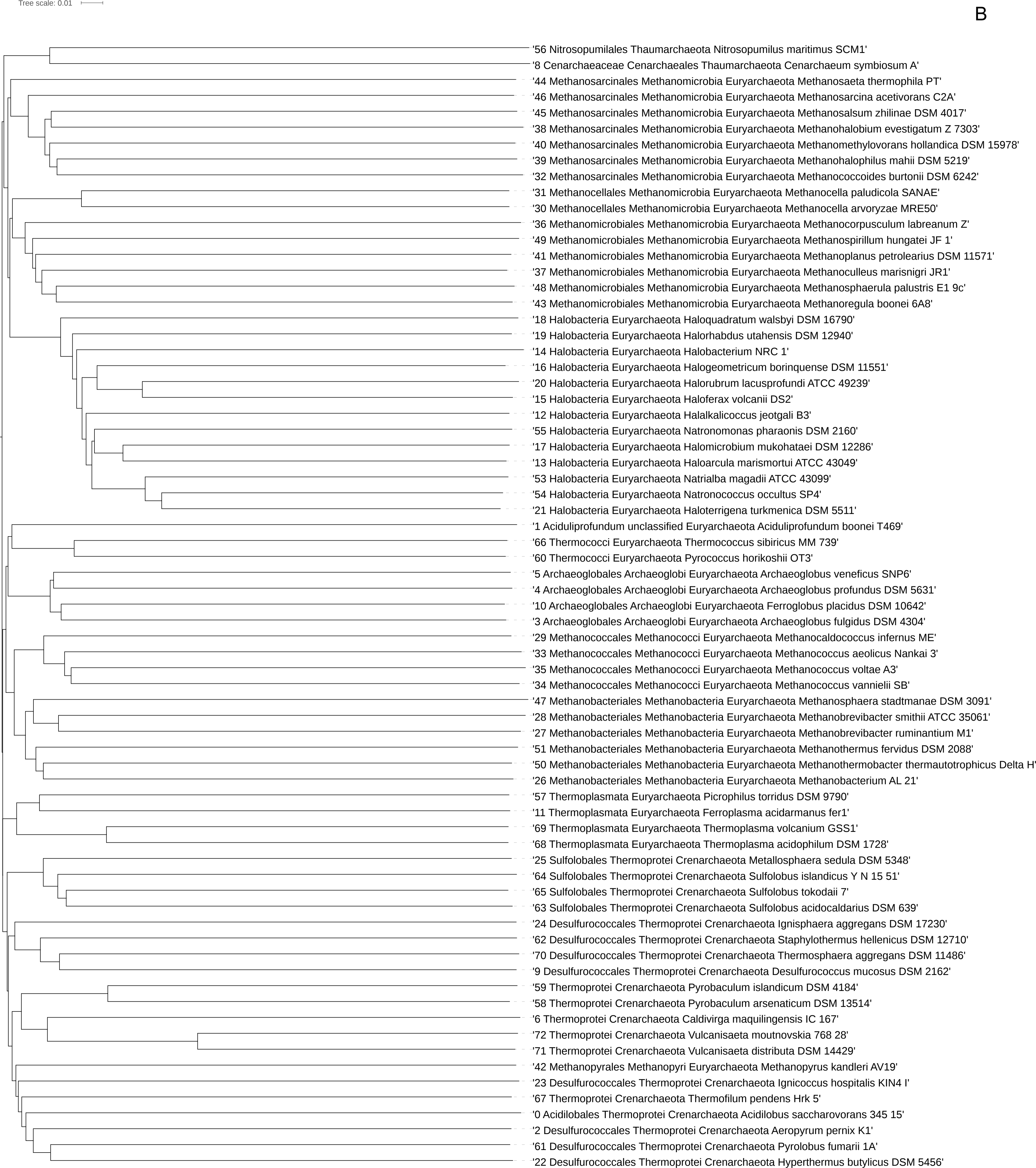

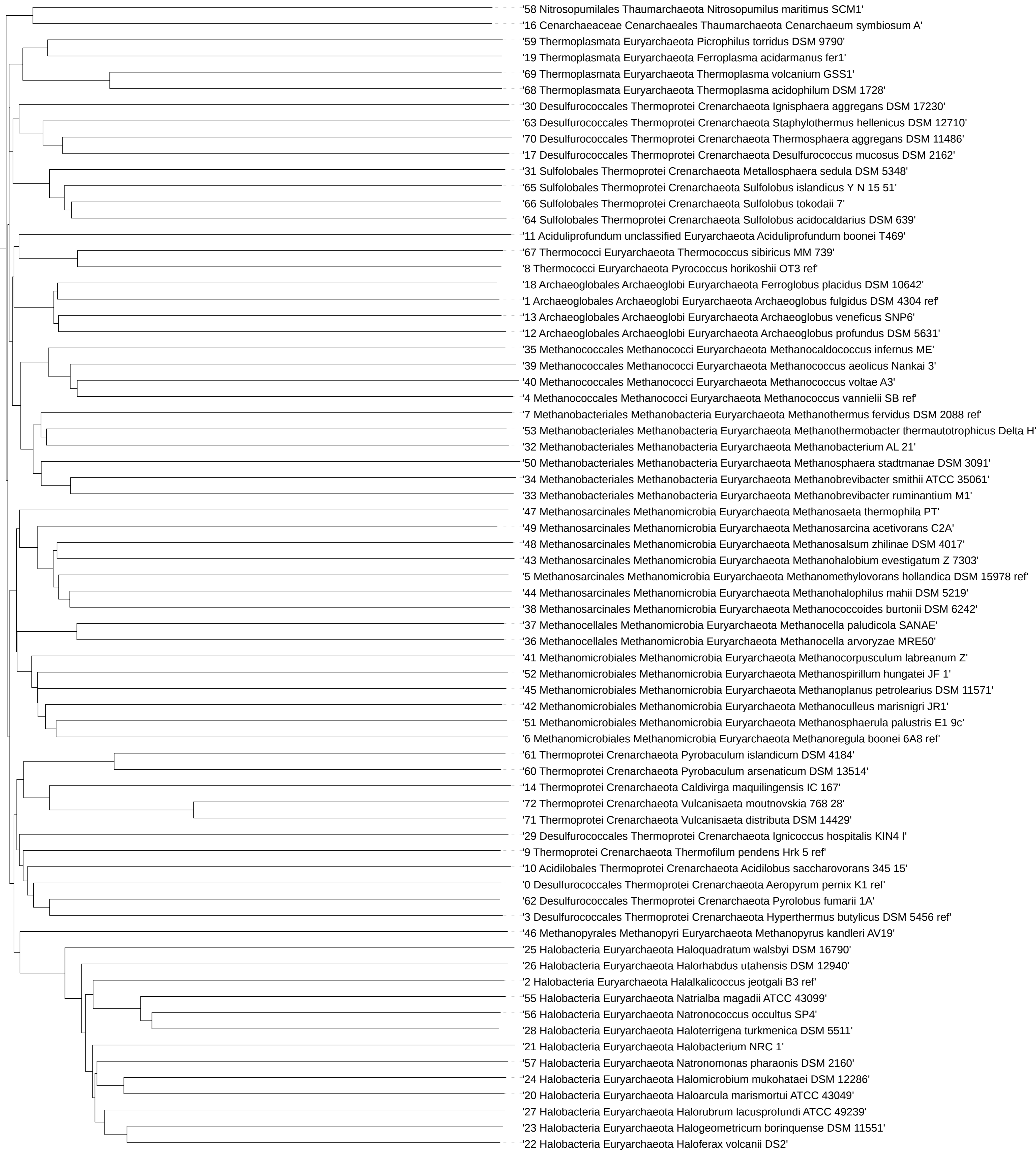

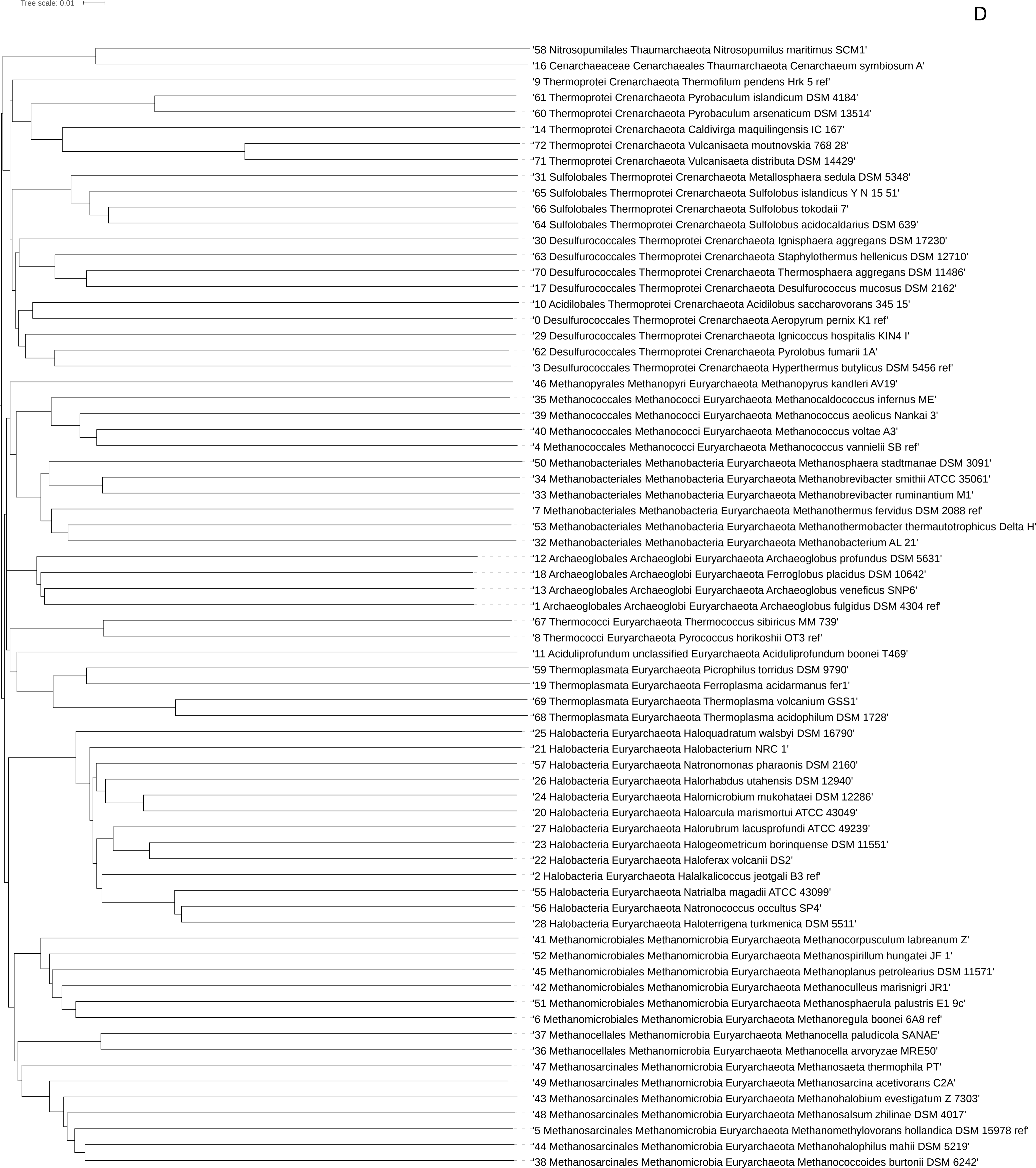

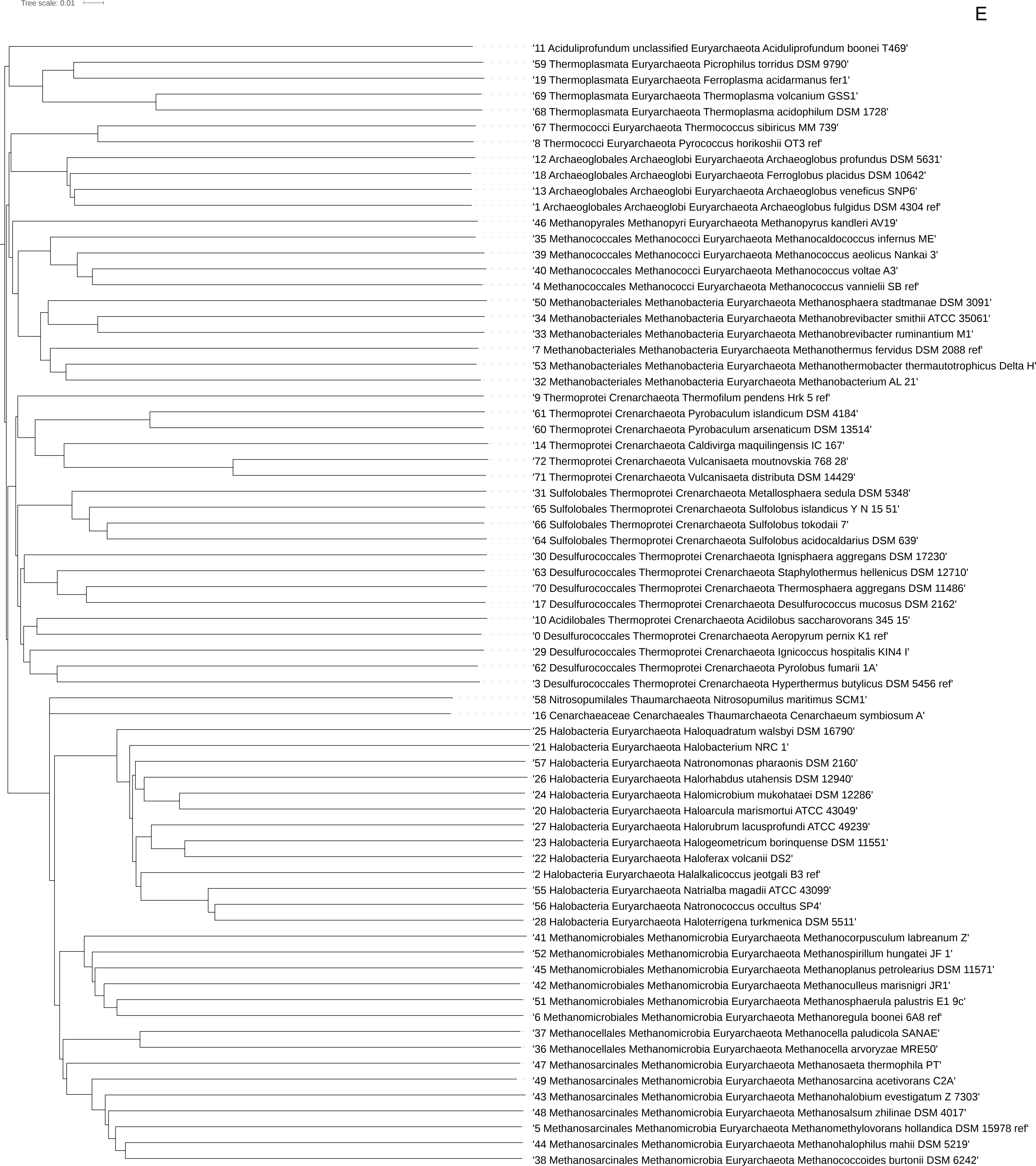

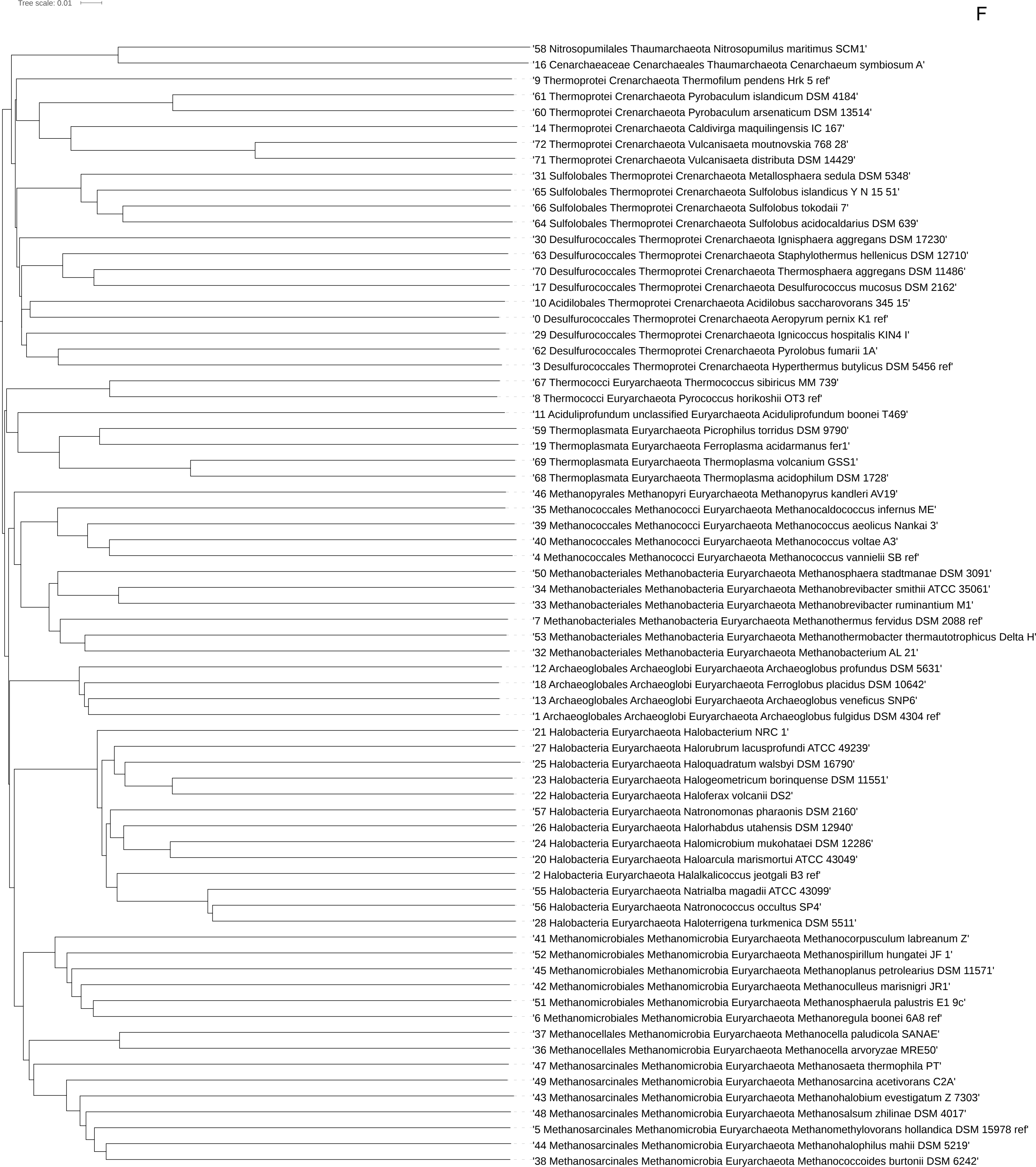

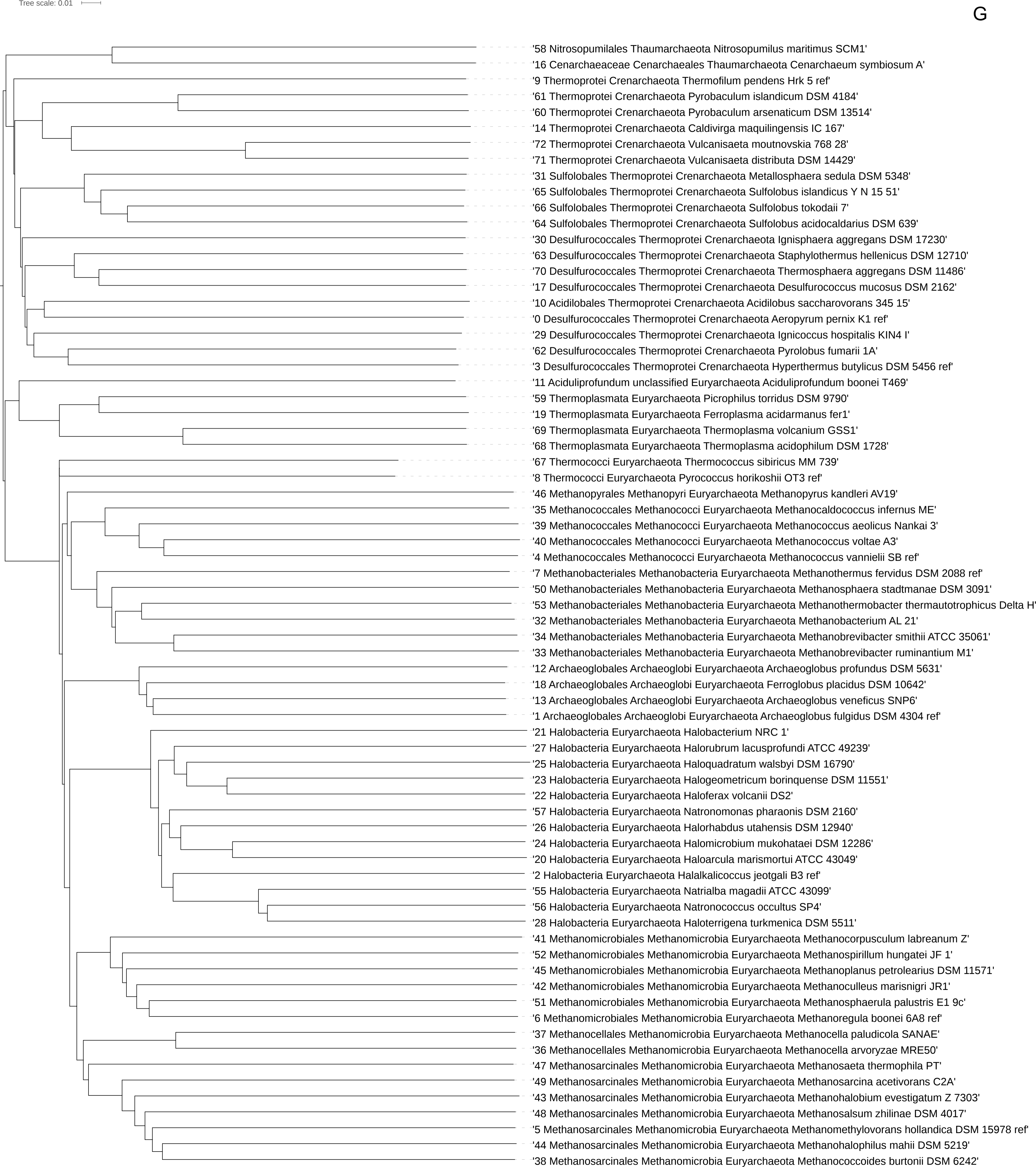

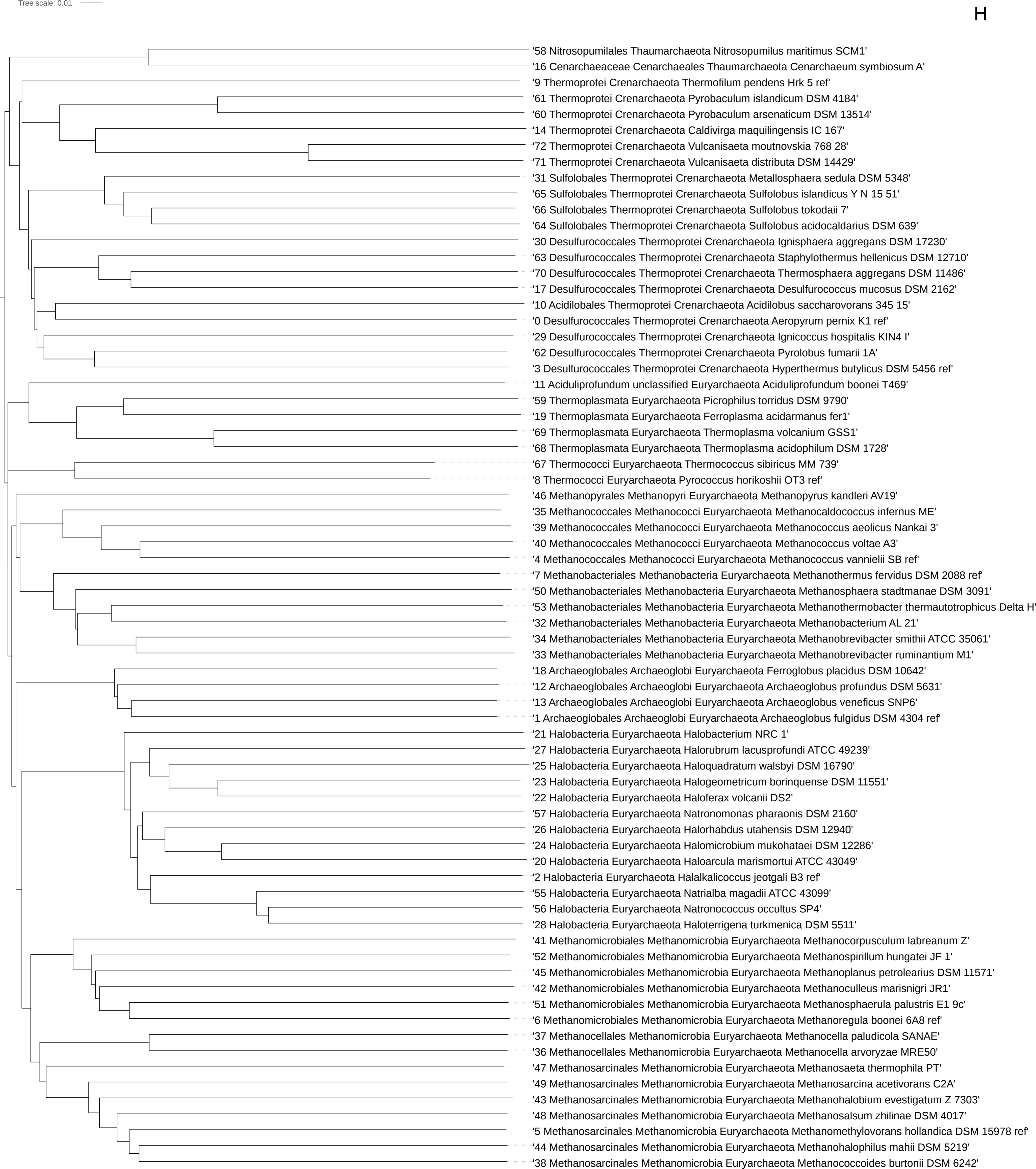

Supplement: S7 Fig — A) Raw CVTree on raw 73 archaea. Unfiltered and unpruned. B) Raw CVTree on 71 archaea. Unfiltered and pruned. C) CVTree on 71 archaea. Filtered of mobile elements and pruned. D) CVTree on 71 archaea. Filtered of mobile elements, pruned, and filtered by stability and conservation on o = 0. E) CVTree on 71 archaea. Filtered of mobile elements, pruned, and filtered by stability and conservation on o = 1. F) CVTree on 71 archaea. Filtered of mobile elements, pruned, and filtered by stability and conservation on o = 3. G) CVTree on 71 archaea. Filtered of mobile elements, pruned, and filtered by stability and conservation on o = 5. H) CVTree on 71 archaea. Filtered of mobile elements, pruned, and filtered by stability and conservation on o = 7. (PDF) [file pcbi.1004985.s007.pdf]

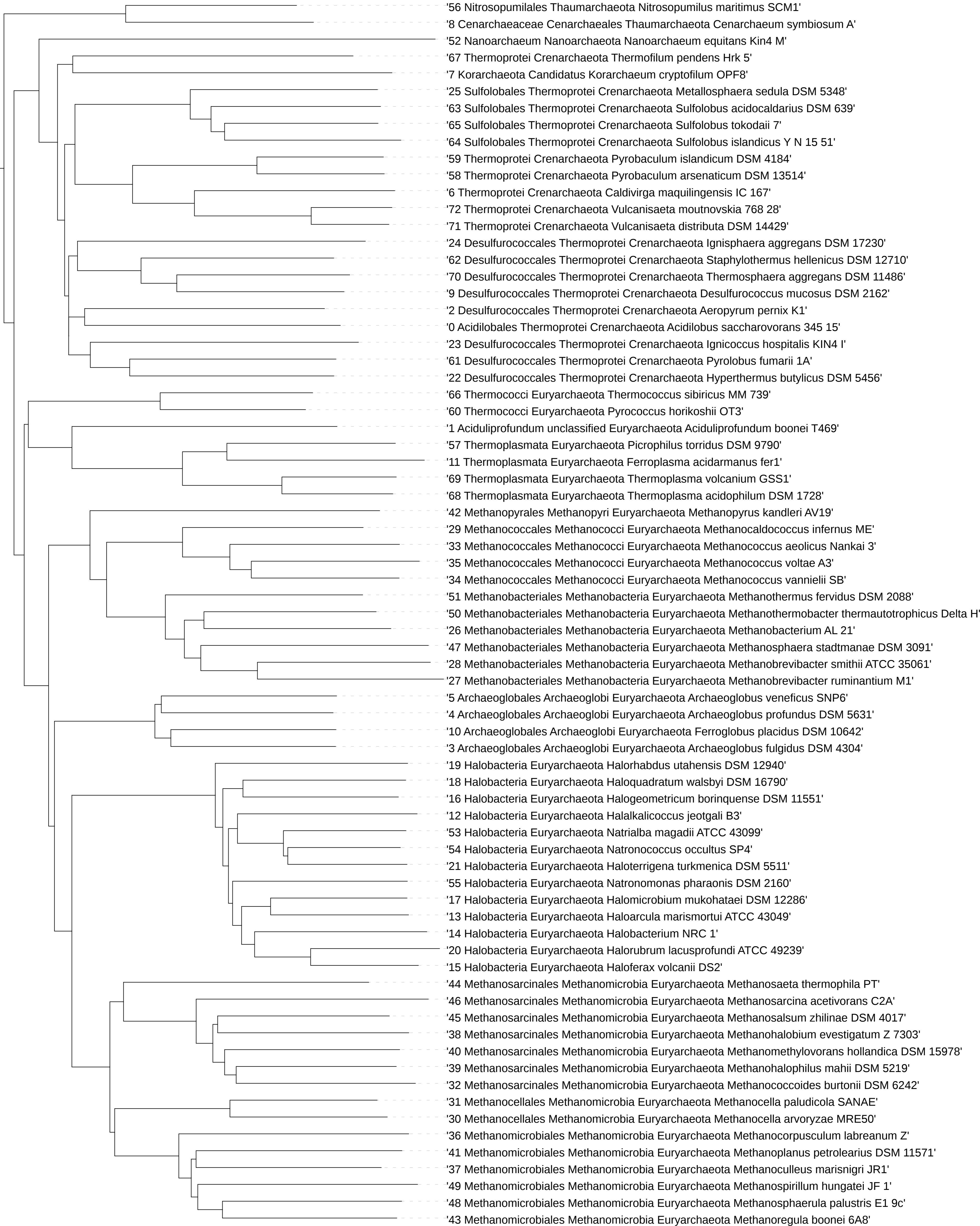

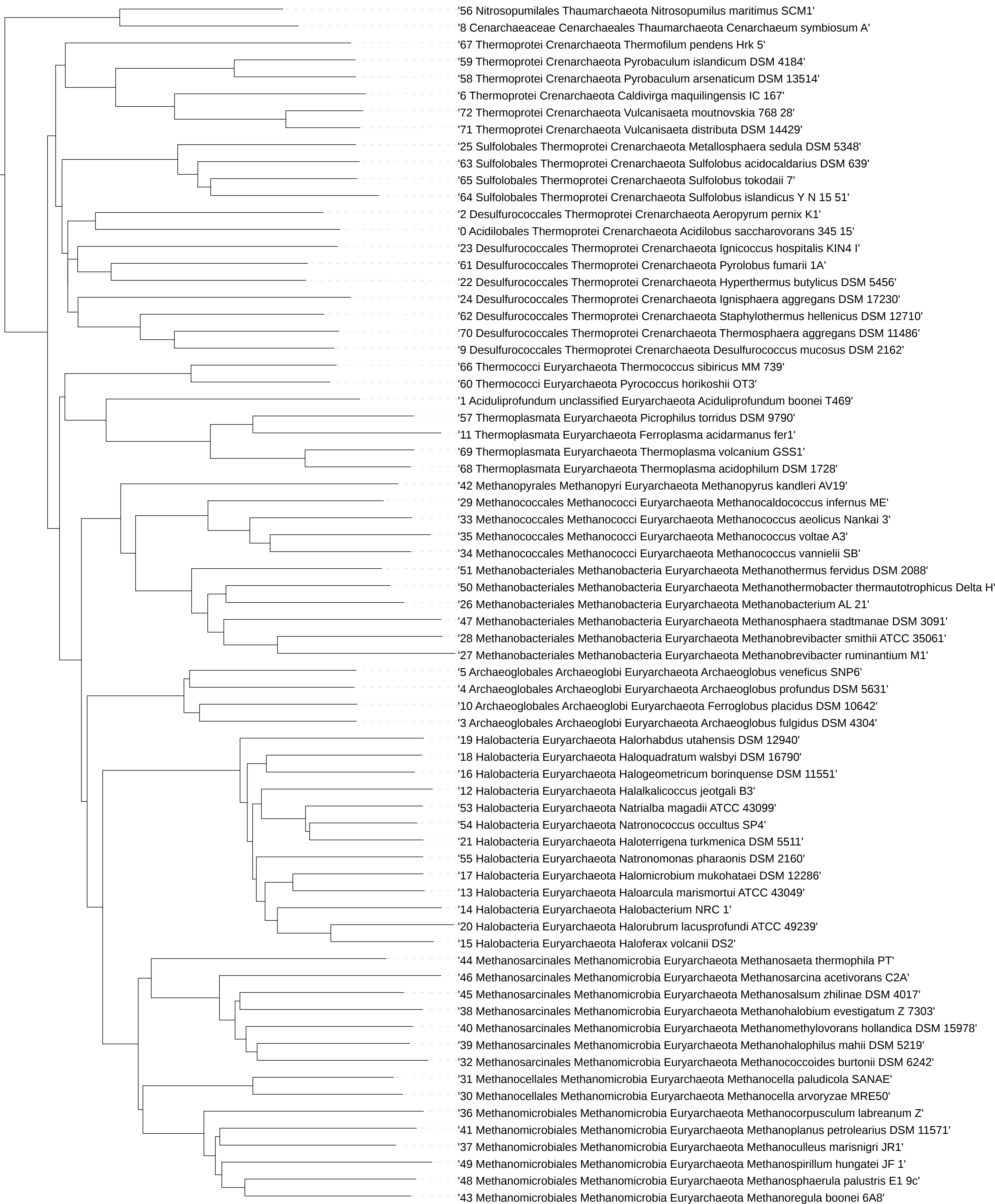

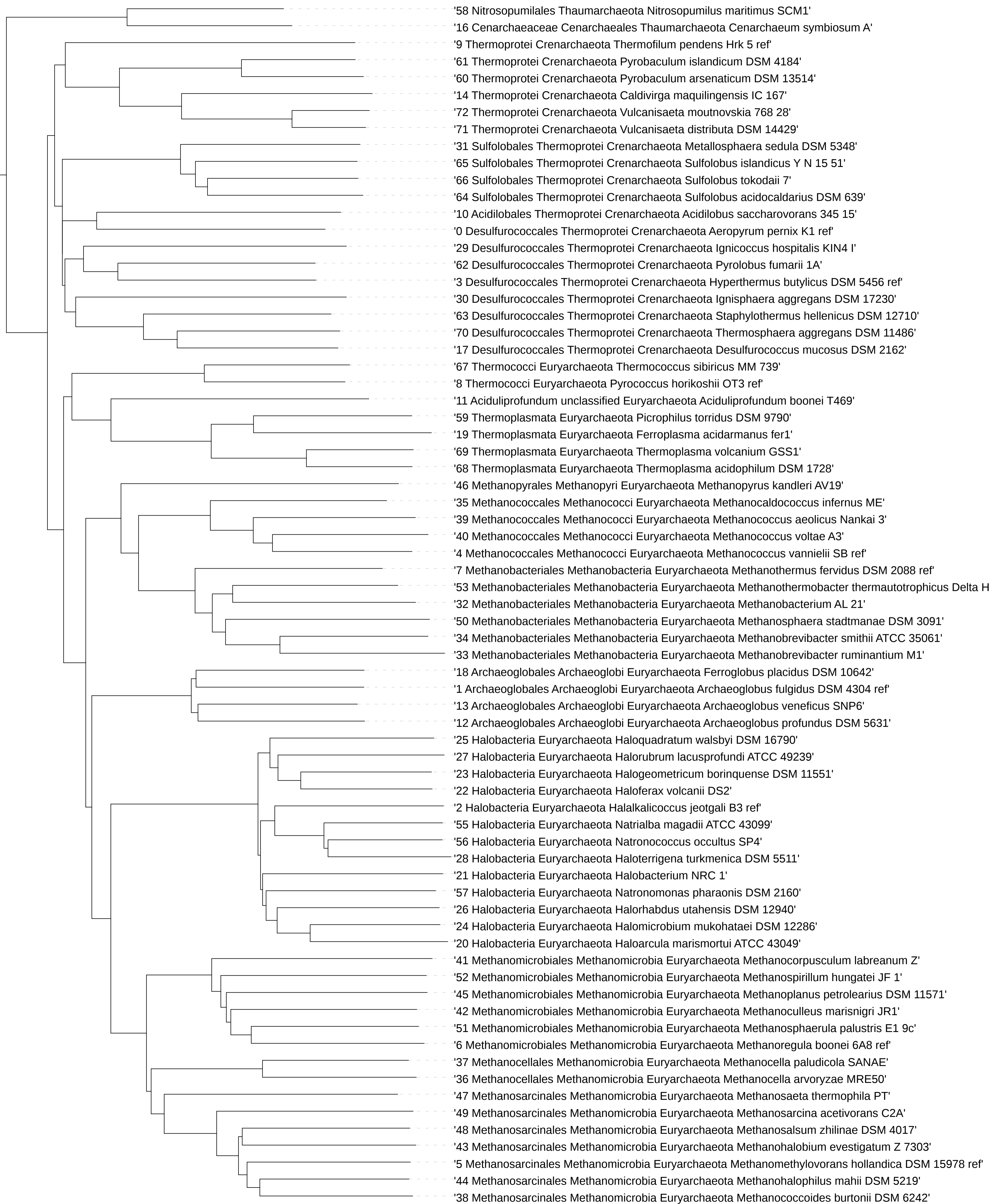

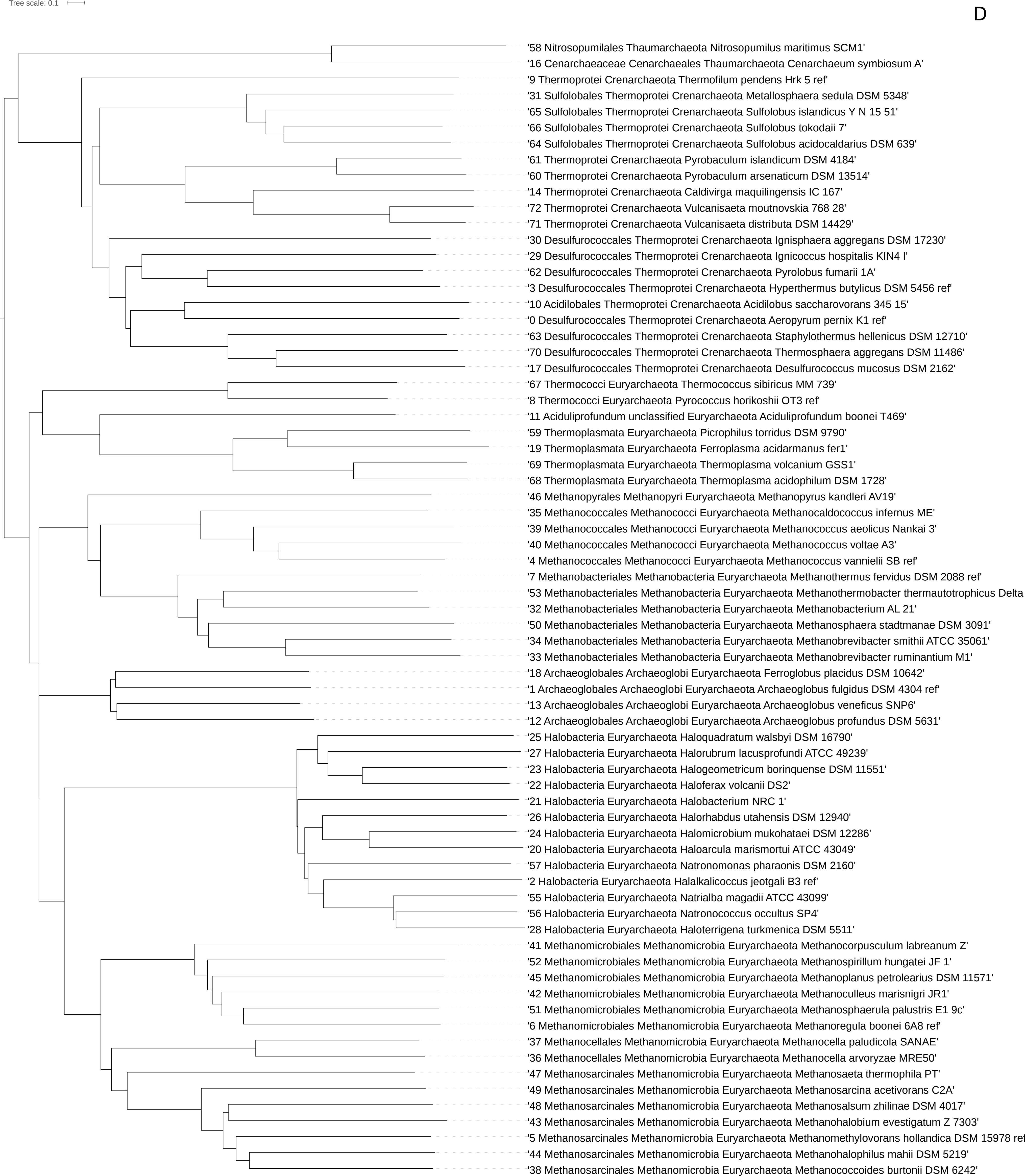

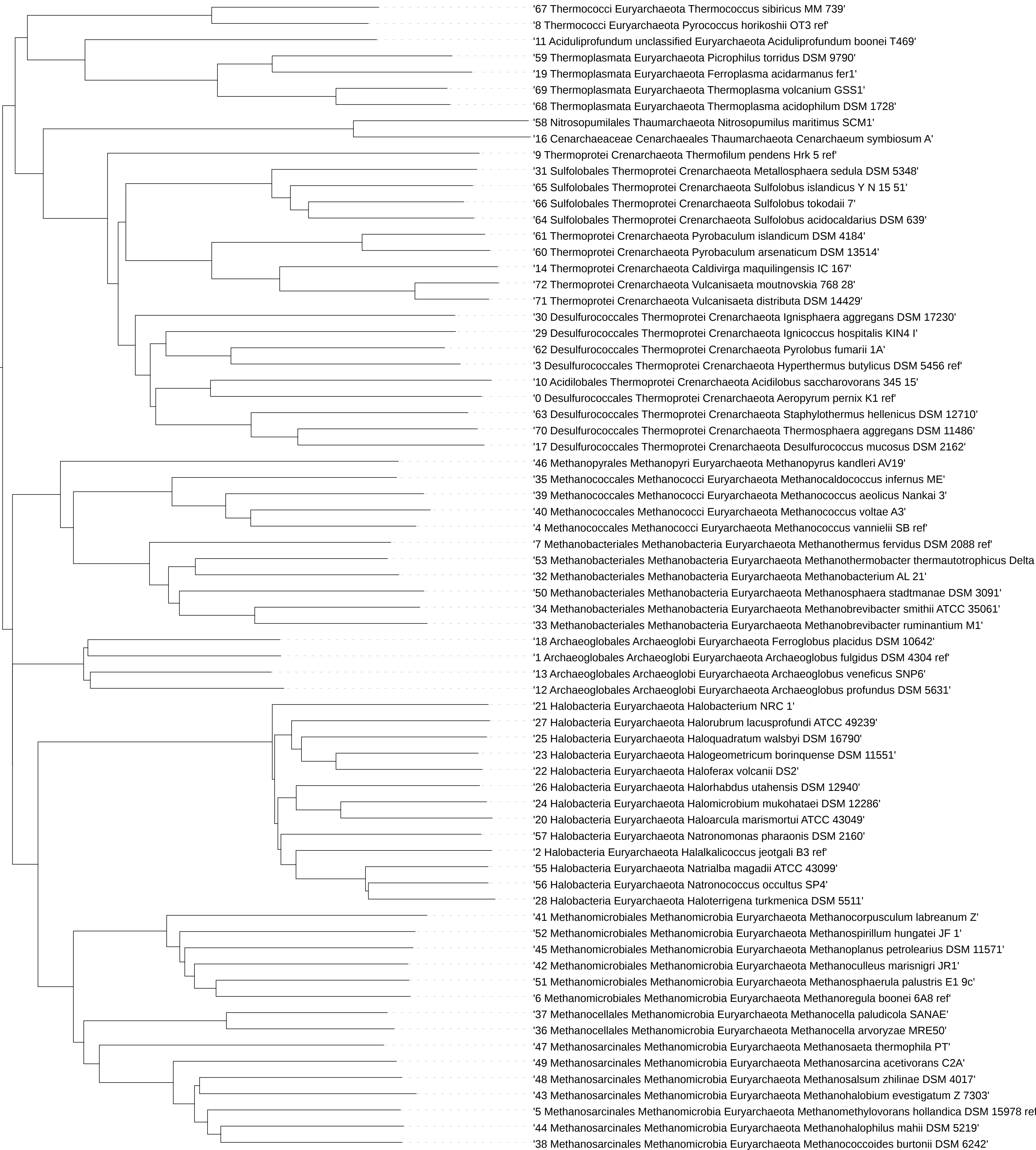

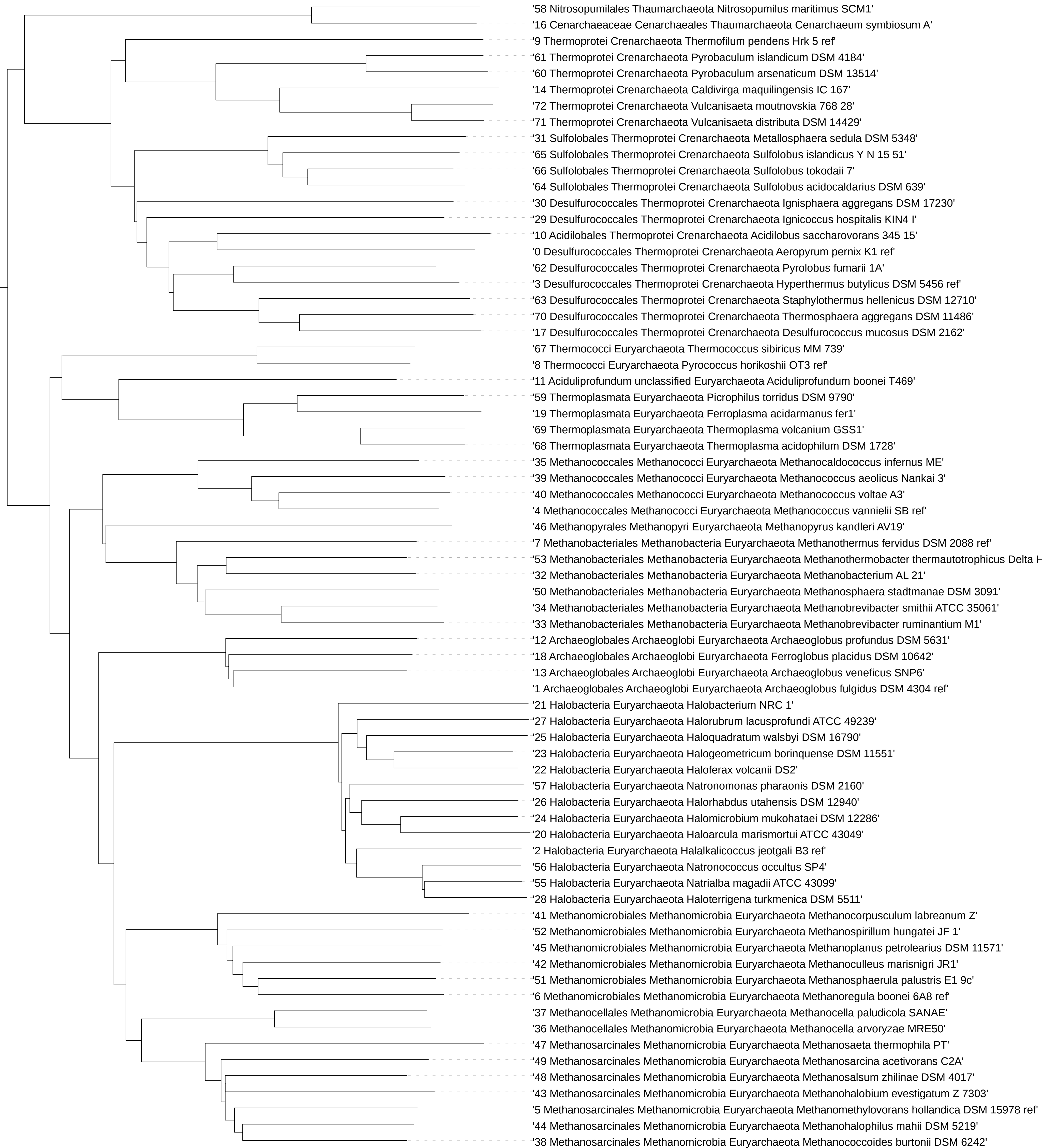

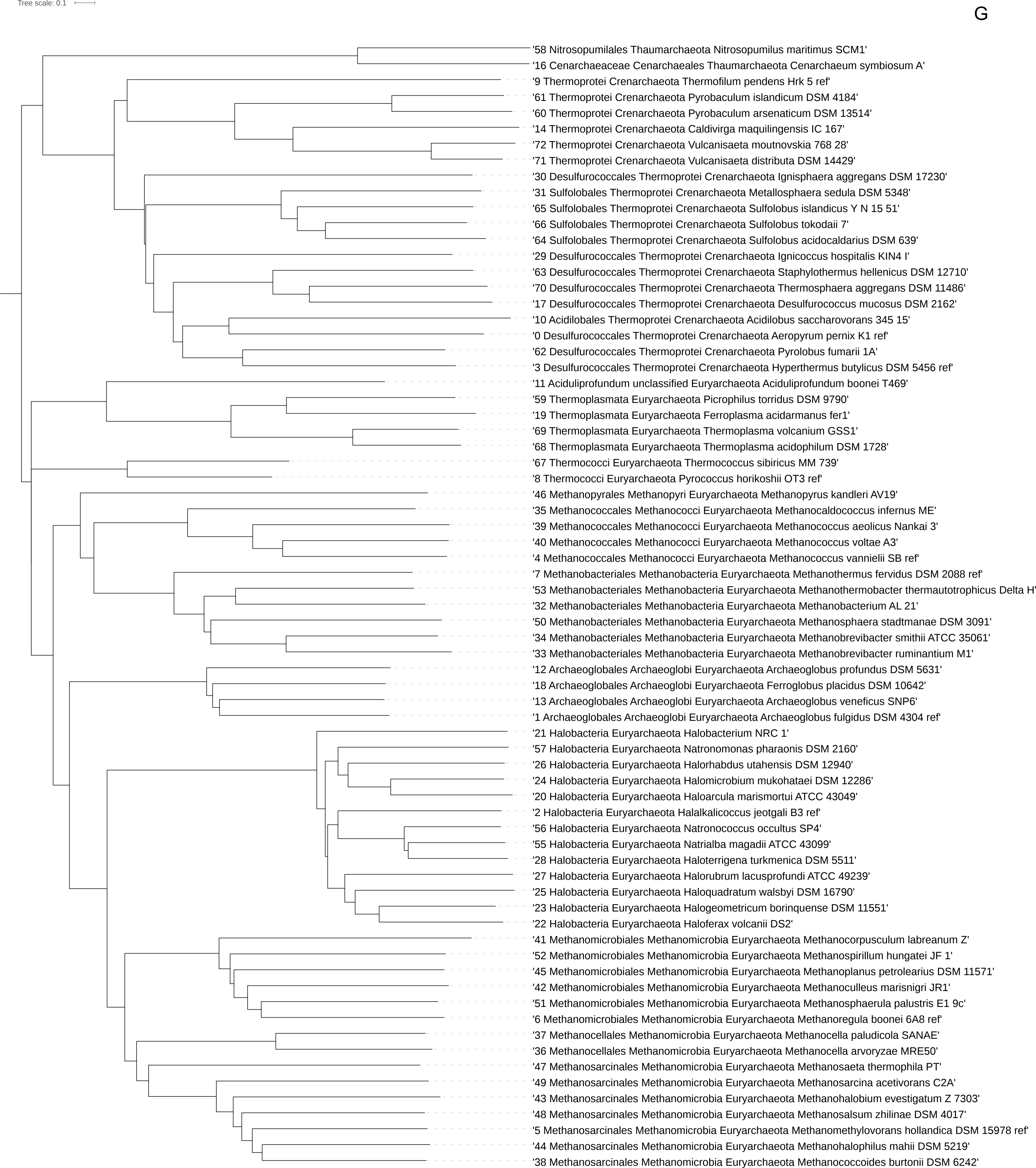

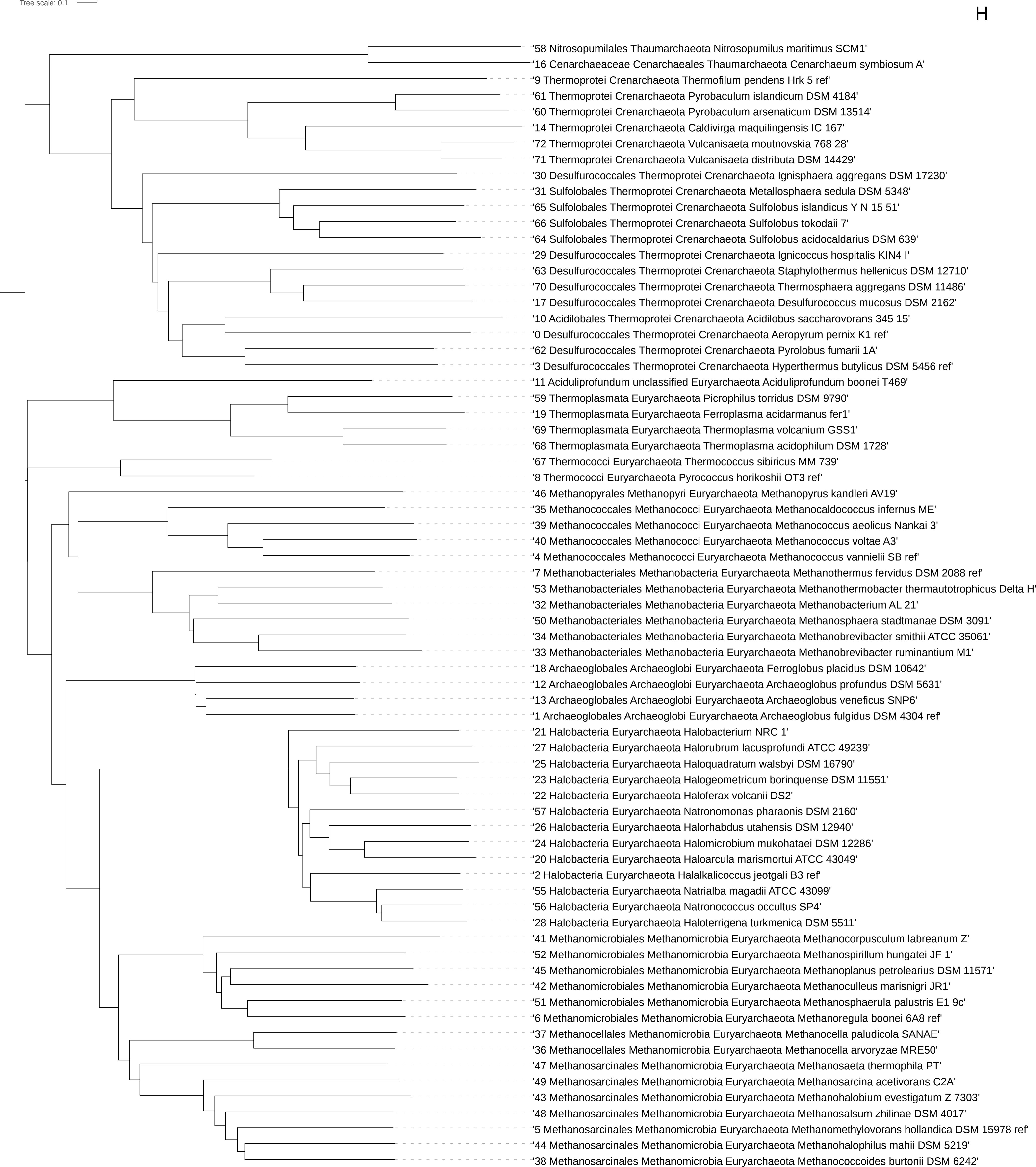

Supplement: S8 Fig — A) D2 on raw 73 archaea. Unfiltered and unpruned. B) D2 on raw 71 archaea. Unfiltered and pruned. C) D2 on 71 archaea. Filtered of mobile elements and pruned. D) D2 on 71 archaea. Filtered of mobile elements, pruned, and filtered by stability and conservation on o = 0. E) D2 on 71 archaea. Filtered of mobile elements, pruned, and filtered by stability and conservation on o = 1. F) D2 on 71 archaea. Filtered of mobile elements, pruned, and filtered by stability and conservation on o = 3. G) D2 on 71 archaea. Filtered of mobile elements, pruned, and filtered by stability and conservation on o = 5. H) D2 on 71 archaea. Filtered of mobile elements, pruned, and filtered by stability and conservation on o = 7. (PDF) [file pcbi.1004985.s008.pdf]

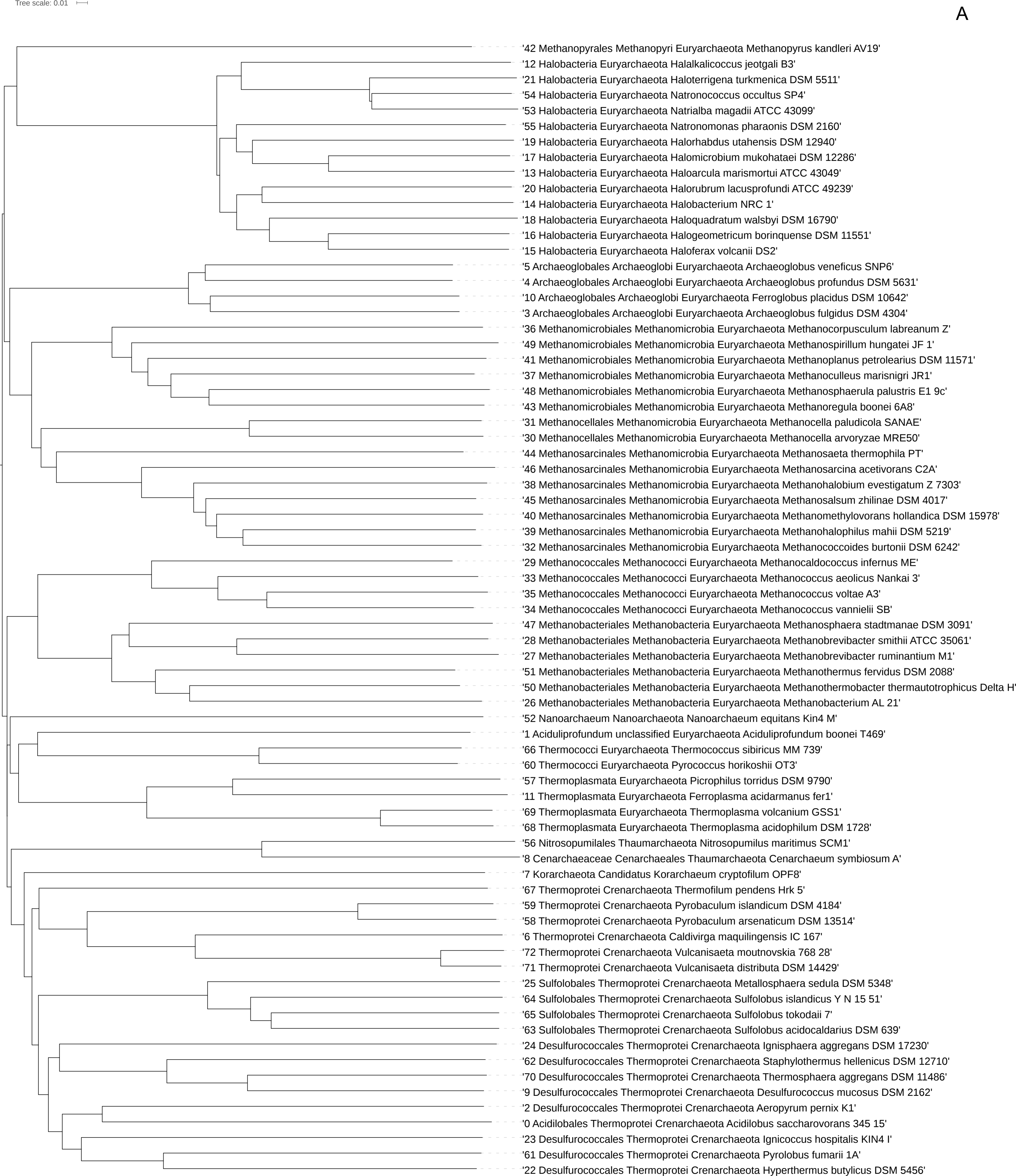

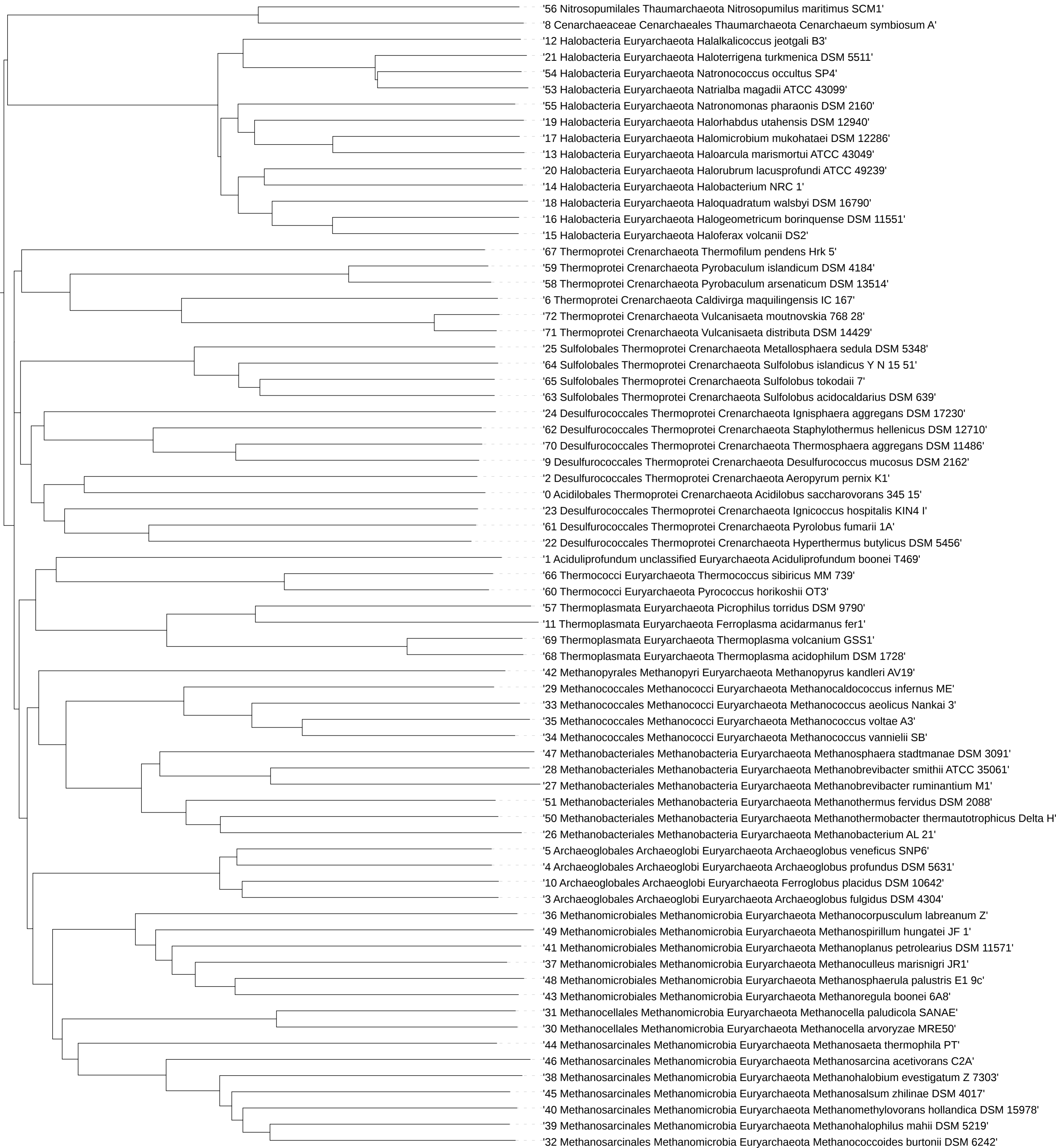

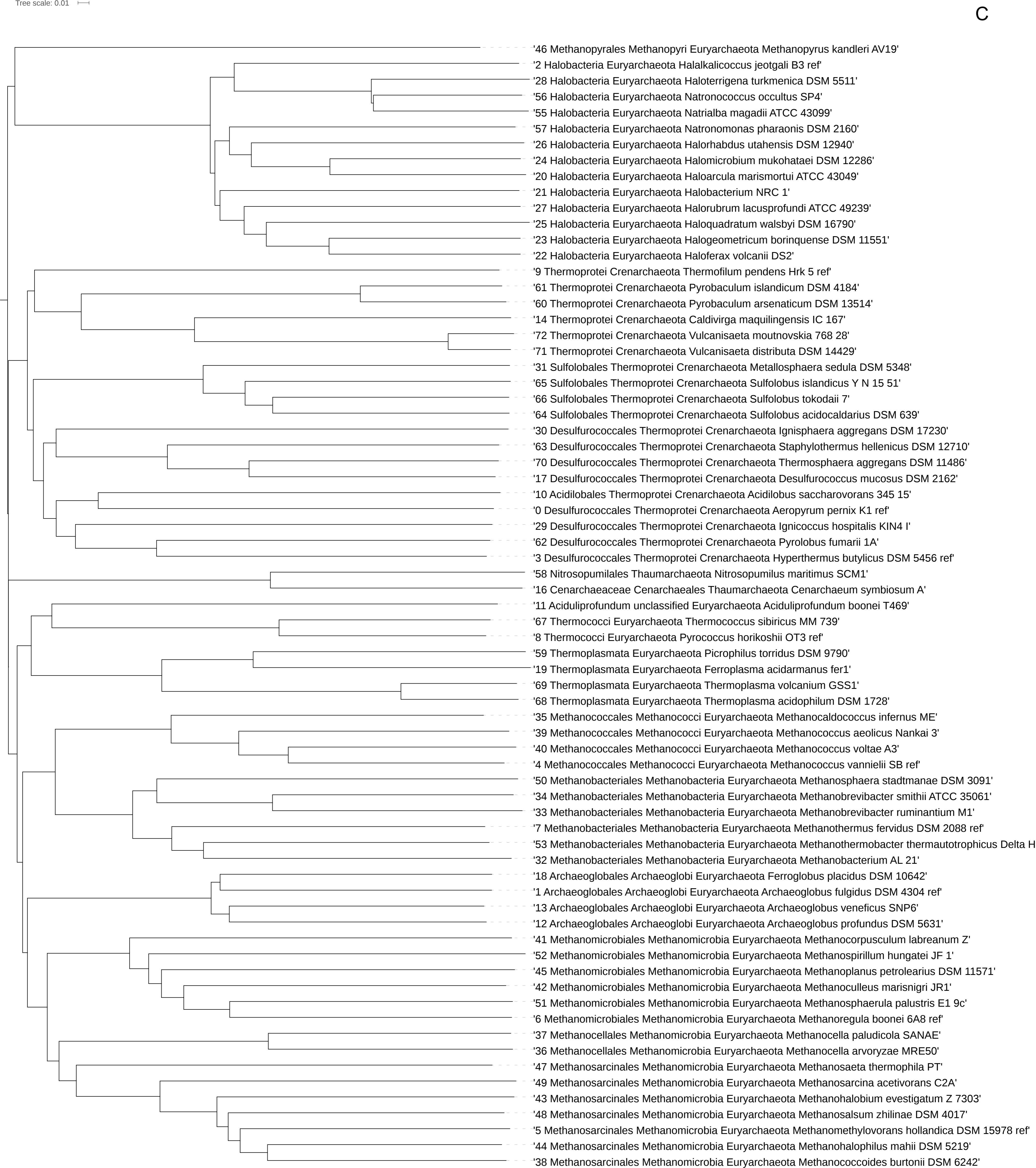

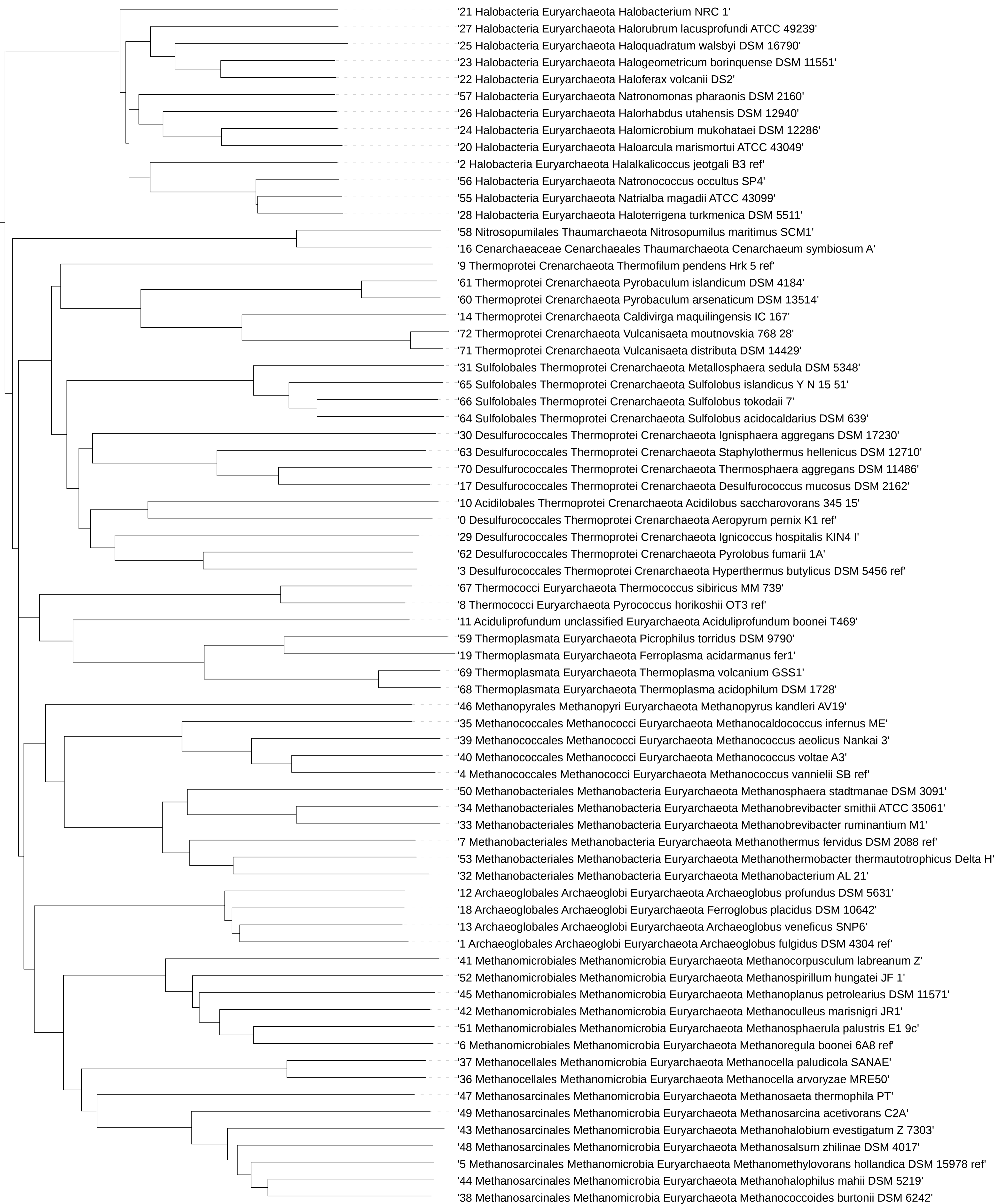

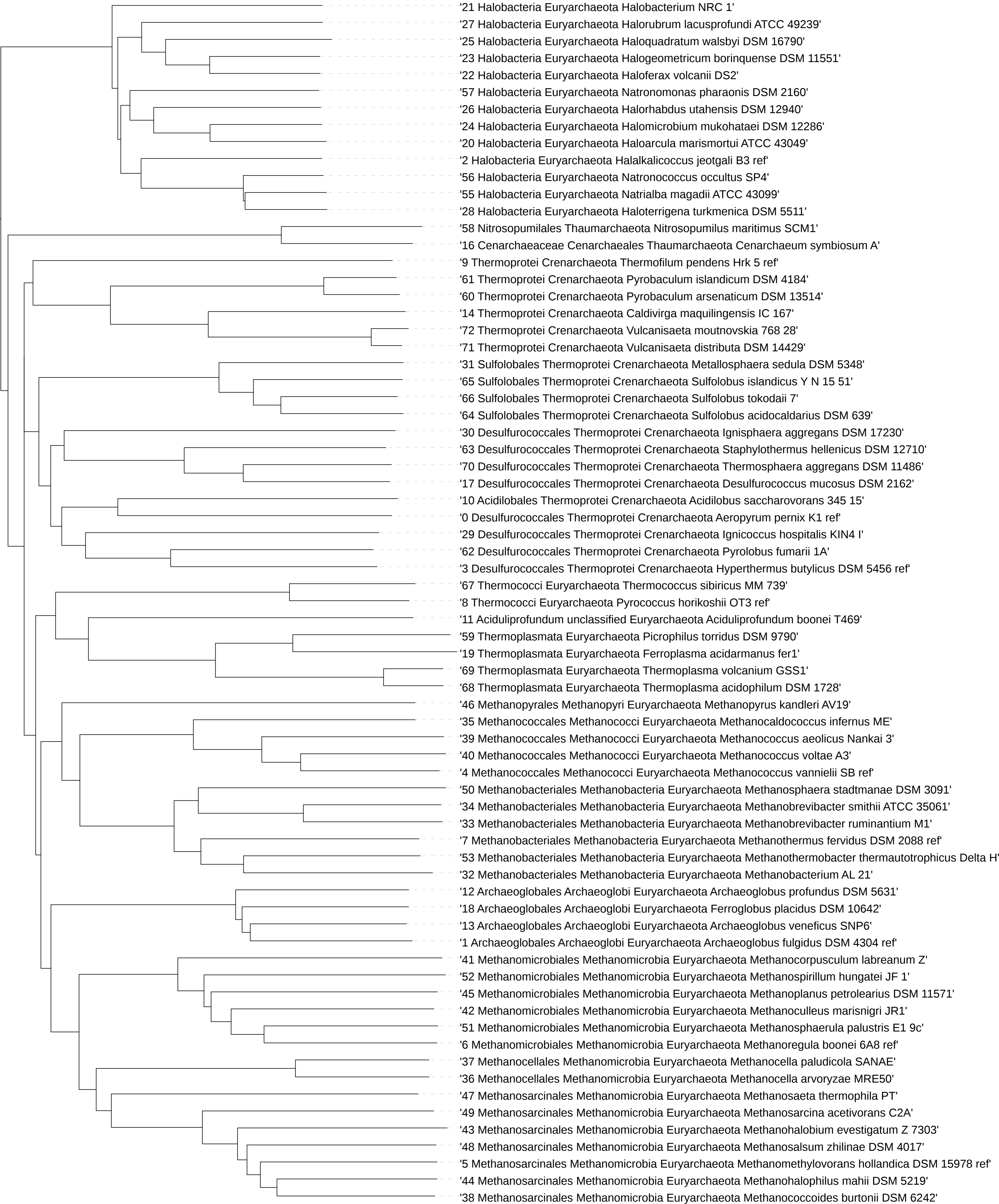

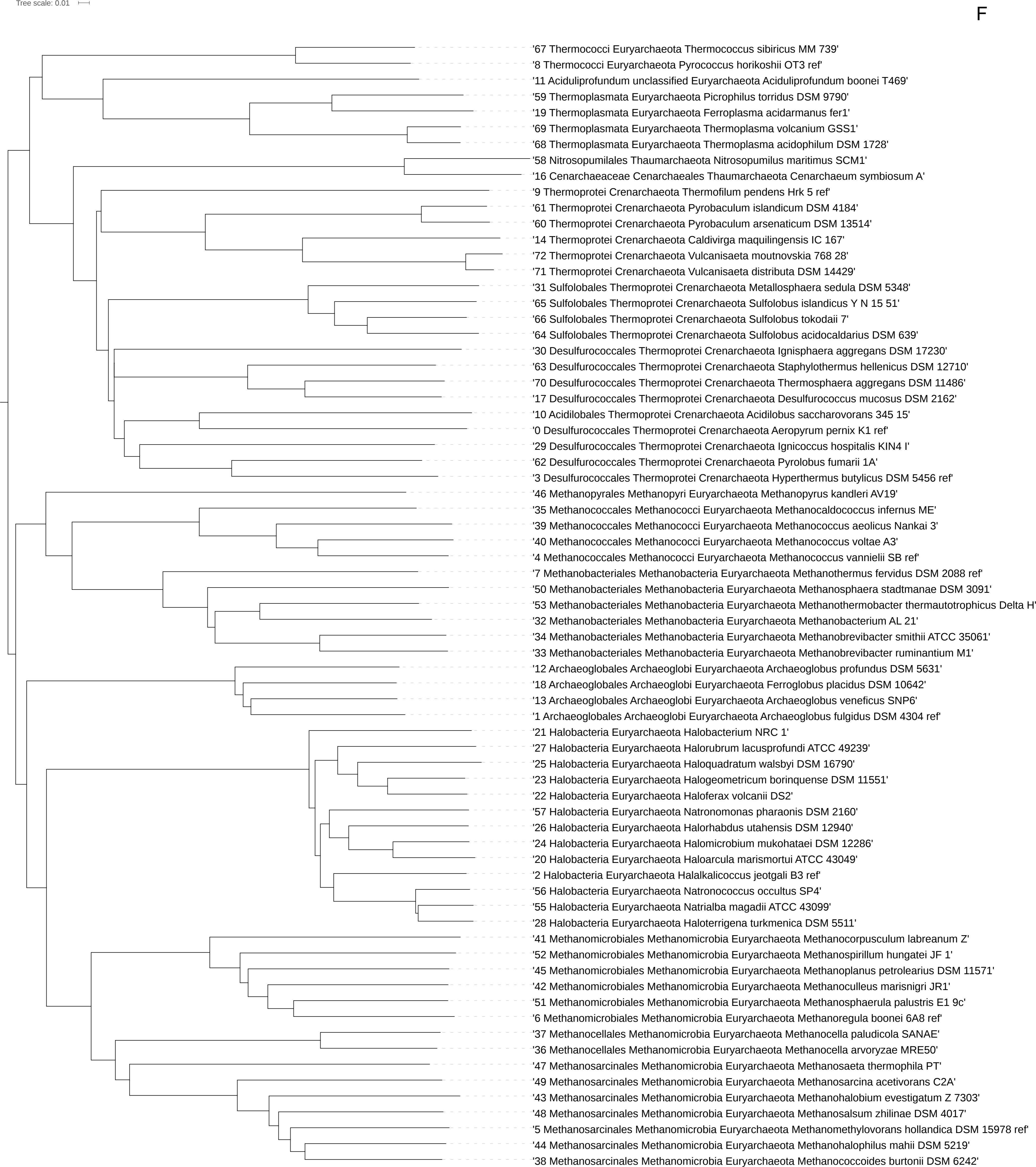

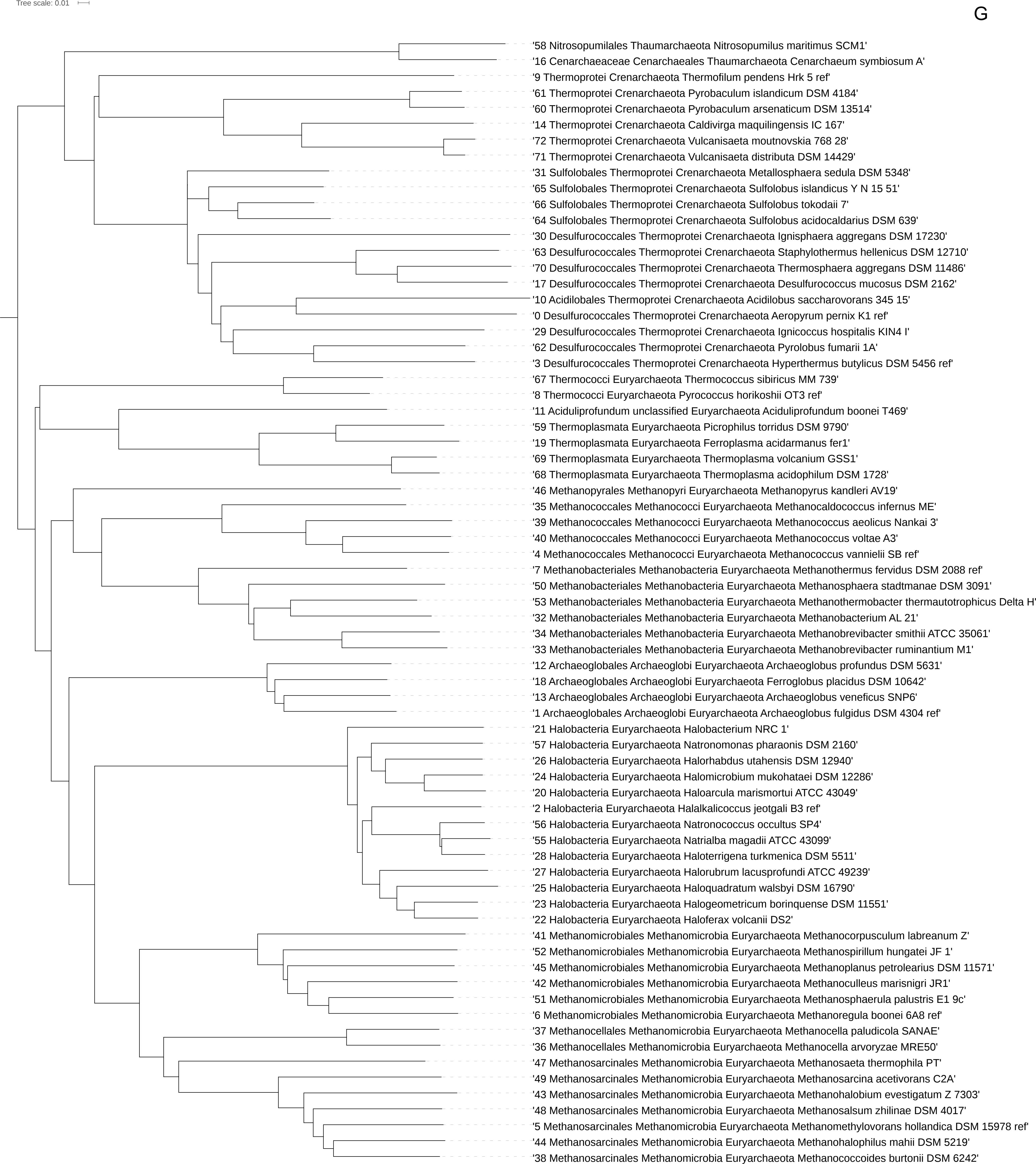

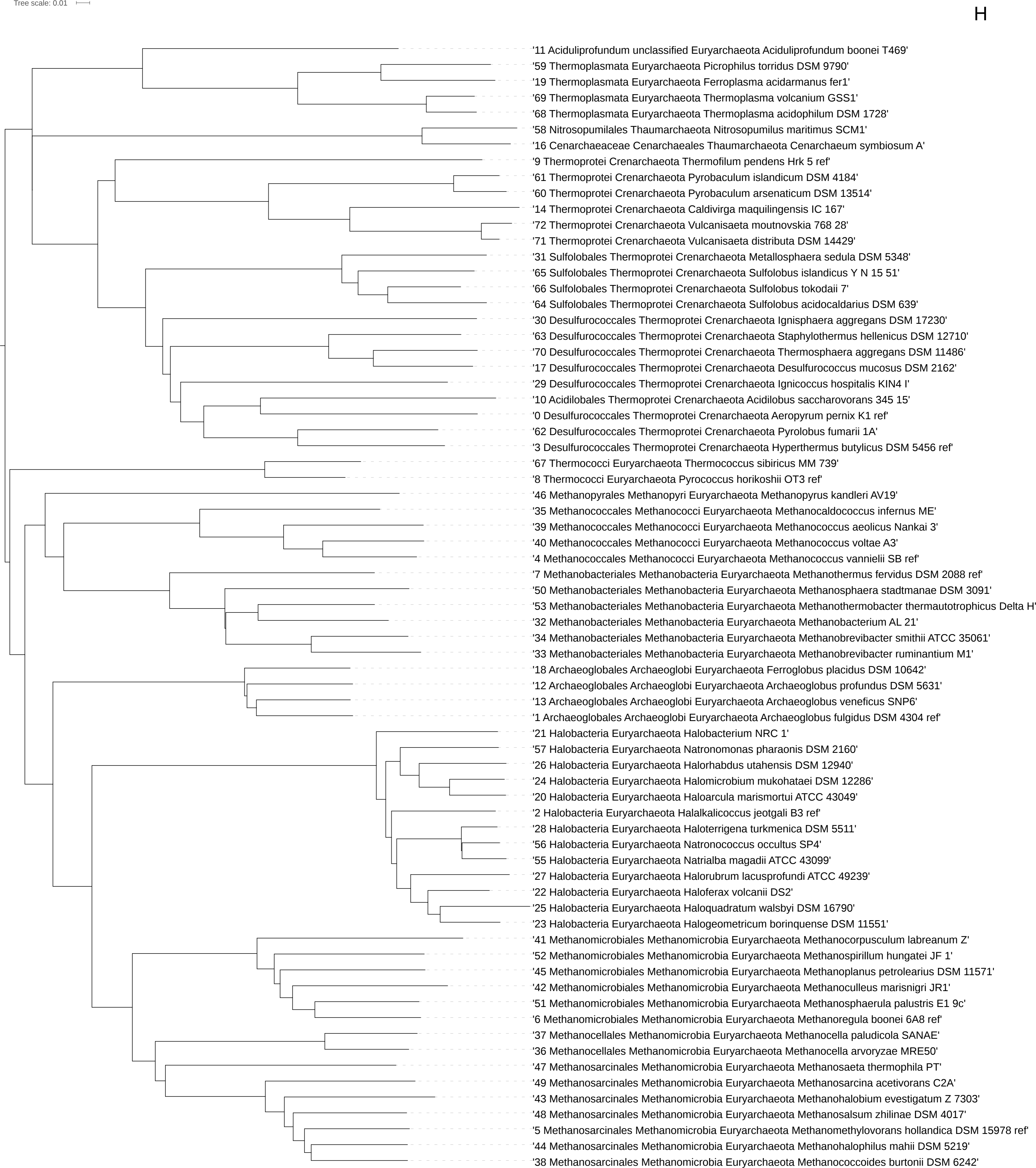

Supplement: S9 Fig — A) kmacs on raw 73 archaea. Unfiltered and unpruned. B) kmacs on raw 71 archaea. Unfiltered and pruned. C) kmacs on 71 archaea. Filtered of mobile elements and pruned. D) kmacs on 71 archaea. Filtered of mobile elements, pruned, and filtered by stability and conservation on o = 0. E) kmacs on 71 archaea. Filtered of mobile elements, pruned, and filtered by stability and conservation on o = 1. F) kmacs on 71 archaea. Filtered of mobile elements, pruned, and filtered by stability and conservation on o = 3. G) kmacs on 71 archaea. Filtered of mobile elements, pruned, and filtered by stability and conservation on o = 5. H) kmacs on 71 archaea. Filtered of mobile elements, pruned, and filtered by stability and conservation on o = 7. (PDF) [file pcbi.1004985.s009.pdf]

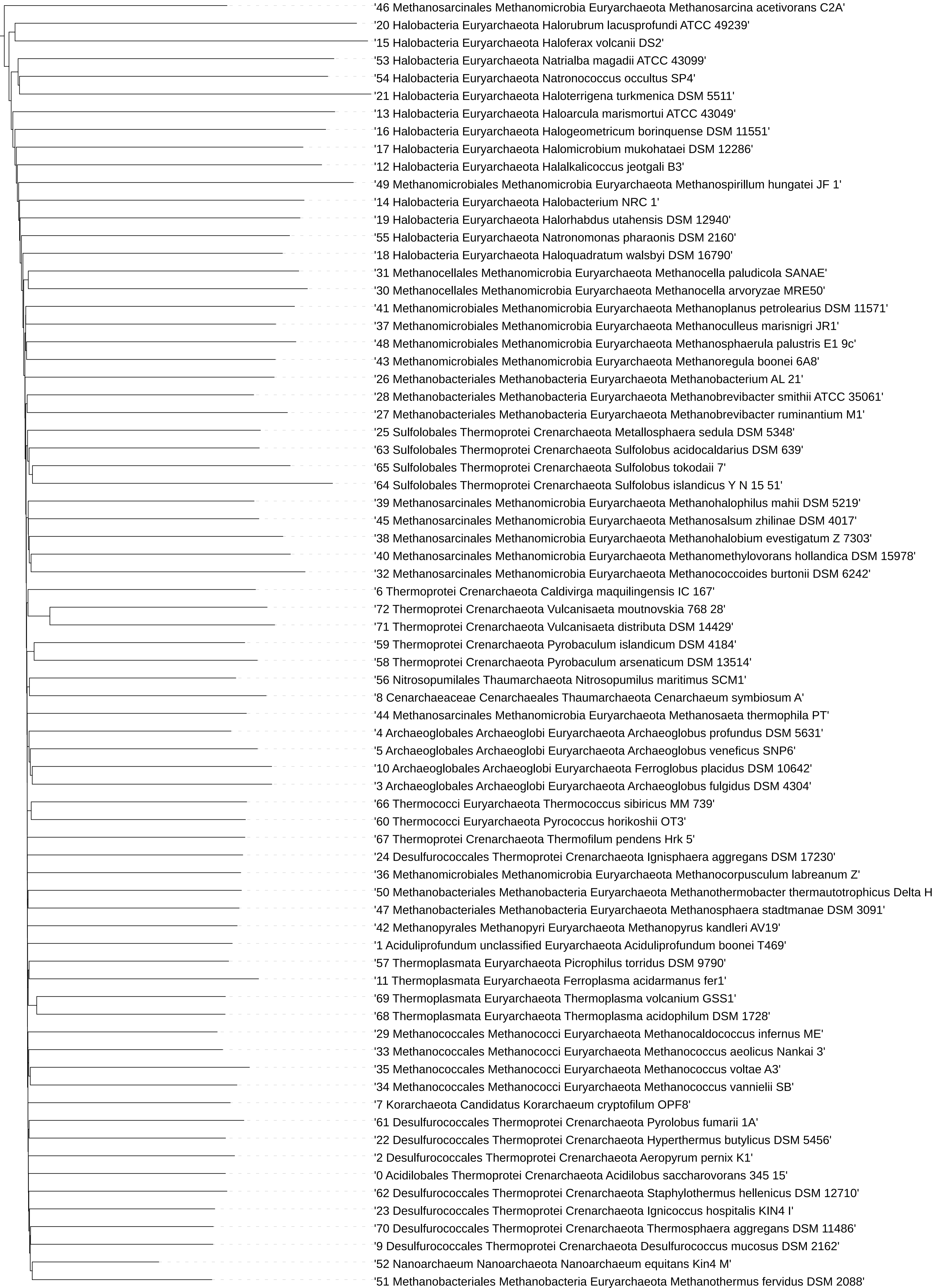

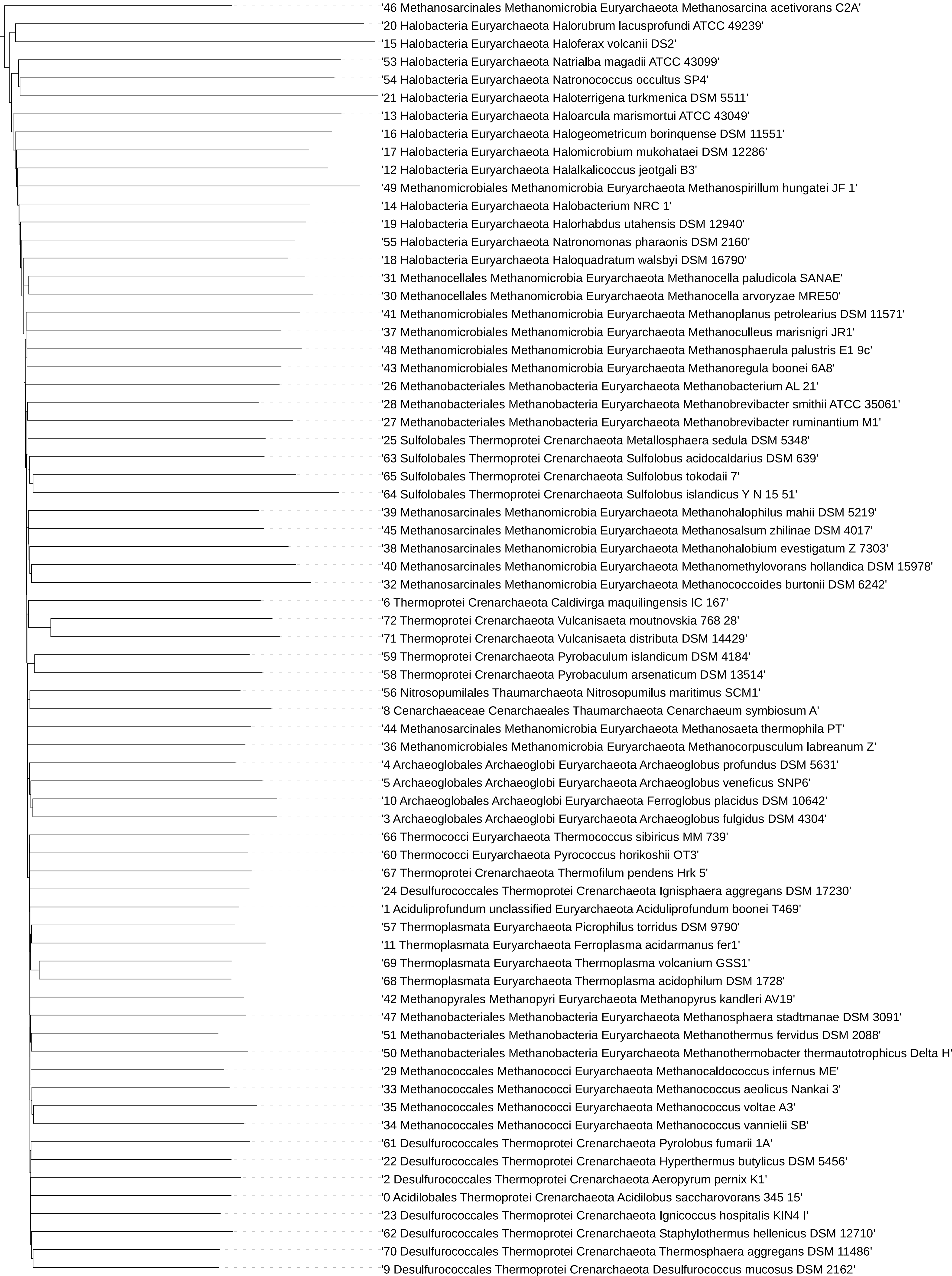

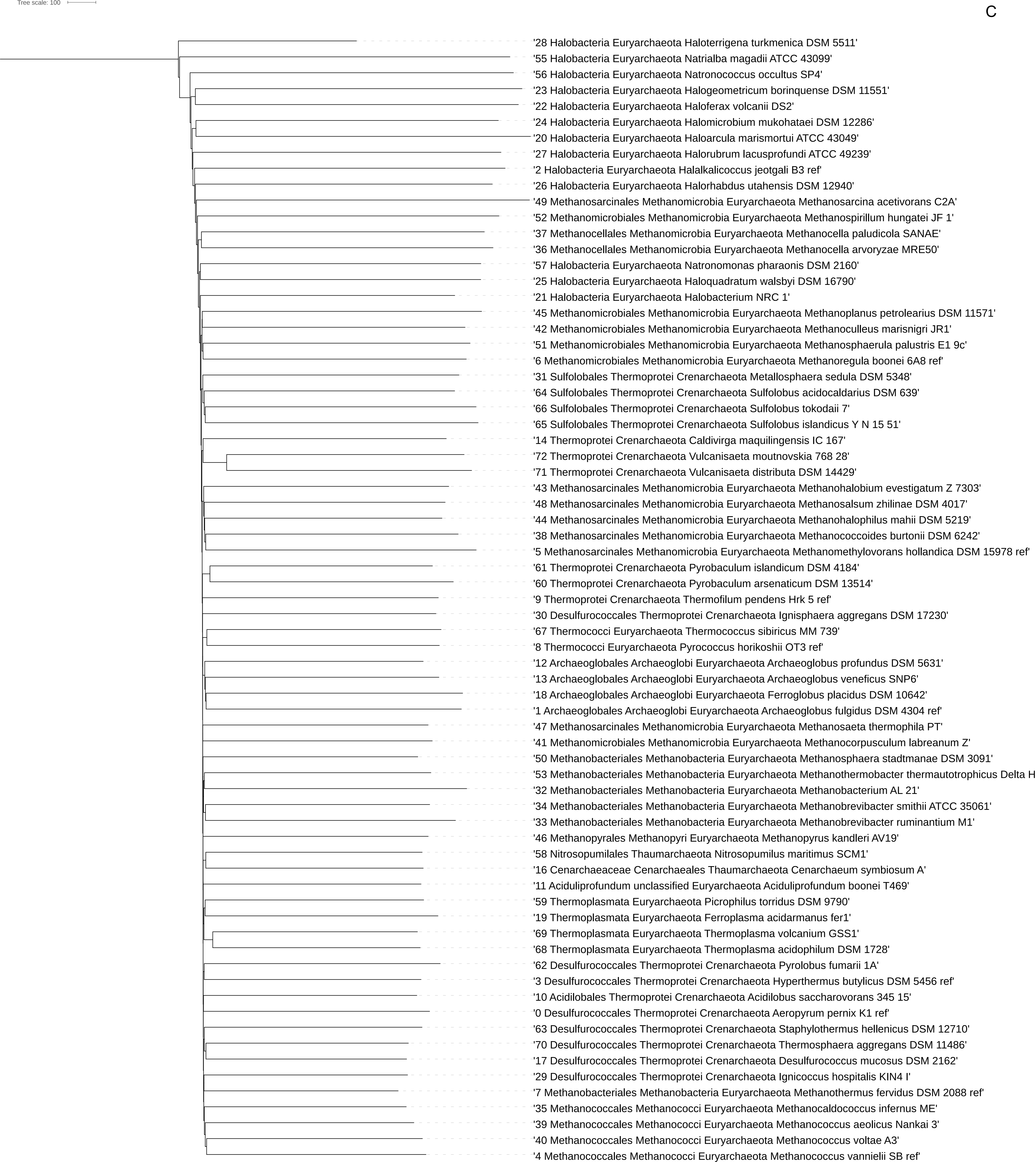

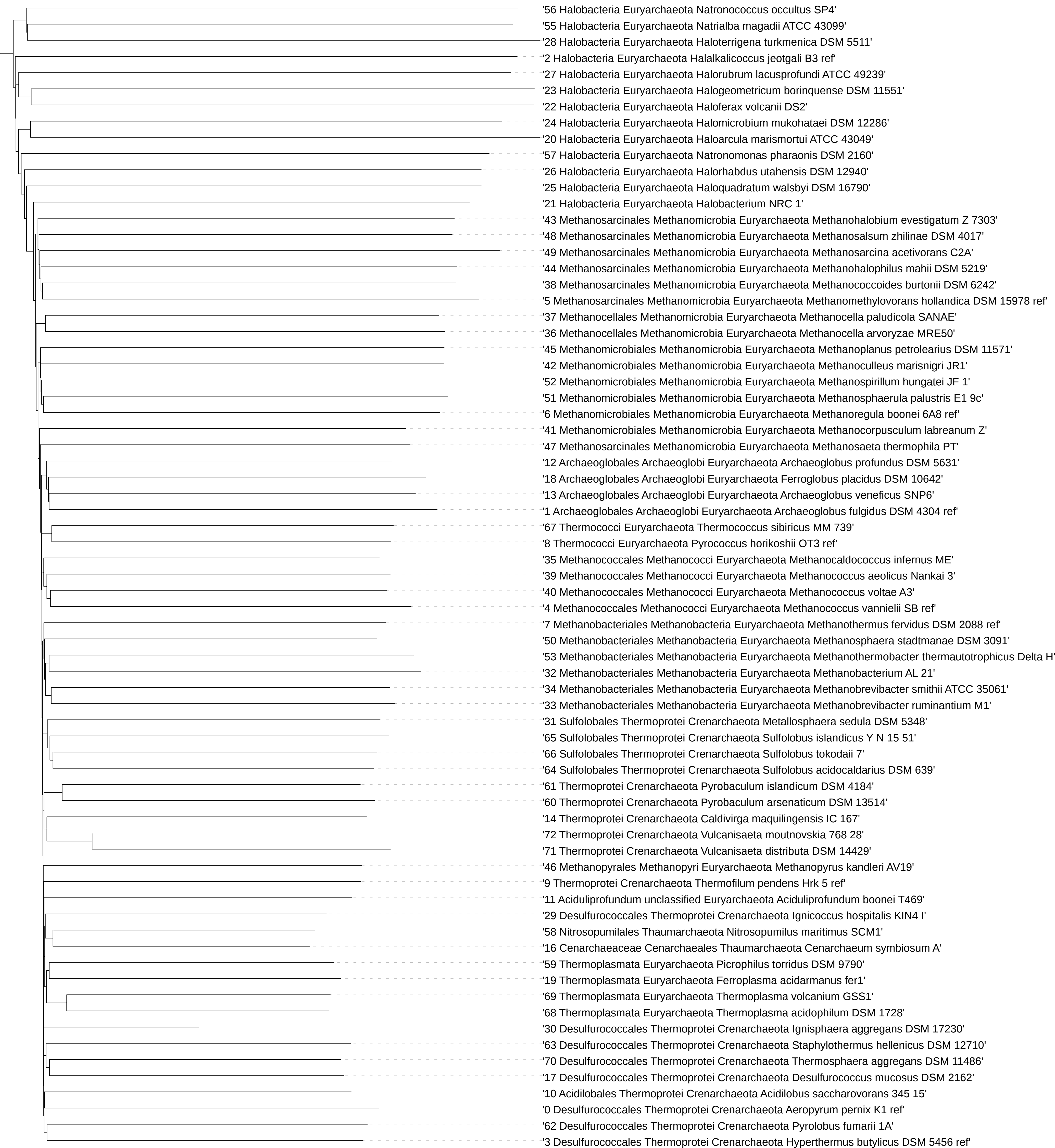

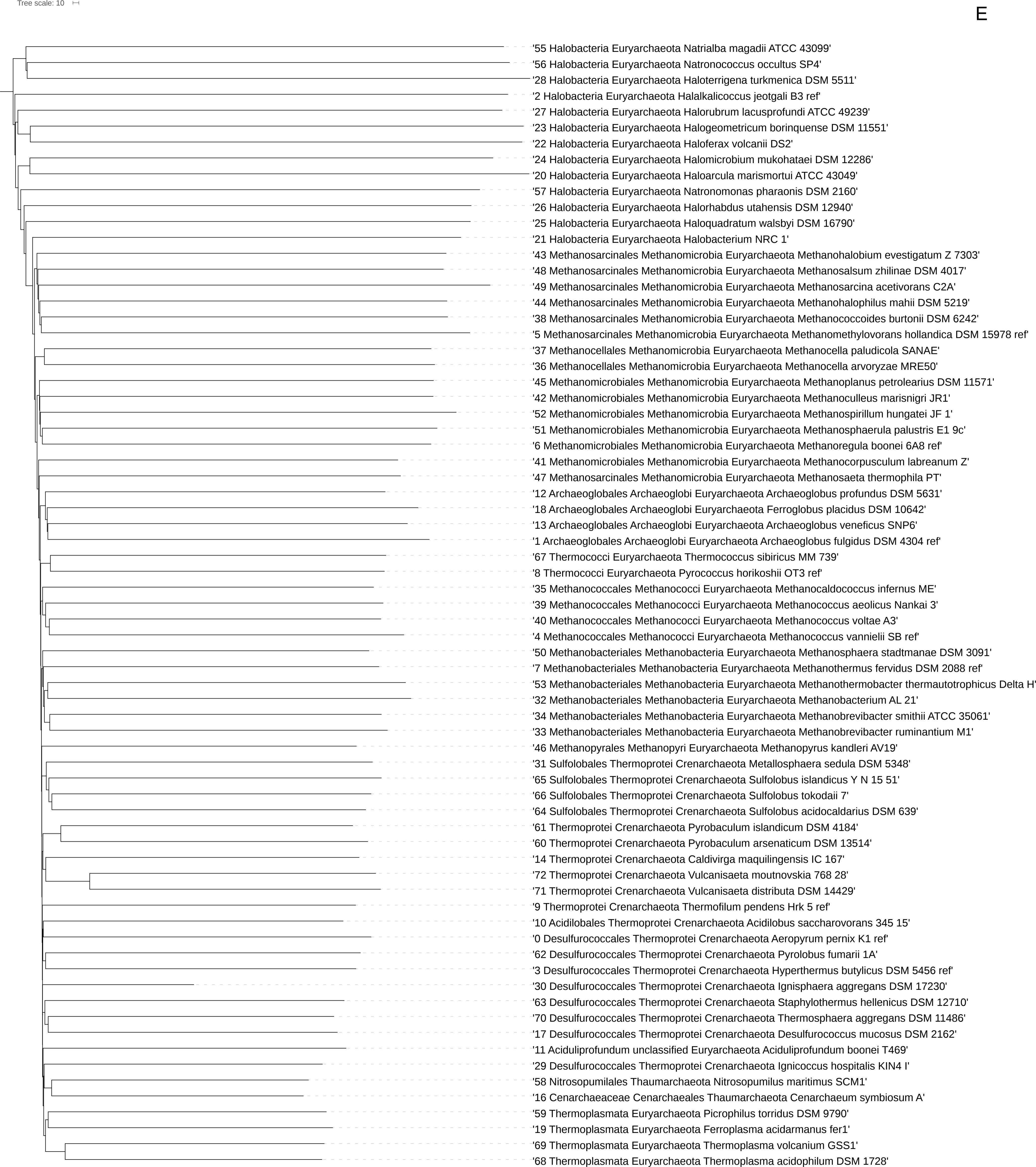

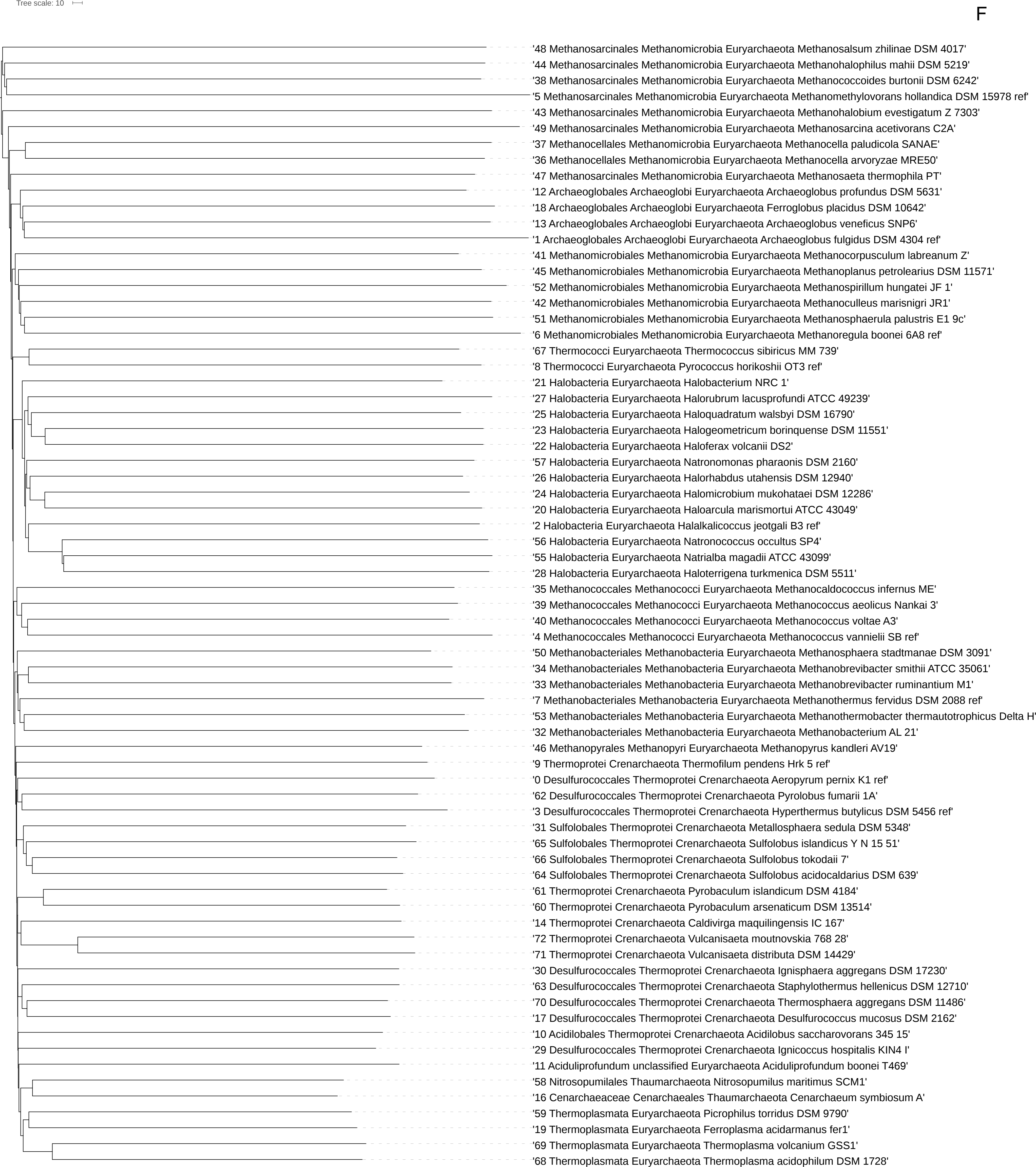

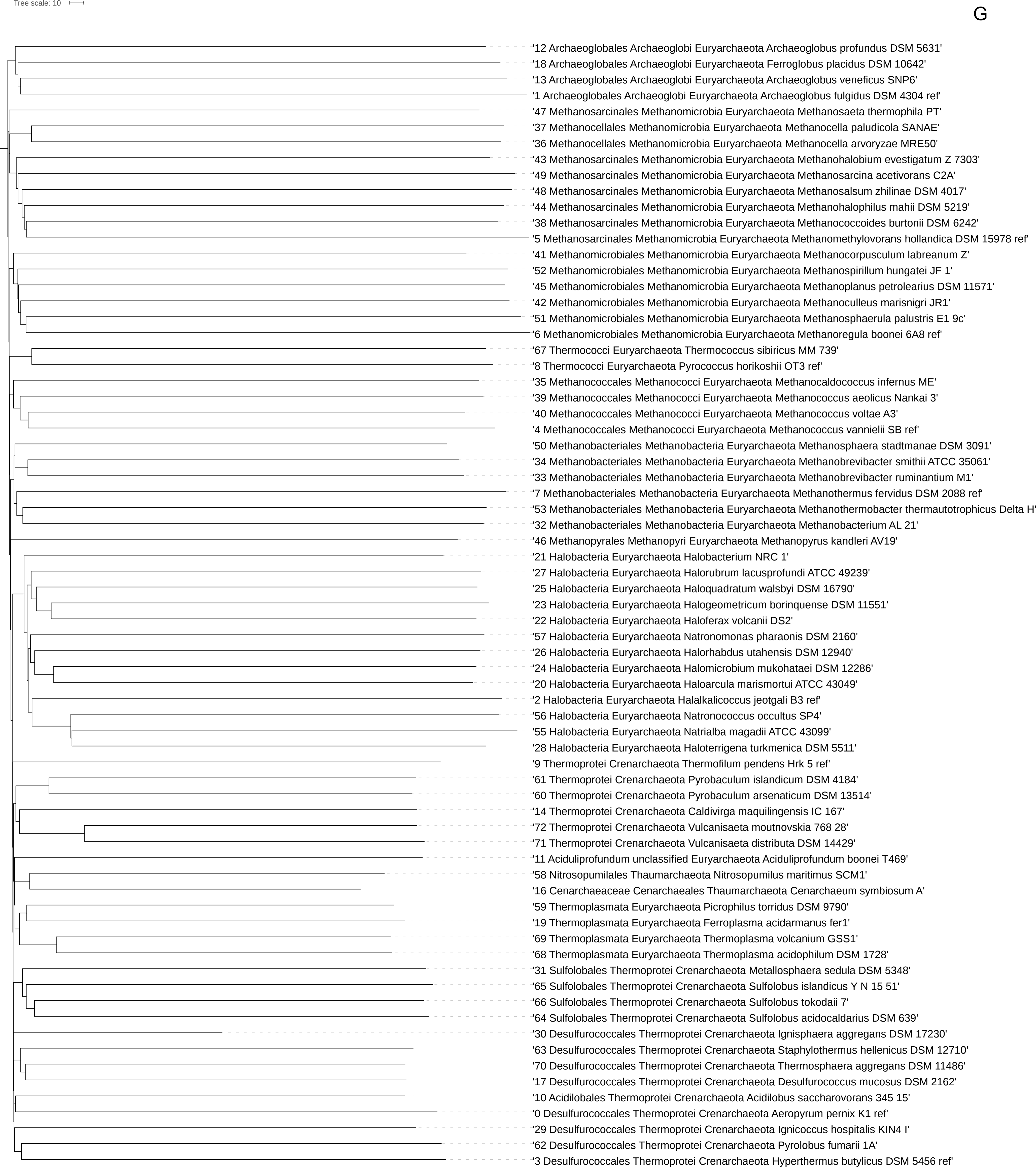

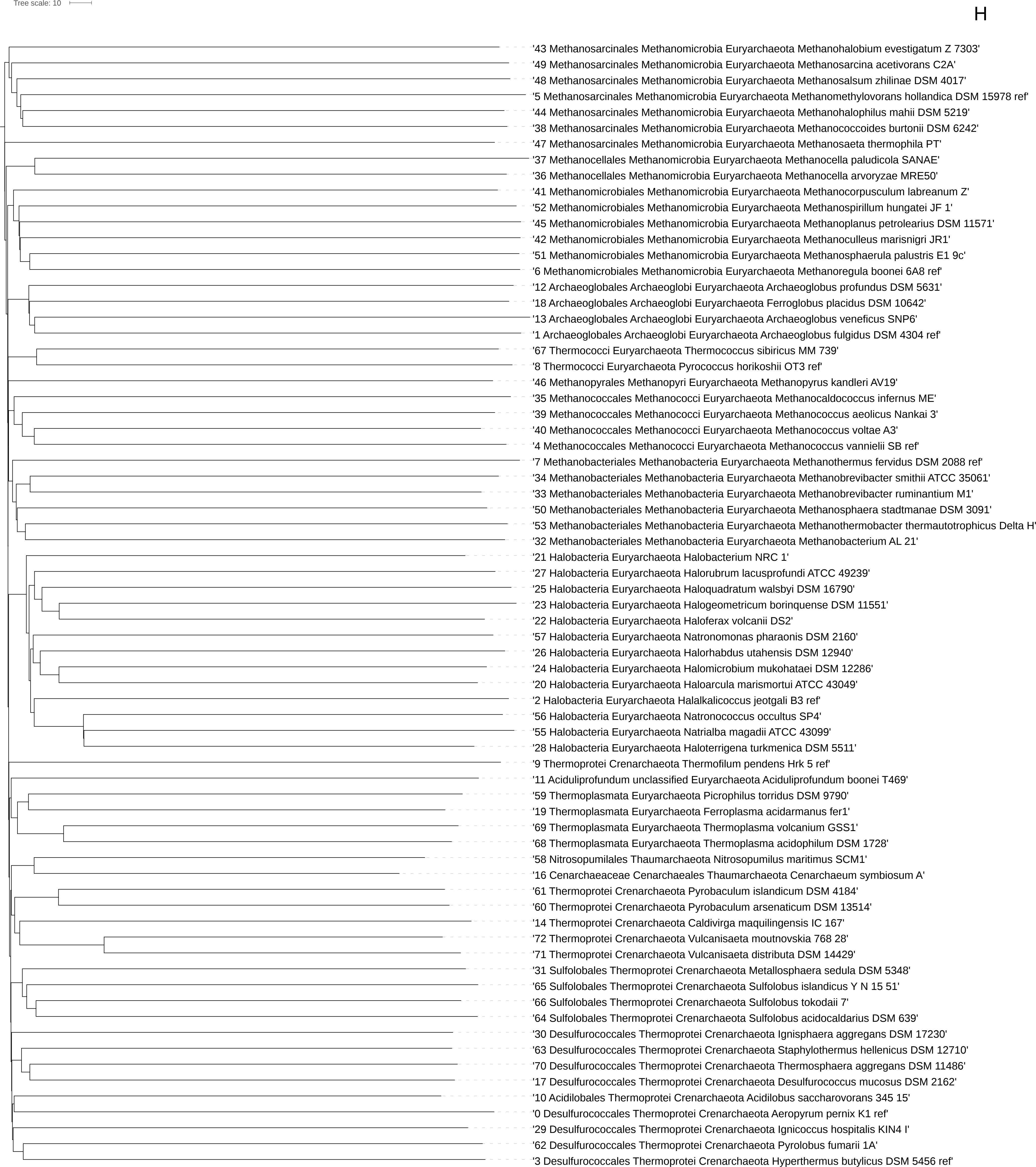

Supplement: S10 Fig — A) SW tree for raw 73 archaea. Unfiltered and unpruned. B) SW tree for raw 71 archaea. Unfiltered and pruned. C) SW tree for 71 archaea. Filtered of mobile elements and pruned. D) SW tree for 71 archaea. Filtered of mobile elements, pruned, and filtered by stability and conservation on o = 0. E) SW tree for on 71 archaea. Filtered of mobile elements, pruned, and filtered by stability and conservation on o = 1. F) SW tree for 71 archaea. Filtered of mobile elements, pruned, and filtered by stability and conservation on o = 3. G) SW tree for 71 archaea. Filtered of mobile elements, pruned, and filtered by stability and conservation on o = 5. H) SW tree for 71 archaea. Filtered of mobile elements, pruned, and filtered by stability and conservation on o = 7. (PDF) [file pcbi.1004985.s010.pdf]

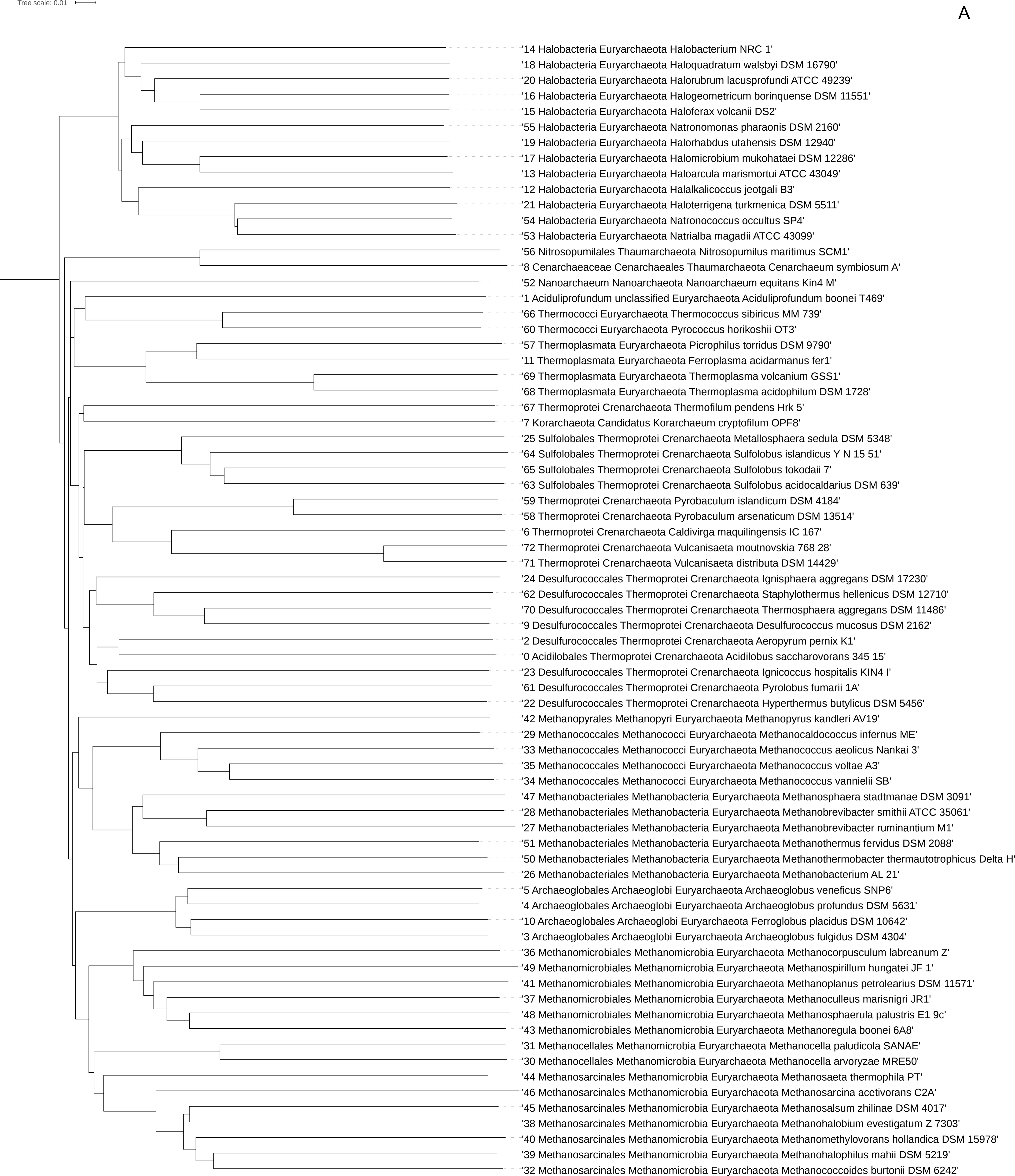

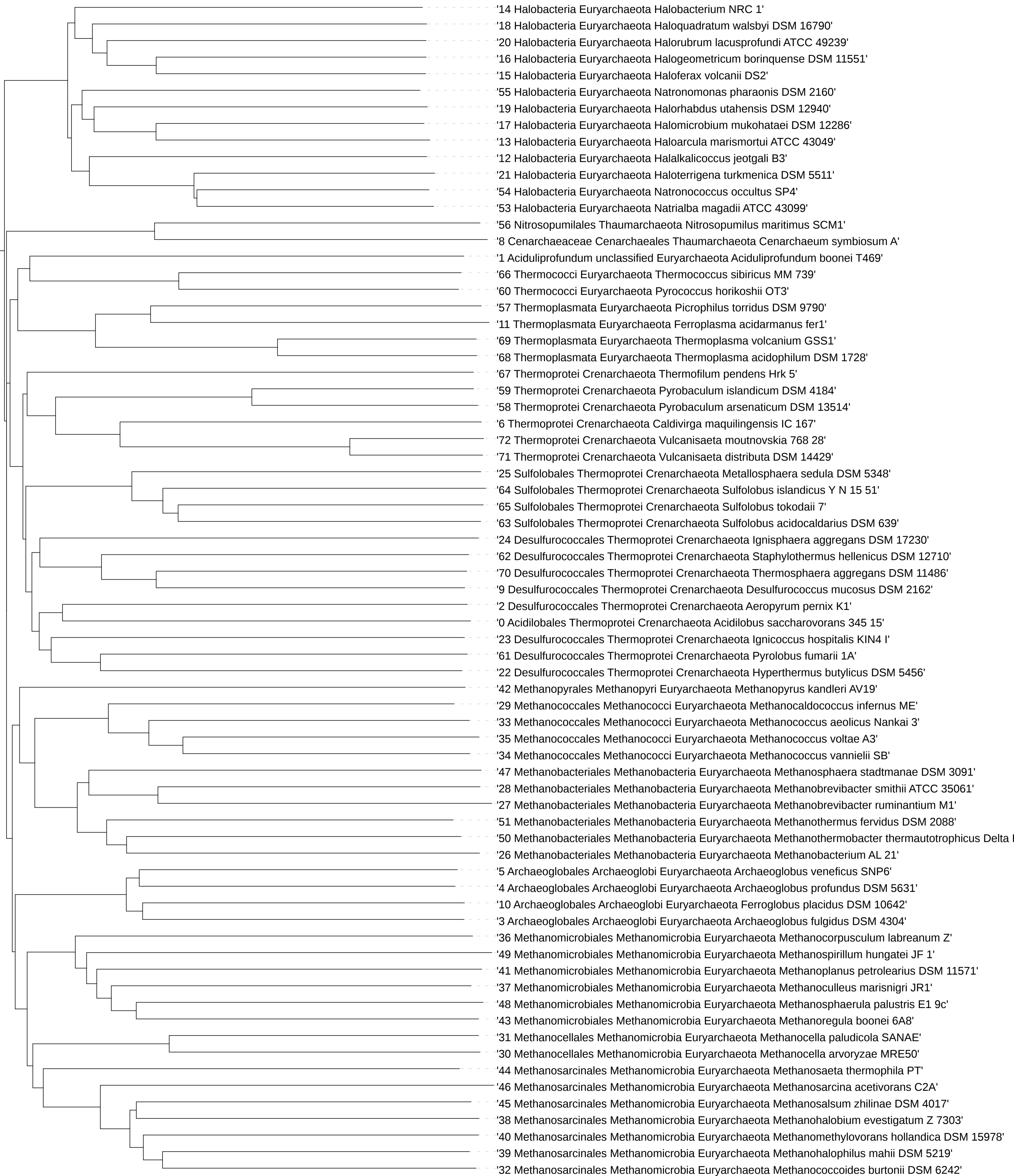

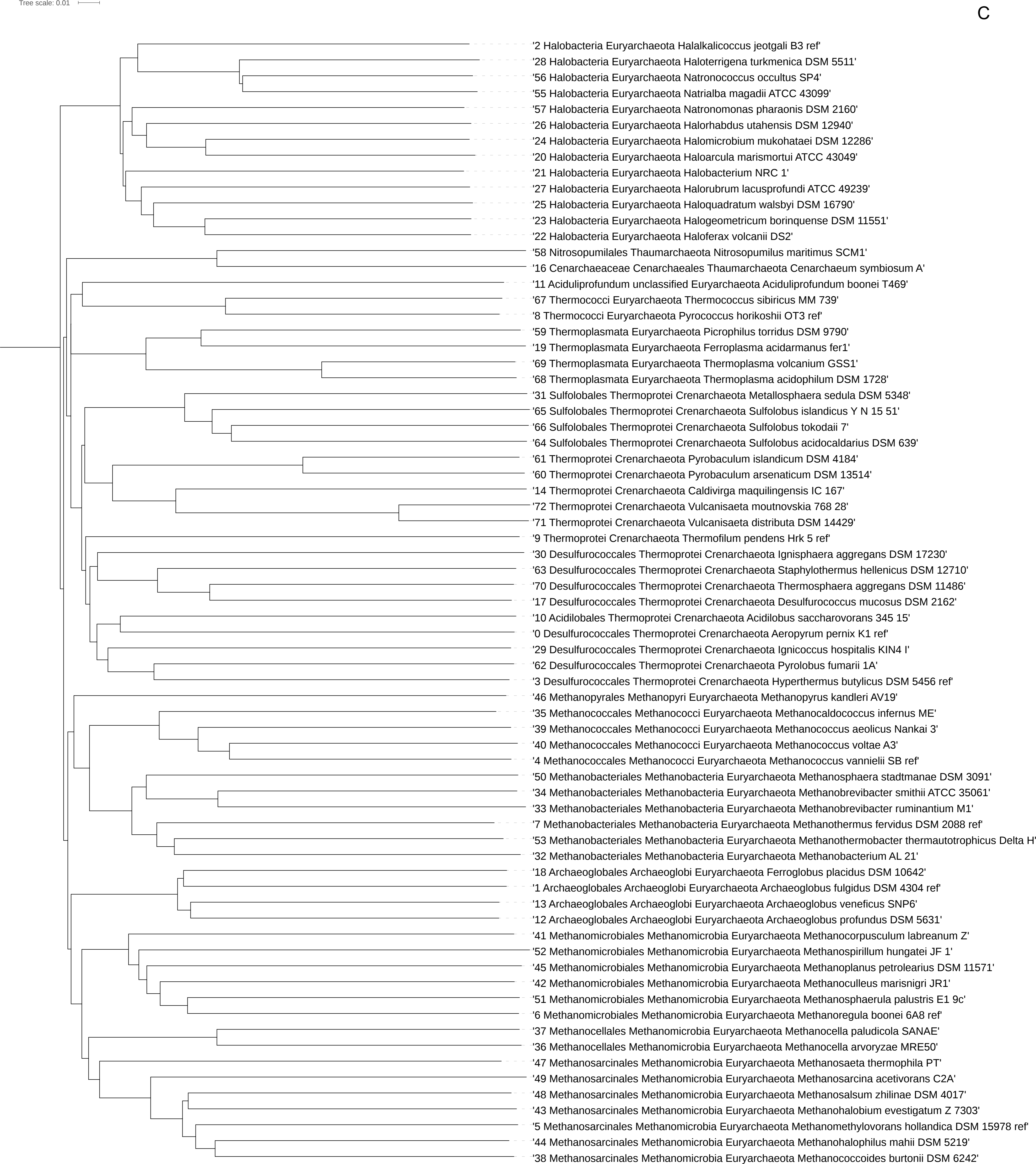

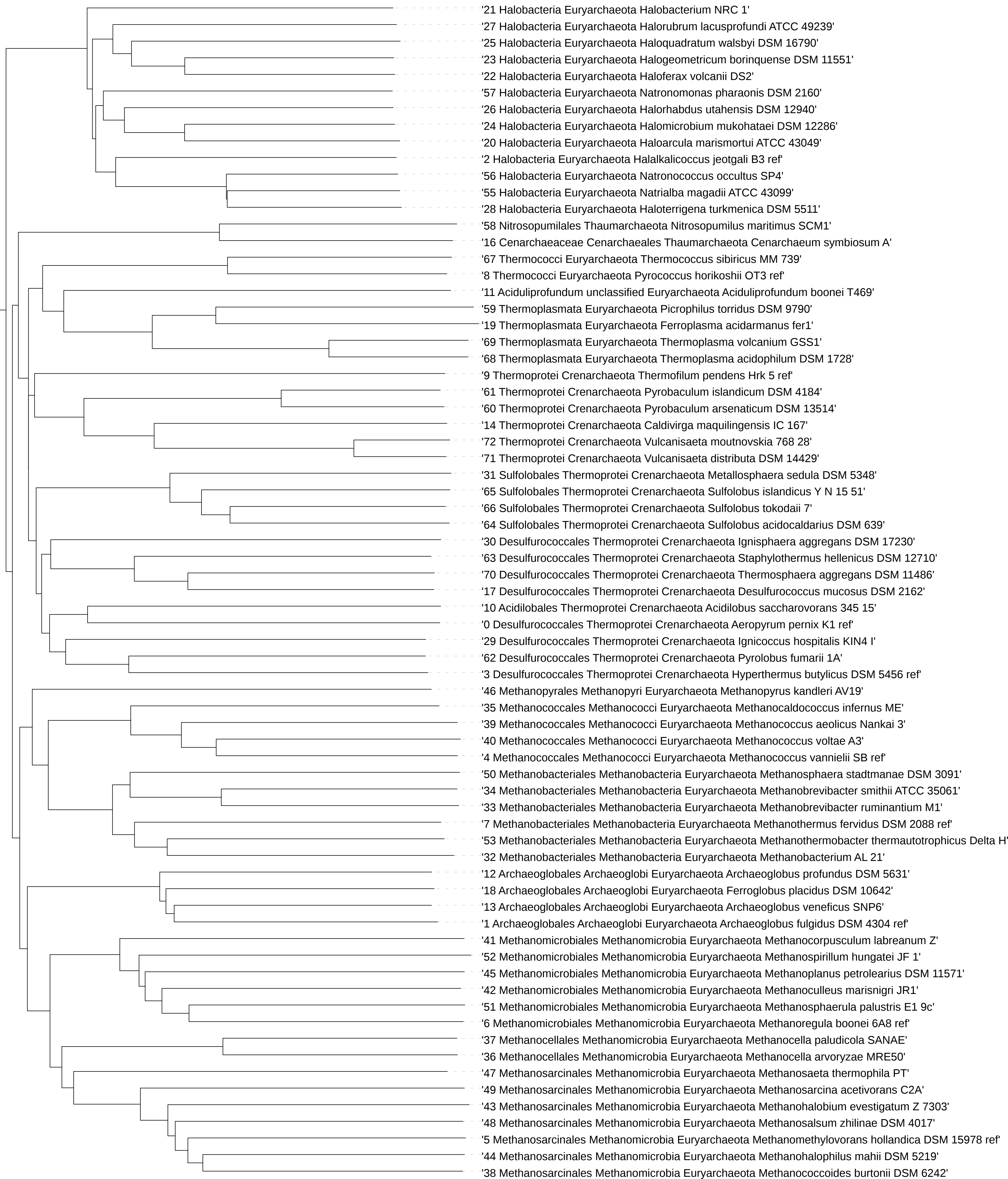

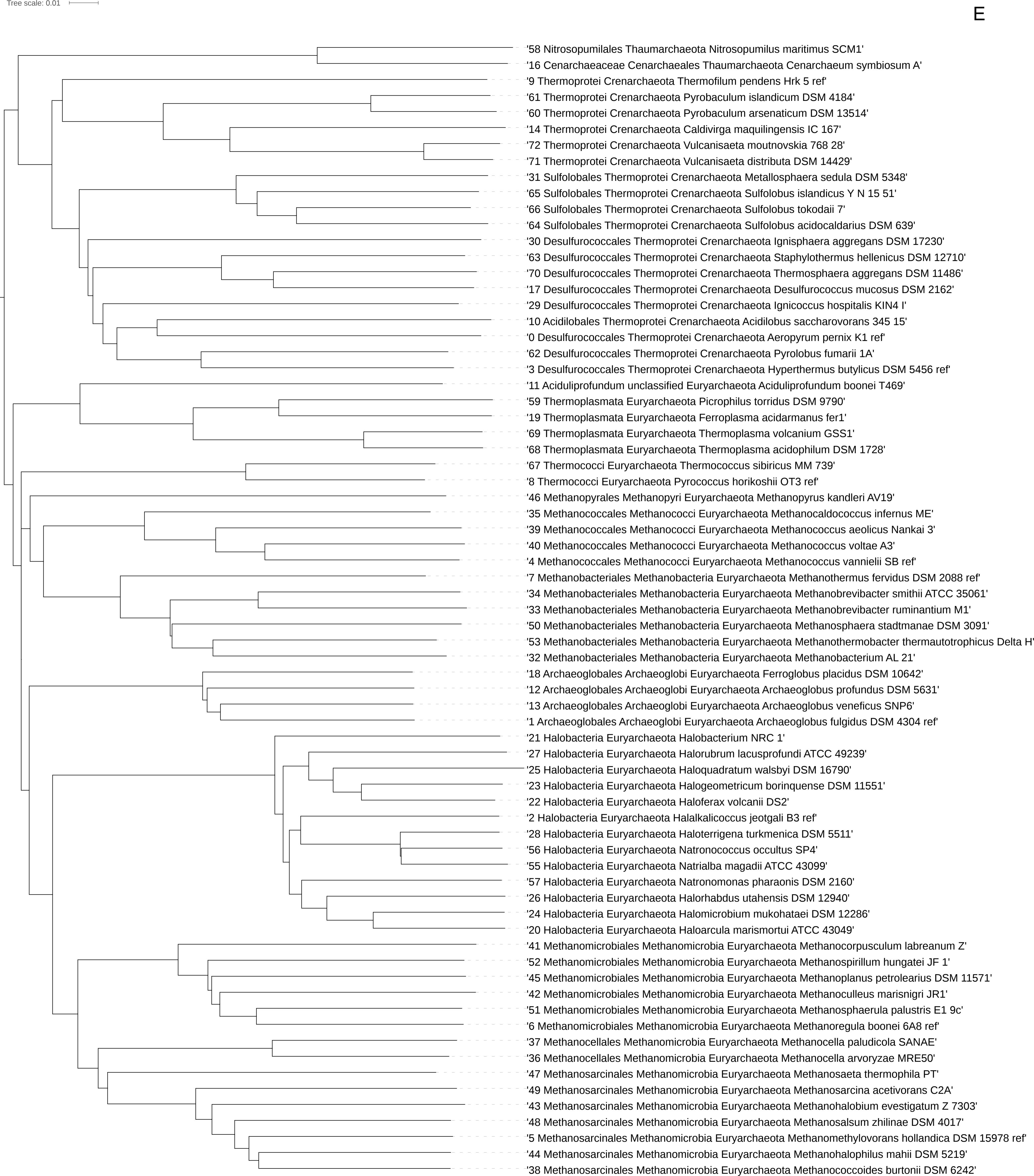

Supplement: S11 Fig — A) ALF tree for raw 73 archaea. Unfiltered and unpruned. B) ALF tree for raw 71 archaea. Unfiltered and pruned. C) ALF tree for 71 archaea. Filtered of mobile elements and pruned. D) ALF tree for 71 archaea. Filtered of mobile elements, pruned, and filtered by stability and conservation on o = 0. E) ALF tree for 71 archaea. Filtered of mobile elements, pruned, and filtered by stability and conservation on o = 7. (PDF) [file pcbi.1004985.s011.pdf]

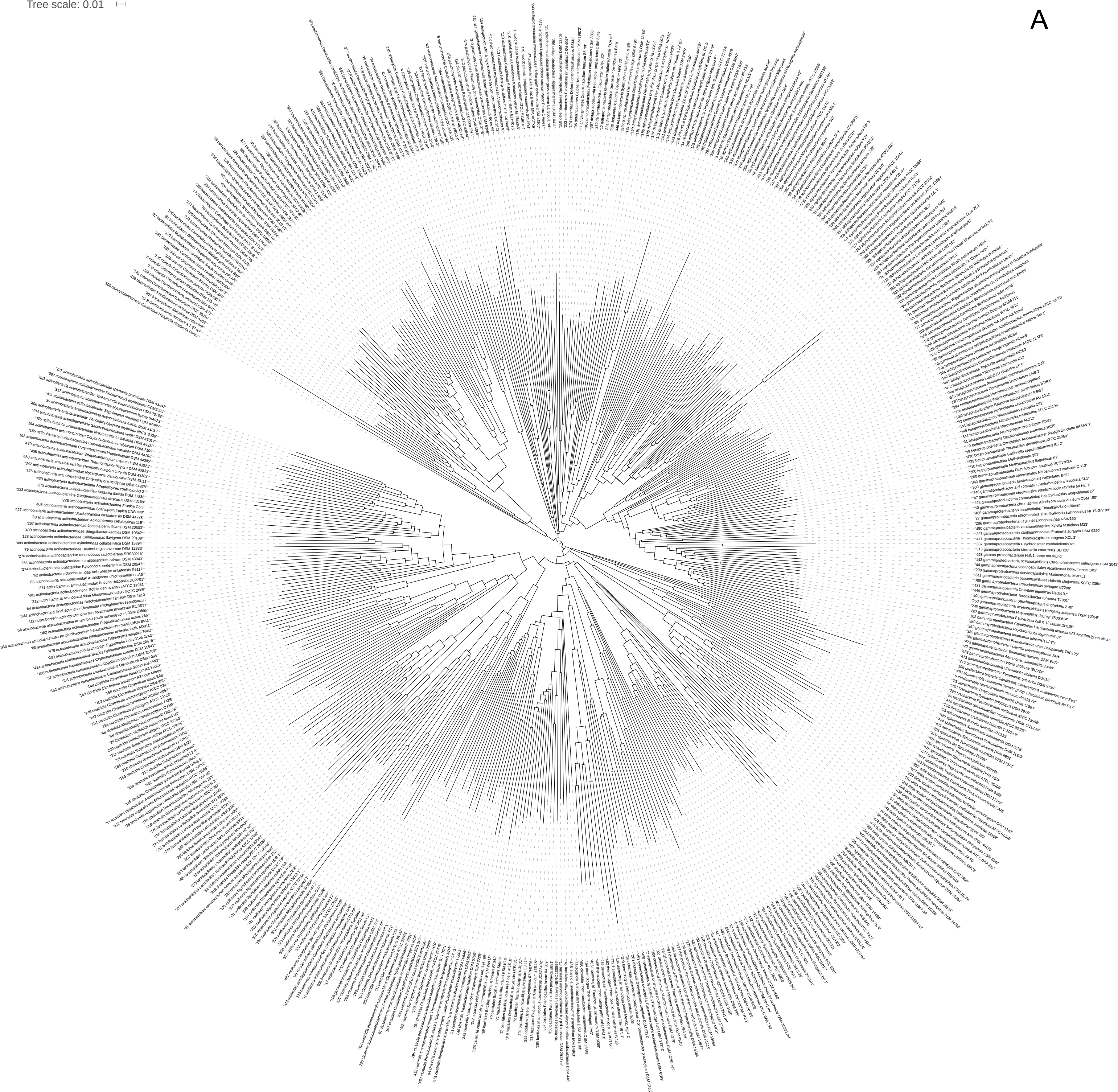

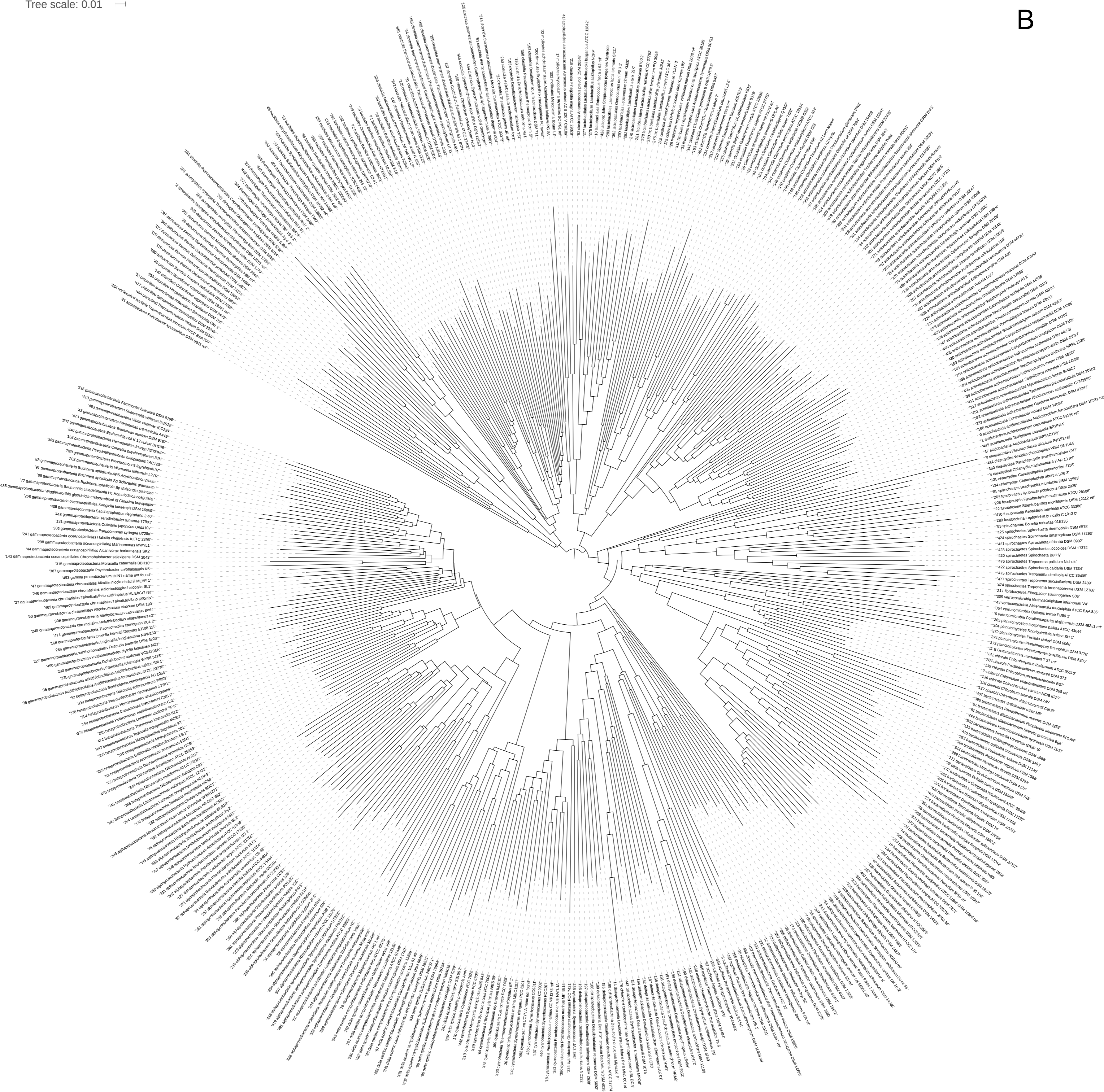

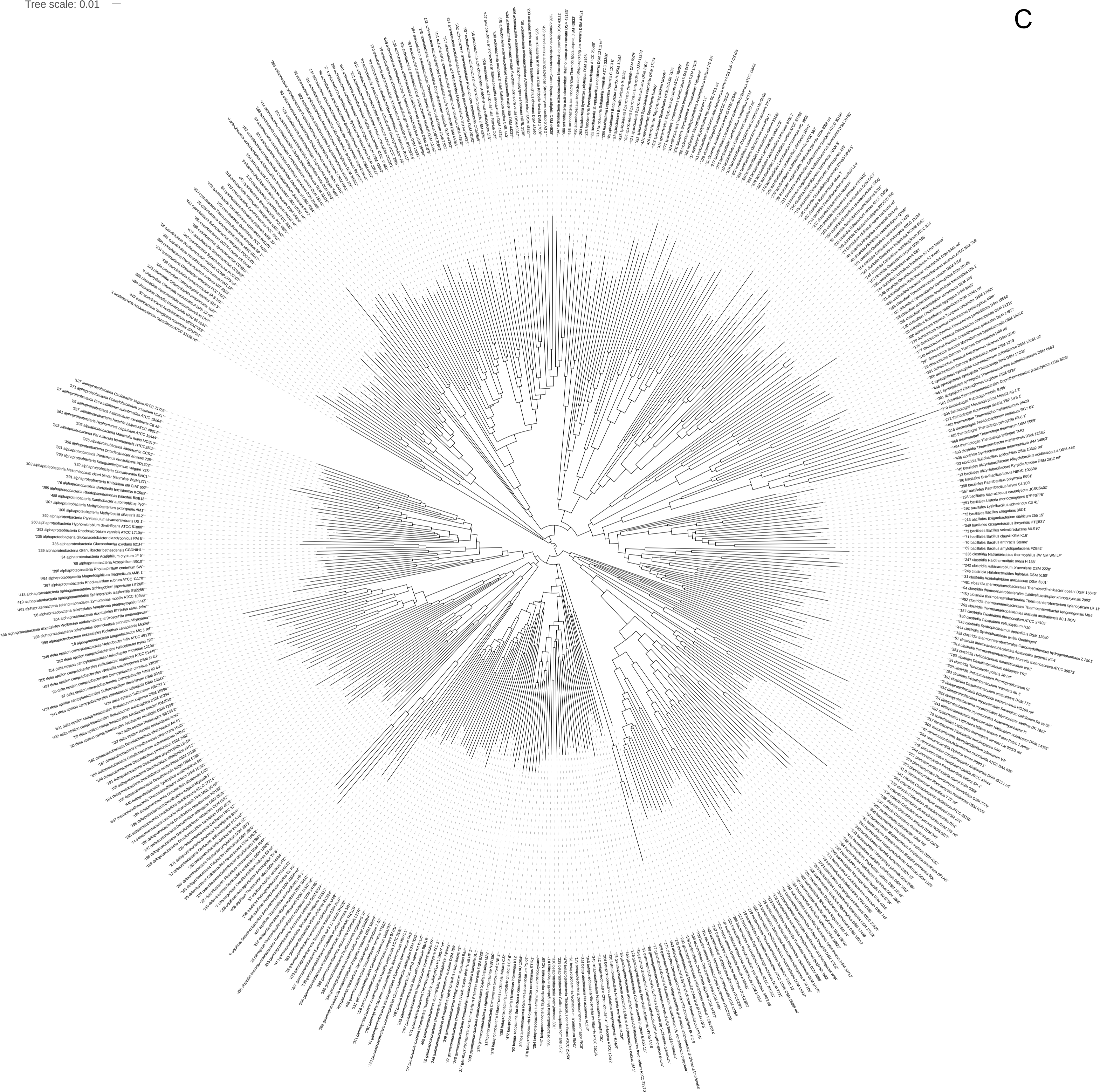

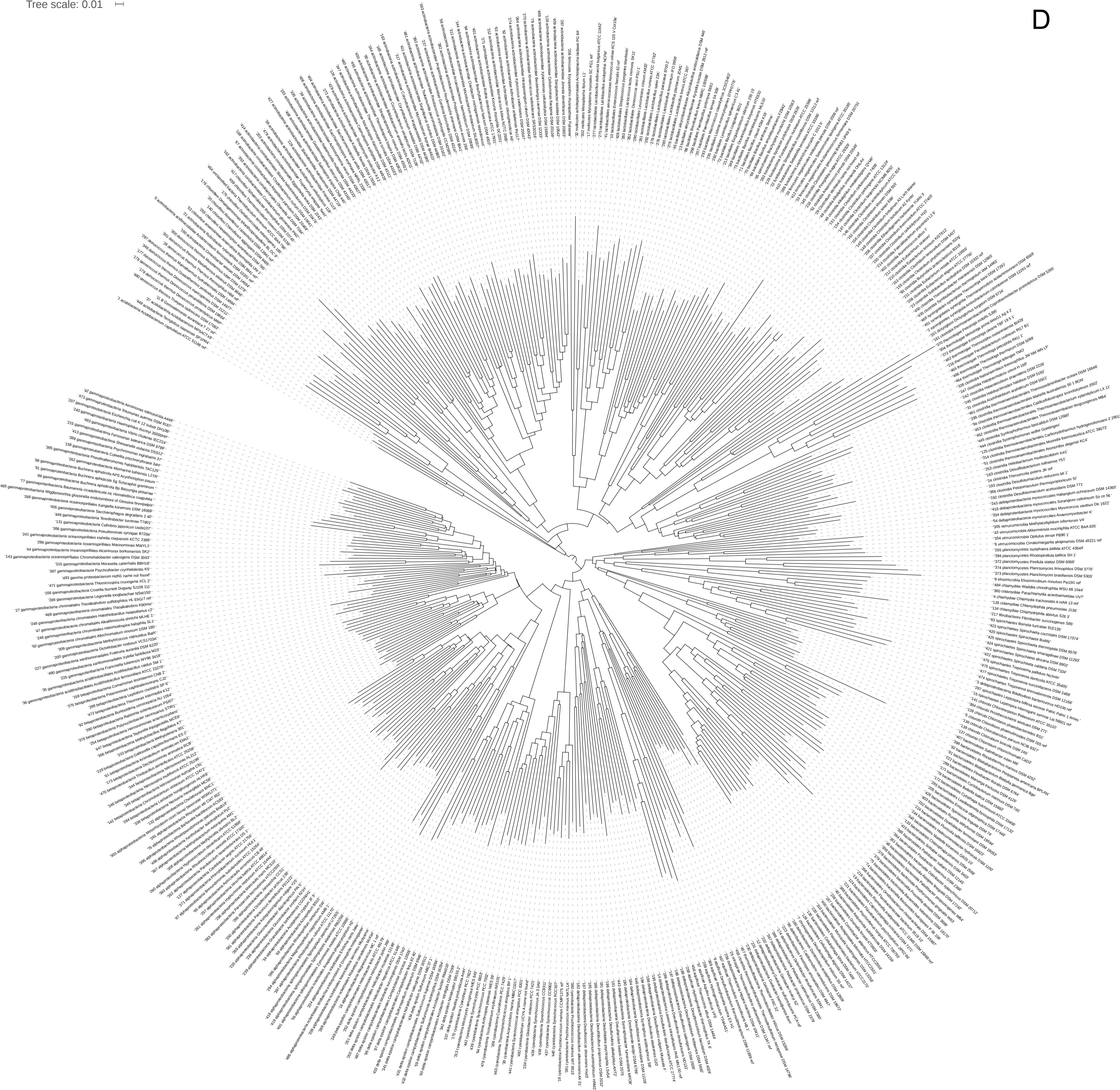

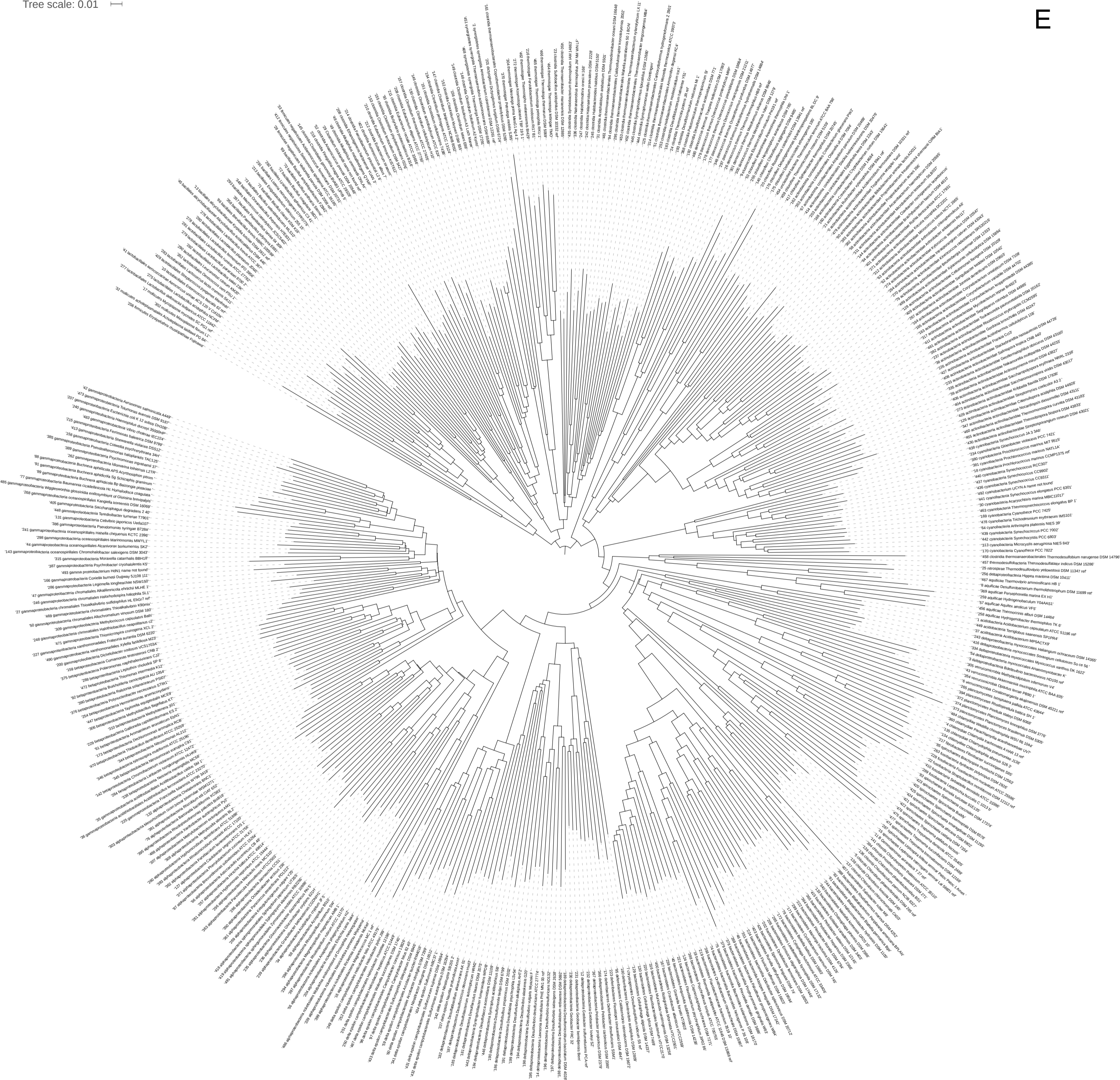

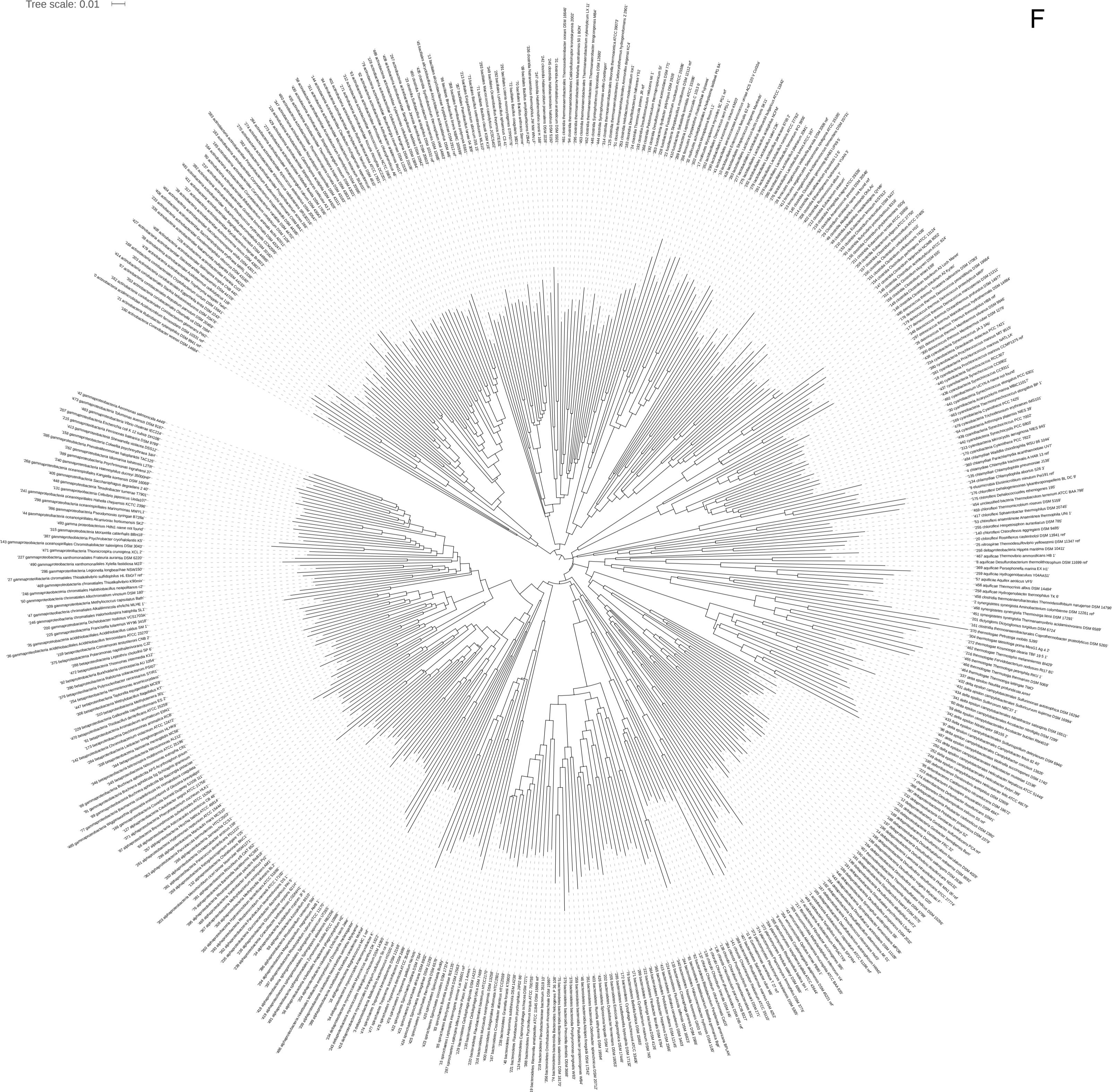

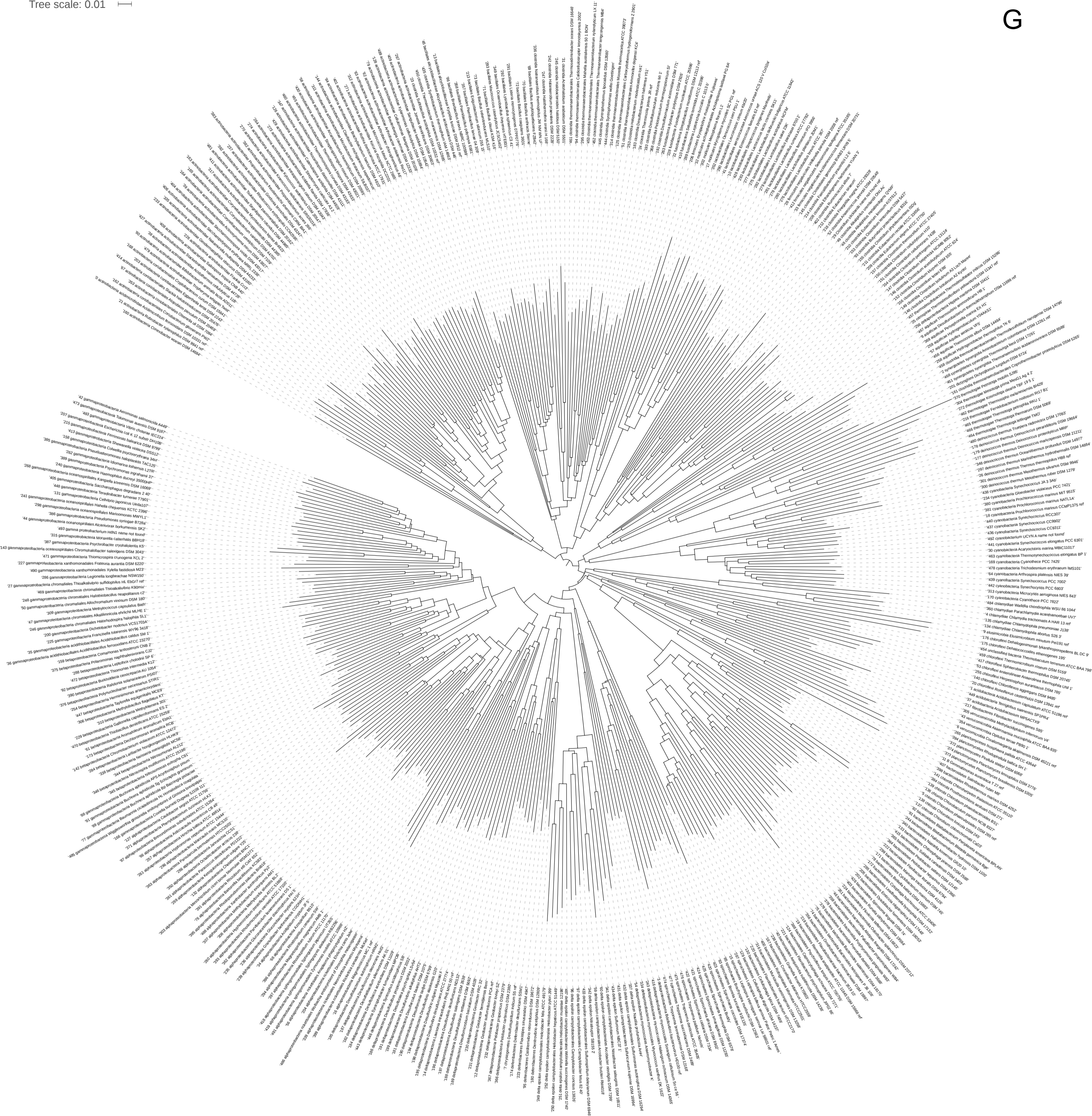

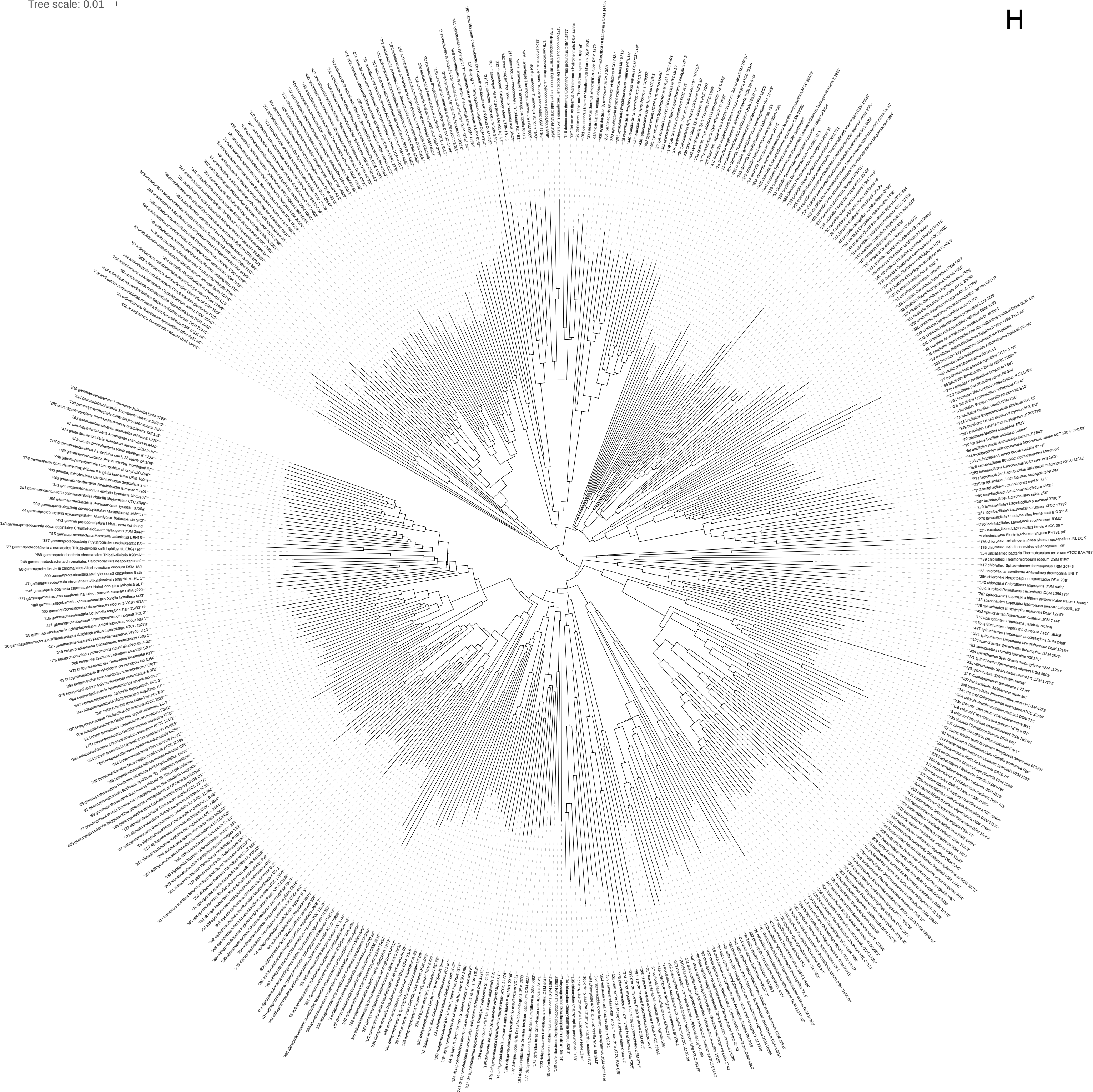

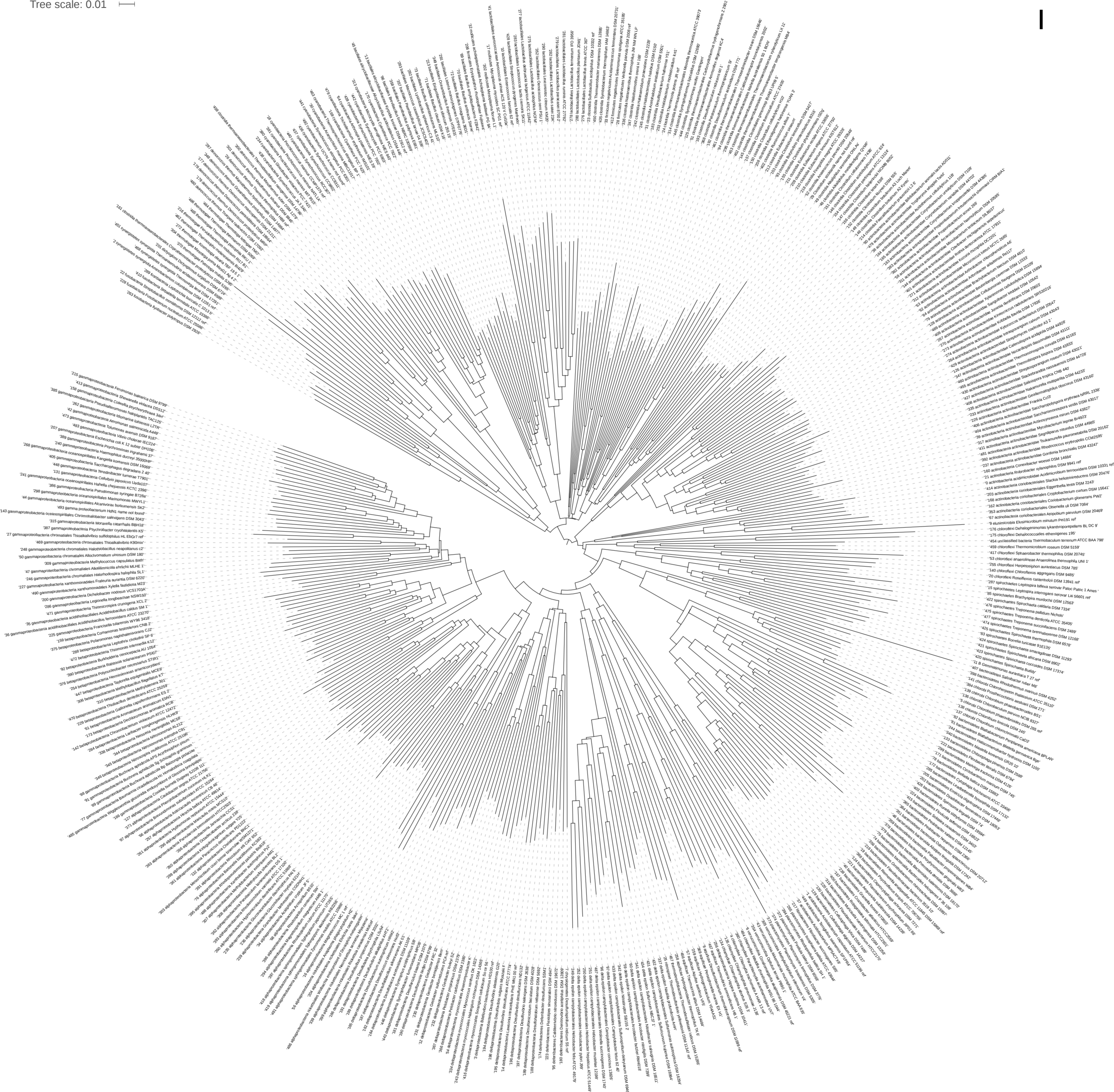

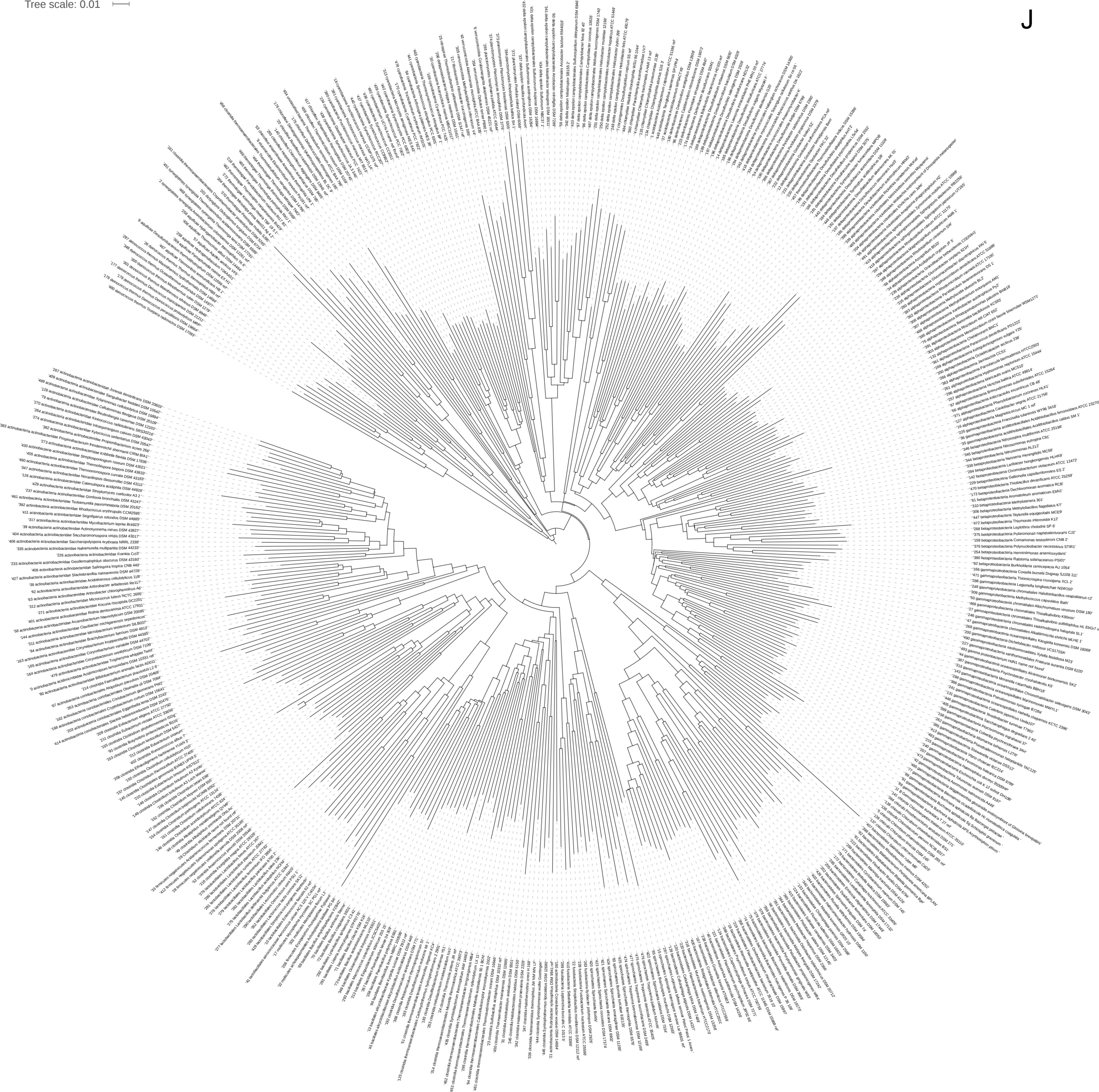

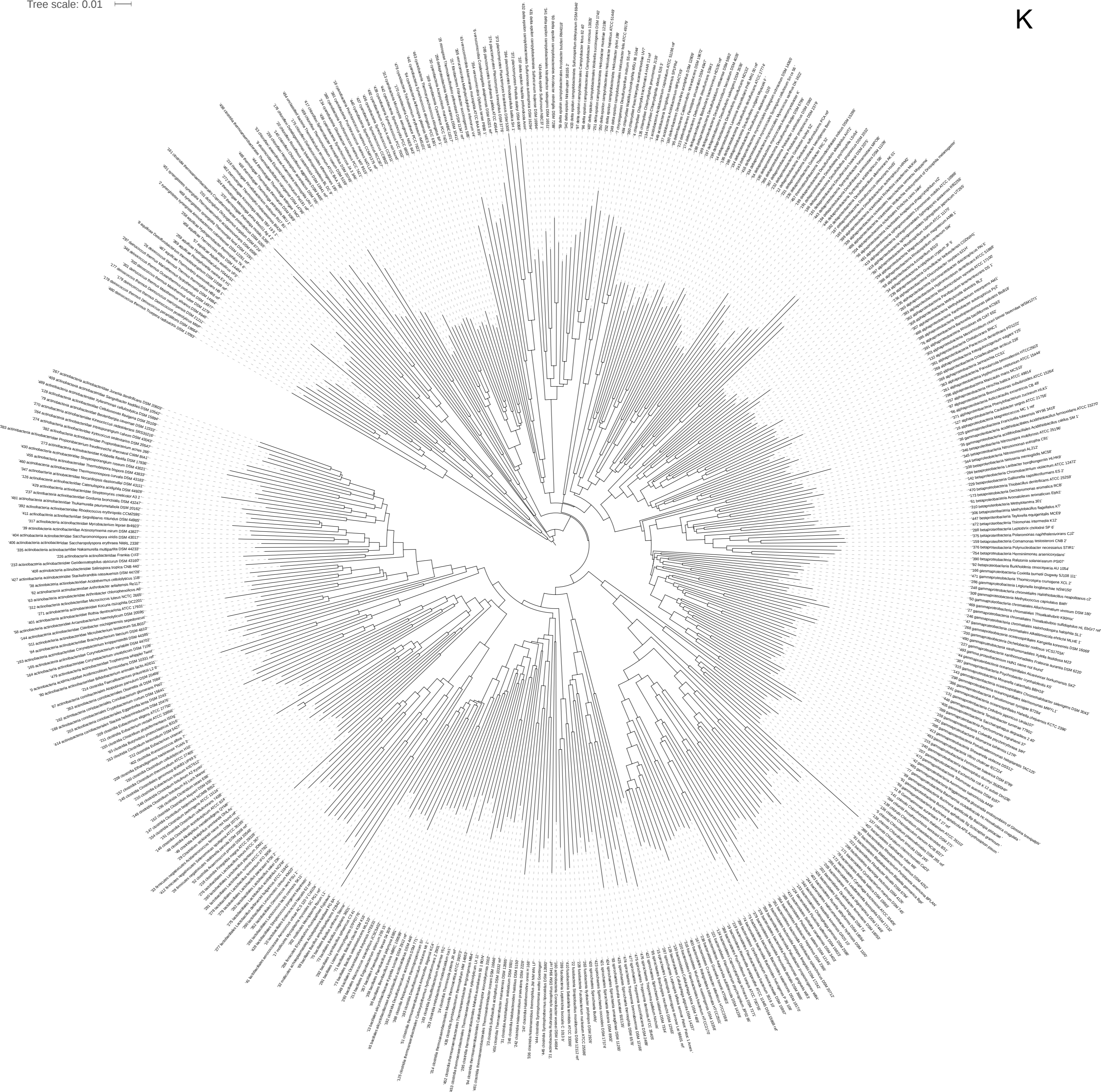

Supplement: S12 Fig — A) Raw ST-tree for 495 bacteria. Unfiltered and unpruned. B) Raw ST-tree for 445 bacteria. Unfiltered and pruned. C) ST- tree for 445 bacteria. Filtered of mobile elements and pruned. D) ST-tree for 445 bacteria. Filtered of mobile elements, pruned, and filtered by stability and conservation on o = 0. E) ST-tree for 445 bacteria. Filtered of mobile elements, pruned, and filtered by stability and conservation on o = 1. F) ST-tree for 445 bacteria. Filtered of mobile elements, pruned, and filtered by stability and conservation on o = 3. G) ST-tree for 445 bacteria. Filtered of mobile elements, pruned, filtered by stability and conservation on o = 3, and final pair-wise HGT correction applied. H) ST-tree for 445 bacteria. Filtered of mobile elements, pruned, and filtered by stability and conservation on o = 5. I) ST-tree for 445 bacteria. Filtered of mobile elements, pruned, filtered by stability and conservation on o = 5, and final pair-wise HGT correction applied. J) ST-tree for 445 bacteria. Filtered of mobile elements, pruned, and filtered by stability and conservation on o = 7. K) ST-tree for 445 bacteria. Filtered of mobile elements, pruned, filtered by stability and conservation on o = 7, and final pair-wise HGT correction applied. (PDF) [file pcbi.1004985.s012.pdf]

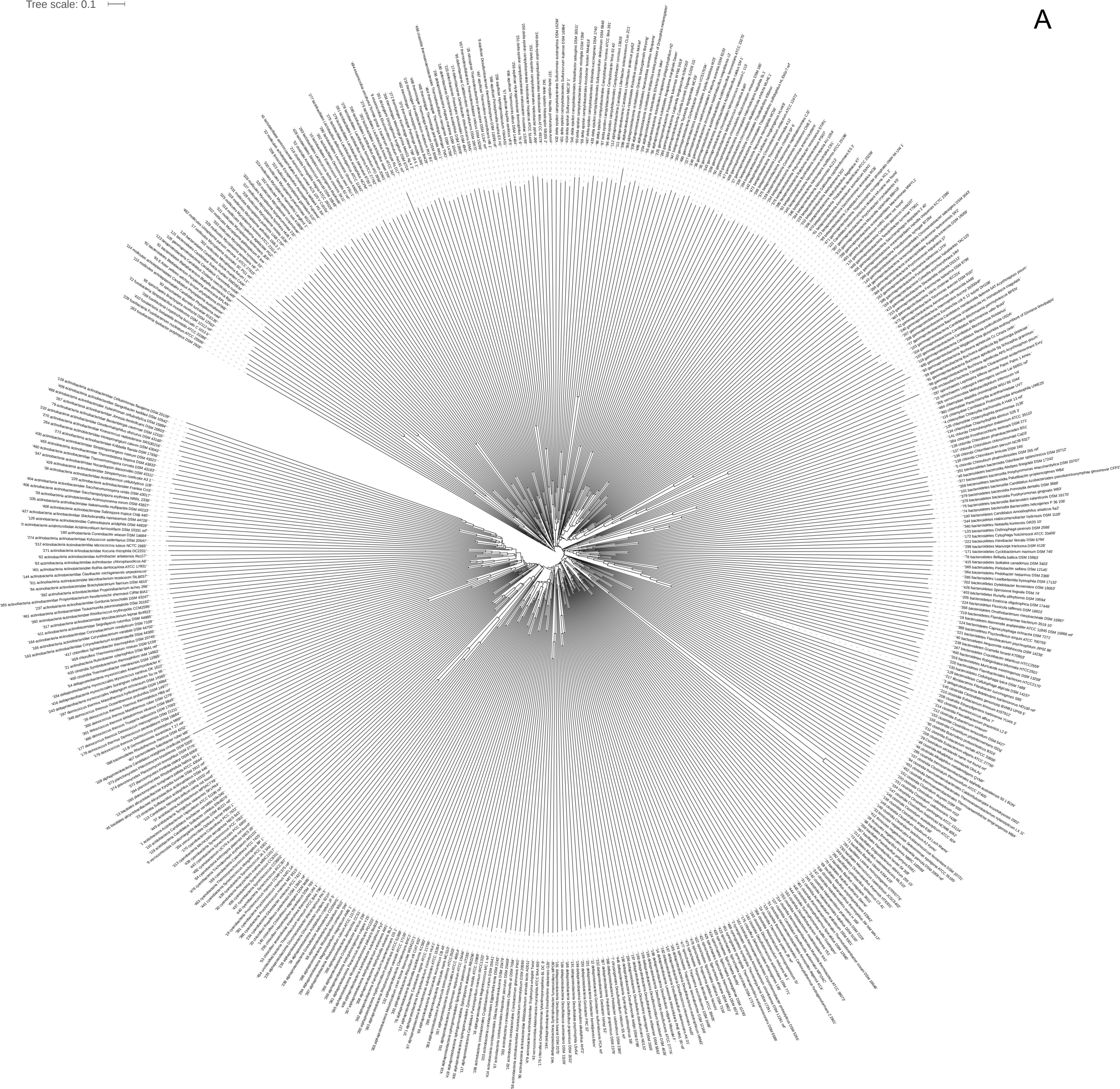

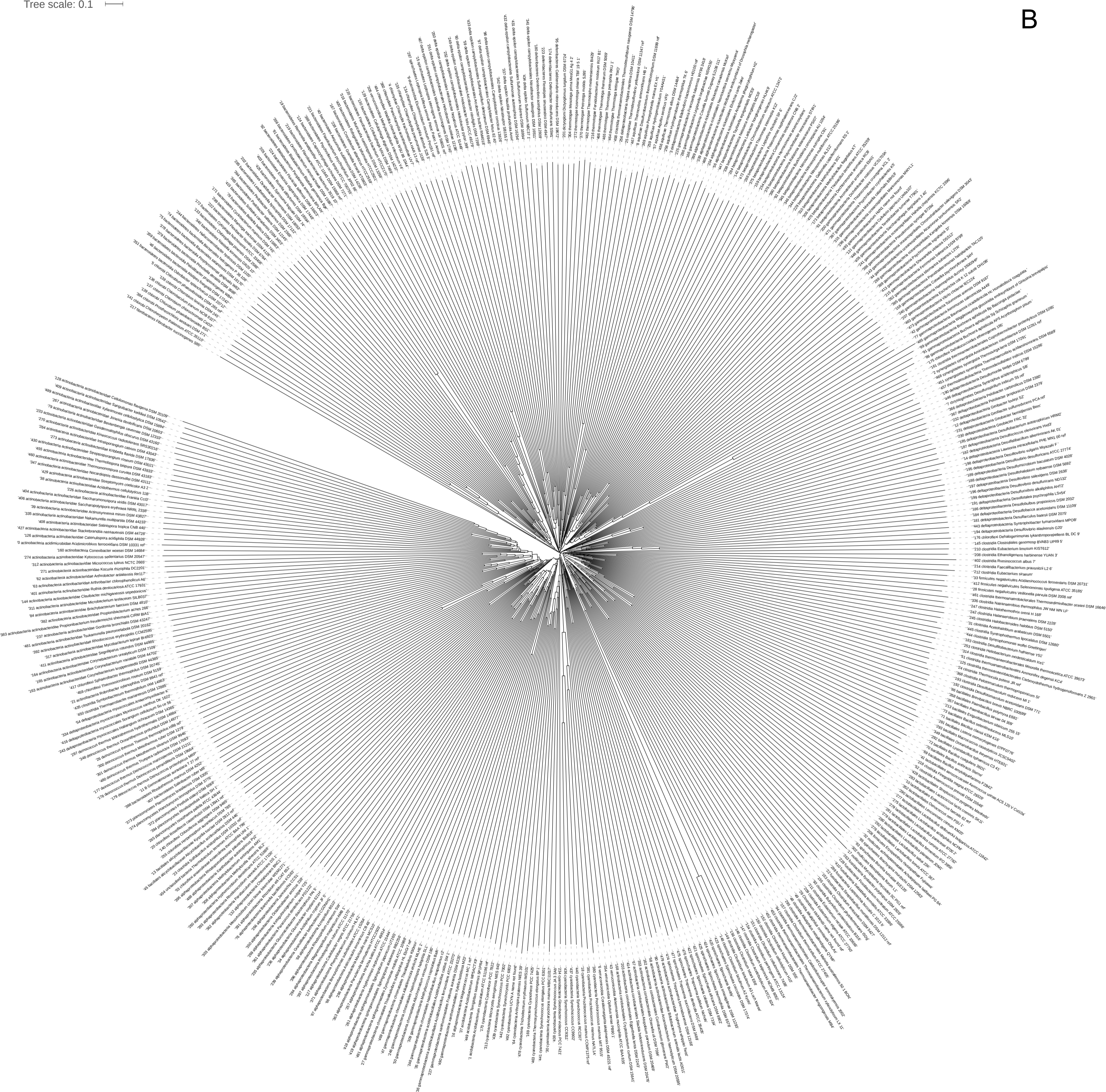

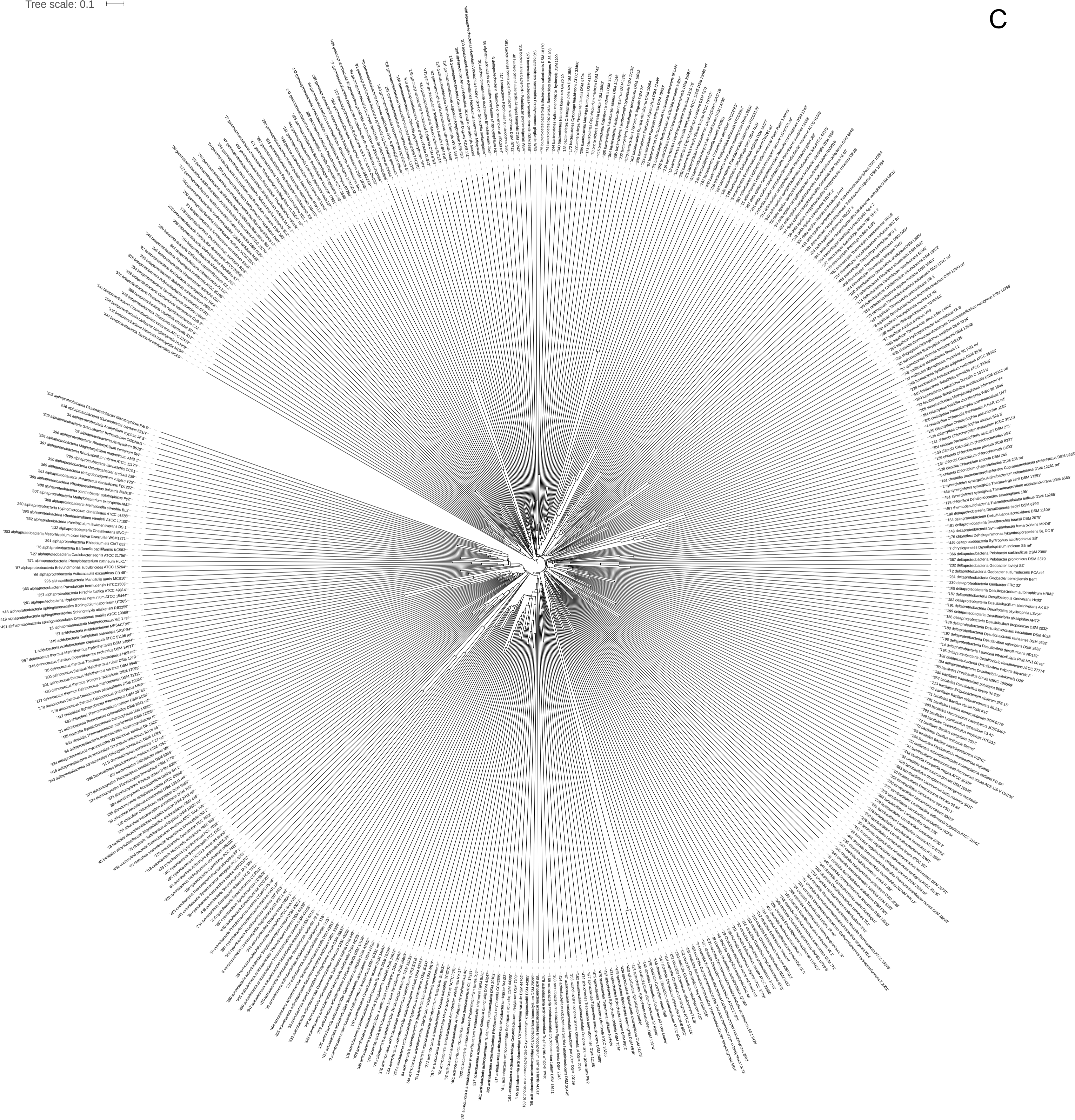

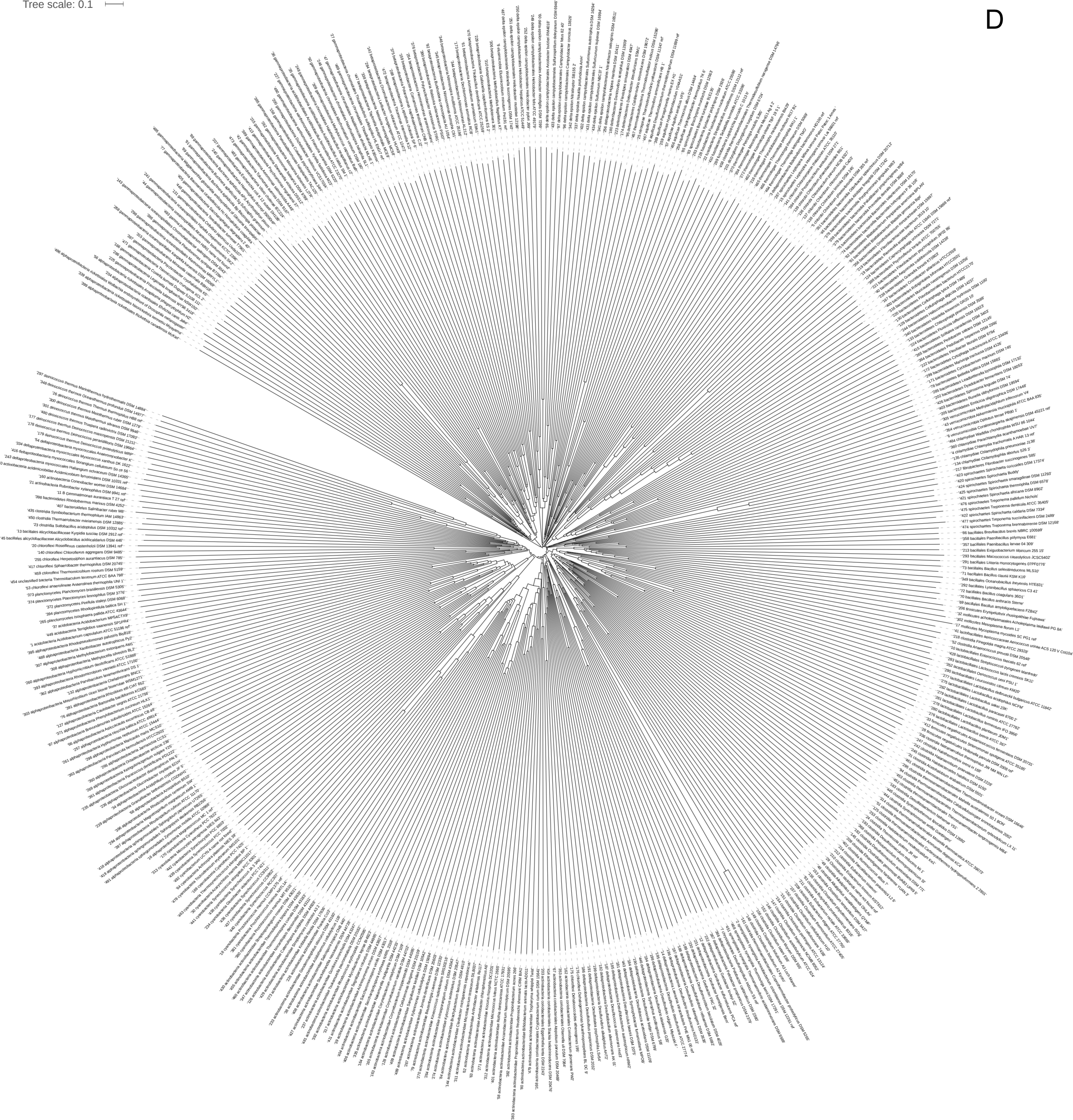

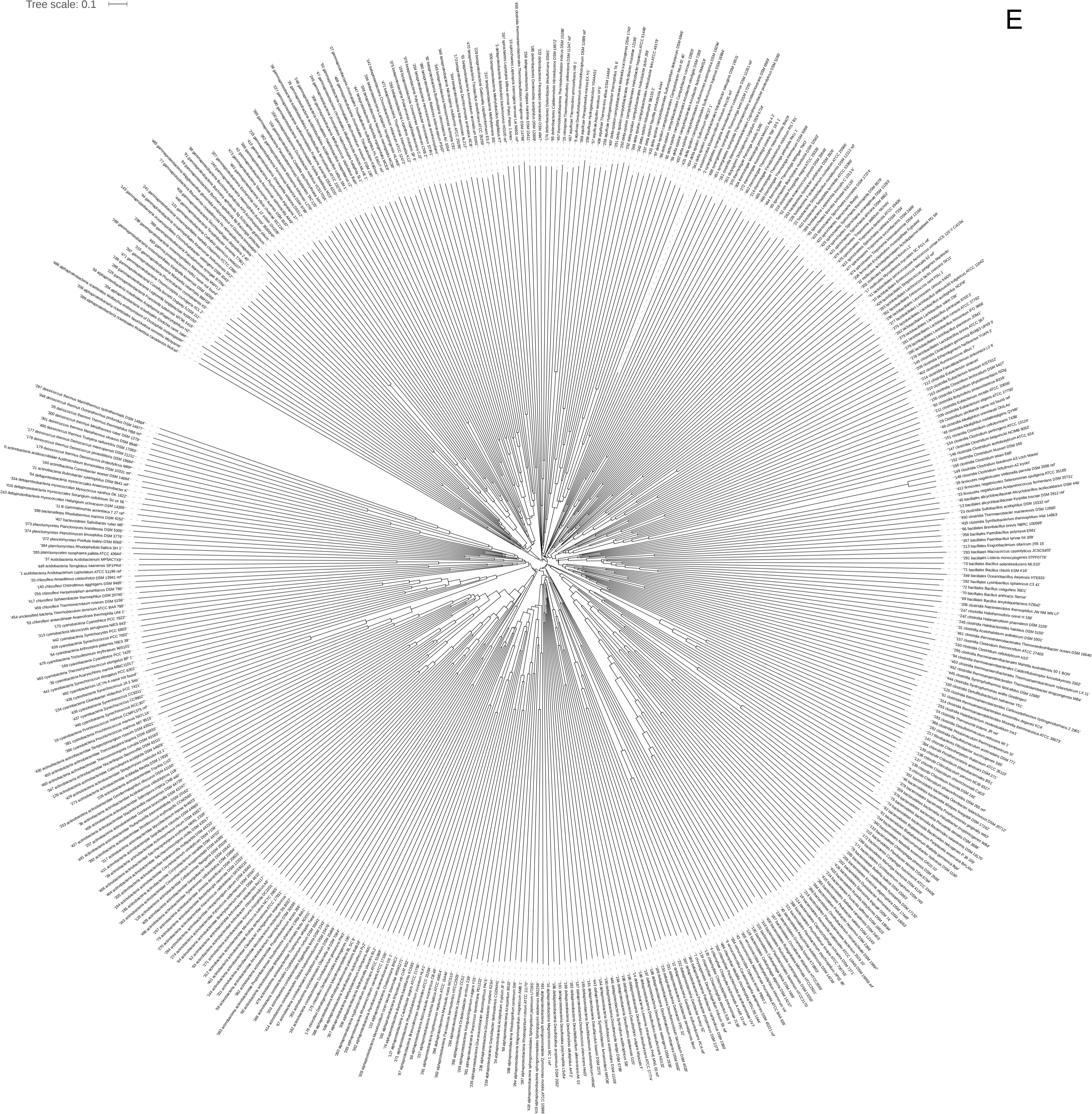

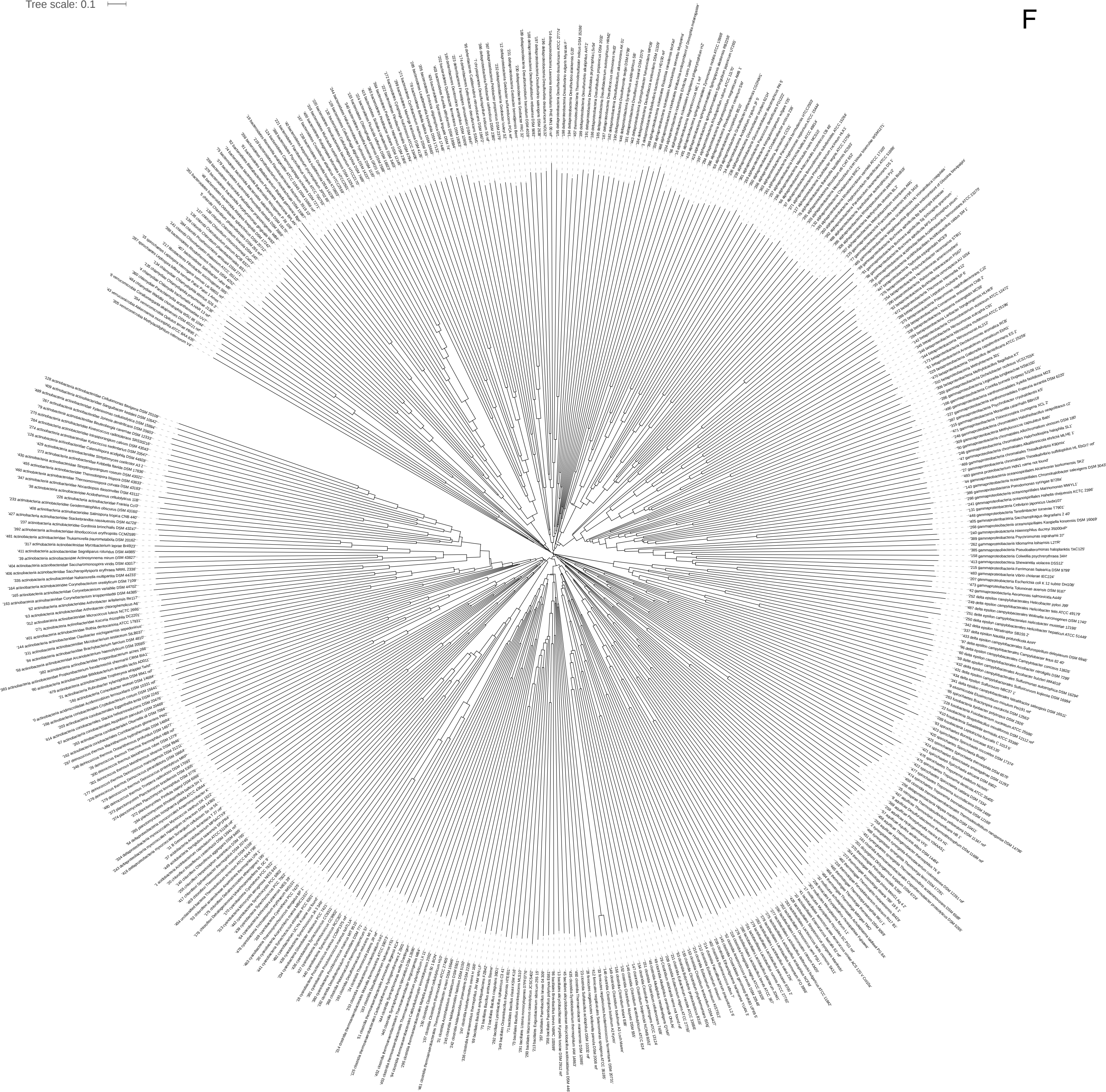

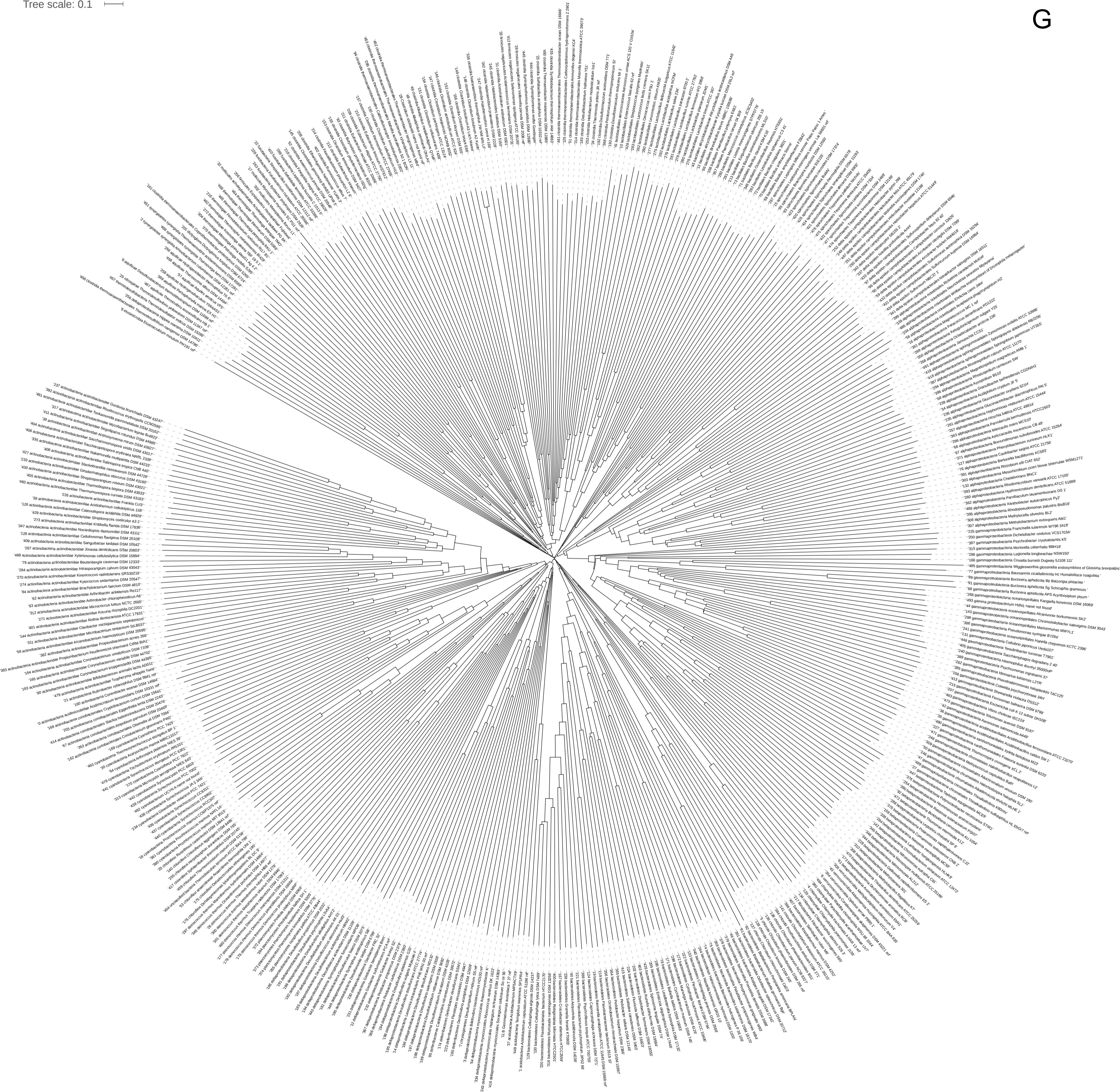

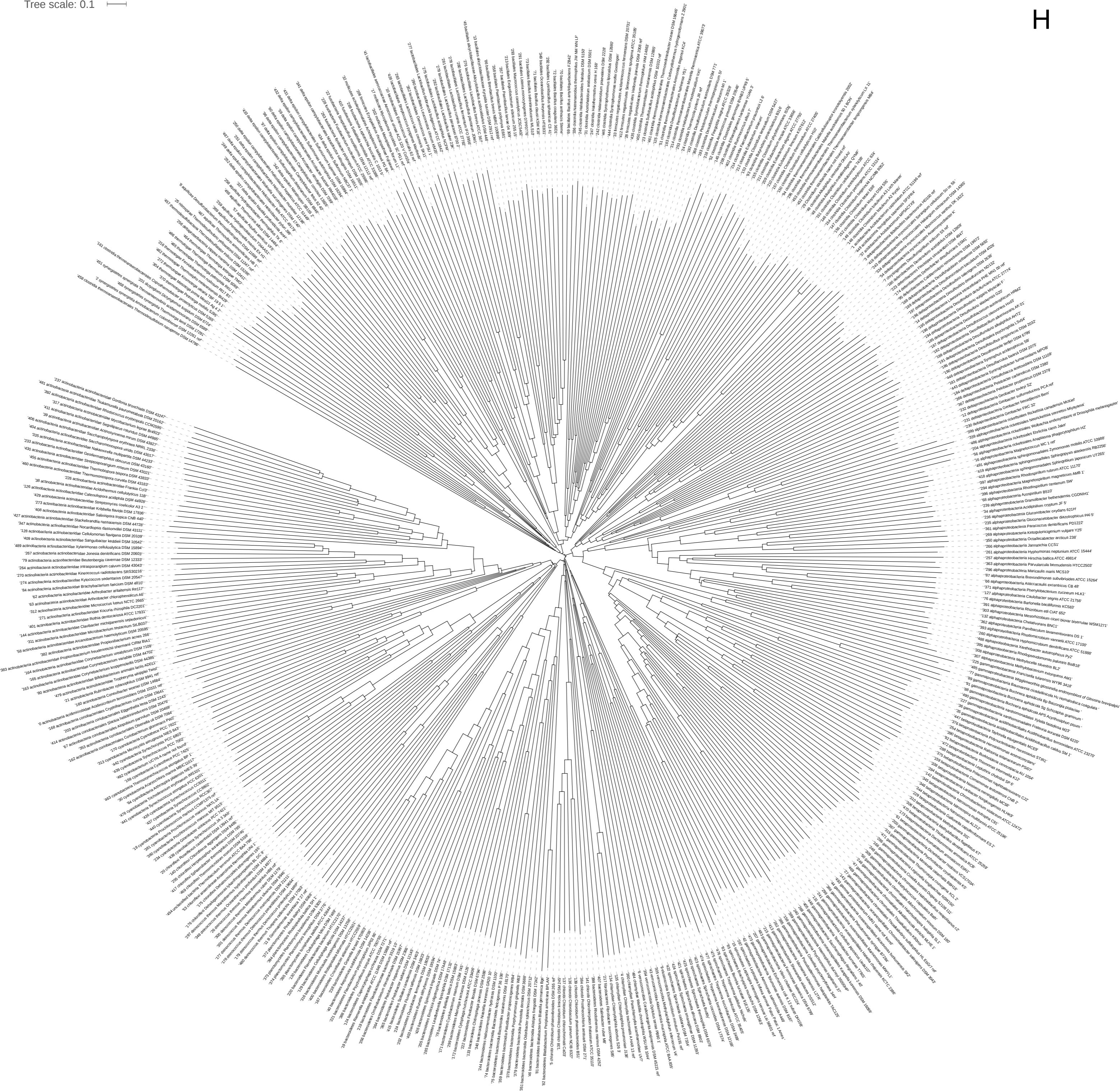

Supplement: S13 Fig — Trees rooted using ‘Root mid-point’ option in ITOL server. A) Raw ACS-tree for 495 bacteria. Unfiltered and unpruned. B) Raw ACS-tree for 445 bacteria. Unfiltered and pruned. C) ACS- tree for 445 bacteria. Filtered of mobile elements and pruned. D) ACS-tree for 445 bacteria. Filtered of mobile elements, pruned, and filtered by stability and conservation on o = 0. E) ACS-tree for 445 bacteria. Filtered of mobile elements, pruned, and filtered by stability and conservation on o = 1. F) ACS-tree for 445 bacteria. Filtered of mobile elements, pruned, and filtered by stability and conservation on o = 3. G) ACS-tree for 445 bacteria. Filtered of mobile elements, pruned, and filtered by stability and conservation on o = 5. H) ACS-tree for 445 bacteria. Filtered of mobile elements, pruned, and filtered by stability and conservation on o = 7. (PDF) [file pcbi.1004985.s013.pdf]

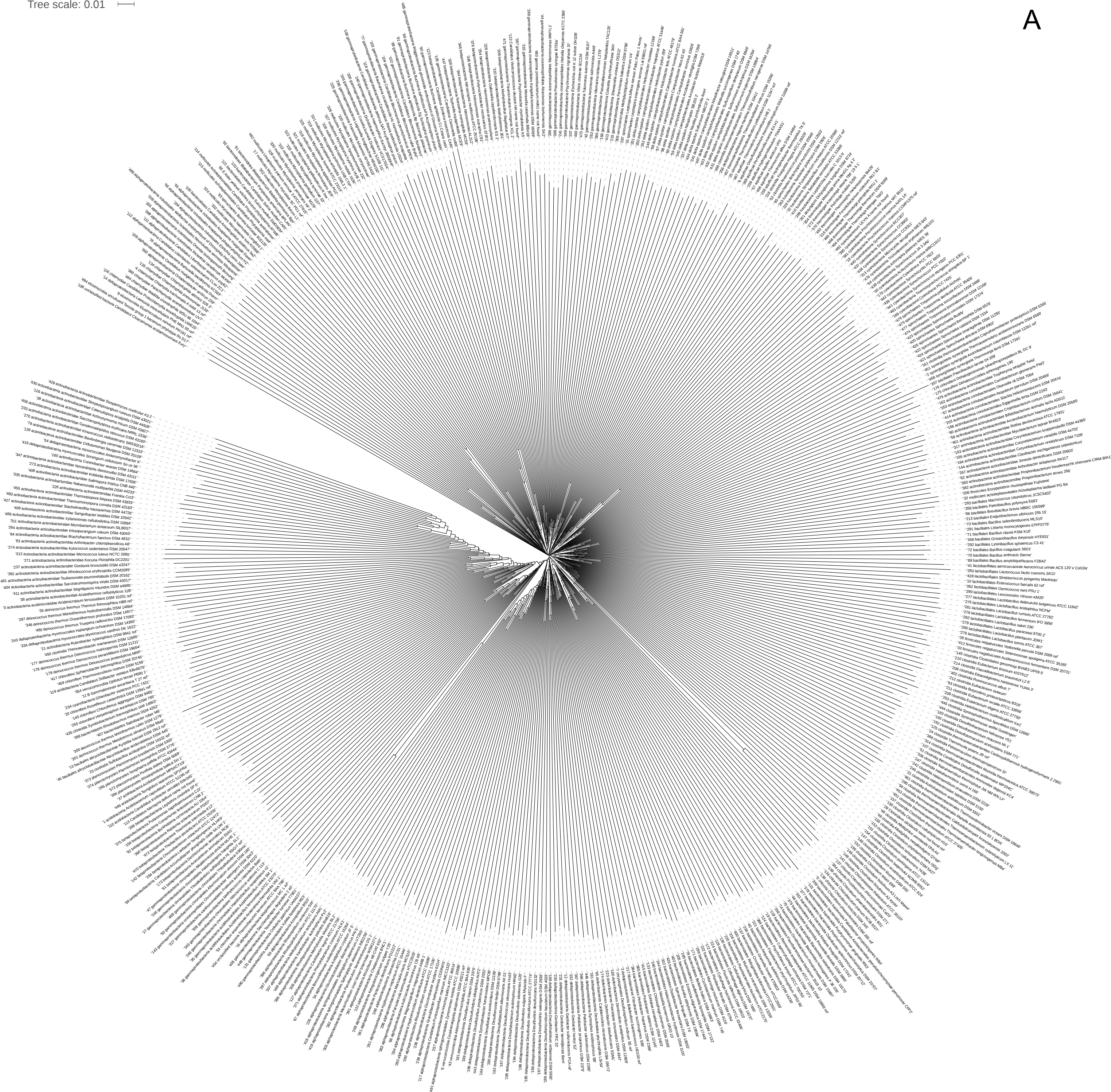

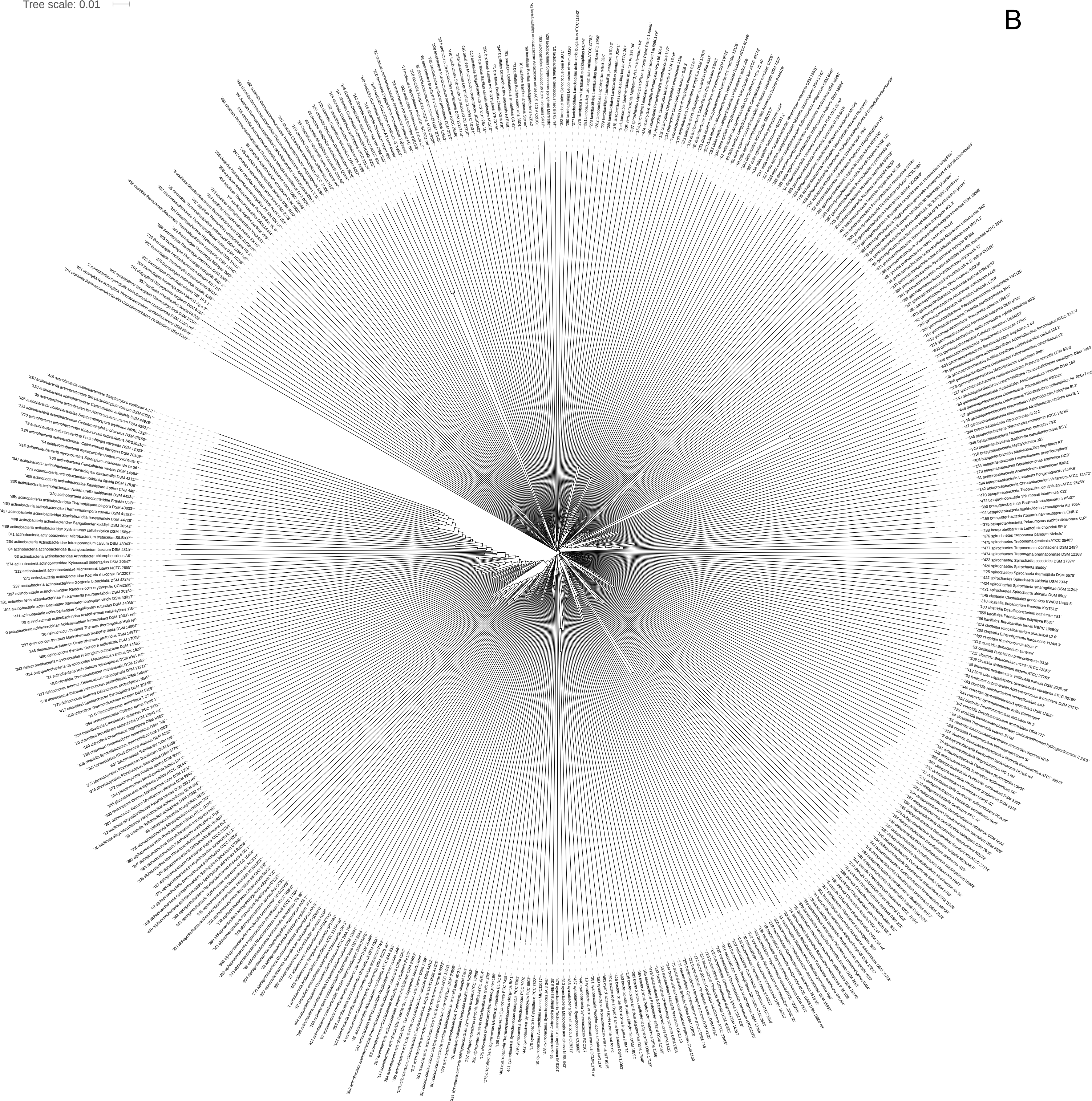

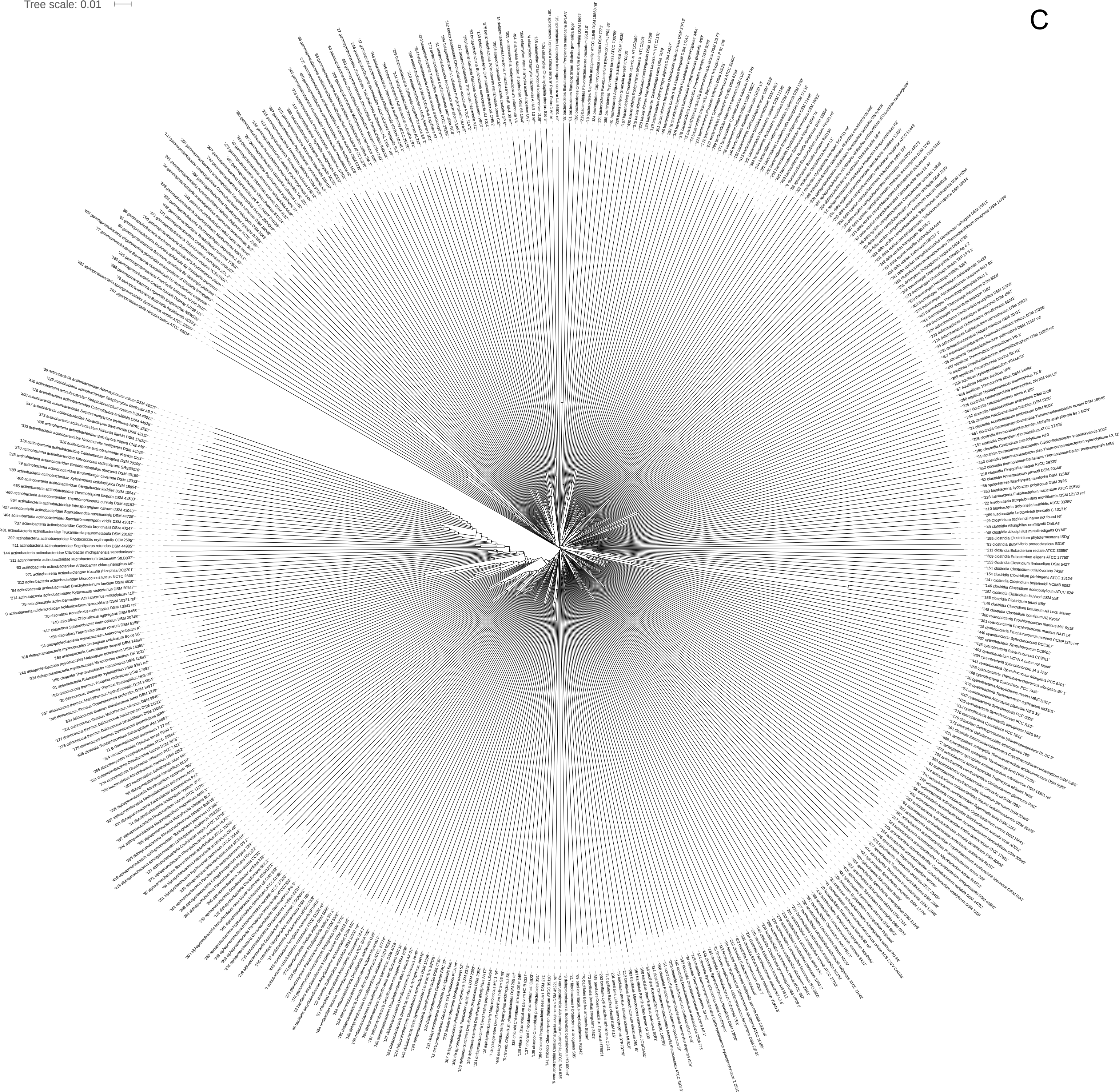



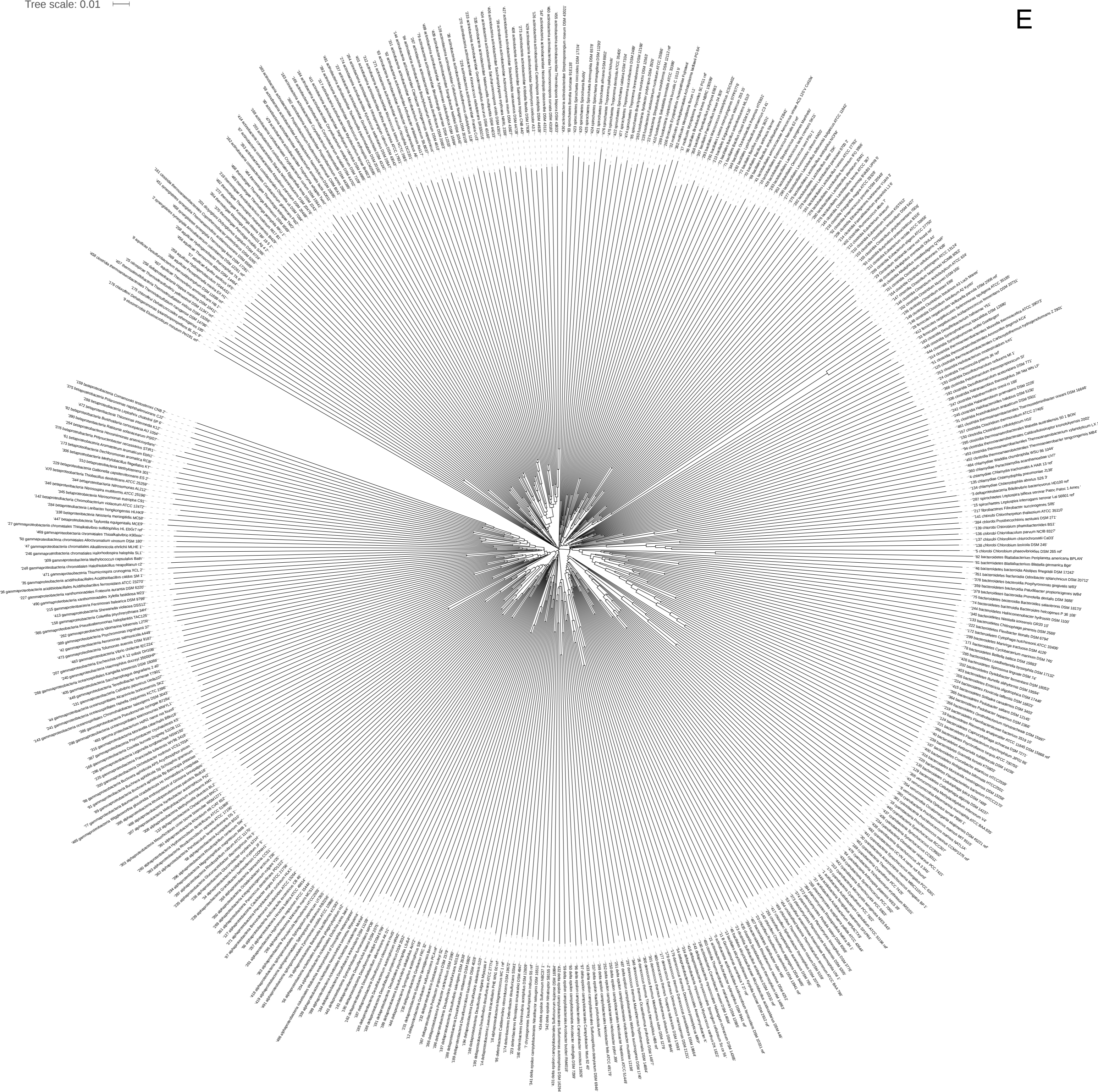

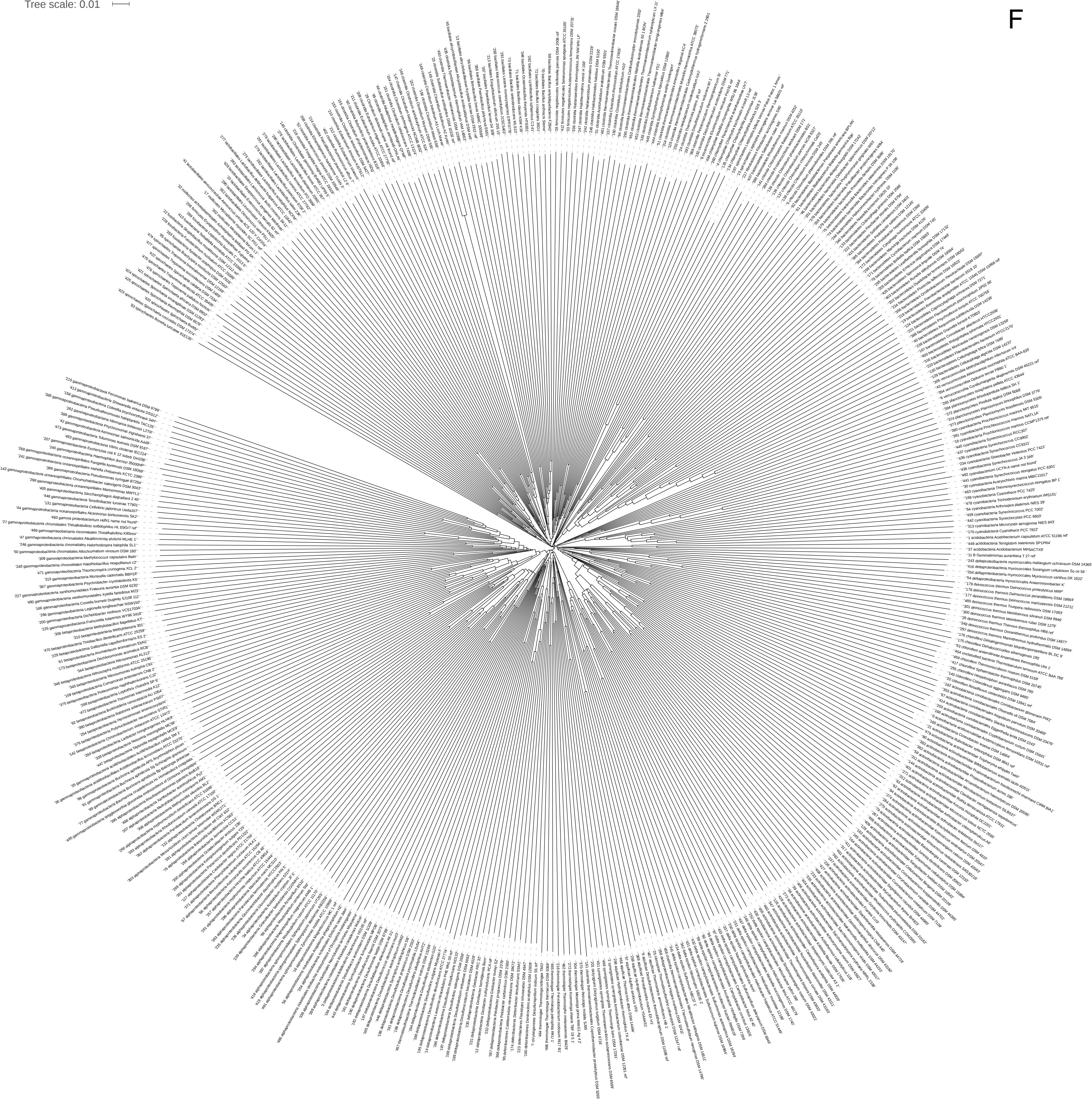

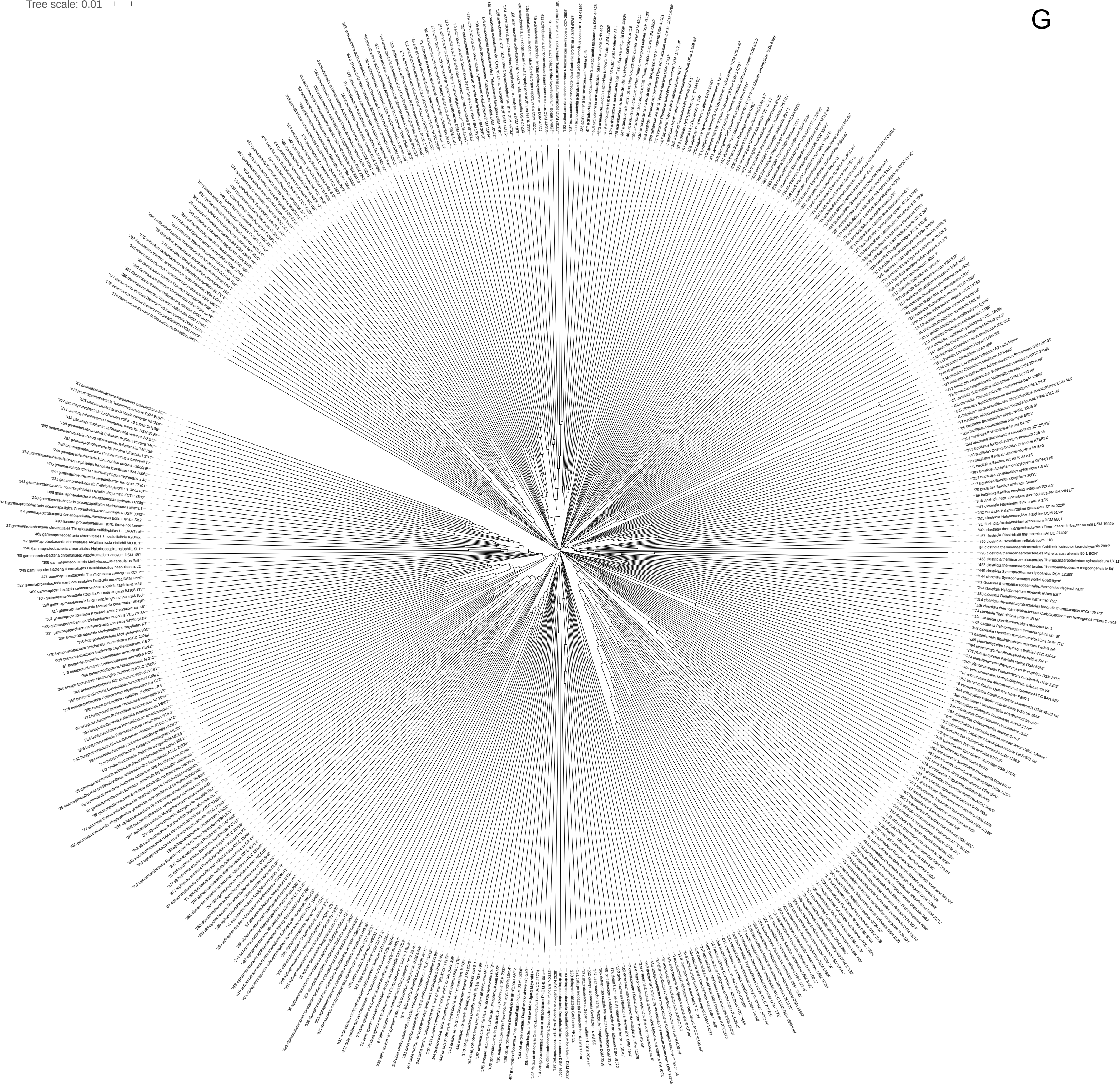

Supplement: S14 Fig — Trees rooted using ‘Root mid-point’ option in ITOL server. A) CVTree on raw 495 bacteria. Unfiltered and unpruned. B) CVTree on raw 445 bacteria. Unfiltered and pruned. C) CVTree on 445 bacteria. Filtered of mobile elements and pruned. D) CVTree on 445 bacteria. Filtered of mobile elements, pruned, and filtered by stability and conservation on o = 0. E) CVTree on 445 bacteria. Filtered of mobile elements, pruned, and filtered by stability and conservation on o = 1. F) CVTree on 445 bacteria. Filtered of mobile elements, pruned, and filtered by stability and conservation on o = 3. G) CVTree on 445 bacteria. Filtered of mobile elements, pruned, and filtered by stability and conservation on o = 5. H) CVTree on 445 bacteria. Filtered of mobile elements, pruned, and filtered by stability and conservation on o = 7. (PDF) [file pcbi.1004985.s014.pdf]

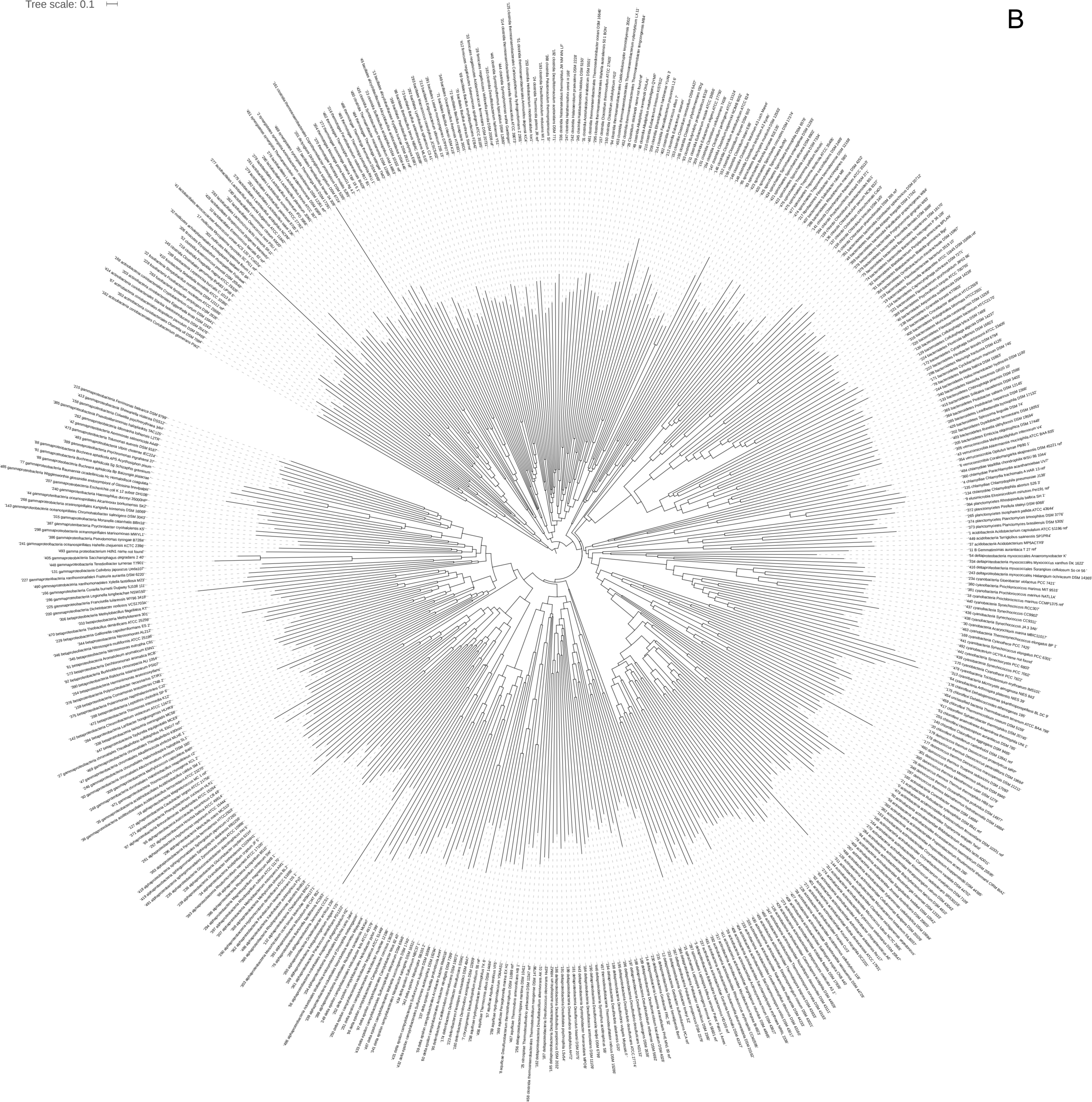

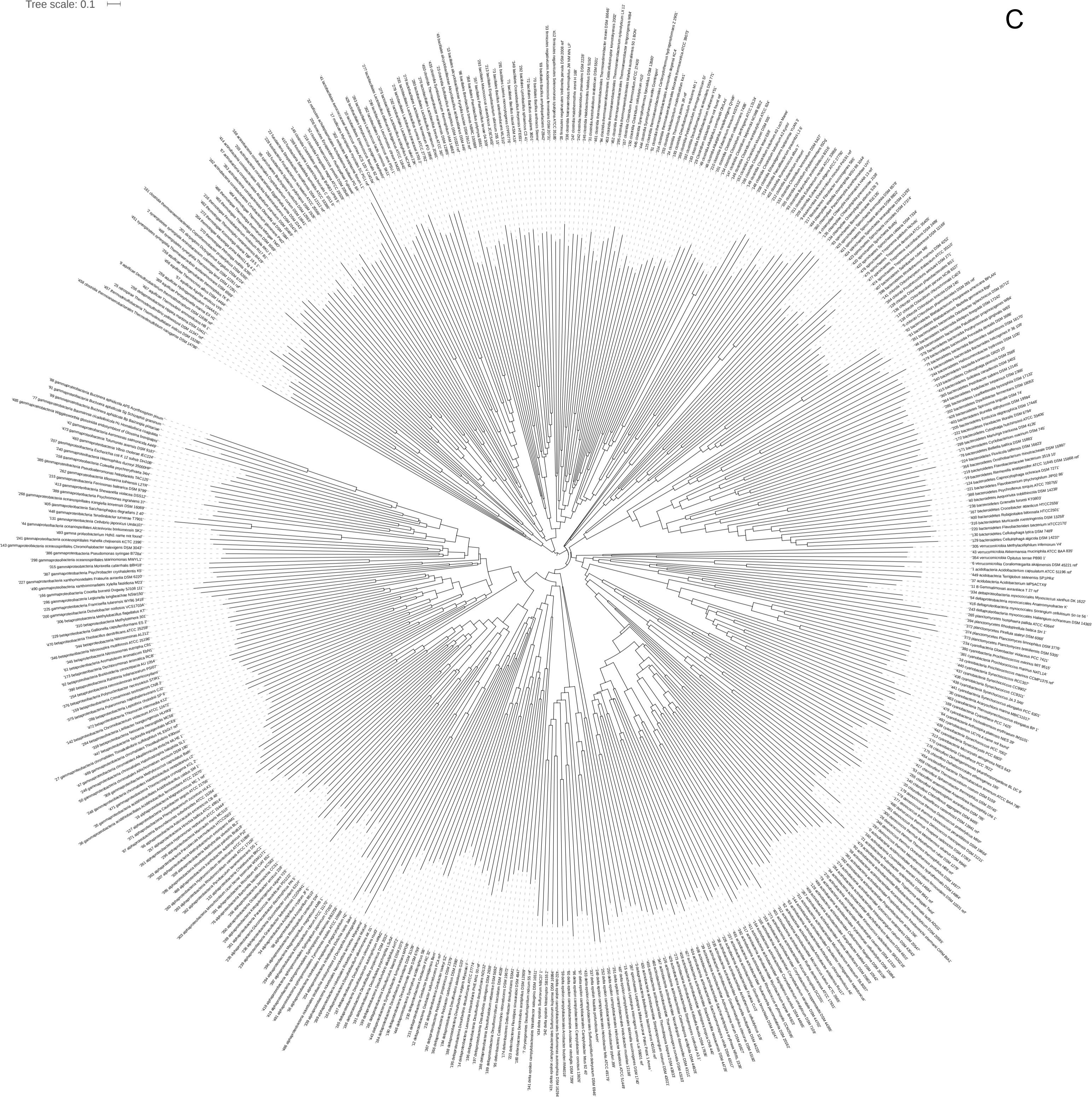

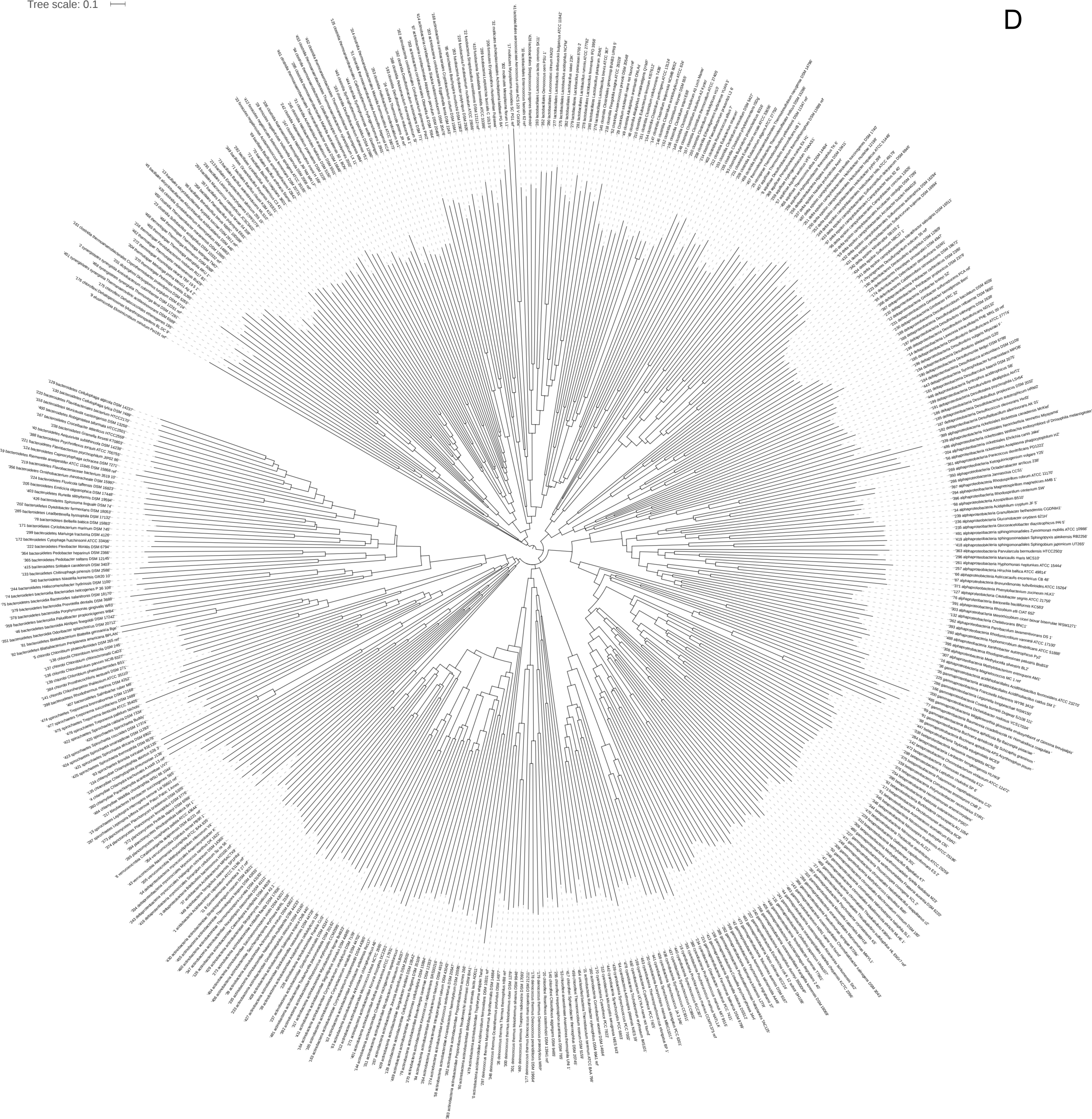

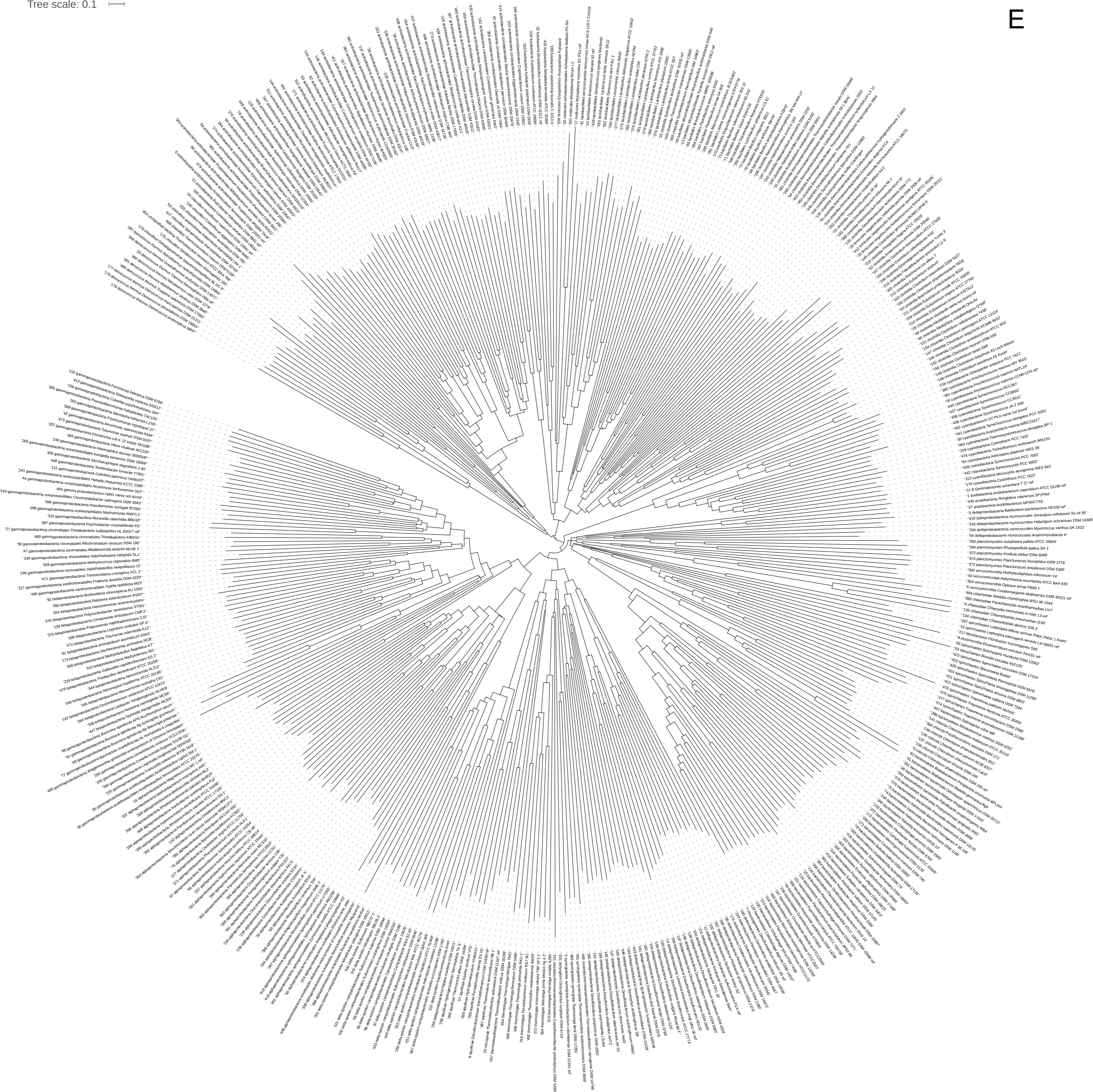

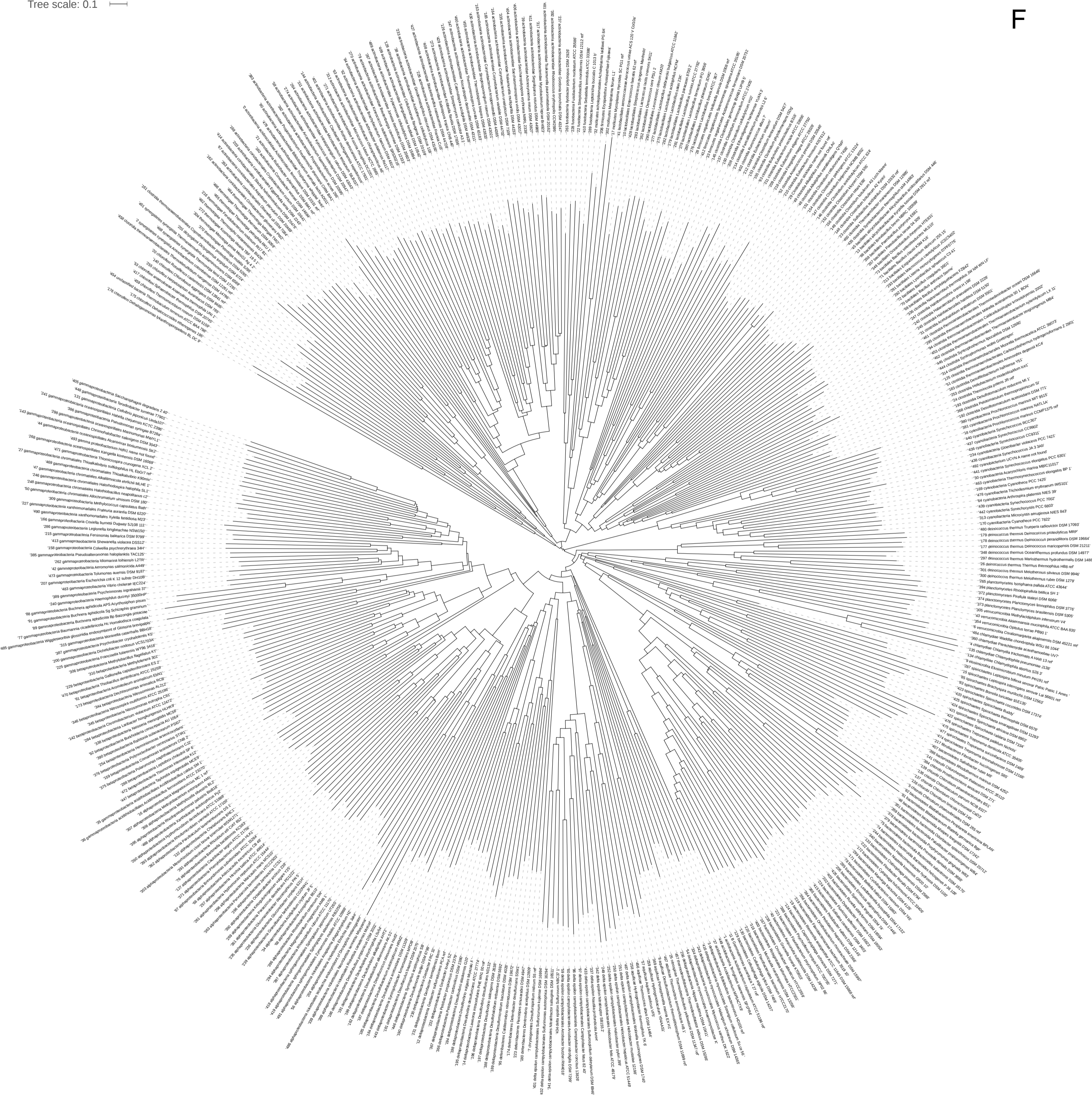

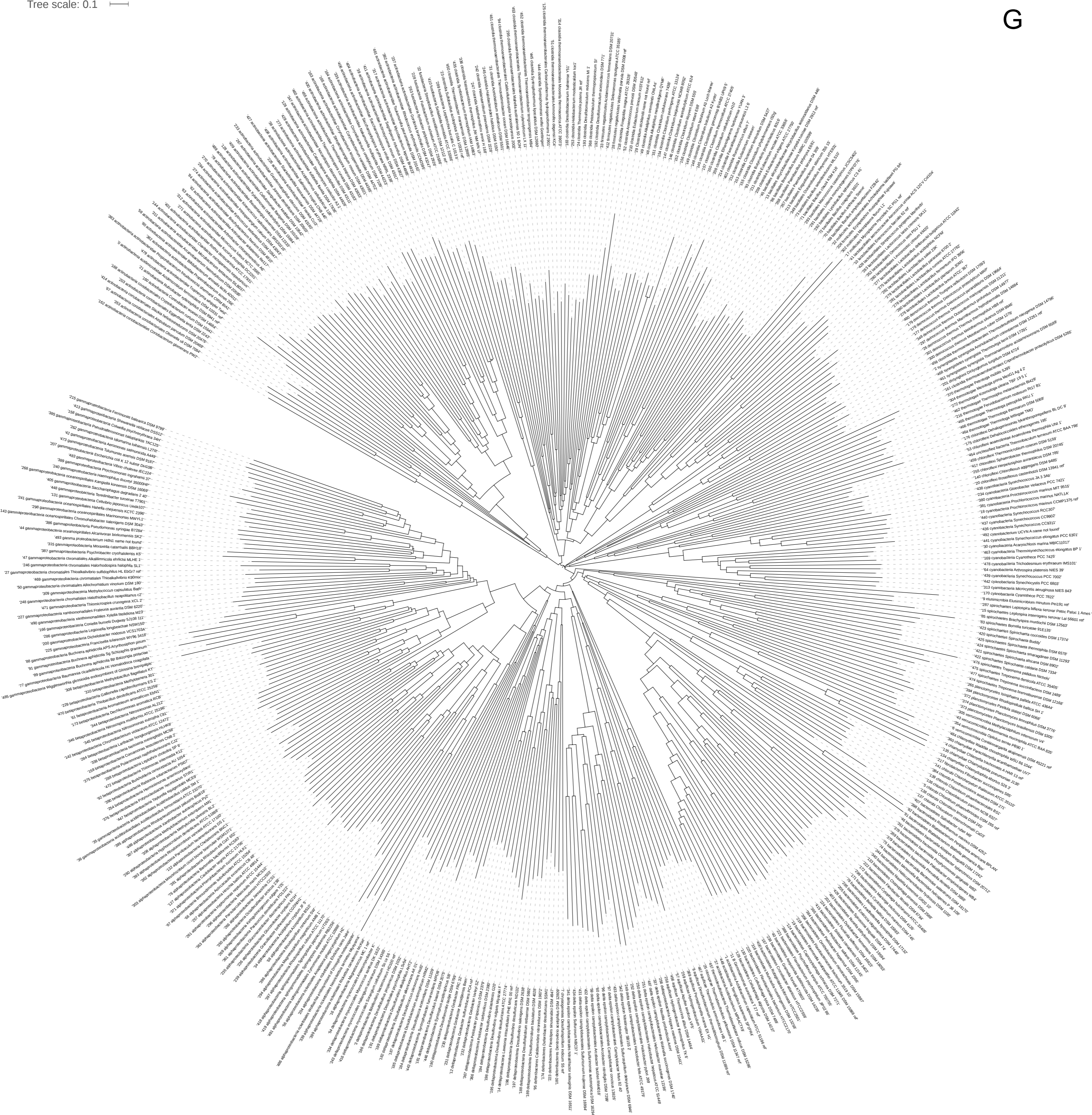

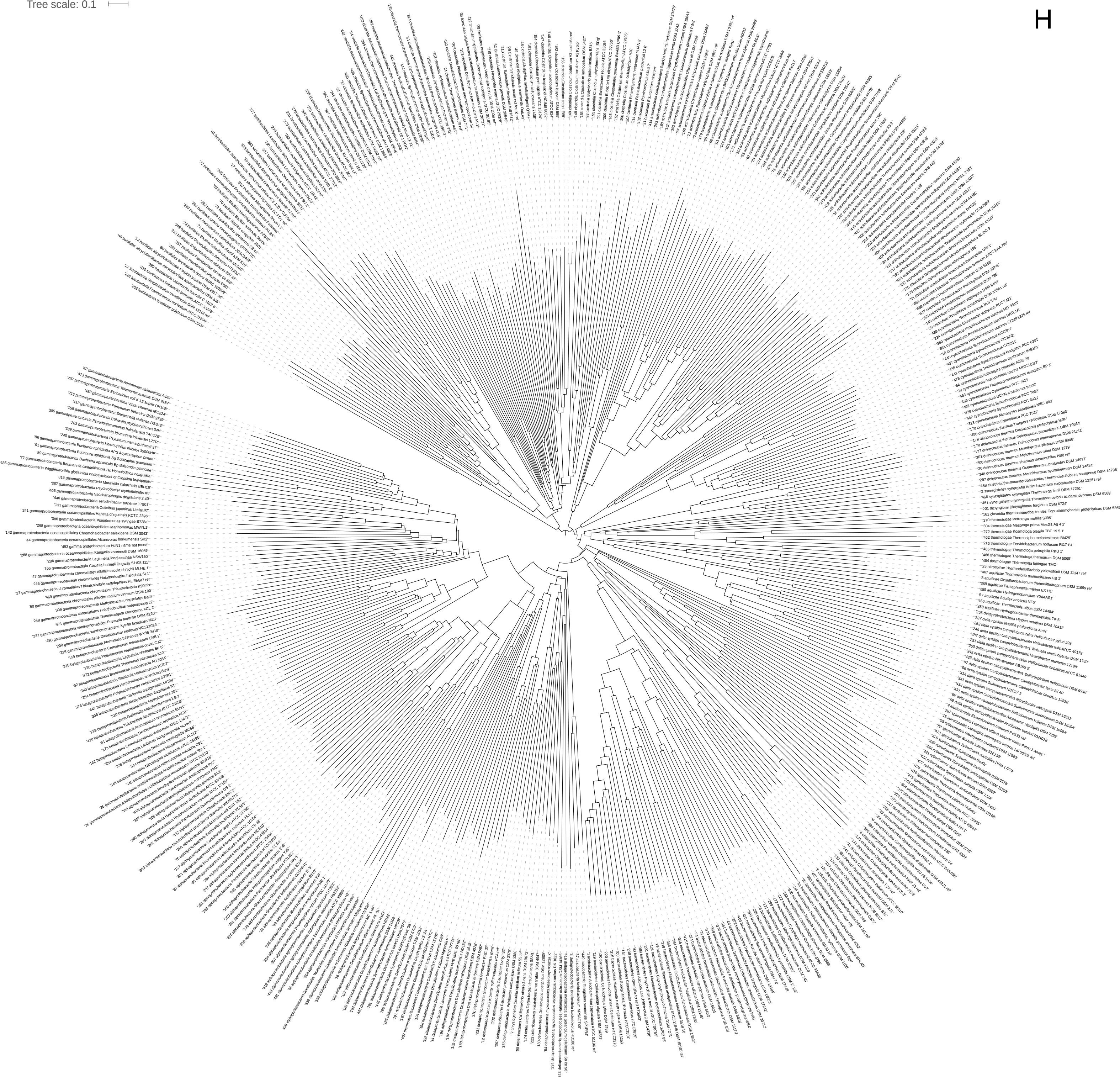

Supplement: S15 Fig — Trees rooted using ‘Root mid-point’ option in ITOL server. A) D2 on raw 495 bacteria. Unfiltered and unpruned. B) D2 on raw 445 bacteria. Unfiltered and pruned. C) D2 on 445 bacteria. Filtered of mobile elements and pruned. D) D2 on 445 bacteria. Filtered of mobile elements, pruned, and filtered by stability and conservation on o = 0. E) D2 on 445 bacteria. Filtered of mobile elements, pruned, and filtered by stability and conservation on o = 1. F) D2 on 445 bacteria. Filtered of mobile elements, pruned, and filtered by stability and conservation on o = 3. G) D2 on 445 bacteria. Filtered of mobile elements, pruned, and filtered by stability and conservation on o = 5. H) D2 on 445 bacteria. Filtered of mobile elements, pruned, and filtered by stability and conservation on o = 7. (PDF) [file pcbi.1004985.s015.pdf]

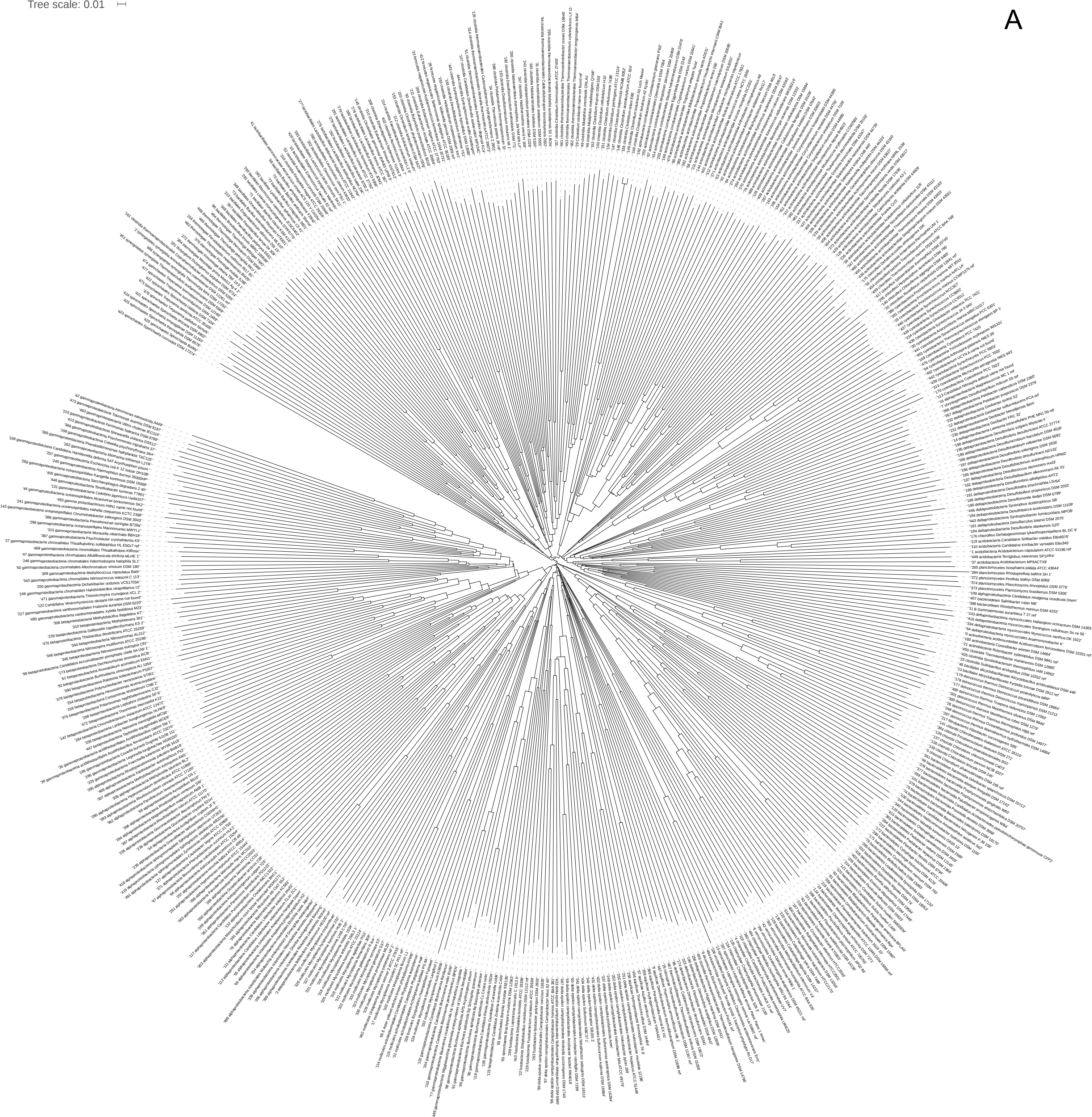

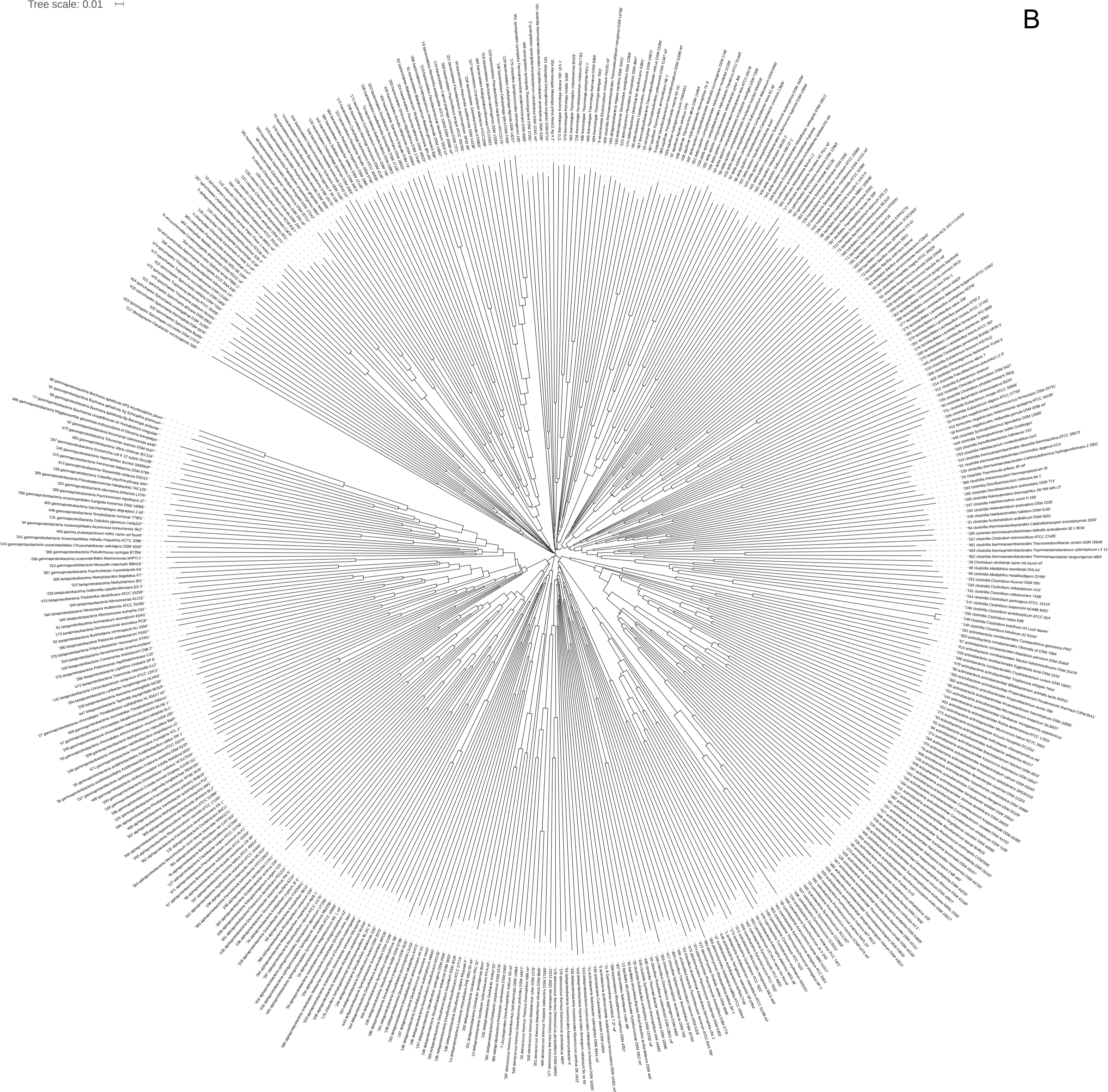

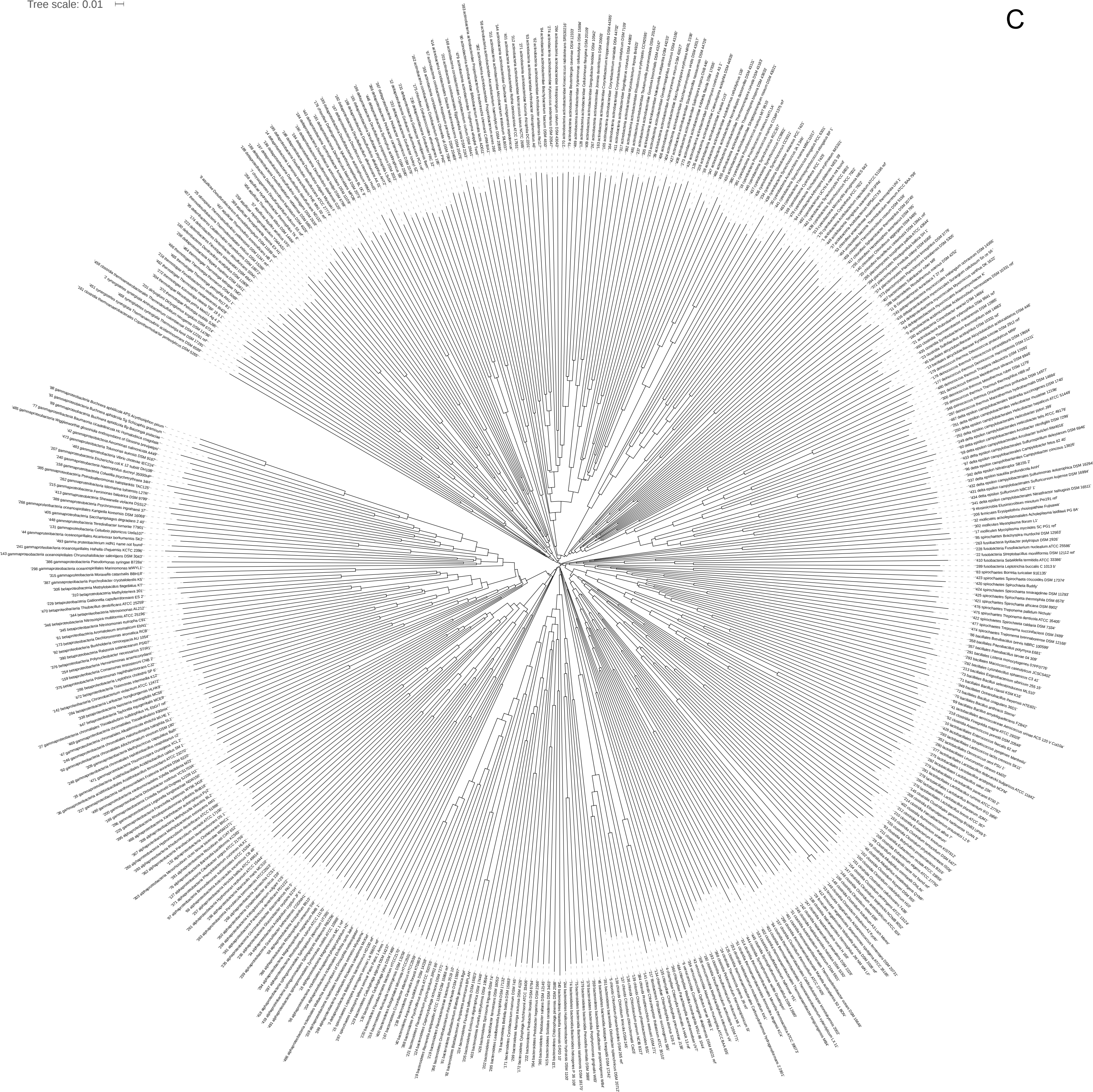

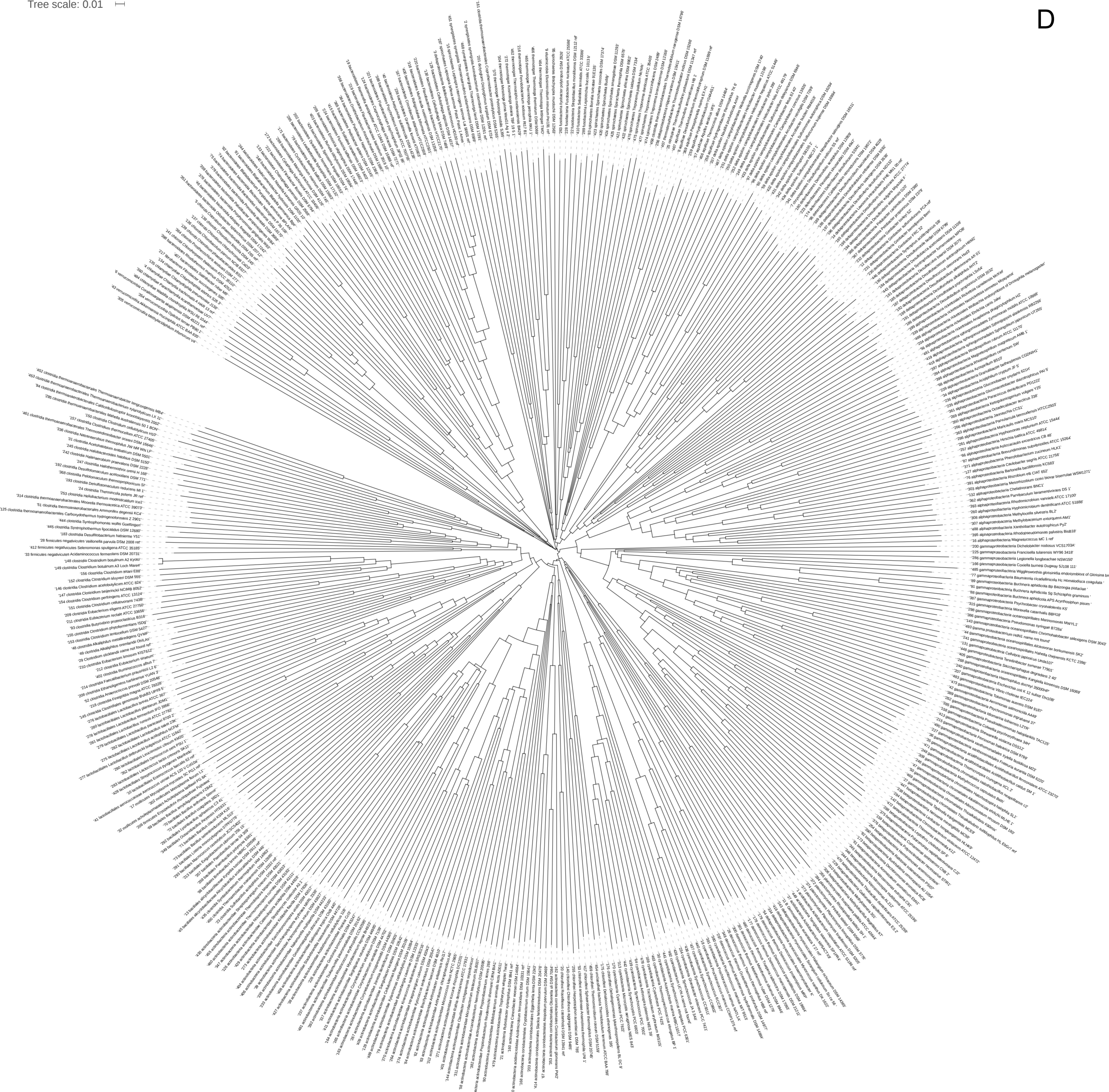

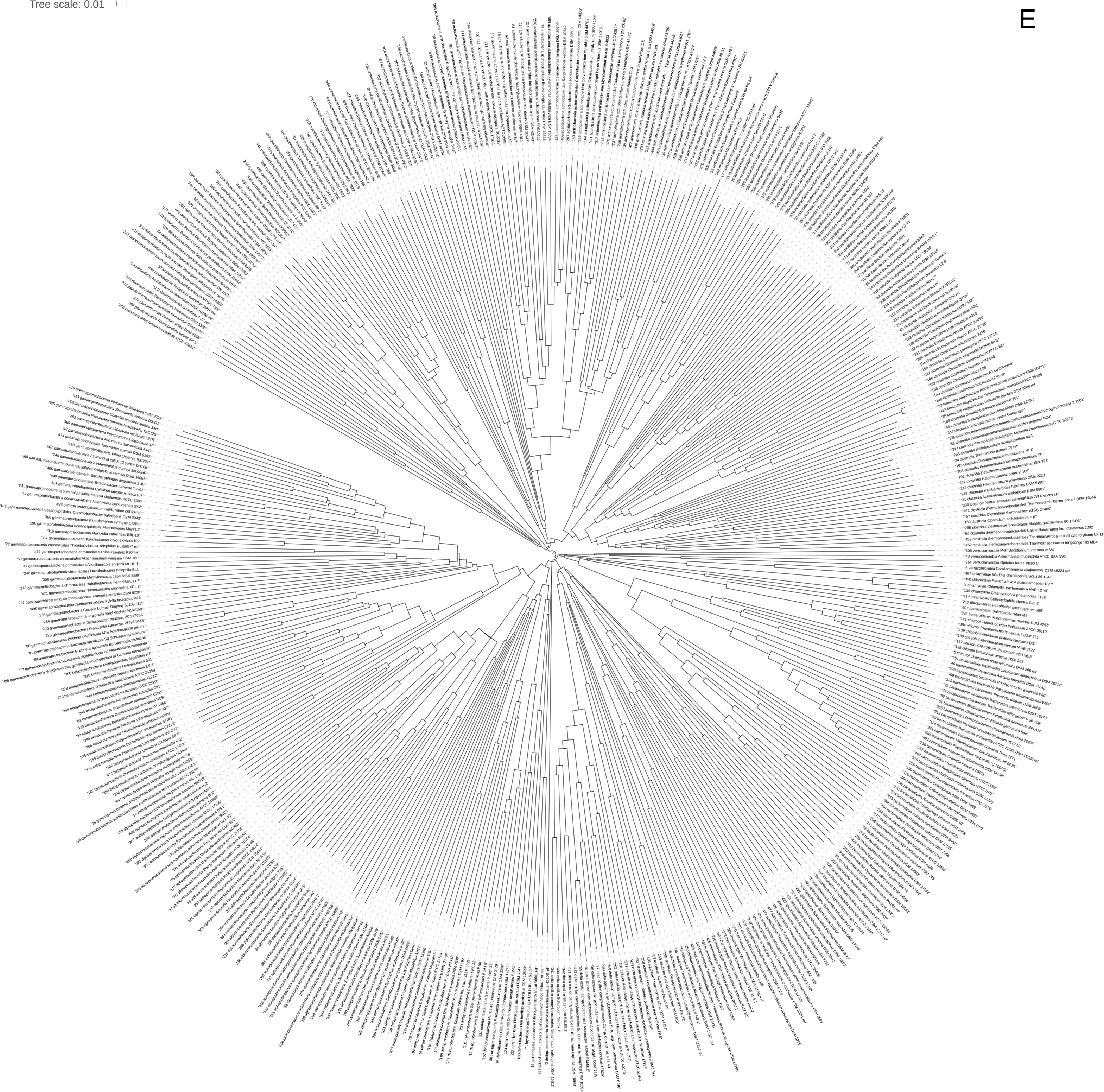

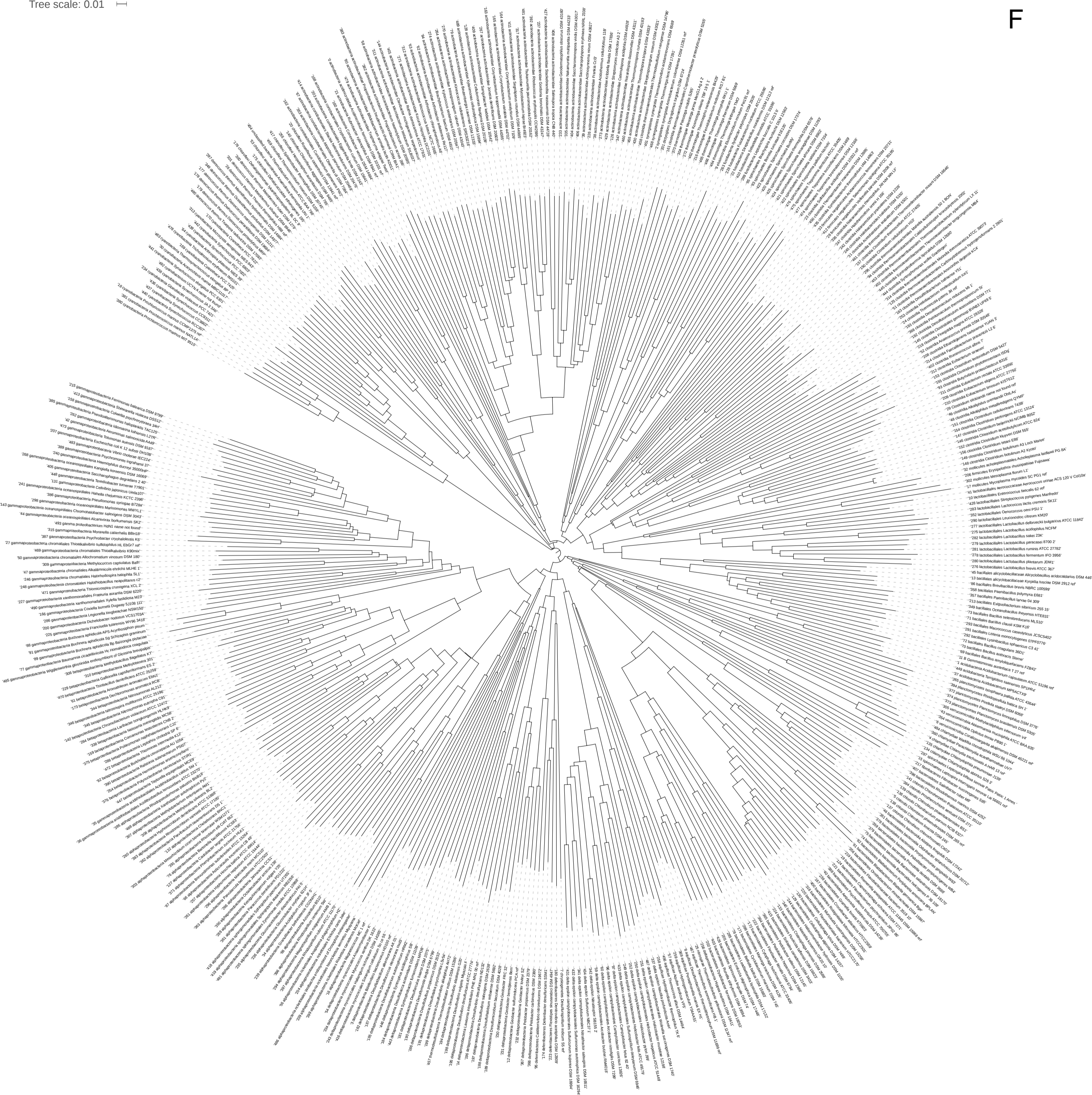

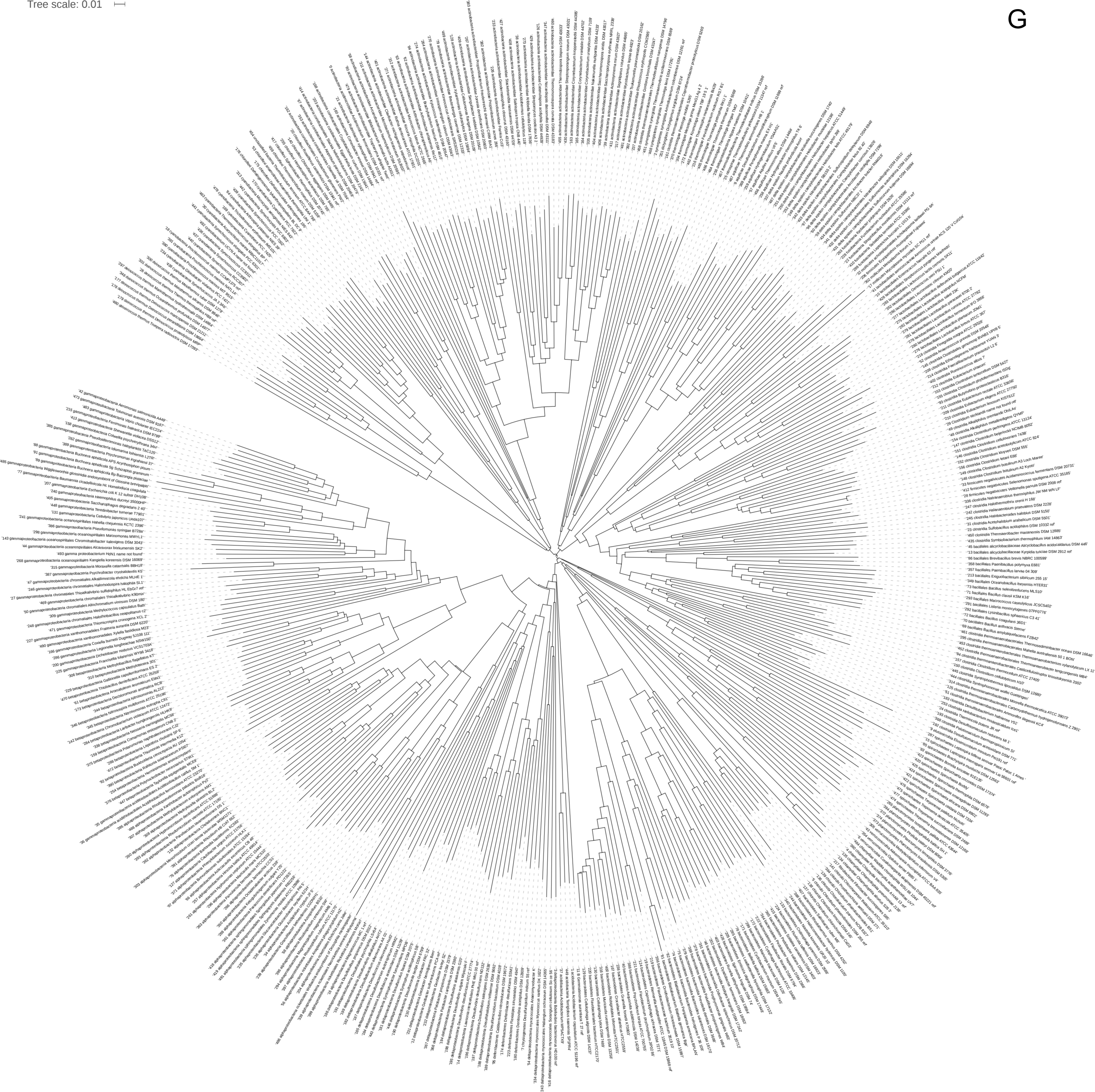

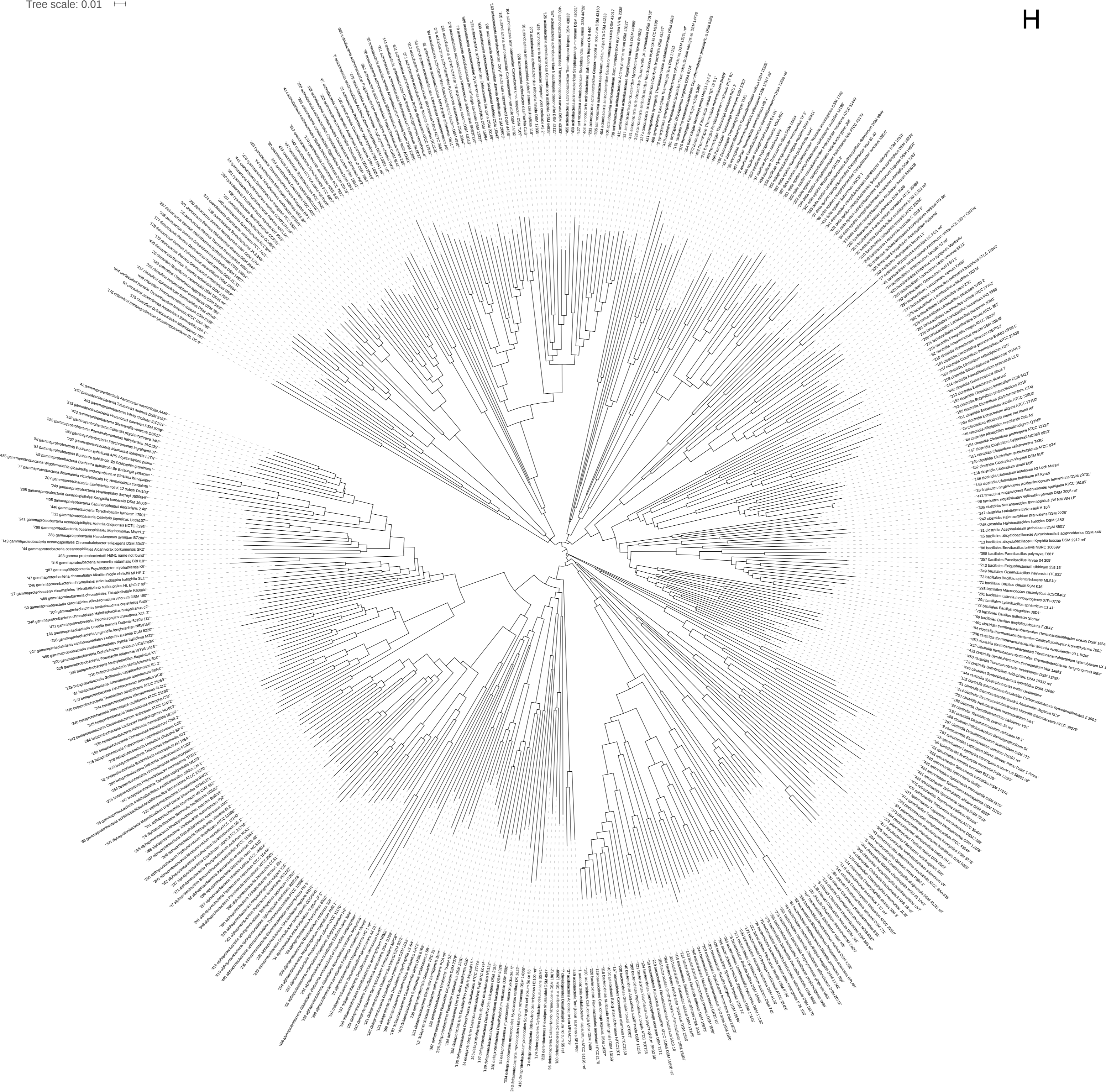

Supplement: S16 Fig — Trees rooted using ‘Root mid-point’ option in ITOL server. A) kmacs on raw 495 bacteria. Unfiltered and unpruned. B) kmacs on raw 445 bacteria. Unfiltered and pruned. C) kmacs on 445 bacteria. Filtered of mobile elements and pruned. D) kmacs on 445 bacteria. Filtered of mobile elements, pruned, and filtered by stability and conservation on o = 0. E) kmacs on 445 bacteria. Filtered of mobile elements, pruned, and filtered by stability and conservation on o = 1. F) kmacs on 445 bacteria. Filtered of mobile elements, pruned, and filtered by stability and conservation on o = 3. G) kmacs on 445 bacteria. Filtered of mobile elements, pruned, and filtered by stability and conservation on o = 5. H) kmacs on 445 bacteria. Filtered of mobile elements, pruned, and filtered by stability and conservation on o = 7. (PDF) [file pcbi.1004985.s016.pdf]

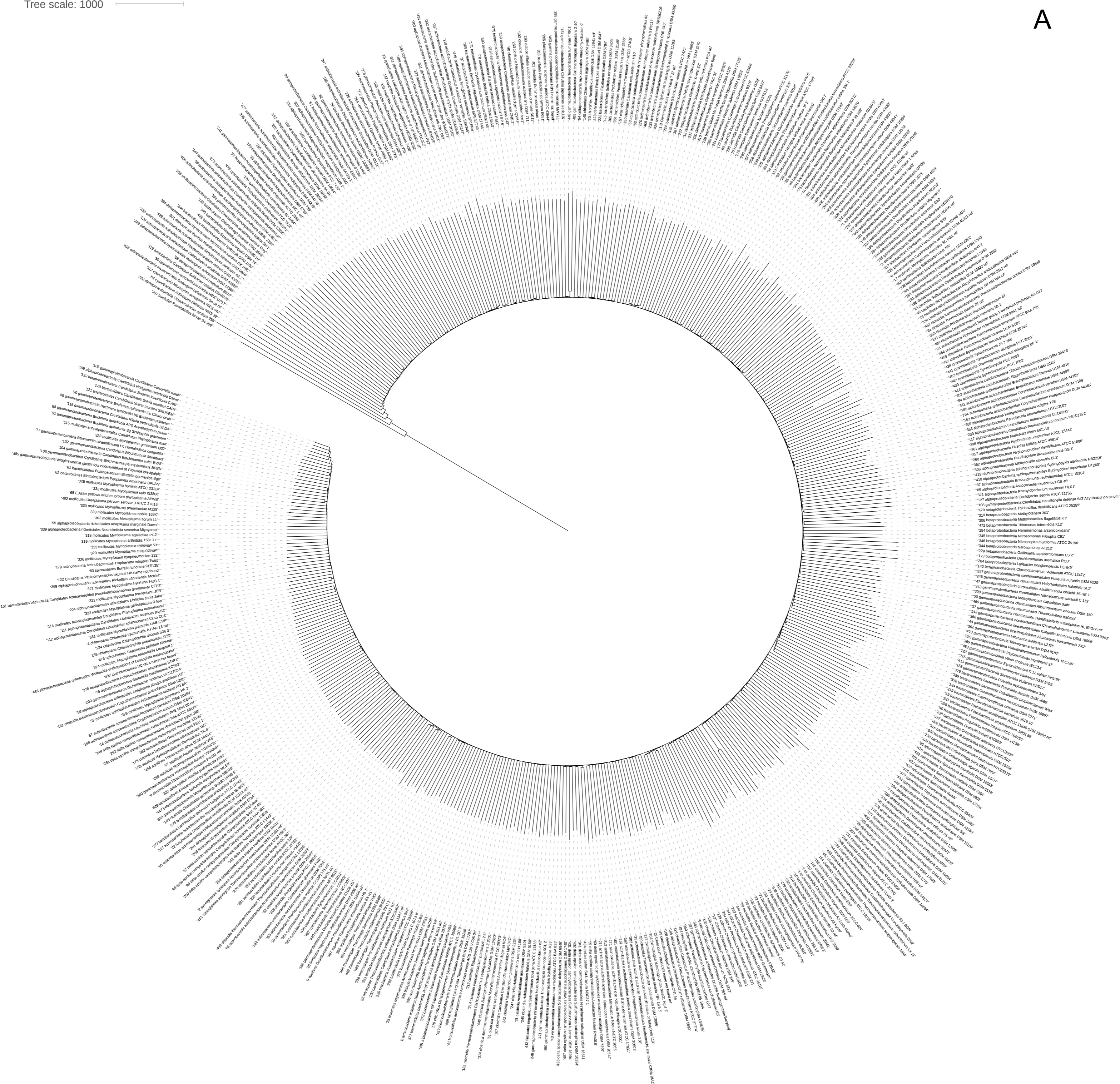

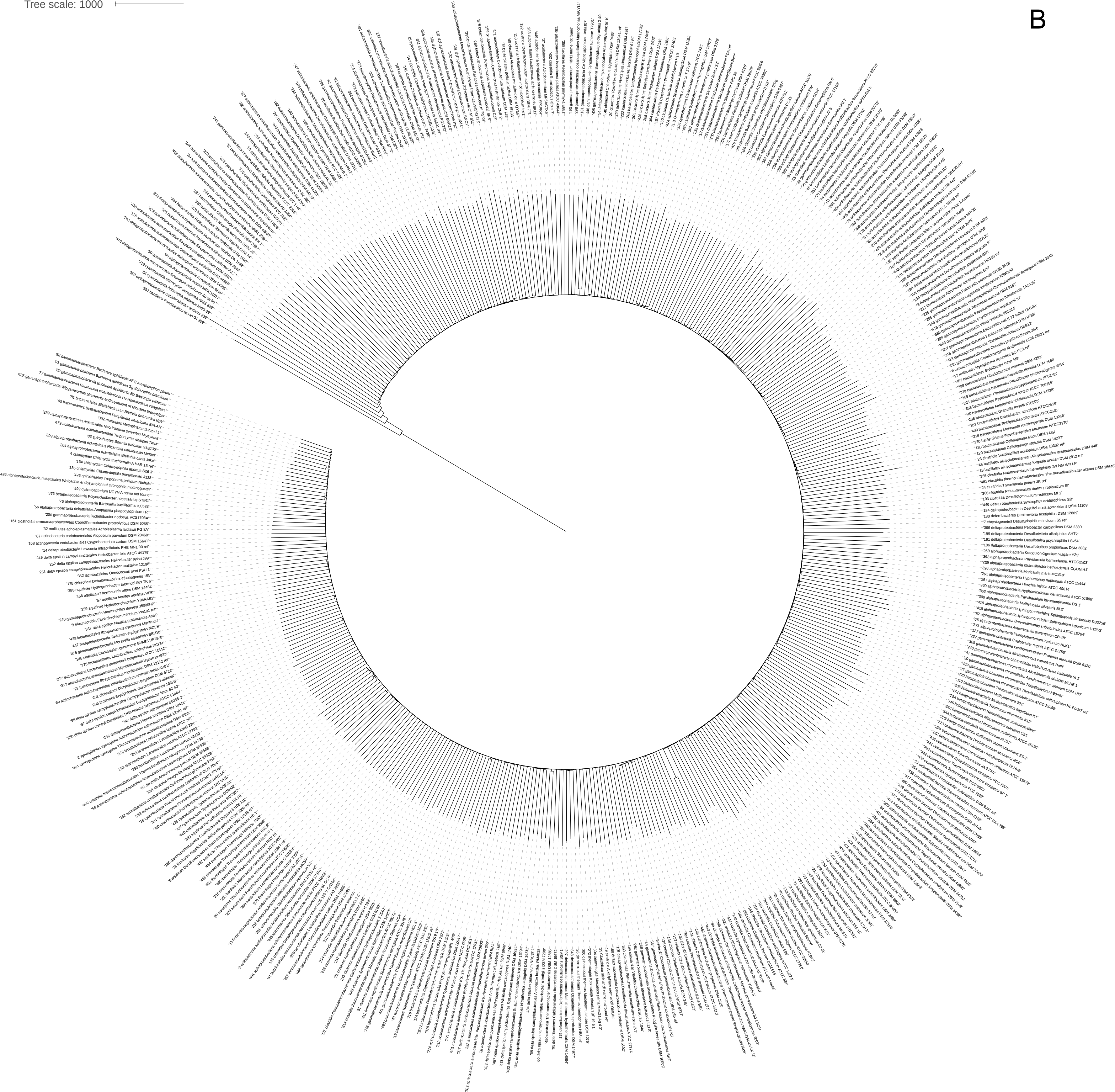

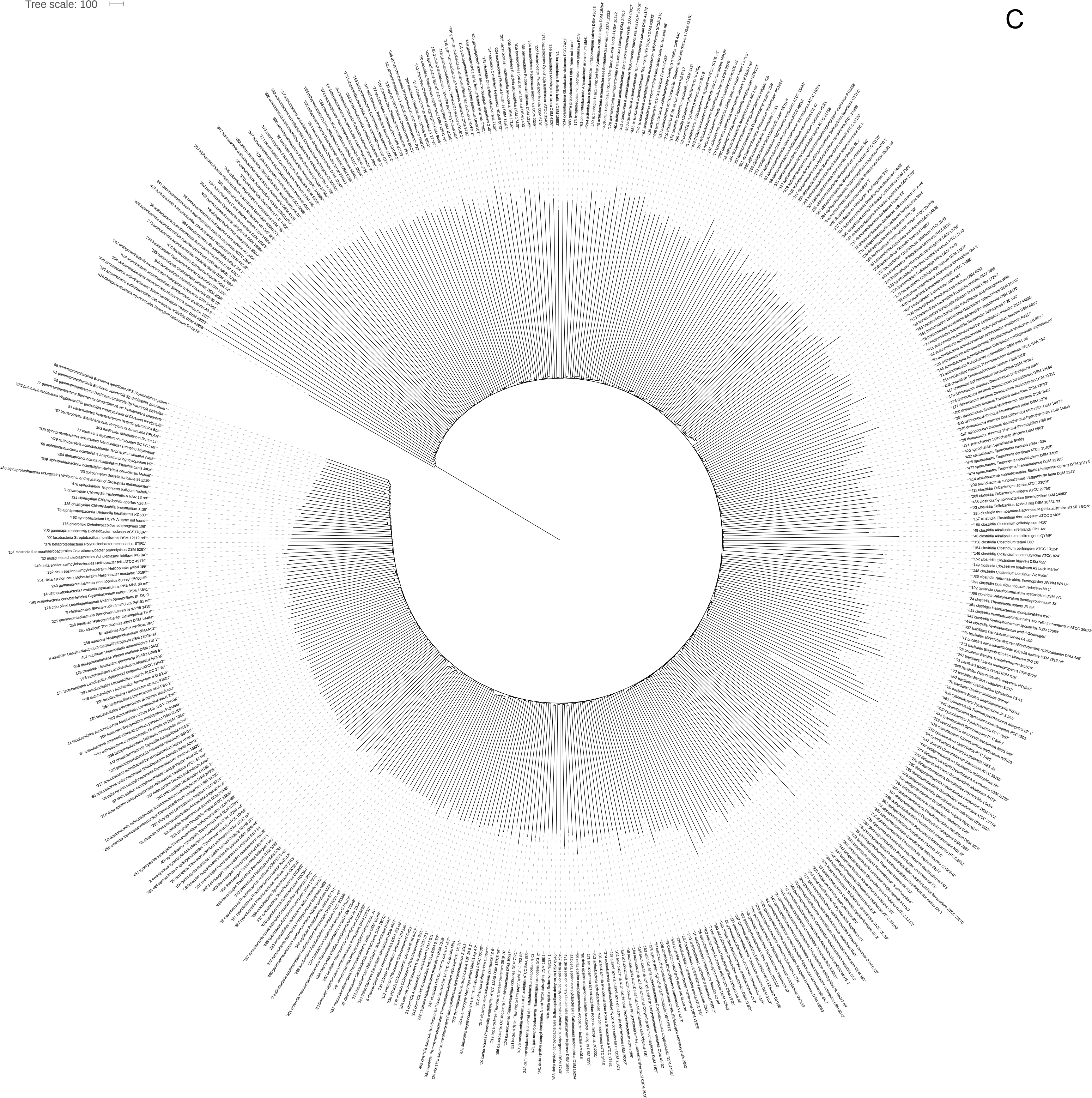

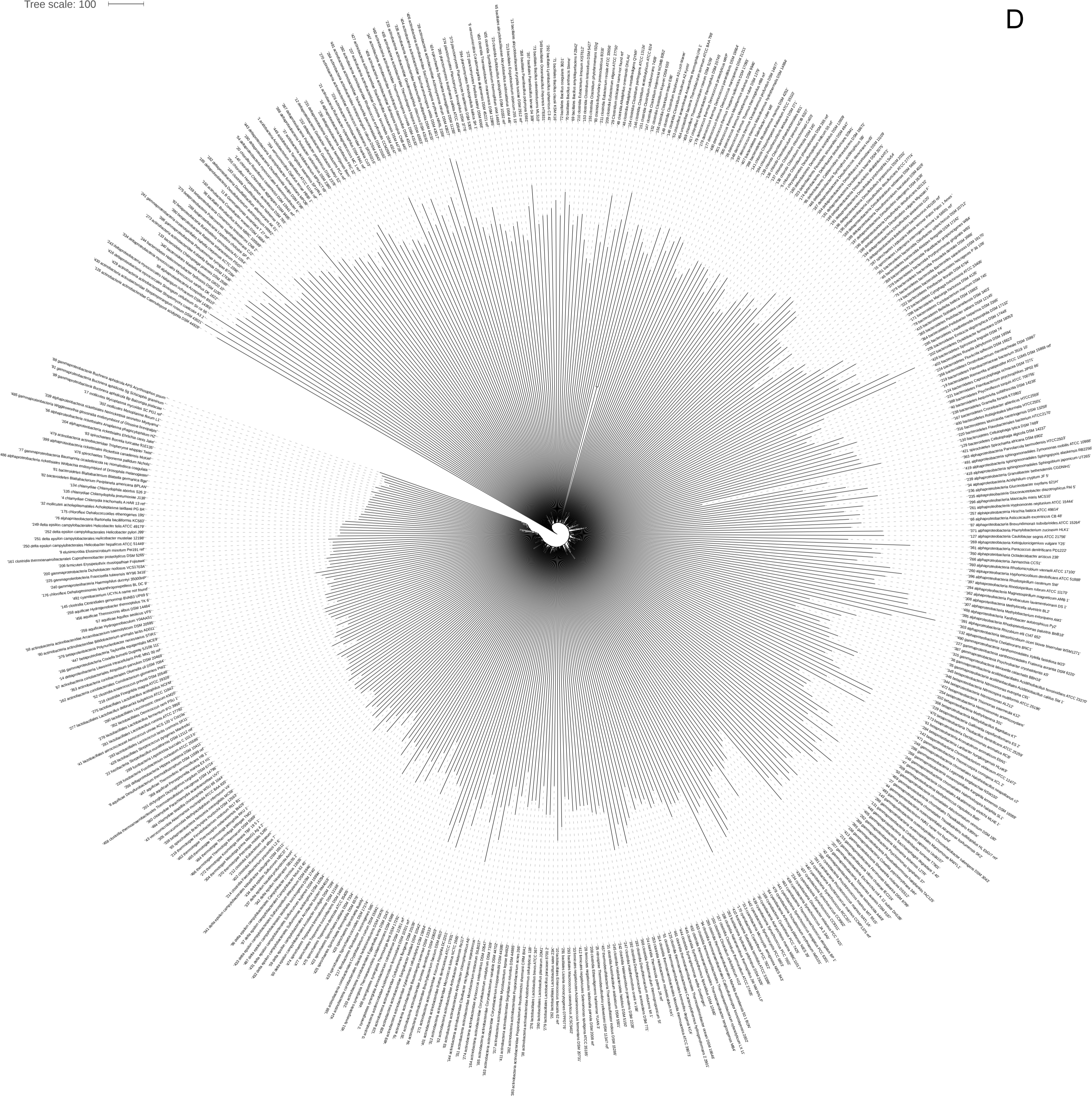

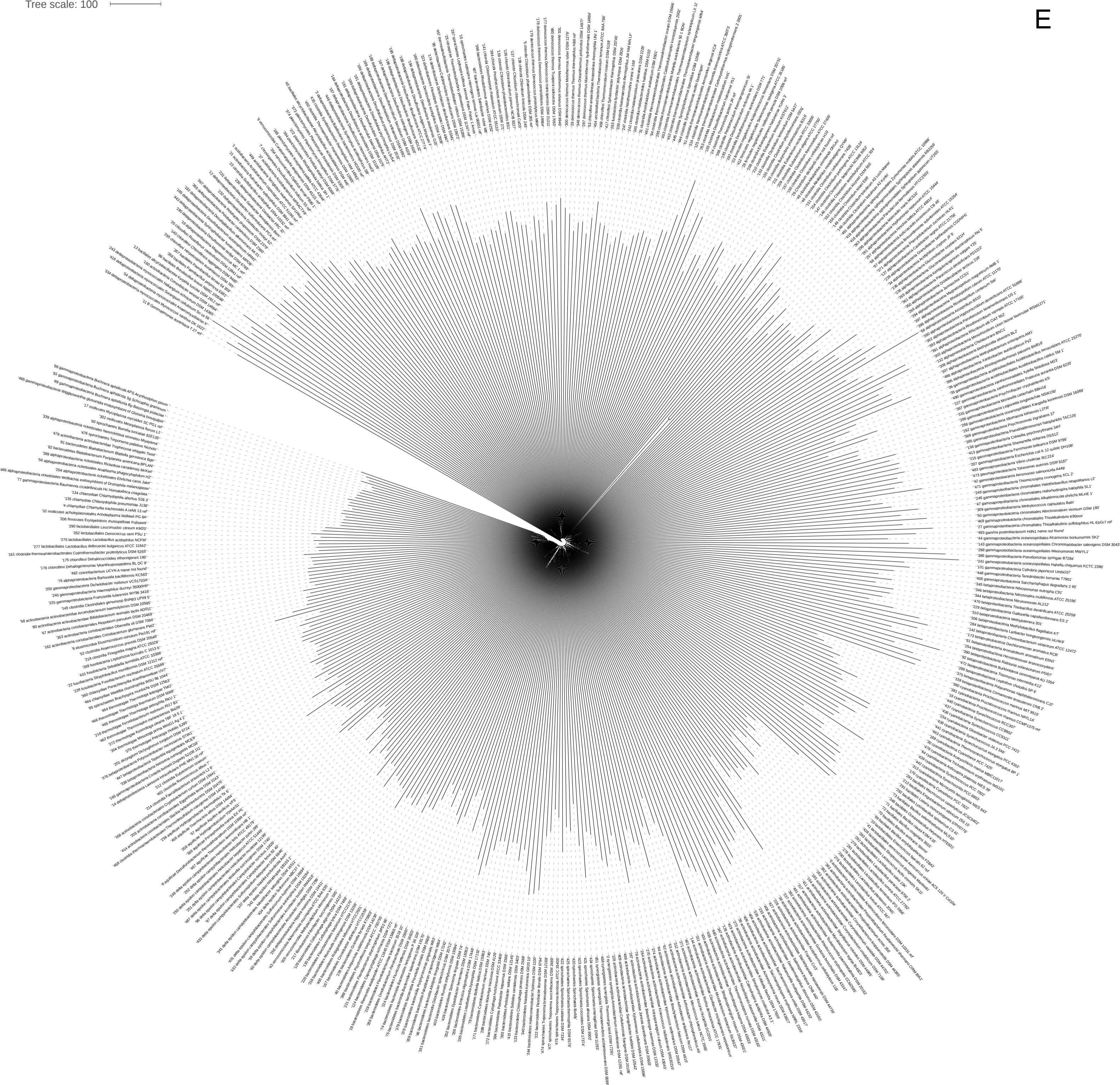

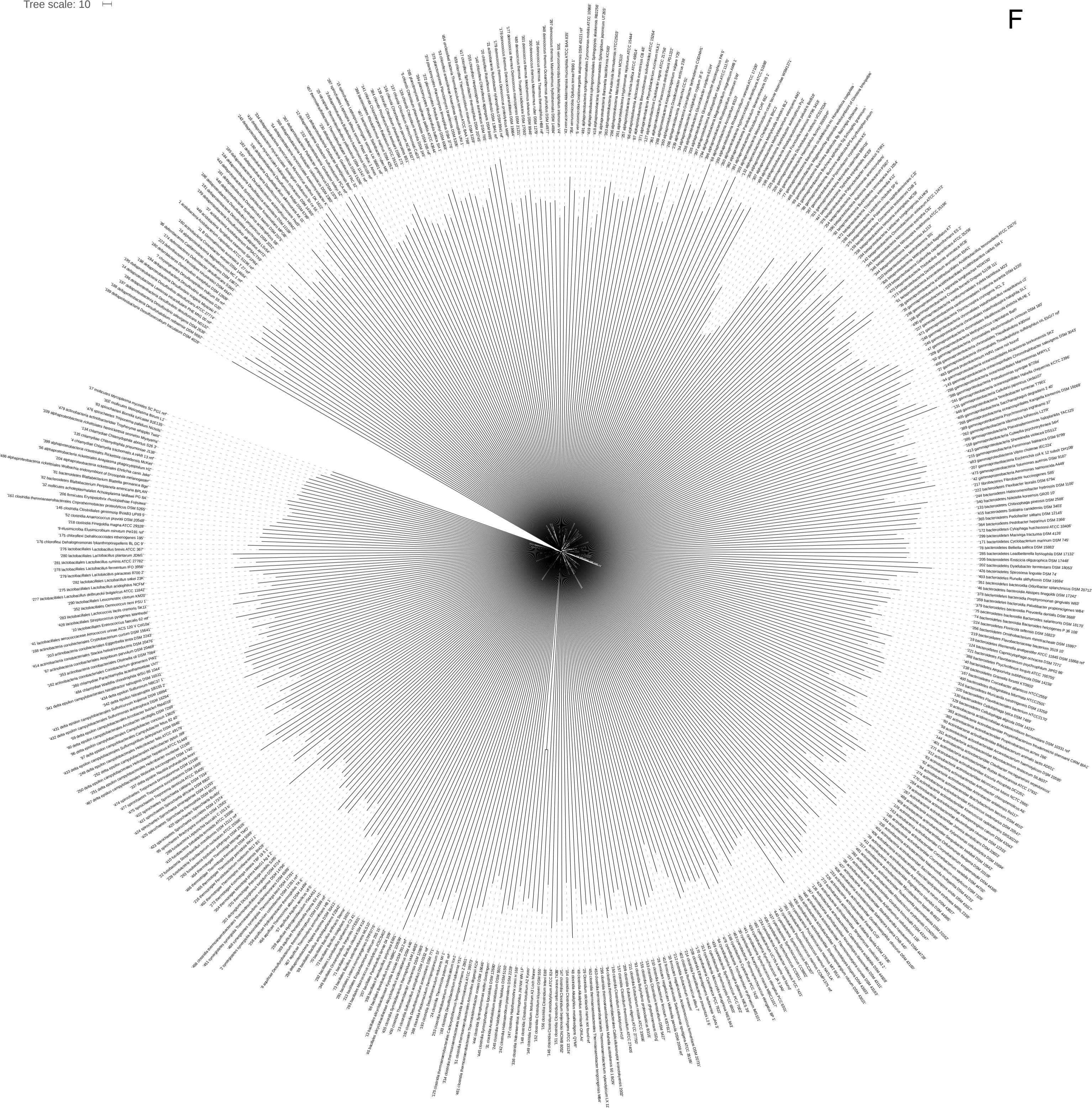

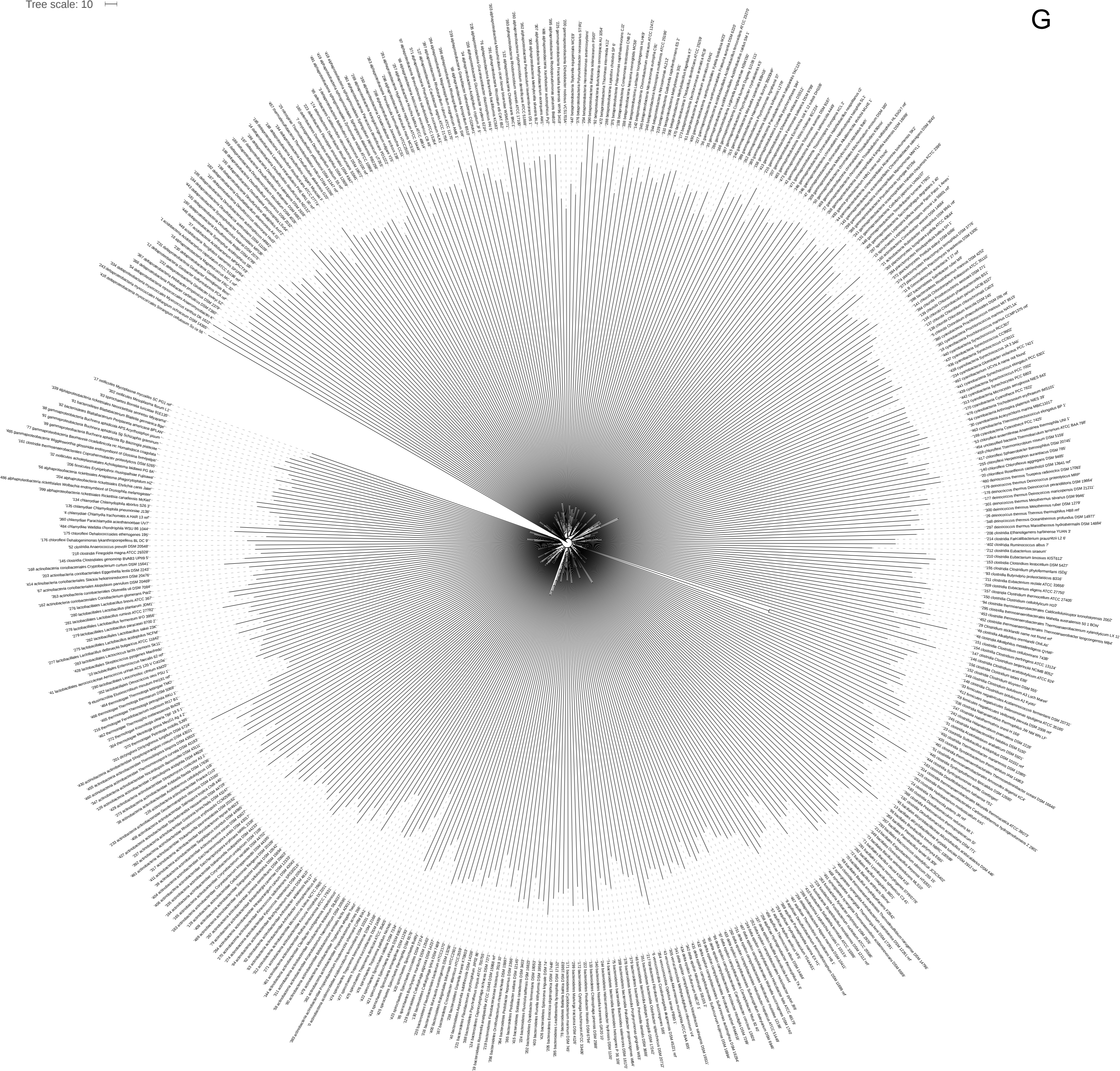

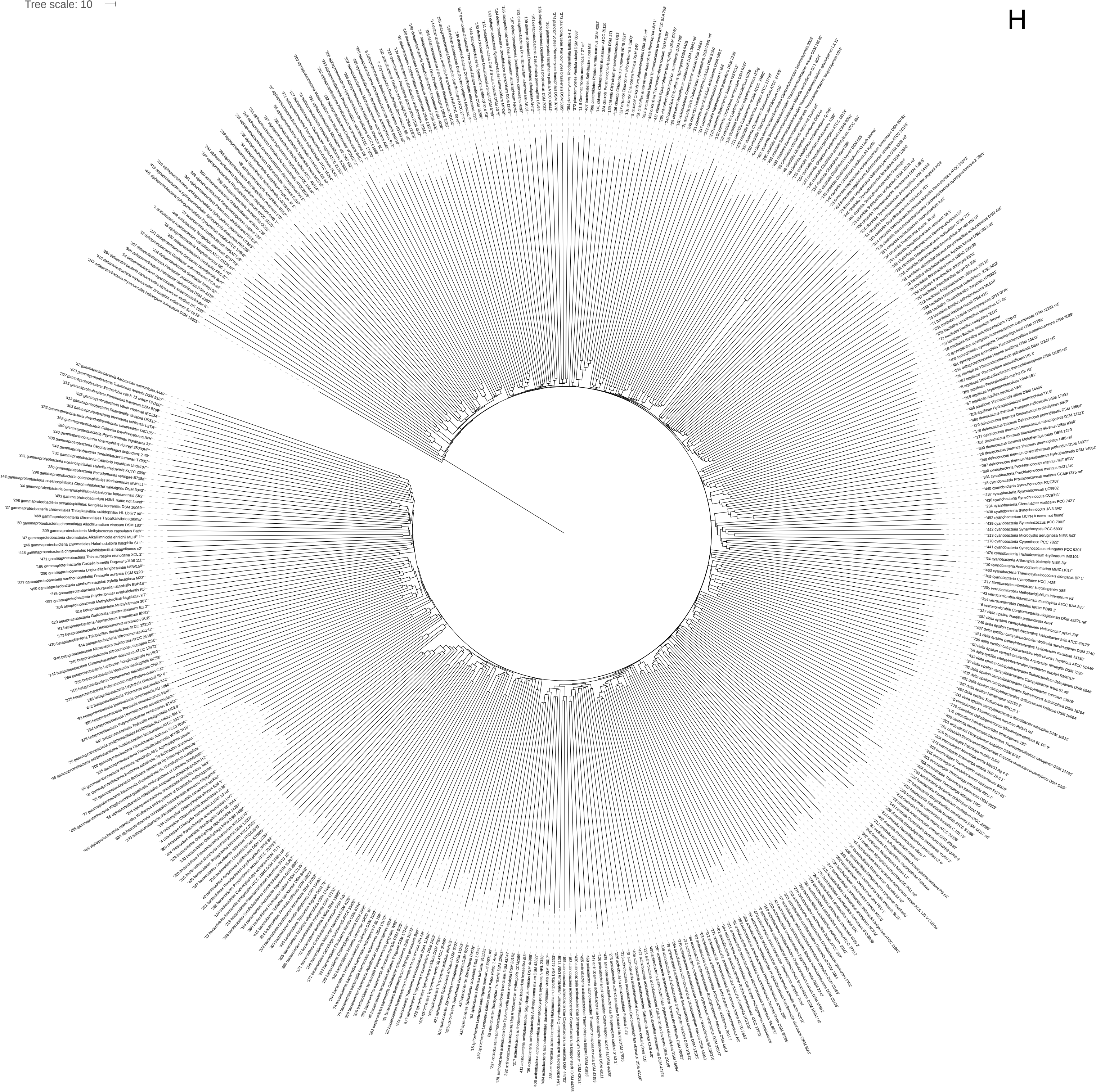

Supplement: S17 Fig — Trees rooted using ‘Root mid-point’ option in ITOL server. A) SW on raw 495 bacteria. Unfiltered and unpruned. B) SW on raw 445 bacteria. Unfiltered and pruned. C) SW on 445 bacteria. Filtered of mobile elements and pruned. D) SW on 445 bacteria. Filtered of mobile elements, pruned, and filtered by stability and conservation on o = 0. E) SW on 445 bacteria. Filtered of mobile elements, pruned, and filtered by stability and conservation on o = 1. F) SW on 445 bacteria. Filtered of mobile elements, pruned, and filtered by stability and conservation on o = 3. G) SW on 445 bacteria. Filtered of mobile elements, pruned, and filtered by stability and conservation on o = 5. H) SW on 445 bacteria. Filtered of mobile elements, pruned, and filtered by stability and conservation on o = 7. (PDF) [file pcbi.1004985.s017.pdf]

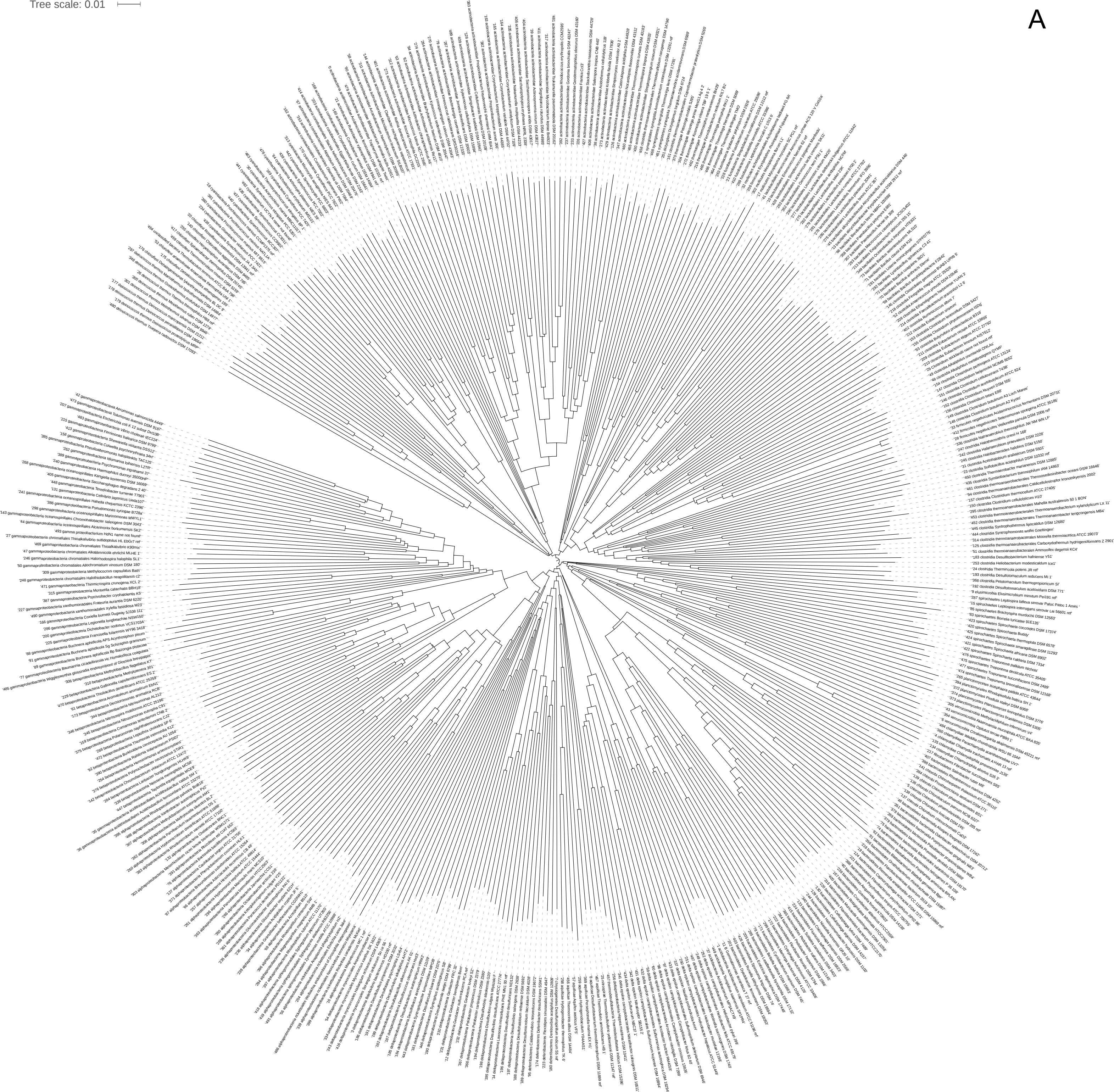

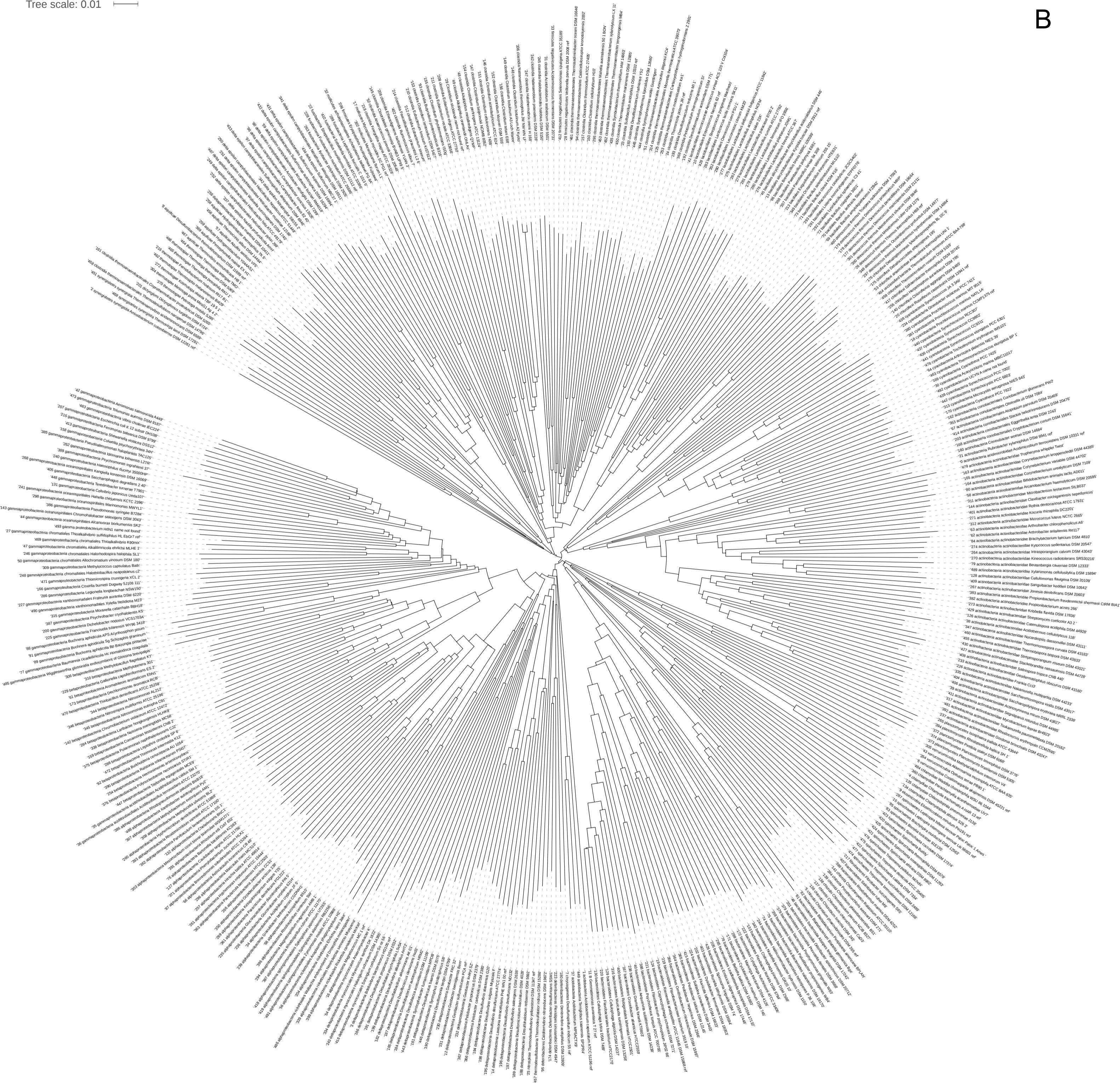

Supplement: S18 Fig — Trees rooted using ‘Root mid-point’ option in ITOL server. Only a partial set of trees was calculated due to the long run-time of the program. A) ALF on 445 bacteria. Filtered of mobile elements, pruned, and filtered by stability and conservation on o = 5. B) ALF on 445 bacteria. Filtered of mobile elements, pruned, and filtered by stability and conservation on o = 7. (PDF) [file pcbi.1004985.s018.pdf]

A.

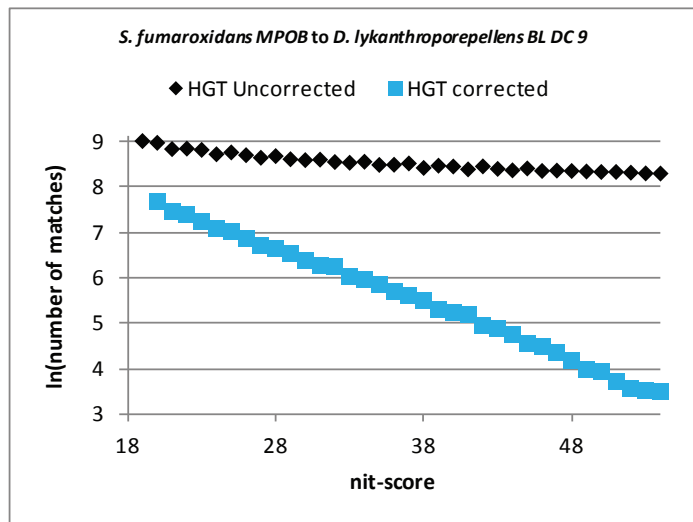

B.

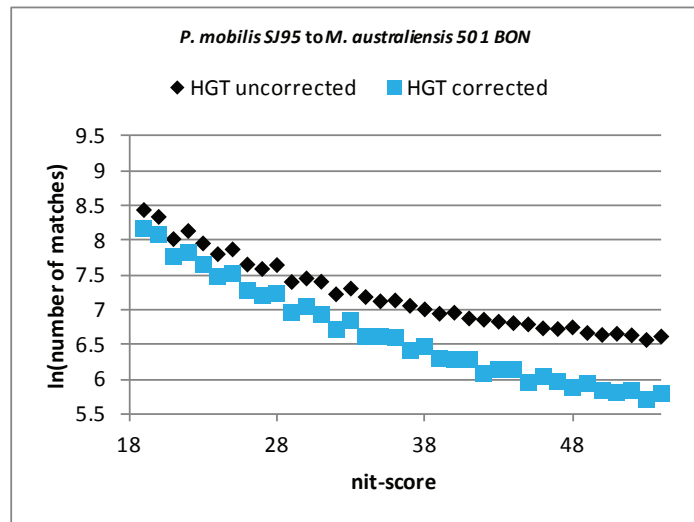

C.

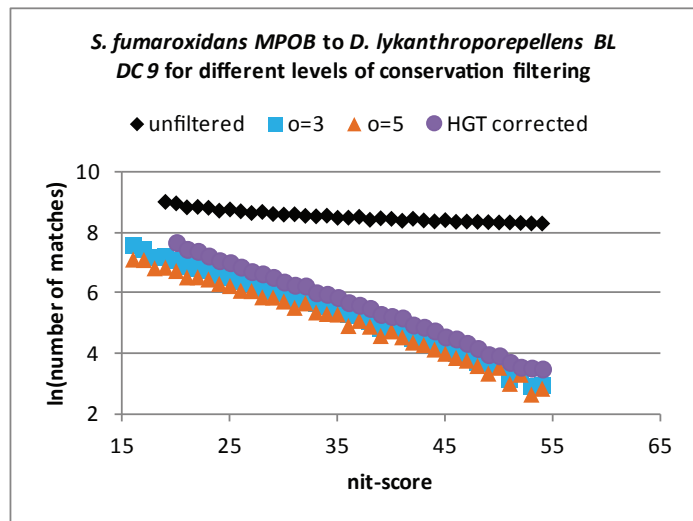

D.

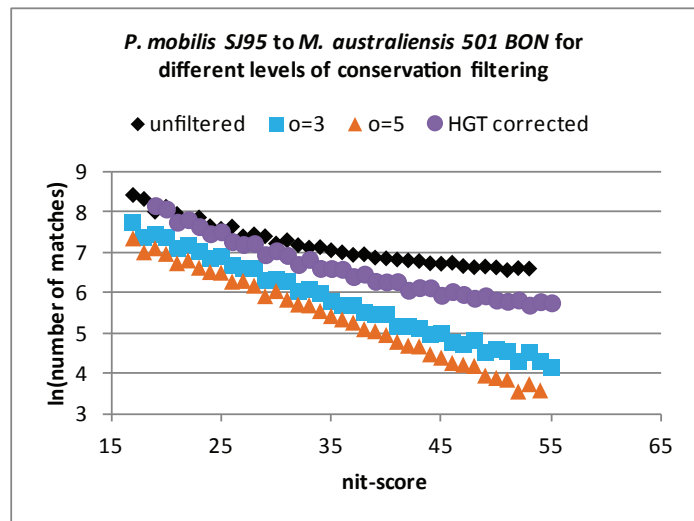

Supplement: S19 Fig — A) A pair sharing a single copy phage. B) A pair sharing large-scale transfer of proteins associated with adaptation to environment. C) For pair sharing phage, effect on plots that mobile-element filtering combined with conservation filtering have, compared to the pair-wise HGT correction. D) For a pair sharing adaptive proteins, effect on plots that mobile element filtering and conservation filtering have compared to the pair-wise HGT correction. (PDF) [file pcbi.1004985.s019.pdf]
