# Supplementary material for: Phylogeny Reconstruction with Alignment-Free Method That Corrects for Horizontal Gene Transfer
Source: PLoS Comput Biol. 2016 Jun 23;12(6):e1004985. doi: 10.1371/journal.pcbi.1004985 (PMC4918981; doi:10.1371/journal.pcbi.1004985)
Supplement: S1 Text — A) Pruned for archaea. B) Pruned for bacteria. (DOC) [file pcbi.1004985.s021.doc]

**A)**

Candidatus_Korarchaeum_cryptofilum_OPF8_uid58601

Nanoarchaeum_equitans_Kin4_M_uid58009

**B)**

Acidiphilium_cryptum_JF_5_uid58447

Acidobacterium_MP5ACTX9_uid50551

Aeromonas_salmonicida_A449_uid58631

Anaplasma_phagocytophilum_HZ_uid57951

Aster_yellows_witches_broom_phytoplasma_AYWB_uid58297

Azospirillum_B510_uid46085

Buchnera_aphidicola_Cc__Cinara_cedri__uid58579

Campylobacter_hominis_ATCC_BAA_381_uid58981

Candidatus_Accumulibacter_phosphatis_clade_IIA_UW_1_uid59207

Candidatus_Amoebophilus_asiaticus_5a2_uid58963

Candidatus_Azobacteroides_pseudotrichonymphae_genomovar__CFP2_uid59163

Candidatus_Blochmannia_floridanus_uid57999

Candidatus_Blochmannia_pennsylvanicus_BPEN_uid58329

Candidatus_Blochmannia_vafer_BVAF_uid62083

Candidatus_Carsonella_ruddii_uid58773

Candidatus_Cloacamonas_acidaminovorans_Evry_uid62959

Candidatus_Desulforudis_audaxviator_MP104C_uid59067

Candidatus_Hamiltonella_defensa_5AT__Acyrthosiphon_pisum__uid59289

Candidatus_Hodgkinia_cicadicola_Dsem_uid59311

Candidatus_Koribacter_versatilis_Ellin345_uid58479

Candidatus_Liberibacter_asiaticus_psy62_uid59227

Candidatus_Liberibacter_solanacearum_CLso_ZC1_uid61245

Candidatus_Nitrospira_defluvii_uid51175

Candidatus_Phytoplasma_australiense_uid61641

Candidatus_Phytoplasma_mali_uid59087

Candidatus_Protochlamydia_amoebophila_UWE25_uid58079

Candidatus_Puniceispirillum_marinum_IMCC1322_uid47081

Candidatus_Riesia_pediculicola_USDA_uid46841

Candidatus_Solibacter_usitatus_Ellin6076_uid58139

Candidatus_Sulcia_muelleri_CARI_uid52535

Candidatus_Sulcia_muelleri_SMDSEM_uid59393

Candidatus_Vesicomyosocius_okutanii_HA_uid59427

Candidatus_Zinderia_insecticola_CARI_uid52459

Cyanothece_PCC_7822_uid52547

Emticicia_oligotrophica_DSM_17448_uid177079

Gluconobacter_oxydans_621H_uid58239

Lactococcus_lactis_cremoris_SK11_uid57983

Macrococcus_caseolyticus_JCSC5402_uid59003

Mycoplasma_agalactiae_PG2_uid61619

Mycoplasma_arthritidis_158L3_1_uid58005

Mycoplasma_conjunctivae_uid59325

Mycoplasma_fermentans_JER_uid53543

Mycoplasma_gallisepticum_R_low__uid57993

Mycoplasma_genitalium_G37_uid57707

Mycoplasma_haemofelis_Langford_1_uid62461

Mycoplasma_hominis_ATCC_23114_uid41875

Mycoplasma_hyopneumoniae_232_uid58205

Mycoplasma_hyorhinis_HUB_1_uid51695

Mycoplasma_mobile_163K_uid58077

Mycoplasma_penetrans_HF_2_uid57729

Mycoplasma_pneumoniae_M129_uid57709

Mycoplasma_pulmonis_UAB_CTIP_uid61569

Mycoplasma_suis_KI3806_uid63665

Mycoplasma_synoviae_53_uid58061

Nitrosococcus_watsonii_C_113_uid50331

Orientia_tsutsugamushi_Boryong_uid61621

Polaromonas_naphthalenivorans_CJ2_uid58273

Porphyromonas_asaccharolytica_DSM_20707_uid66603

Runella_slithyformis_DSM_19594_uid68317

Spirosoma_linguale_DSM_74_uid43413

Synechococcus_PCC_7002_uid59137

Ureaplasma_parvum_serovar_3_ATCC_27815_uid58887

Zymomonas_mobilis_ATCC_10988_uid55403

uncultured_Termite_group_1_bacterium_phylotype_Rs_D17_uid59059
