# Supplementary material for: Phylogeny Reconstruction with Alignment-Free Method That Corrects for Horizontal Gene Transfer
Source: PLoS Comput Biol. 2016 Jun 23;12(6):e1004985. doi: 10.1371/journal.pcbi.1004985 (PMC4918981; doi:10.1371/journal.pcbi.1004985)
Supplement: S2 Text — A) Archaea. B) Bacteria. (DOCX) [file pcbi.1004985.s022.docx]

**A)**

Aeropyrum_pernix_K1_uid57757 Crenarchaeota: Thermoprotei

Archaeoglobus_fulgidus_DSM_4304_uid57717 Euryarchaeota: Archaeoglobi

Halalkalicoccus_jeotgali_B3_uid50305 Euryarchaeota: Halobacteria

Hyperthermus_butylicus_DSM_5456_uid57755 Crenarchaeota: Thermoprotei

Methanococcus_vannielii_SB_uid58767 Euryarchaeota: Methanococci

Methanomethylovorans_hollandica_DSM_15978_uid184864 Euryarchaeota: Methanomicrobia

Methanoregula_boonei_6A8_uid58815 Euryarchaeota: Methanomicrobia

Methanothermus_fervidus_DSM_2088_uid60167 Euryarchaeota: Methanobacteria

Pyrococcus_horikoshii_OT3_uid57753 Euryarchaeota: Thermococci

Thermofilum_pendens_Hrk_5_uid58563 Crenarchaeota: Thermoprotei

**B)**

Acidimicrobium_ferrooxidans_DSM_10331_uid59215 Actinobacteria

Acidobacterium_capsulatum_ATCC_51196_uid59127 Acidobacteria

Aminobacterium_colombiense_DSM_12261_uid47083 Synergistetes

Bdellovibrio_bacteriovorus_HD100_uid61595 Deltaproteobacteria

Chlamydia_trachomatis_A_HAR_13_uid58333 Chlamydia

Chlorobium_phaeovibrioides_DSM_265_uid58129 Chlorobi

Coraliomargarita_akajimensis_DSM_45221_uid47079 Verrucomicrobia

Desulfurispirillum_indicum_S5_uid45897 Chrysiogenetes

Desulfurobacterium_thermolithotrophum_DSM_11699_uid63405 Aquificae

Elusimicrobium_minutum_Pei191_uid58949 Elusimicrobia

Enterococcus_faecalis_62_uid159663 Firmicutes: Lactobacillales

Gemmatimonas_aurantiaca_T_27_uid58813 Gemmatimonas

Geobacter_sulfurreducens_PCA_uid57743 Deltaprotoebacteria

Kyrpidia_tusciae_DSM_2912_uid48361 Firmicutes: Bacillales

Lawsonia_intracellularis_PHE_MN1_00_uid61575 Deltaproteobacteria

Leptospira_interrogans_serovar_Lai_56601_uid57881 Spirochaetes

Magnetococcus_MC_1_uid57833 Alphaproteobacteria

Mycoplasma_mycoides_SC_PG1_uid58031 Mollicutes

Prochlorococcus_marinus_CCMP1375_uid57995 Cyanobacteria

Riemerella_anatipestifer_ATCC_11845___DSM_15868_uid159857 Bacteroidetes

Roseiflexus_castenholzii_DSM_13941_uid58287 Chloroflexi

Rubrobacter_xylanophilus_DSM_9941_uid58057 Actinobacteria

Streptobacillus_moniliformis_DSM_12112_uid41863 Fusobacteria

Sulfobacillus_acidophilus_DSM_10332_uid88061 Firmicutes: Clostridia

Thermincola_potens_JR_uid48823 Firmicutes: Clostridia

Thermodesulfovibrio_yellowstonii_DSM_11347_uid59257 Nitrospirae

Thermus_thermophilus_HB8_uid58223 Deinococcus-Thermus

Thioalkalivibrio_sulfidophilus_HL_EbGr7_uid59179 Gammaproteobacteria

Veillonella_parvula_DSM_2008_uid41927 Firmicutes: Negativicutes

_Clostridium__sticklandii_uid59585 Firmicutes: Clostridia
